# Synthesis and Characterization of a Series of (PP^R^P)Co Complexes and Comparison of their Electronic Properties and Catalytic Activities

**DOI:** 10.1021/acs.organomet.6c00131

**Published:** 2026-06-19

**Authors:** Maria C. Seith, Matthew C. Fitzsimmons, Hanan A. Muhammad, Chris A. Nieto, Curtis E. Moore, Luke C. Lewis, Christine M. Thomas

**Affiliations:** Department of Chemistry and Biochemistry, 201912 The Ohio State University, 100 W. 18th Ave, Columbus, Ohio 43210, United States

## Abstract

The substituent on the central cyclic diamido phosphorus fragment of a tridentate bis­(phosphine)­pincer ligand framework (PP^R^P) was varied systematically (R = NEt_2_, N^*i*^Pr_2_, OEt, O^*i*^Pr, OCH_2_CF_3_, OCH­(CF_3_)_2_, (−)-menthoxide, Me, CF_3_) to tune the steric profiles as well as the σ-donor and π-acceptor properties of the pincer ligands. A series of (PP^R^P)­CoI_2_ compounds were synthesized and characterized using ^1^H NMR and EPR spectroscopy and single crystal X-ray diffraction. The differences in electron density at the cobalt centers imparted by the varying ligand substituents were evaluated using cyclic voltammetry of the (PP^R^P)­CoI_2_ compounds and calculated ν­(CO) stretching frequencies of hypothetical (PP^R^P)­Co­(CO)­(H) compounds. In an attempt to deconvolute variations in σ-donor and π-acceptor properties, the ^1^ *J*_P–Se_ coupling constants of a series of phosphine selenide compounds were used to evaluate basicity as a direct measure of σ-donor strength. The catalytic activities of the (PP^R^P)­CoI_2_ compounds for the hydroboration of styrene and α-methylstyrene at room temperature were evaluated quantitatively through kinetic studies to determine the rate constant (*k*_*obs*_) for each precatalyst. In general, it was found that compounds featuring a less electron-rich cobalt center were more active alkene hydroboration catalysts, with steric properties playing a more prominent role with the bulkier 1,1′-disubstituted alkene substrate.

## Graphical abstract

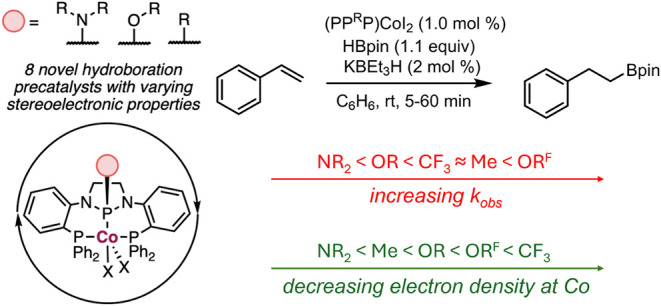

## Introduction

In recent years, the field of transition metal catalysis has seen rapidly increasing efforts to pivot from precious metal catalysts to first-row transition metal catalysts due to their lower cost, higher crustal abundance, and diminished economic impact.^1–8^ With this goal in mind, researchers have sought to design ligands that enforce a low-spin electronic configuration to drive traditional two-electron pathways and disfavor single-electron transfer reactions.^9,10^ Tridentate pincer ligands are particularly common in Earth-abundant metal catalysis, with this ligand class offering rigidity/stability while containing multiple sites for derivatization to tune the steric and electronic properties of the ligand to enhance reactivity and/or selectivity.^10,11^ The prevailing strategy in the design of first-row transition metal pincer catalysts is the incorporation of strong σ-donors to enforce a strong ligand field and low-spin configuration; however, π-acceptors can achieve the same effect while potentially promoting different reactivity patterns.

In this context we have turned our attention to pincer ligands featuring π-accepting cyclic diamidophosphine, diamidophosphite, or triamidophosphine moieties in the central position of a tridentate bis­(phosphine) pincer ligand. Such fragments have found widespread applications in catalysis including asymmetric catalysis,^12–29^ but there are relatively few examples of pincer ligands that incorporate these fragments and few reports of their coordination to first-row transition metals.^30–43^ The most common pincer ligand designs include central aryl, amine/amide, or pyridine functionalities.^10,44–47^ In contrast, pincer ligands with a central tricoordinate phosphorus atom are much less common.^36,48–61^

Our group initially reported (PP^X^P) (X = Cl, H) pincer ligands composed of a central N-heterocyclic chloro- or hydrophosphine (PP^R^P) functionality with two appended phosphine side arms.^65,66^ These ligands were coordinated to Co^II^ salts, affording (PP^Cl^P)­CoCl_2_ (**1**) and (PP^H^P)­CoI_2_ (**2**), which were found to be active precatalysts for the hydroboration and hydrogenation of terminal alkenes under mild conditions in the presence of KBEt_3_H as an activator (Figure 1).^62,63^ While these catalysts showed promising activity toward a variety of terminal alkene substrates, they were deactivated upon reaction completion and failed to demonstrate activity toward substituted alkene substrates. These shortcomings were attributed to the irreversible formation of a catalytically inactive dimerized species through the loss of the central phosphorus substituent (Cl or H) upon depletion of the alkene substrate or in instances of slow alkene binding.^62,63,67^ To prevent dimerization, a more tightly bound central phosphorus substituent, CF_3_, was installed in (PP^CF3^P)­CoI_2_ (**3**), resulting in a ∼100-fold increase in catalytic styrene hydroboration activity compared to **1**.^64^ Further, **3** could access a much wider substrate scope including internal and 1,1′-disubstituted alkenes and, since it did not undergo deactivation via dimerization as seen with **1** and **2**, catalytic reaction mixtures using **3** could be recharged with substrates upon full consumption, allowing for a total of three rounds of catalysis over 72 h without loss in activity.^64^ The dramatic enhancement of reactivity upon altering the central phosphorus substituent prompted further investigation into the role of the identity of the central phosphorus substituent in (PP^R^P) ligands in promoting catalysis. Herein, we report a series of eight new (PP^R^P)­CoI_2_ derivatives, covering a scope of substituents with different electronic and steric properties (R = NEt_2_, N^*i*^Pr_2_, OEt, O^*i*^Pr, OCH_2_CF_3_, OCH­(CF_3_)_2_, (−)-menthoxide, and Me), and systematically study the donor/acceptor properties of the ligands and their impact on catalytic activity.

**Figure 1:**
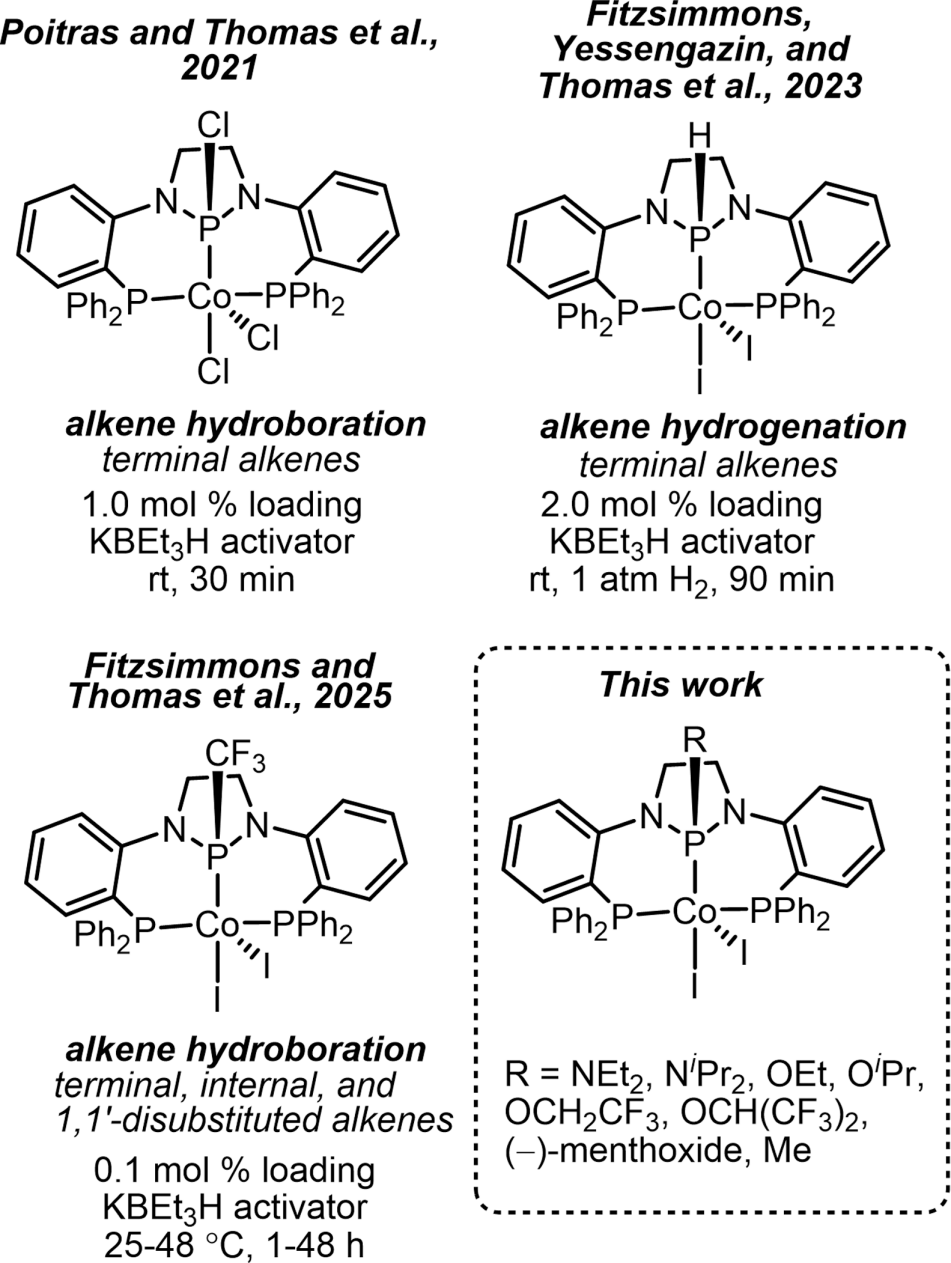
Previously reported (PP^Cl^P)­CoCl_2_ (**1**), (PP^H^P)­CoI_2_ (**2**), and (PP^CF3^P)­CoI_2_ (**3**) complexes that are active precatalysts for alkene hydroboration and hydrogenation.^62–64^

## Results and Discussion

### Synthesis and Characterization of Ligands **5**–**12** and Cobalt Complexes **13**–**20**

The previously reported chlorophosphine precursor (PP^Cl^P) (**4**) served as a versatile synthon to allow for further modification at the central phosphorus site.^15^ The synthesis of the amide-substituted compounds (PP^NR2^P) (R = Et (**5**) and ^*i*^Pr (**6**)) was achieved via replacement of the phosphorus-bound chloride via an S_N_2 reaction between **4** and R_2_NH, where R_2_NH could be used in excess to also serve as a base or NEt_3_ could be added (Scheme 1). The alkoxide-substituted derivatives (PP^OR^P) (R = OEt (**7**), O^*i*^Pr (**8**), OCH_2_CF_3_ (**9**), OCH­(CF_3_)_2_ (**10**), (−)-menthoxide (**11**)) were synthesized via the addition of ROH to **4** in the presence of excess NEt_3_ (Scheme 1). Alternatively, the addition of MeMgBr to **4** generated the P-methyl-substituted (PP^Me^P) ligand (**12**) (Scheme 1). Ligands **5**–**12** were isolated as white powders in 63–96% yield.

**Scheme 1:**
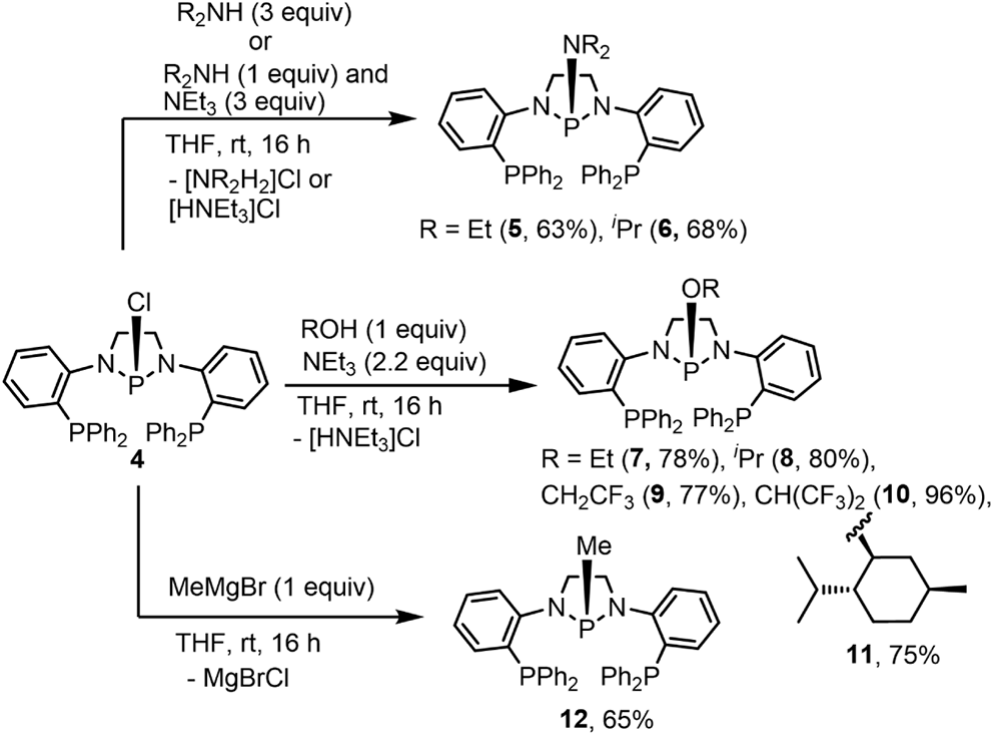
Synthesis of (PP^R^P) Ligand Derivatives **5**–**12**

^31^P­{^1^H} NMR spectroscopy was particularly useful in probing the electronic character of the central phosphorus, with the chemical shift of the central phosphorus serving as a direct indicator of the electron density at the phosphorus atom: a more electron-rich or shielded central phosphorus atom should exhibit a more upfield signal, while a more electron-poor or deshielded central phosphorus atom should have a more downfield signal. Table 1 contains the central phosphorus chemical shifts observed in the ^31^P­{^1^H} NMR spectra for the (PP^Cl^P), (PP^H^P), and (PP^CF3^P) ligands, as well as compounds **5**–**12**.^9,13,15^ The chemical shifts within the series generally follow the expected trend, with the most downfield chemical shift observed for the halide-substituted (PP^Cl^P) derivative **4**, followed by the alkoxide-substituted derivatives **7**–**11**, then the amide-substituted derivatives **5** and **6**. The chemical shift of the more electron-rich N^*i*^Pr_2_ derivative **6** is more upfield than that of the NEt_2_ analogue **5** and the ^31^P chemical shift of the alkoxide-substituted derivatives increases in the order OEt < O^*i*^Pr ∼ (−)-menthoxide < OCH_2_CF_3_ < OCH­(CF_3_)_2_, in general agreement with expected basicity trends. The Me-substituted derivative **12** has a more downfield chemical shift than the amide derivatives **5**–**6**, the CF_3_ analogue, and the (PP^H^P) derivative, demonstrating that straightforward trends in ^31^P chemical shift are not universally held.

**Table 1:** ^31^P­{^1^H} NMR Chemical Shifts of the Central Phosphorus Atom of the (PP^R^P) Ligands

**(PP** ^**R**^ **P)**	^**31**^ **P{** ^**1**^ **H} NMR chemical shift (ppm)** ^a^
(PP^NEt2^P) (**5**)	104.7
(PP^N*i*Pr2^P) (**6**)	93.7
(PP^OEt^P) (**7**)	118.7
(PP^O*i*Pr^P) (**8**)	122.4
(PP^OCH2CF3^P) (**9**)	125.7
(PP^OCH(CF3)2^P) (**10**)	129.9
(PP^menthoxide^P) (**11**)	122.7
(PP^Me^P) (**12**)	107.7
(PP^CF3^P)^64^	75.0
(PP^Cl^P) (**4**)^66^	147.9
(PP^H^P)^65^	51.6

a ^31^P­{^1^H} chemical shifts were recorded in C_6_D_6_.

Ligand derivatives **5**–**12** were metalated with CoI_2_ in THF at room temperature to generate (PP^R^P)­CoI_2_ compounds **13**–**20** (Scheme 2). Complexes **13**–**17** and **20** are insoluble in THF and precipitated from the reaction solution and are collected via filtration. Compounds **18** and **19** are soluble in THF, so the reaction mixture was layered with pentane and stored at −35 °C to yield crystalline product. Cobalt complexes **13**–**20** are low-spin d^7^ Co^II^ species with solution magnetic moments ranging from 1.8 to 2.5 μ_B_. The ^1^H NMR spectra of these compounds feature slightly fewer than the expected number (based on symmetry) of broad and paramagnetically shifted resonances within a relatively small chemical shift window of 0–10 ppm. There are no discernible ^31^P­{^1^H} NMR or ^19^F NMR (where applicable) signals for **13**–**20**, consistent with their paramagnetism.

**Scheme 2:**
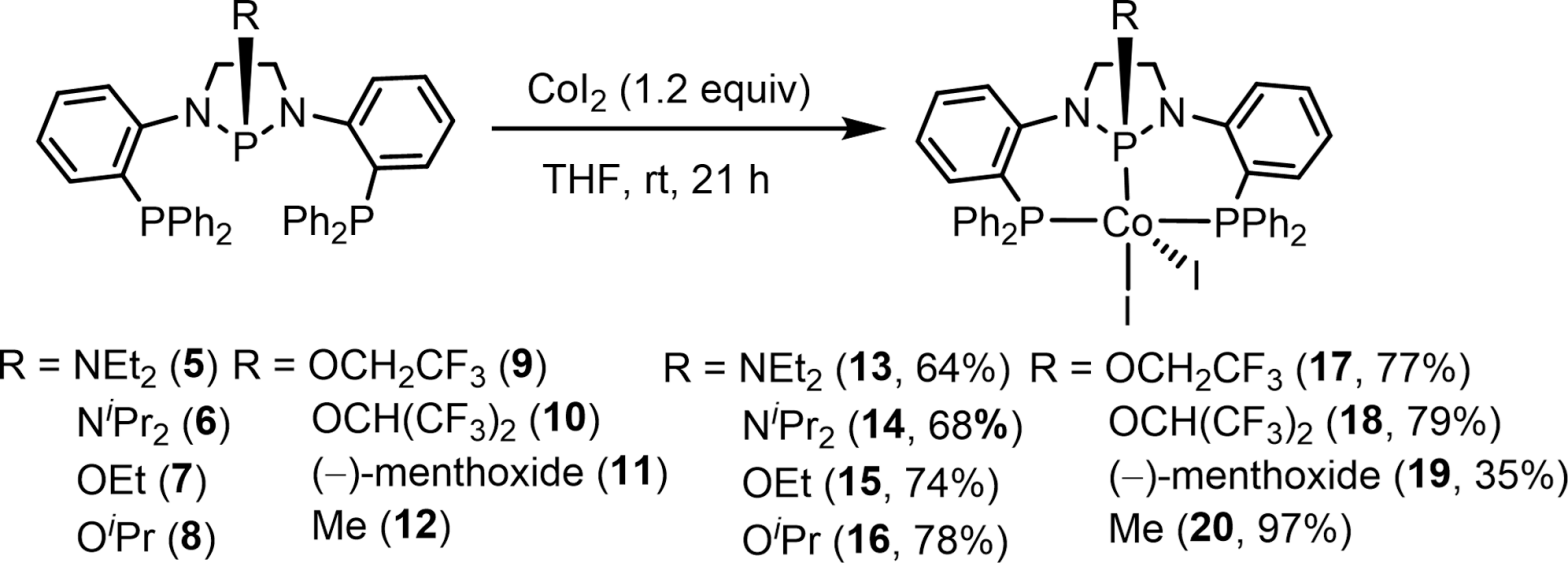
Metalation of Ligands **5**–**12** to Generate Cobalt Complexes **13**–**20**

The EPR spectra of **13**–**20** and **3** were collected at 40 K in frozen THF solutions with the goal of extracting hyperfine coupling parameters that would provide more insight into the extent of Co–P^R^ interactions as a function of the central phosphorus substituent. All nine spectra feature axial signals with varying g values *g*_*1*_ = *g*_*2*_ = 2.20–2.21 and *g*_3_ = 2.03–2.04 (*g*_*iso*_ ∼ 2.14–2.15 ), consistent with a low-spin, S = 1/2, d^7^ configuration on the cobalt center. The spectra feature discernible hyperfine coupling to the ^59^Co (*I* = 7/2) nucleus, along with superhyperfine coupling with varying degrees of complexity and resolution. Since this additional hyperfine coupling could be the result of coupling to the central ^31^P nucleus (*I* = 1/2), and/or the two equivalent ^31^P nuclei of the phosphorus side arms, the low-temperature spectra could not be confidently simulated using a single set of parameters that would allow conclusions to be drawn about the magnitude of coupling constants to the ^59^Co or the ^31^P nucleus of interest. Moreover, with a square pyramidal geometry, the SOMO is expected to be the d_z2_ orbital, which may lead to significant hyperfine coupling to the axial ^127^I nucleus (*I* = 5/2), further complicating and broadening the spectra. In order to simplify the complicated EPR spectra and assist in the determination of the ^59^Co hyperfine coupling constant, samples of **13**, **15–18**, and **20** were also collected at room temperature in THF solution, revealing isotropic signals centered at *g*_*iso*_ = 2.13–2.15 (). Simulation of these isotropic signals allowed for determination of *A*_*iso*_ values corresponding to hyperfine coupling to ^59^Co; however, the *A*_*iso*_ values were similar among **13**, **15–18**, and **20** (*A*_*iso*_ = 89–97 MHz) and did not appear to correlate with any other trends related to structural features or electron density at the cobalt center (*vide infra*). To further resolve the *g* tensor and hyperfine coupling parameters of the ^59^Co and ligands and how they relate to reactivity, multifrequency pulsed EPR techniques will be required, but are beyond the scope of this work.

### Structural Comparison of Cobalt Complexes **13**–**20**

Single crystals of **13**–**20** were grown and their solid-state structures determined using single-crystal X-ray diffraction analysis (Table 2, Figure 2). All eight compounds feature a square pyramidal geometry about the cobalt center (τ_5_ = 0–0.36),^68^ as was previously reported for **1**–**3**.^63,64,69^ The Co–P1 distances associated with the central phosphorus atom provide information about the extent of Co–P bonding and show general trends as the phosphorus substituent is varied (Table 2).

**Figure 2:**
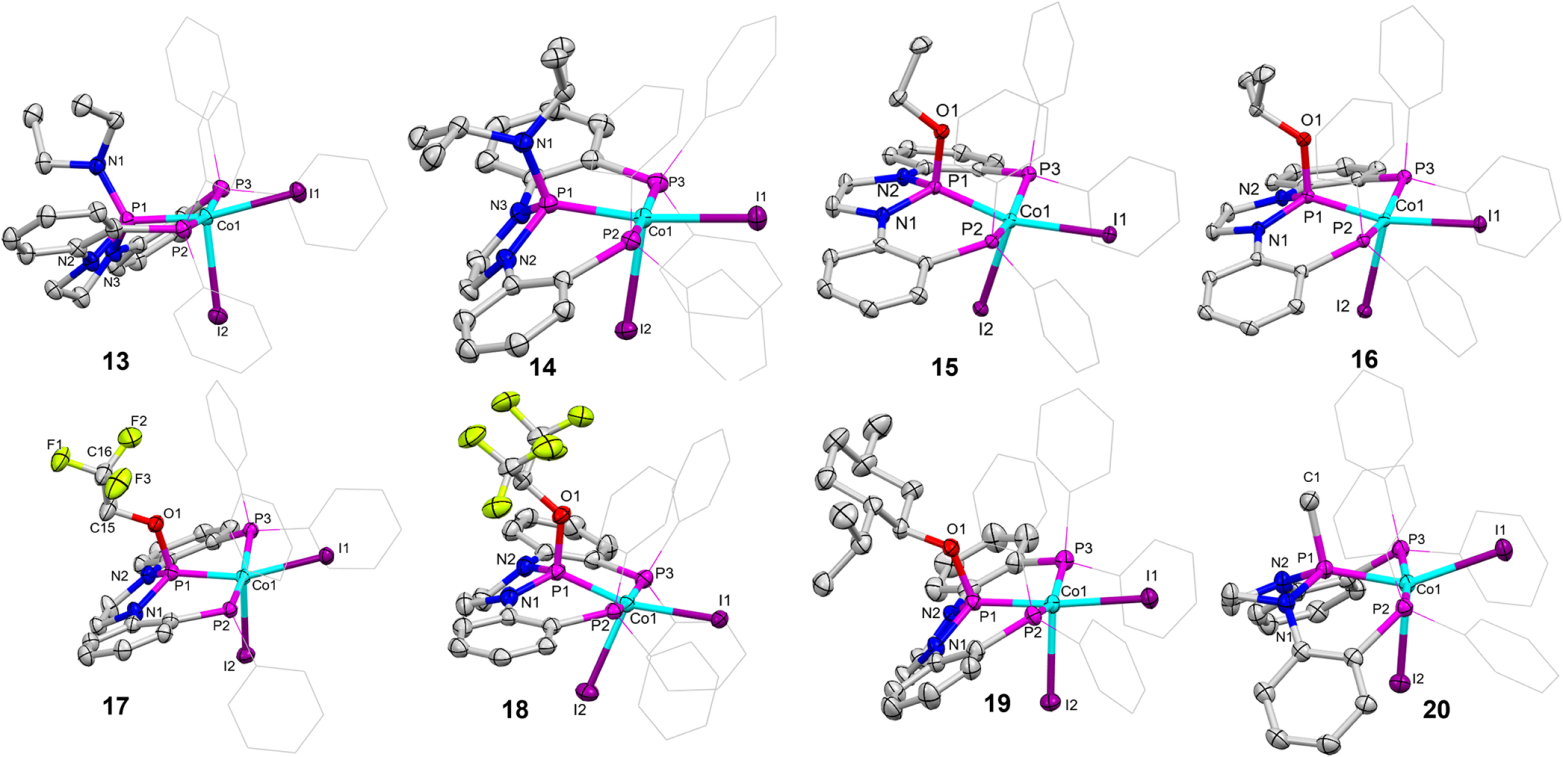
Displacement ellipsoid (50%) representations of **13**–**20**. H atoms and solvent molecules are omitted for clarity. Only one of the two independent molecules in the asymmetric units of **17**, **18**, and **19** is shown. The CF_3_ group in **17** is disordered over two positions, but only one is shown for simplicity.

**Table 2:** Co–P1 and P1–X (X = O, N, C) Distances, τ_5_ Parameters,^68^ and Percent Buried Volume (%V_bur_)^70^ Values for **13**–**20** and **3** Obtained from Single Crystal X-ray Diffraction Data^a^

**Compound**	**τ**_**5**_	**Co–P1 (Å)**	**P1–X (Å)**	**%V**_**bur**_
(PP^NEt2^P)CoI_2_ (**13**)	0.00	2.1329(14)	1.660(5)	72.2
(PP^N*i*Pr2^P)CoI_2_ (**14**)	0.04	2.1523(9)	1.661(3)	73.0
(PP^OEt^P)CoI_2_ (**15**)	0.02	2.1077(5)	1.6021(12)	70.3
(PP^O*i*Pr^P)CoI_2_ (**16**)	0.00	2.1102(4)	1.5955(10)	71.2
(PP^OCH2CF3^P)CoI_2_ (**17**)	0.11	2.0960(8)	1.622(2)	71.0
	0.26	2.0951(8)	1.615(2)	
(PP^OCH(CF3)2^P)CoI_2_ (**18**)	0.17	2.0965(7)	1.6454(18)	70.3
	0.04	2.0967(7)	1.6486(19)	
(PP^menthoxide^P)CoI_2_ (**19**)	0.30	2.1148(14)	1.599(4)	71.1
	0.18	2.1132(15)	1.602(4)	
(PP^Me^P)CoI_2_ (**20**)	0.36	2.1061(5)	1.816(2)	70.1
(PP^CF3^P)CoI_2_ (**3**)^64^	0.04	2.1034(6)	1.903(3)	71.0

a In cases where two independent molecules are present in the asymmetric unit, the distances in both molecules are listed.

Amide-substituted compounds **13** and **14** exhibit the longest Co–P1 bonds, while compounds **17** and **18**, which possess partially fluorinated alkoxide substituents, feature the shortest Co–P1 bond distances. The Co–P1 bonds in **17** and **18** are shorter than the Co–P1 bonds in the nonfluorinated alkoxide analogues **15**, **16**, and **19**. Given the inductively electron-withdrawing nature of the fluorinated substituents in **17** and **18**, it is unlikely that the shorter Co–P1 bonds are the result of stronger σ-donation from phosphorus to cobalt but, instead, result from increased π-backbonding from the cobalt center to the central phosphorus atom as the phosphorus atom becomes more electron-deficient.

This hypothesis is supported by the elongation of the P1–O1 bonds in **17** and **18** compared to **15**, **16**, and **19** due to increased π-backbonding into the P–O σ* orbital (Table 2). Increased cobalt-to-phosphorus π-backbonding also explains the shorter Co–P1 distances observed for the alkoxide-substituted compounds **15**–**19** compared to the amide-substituted compounds **13** and **14**, as the oxygen-containing substituents are expected to be more electronegative than the nitrogen-containing substituents, lowering the energy of the P–X σ* orbitals and enhancing π-backbonding.

The Co–P1 bond length in the methyl-substituted complex **20** (2.1061(5) Å) is in the same range as that of the alkoxide-substituted complexes **15**–**19**. This could be the result of less cobalt-to-phosphorus π-backbonding in the methyl derivative **20** or enhanced phosphorus-to-cobalt σ-donation resulting from the more electron-donating methyl group. The Co–P1 bond distance in **20** is also essentially identical to that in the previously reported trifluoromethyl-substituted compound **3** (2.1034(6) Å).^64^

The steric differences between the pincer ligands in **13**–**20** and **3** can be quantified using the percent buried volume (%V_bur_)^70^ values calculated using the structural coordinates from the solid-state structures (Table 2). The two NR_2_-substituted ligands **5** and **6** have the largest %V_bur_ as a result of the two substituents on the amide. The OR-substituted compounds **7**–**11**, the Me-substituted compound **12**, and (PP^CF3^P) all have similar %V_bur_ values within 1% of each other. Generally, the replacement of H with F leads to an increase in steric crowding, as observed when comparing the ligands in **20** and **3** and the ligands in **15** and **17**. Although the %V_bur_ values indicate little variation in the amount of space around the cobalt center occupied by the ligands in **13**–**20** and **3**, these values do not take into account steric clashes between ligand substituents or between ligand substituents and approaching organic substrates, which are important factors in catalytic reactions (*vide infra*).

### Comparison of Redox Behavior of Cobalt Complexes **13**–**20**

Cyclic voltammetry was used to further probe and compare the electronic properties of the cobalt centers within the series of (PP^R^P)­CoI_2_ compounds **13**–**20** and **3** (Figure 3). Two irreversible reduction events are evident in the cyclic voltammograms (CVs) of all nine complexes, denoted herein as *E*_*pc1*_ and *E*_*pc2*_. We assign the irreversibility of both features to the facile dissociation of iodide ligands upon reduction in all cases, consistent with the isolation of the one-electron reduced (PP^CF3^P)­Co­(H)­(L) (L = styrene, PMe_3_) species under reductive catalyst activation conditions.^64^ The *E*_*pc1*_ values, which correspond to a reduction from Co^II^ to Co^I^, are in the range of −1.51 V to −1.85 V vs Fc/Fc^+^, with relatively little deviation across complexes **13–20**. The trends in *E*_*pc2*_, which correspond to reduction from Co^I^ to Co^0^, follow the same pattern as the *E*_*pc1*_ values, so our discussion will focus on comparisons of *E*_*pc1*_ moving forward for the sake of brevity.

**Figure 3:**
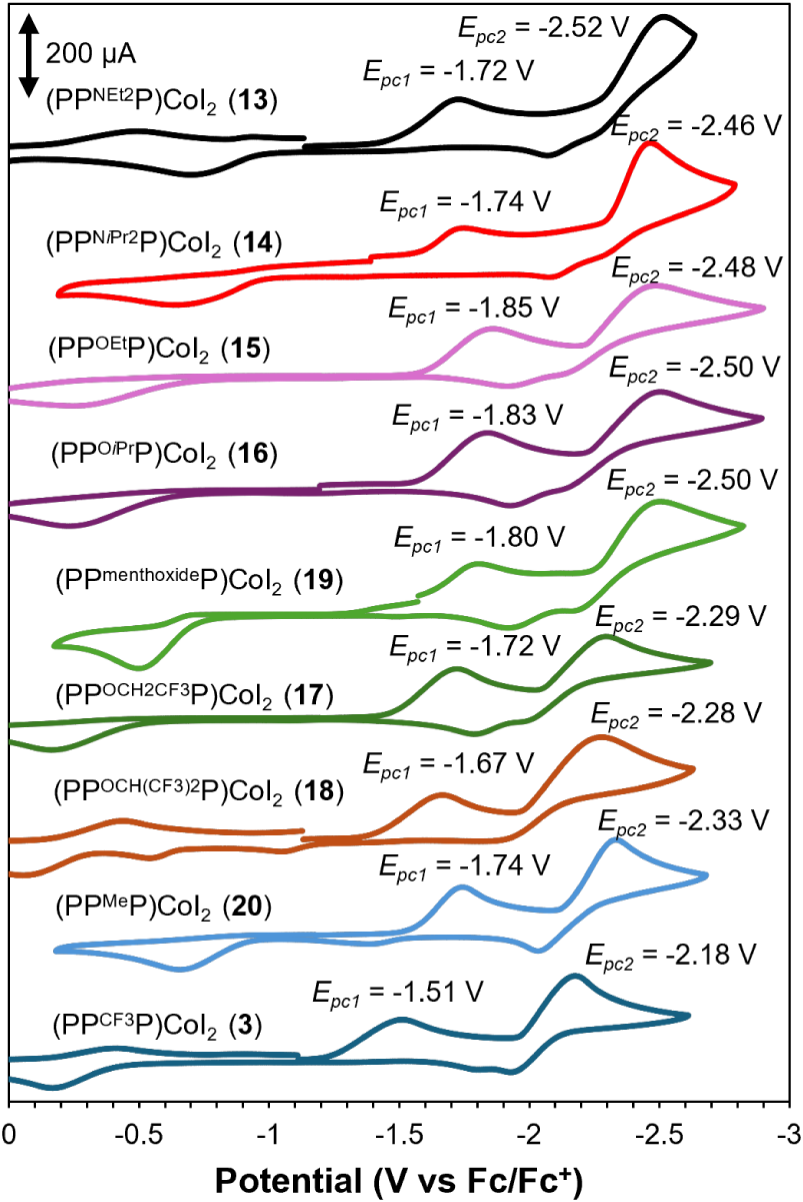
Cyclic voltammograms of complexes **3** and **13**–**20**. All spectra were collected in a THF solution of 0.1 mM [N*^n^* Bu_4_]­[PF_6_] electrolyte at a scan rate of 0.1 V/s.

The fluorinated alkoxide-substituted complexes **17** and **18** display more positive reduction potentials (−1.72 V and −1.67 V) compared to their nonfluorinated congeners **15**, **16**, and **19** (−1.80 V to −1.85 V) (Figure 3). This trend is consistent with the aforementioned shorter Co–P1 distances in **17** and **18**, which indicate stronger π-backbonding from the cobalt center to the central phosphorus ligand. The resulting diminished electron density at the cobalt center in **17** and **18** facilitates reduction at more positive *E_pc1_* values. The *E*_*pc1*_ of the amide-substituted compounds **13** and **14** are slightly more positive than the potentials for **15**, **16**, and **19**, implying that the cobalt center is less electron-rich in these derivatives compared to the alkoxyphosphine compounds. This trend is counterintuitive, as one would have expected that the diminished π-backbonding in **13**–**14** compared to **15**–**19**, as implied by Co–P distances (*vide infra*), and the enhanced basicity of amide substituents compared to alkoxide substituents to lead to a more electron-rich cobalt center in the former compounds.

Methyl-substituted complex **20** has an *E*_*pc1*_ value comparable to the amide-substituted complexes **13–14**, implying similar electron density on the cobalt center. The inductively electron-donating effects of the amide substituents would be expected to render the central phosphorus atom of **13**–**14** a stronger σ-donor than the central phosphorus moiety of the P-Me derivative **20**, leading to more negative reduction potentials for **13**–**14**. However, the amide substituents of **13**–**14** should also lead to stronger π-backbonding from cobalt to phosphorus, diminishing the electron density on cobalt. Thus, we posit that the combination of diminished σ-donation and diminished π-backbonding associated with the central phosphorus atom in **20** compared to **13**–**14** counteracts to result in similar *E*_*pc1*_ values.

The CF_3_-substituted derivative **3** exhibits the most positive Co^II/I^ reduction potential, a potential that is more than 200 mV more positive than the CH_3_-substituted analogue **20**. This signifies that the cobalt center is the least electron-rich in this compound, which can be attributed to either diminished σ-donation by the electron-poor trifluoromethyl-substituted central phosphorus and/or improved π-backbonding from the cobalt to the central phosphorus due to its substitution by a strongly electron-withdrawing group.

### Comparison of Computed ν­(CO) Stretching Frequencies for Hypothetical (PP^R^P)­Co­(CO)­(H) Compounds

In addition to redox potentials, infrared ν­(CO) stretching frequencies are a useful probe of electron density at a transition metal center. In lieu of synthesizing a series of (PP^R^P)Co carbonyl compounds, we opted to use density functional theory calculations to predict the CO stretching frequencies of a series of (PP^R^P)­Co­(CO)­(H) compounds (R = NEt_2_ (**21**), N^*i*^Pr_2_ (**22**), OEt (**23**), O^*i*^Pr (**24**), OCH_2_CF_3_ (**25**), OCH­(CF_3_)_2_ (**26**), (−)-menthoxide (**27**), Me (**28**), CF_3_ (**29**)). These hypothetical S = 0 Co^I^ compounds are direct analogues of previously reported (PP^CF3^P)­Co­(L)­(H) compounds (L = PMe_3_, styrene).^64^ The geometries were based on the structurally characterized (PP^CF3^P)­Co­(styrene)­(H) compound as a starting point, replacing the styrene ligand with CO, and modifying the phosphorus substituent as needed. The geometries of **21**–**29** were then optimized using the ωB97X-D3 functional and def2-SVP basis set. The resulting five-coordinate optimized geometries were trigonal bipyramidal (τ_5_ = 0.76–1.00)^68^ with the hydride ligand positioned trans to the central phosphorus atom in the two axial positions and the CO ligand occupying an equatorial position aligned in a syn position with respect to the central phosphorus atom substituent. Subsequent frequency calculations provided predicted ν­(CO) values for **21**–**29** (Table 3).

**Table 3:** Computed ν­(CO) IR Stretching Frequencies, Co–P Distances, and τ_5_ Geometric Parameters for (PP^R^P)­Co­(CO)­(H) Compounds **21**–**29** and (PP^NH2^P)­Co­(CO)­(H)

Compound	ν(CO) (cm^–1^)	Co–P (Å)	τ_5_
(PP^NEt2^P)Co(CO)(H) (**21**)	2089	2.095	0.90
(PP^N*i*Pr2^P)Co(CO)(H) (**22**)	2093	2.124	0.76
(PP^OEt^P)Co(CO)(H) (**23**)	2120	2.077	0.87
(PP^O*i*Pr^P)Co(CO)(H) (**24**)	2121	2.076	0.86
(PP^OCH2CF3^P)Co(CO)(H) (**25**)	2119	2.073	0.90
(PP^OCH(CF3)2^P)Co(CO)(H) (**26**)	2129	2.067	0.84
(PP^menthoxide^P)Co(CO)(H) (**27**)	2109	2.080	0.94
(PP^Me^P)Co(CO)(H) (**28**)	2101	2.082	1.00
(PP^CF3^P)Co(CO)(H) (**29**)	2123	2.062	0.96
(PP^NH2^P)Co(CO)(H)	2094	2.094	0.91

The (PP^R^P)­Co­(CO)­(H) derivatives featuring amide-substituted ligands (**21**–**22**) were calculated to have the lowest stretching frequencies among the series, consistent with these compounds possessing the most electron-rich cobalt centers to π-backbond into the CO π* orbital. The calculated ν­(CO) frequencies for the alkoxide-substituted compounds **23**–**27** are higher in energy, indicative of less π-backbonding to CO and more electron-poor cobalt centers. This trend is generally consistent with the alkoxy-substituted central phosphorus moieties participating in stronger π-backbonding than their amide-substituted analogues, in line with trends in Co–P1 distance (*vide infra*). Comparison of the alkoxy derivatives **23** and **24** with their direct fluorinated analogues **25** and **26**, respectively, reveals the ν­(CO) values to increase upon fluorination in one case (**24** vs **26**) but remain essentially constant in another (**23** vs **25**). A much more dramatic effect is observed when comparing the computed ν­(CO) for the methyl-substituted compound **28** with the trifluoromethyl compound **29**: The electron-withdrawing nature of the CF_3_ group leads to a ν­(CO) that is 22 cm^–1^ higher for **29** than for **28**, highlighting the large difference in electron density at the cobalt centers in these compounds that was also observed when comparing redox potentials (*vide infra*). The calculated ν­(CO) of the (−)-menthoxide compound **27** is anomalous, as it is predicted to be much lower than the other alkoxy-substituted derivatives (**23**–**26**). We suggest that this may be related to the slightly longer Co–P1 distance observed for **19** compared to the other (PP^OR^P)­CoI_2_ derivatives, which could limit the ability of the central phosphorus moiety to accept electron density from cobalt via π-backbonding, leading to a more electron-rich cobalt center in **27**.

### Use of ^1^ *J*_P–Se_ to Evaluate σ-Donor Properties

The redox potentials and calculated ν­(CO) stretching frequencies provide information about the impact of the central N-heterocyclic phosphorus substituent on the electron density at the cobalt center; however, variations in cobalt electron density are the net result of the combined differences in σ-donor and π-acceptor properties of the ligand. To deconvolute these two factors, we sought a quantitative method to compare the basicities of the central phosphorus donors in each ligand as a reporter of σ-donor strength. In oxidized phosphine selenide compounds, the ^1^ *J*_P–Se_ coupling constant measured by ^31^P­{^1^H} NMR spectroscopy has been used to provide a direct measurement of the σ-donor strength of a phosphorus-based ligand, with larger ^1^ *J*_P–Se_ values expected for ligands that are weaker σ-donors.^13,71,72^ We, therefore, sought to generate a series of N-heterocyclic phosphine selenide derivatives to measure and compare their relative σ-donor strength as a function of P-R substituent.

Selenation of the central phosphorus moiety of (PP^R^P) ligands **5**–**12** themselves was complicated by competing selenation of the phosphine side arms. Thus, we chose to explore the σ-donating properties of the central phosphorus as a function of substituent using a monodentate analogue of **5**–**12**. Using the *o*-fluoro-substituted aniline precursor we already had in hand for the synthesis of **5**–**12**,^66^ which provides a convenient ^19^F NMR spectroscopic handle, the chlorophosphine precursor denoted herein as (FP^Cl^F) (**30**) was synthesized via the addition of PCl_3_ to *N*,*N*-bis­(2-fluorophenyl)­ethane-1,2-diamine in the presence of excess NEt_3_ (Scheme 3). Substitution of the chloride at the central phosphorus position was accomplished under similar conditions to those used for the (PP^R^P) series **5**–**12** (Scheme 1), affording the complete series of monodentate (FP^R^F) derivatives (R = NEt_2_ (**31**), N^*i*^Pr_2_ (**32**), OEt (**33**), O^*i*^Pr (**34**), OCH_2_CF_3_ (**35**), OCH­(CF_3_)_2_ (**36**), Me (**37**), CF_3_ (**38**)). Upon isolation of the (FP^R^F) compounds **31**–**38** in spectroscopic purity, selenation of the central phosphorus atom was accomplished via stirring with Se^0^ at 50 °C in CHCl_3_ to generate phosphine selenide compounds **39–46** (Scheme 3). The reaction time required for complete selenation varied from 48 h to 3 weeks, with longer reaction times generally required for less electron-rich ligand substituents. Upon selenation, the ^31^P­{^1^H} NMR resonance of **39**–**46** shifts ∼30–50 ppm upfield compared to precursors **31**–**38** (Table 4).

**Scheme 3:**
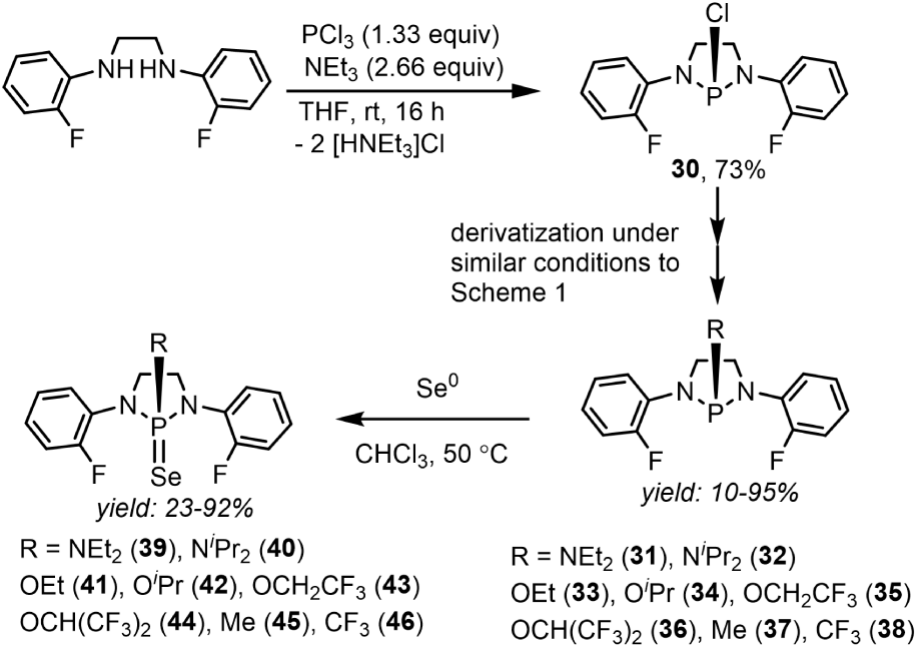
Synthesis of (FP^Cl^F) (**30**), (FP^R^F) Derivatives **31**–**38**, and Their Phosphine Selenide Derivatives **39**–**46**

**Table 4:** ^1^ *J*_P–Se_ Coupling Constants for (F­(Se=)­P^R^F) Derivatives **39**–**46** Obtained from ^31^P­{^1^H} NMR Spectra Recorded in CDCl_3_

(F(Se=)P^R^F) derivative	^31^P chemical shift (ppm)	^1^ *J*_P–Se_ (Hz)
R = NEt_2_ (**39**)	62.1	832
R = N^*i*^Pr_2_ (**40**)	49.5	825
R = OEt (**41**)	68.3	906
R = O^*i*^Pr (**42**)	67.4	897
R= OCH_2_CF_3_ (**43**)	72.4	934
R= OCH(CF_3_)_2_ (**44**)	74.9	950
R = Me (**45**)	78.0	807
R = CF_3_ (**46**)	55.4	912

The ^1^ *J*_P–Se_ coupling constants obtained from the ^77^Se satellites in the ^31^P­{^1^H} NMR spectra of **39**–**46** are listed in Table 4. Based on these values, the basicity and σ-donor strength of the N-heterocyclic phosphine ligand in (FP^R^F) decrease as the substituents are varied in the following order: Me > N^*i*^Pr_2_ > NEt_2_ > O^*i*^Pr > OEt > CF_3_ > OCH_2_CF_3_ > OCH­(CF_3_)_2_. This trend reveals that NR_2_ substituents result in a more basic phosphorus donor than OR substituents as a result of expected electronegativity trends (**39**–**40** vs **41**–**44**) and that more substituted and inductively donating alkyl substituents lead to more basic phosphorus donors (**39** vs **40** and **41** vs **42**). Replacement of methyl groups with electron-withdrawing trifluoromethyl groups universally results in weaker σ-donor ability (**41** vs **43**, **42** vs **44**, **45** vs **46**). The Me-substituted compound **45** was found to be the strongest σ-donor. In short, the observed trend in the basicity of the phosphorus atom of FP^R^F directly follows the expected basicity of R^–^.

Armed with information about σ-donor strength, we can begin to draw conclusions about the relative impact of π-acceptor ability on the nature of Co–P bonding and the relative electron density at the cobalt center in **3** and **13**–**20**. First, it can be noted that differences in Co–P distance do not track with σ-donor ability alone. For example, the Co–P distances in amide-substituted compounds **13–14** and **21**–**22** are significantly longer than those in alkoxy-substituted compounds **15**–**18** and **23**–**26** even though the central phosphorus fragments in **13**–**14** and **21**–**22** should be better σ donors. Similarly, the Co–P distances in fluoroalkoxy derivatives **17–18** and **25–26** are shorter than those in **15**–**16** and **23**–**24**, which contain more basic central phosphorus donors. The Co–P distance, therefore, is more influenced by differences in the extent of Co-to-phosphorus π-backbonding. The Me-substituted and CF_3_-substituted (PP^R^P)­CoI_2_ derivatives **3** and **20** have indistinguishable Co–P distances, which again implies that the differences in σ-donor ability of the two ligands are outweighed by stronger π-backbonding present in **3**. However, the Co–P distance is predicted to be 0.02 Å longer in the Me-substituted cobalt carbonyl/hydride complex **28** compared to the CF_3_-substituted analogue **29** (Table 3), suggesting that the degree of π-backbonding outweighs differences in σ-donor strength in these more electron-rich low-spin Co^I^ compounds. This is consistent with trends in calculated Wiberg bond indices (WBIs) that indicate a degree of Co–P multiple bonding in **21**–**29** and a higher Co–P bond order in **29** than in **28** ().

It is somewhat surprising that the ligand with the strongest σ-donor ability does not lead to the lowest predicted ν­(CO) among compounds **21**–**29** (Table 3). This must be related to a counterintuitive increase in π-backbonding to the central phosphorus in **28** compared to **21** and **22**. We initially posited that the unexpectedly diminished cobalt-to-phosphorus π-backbonding in **21** and **22** could be attributed to the lesser steric impact of the Me group in ligand **12** compared to the NR_2_ substituents in ligands **9** and **10**, allowing a closer approach of the ligand to the cobalt. Consistent with this hypothesis, the Co–P distances in **21** and **22** are predicted to be longer than the Co–P distance in **28**, a trend that was also observed when comparing the crystallographic Co–P distances of **13**/**14** and **20** (Tables 2 and 3). However, a DFT calculation of the hypothetical Co^I^ carbonyl species (PP^NH2^P)­Co­(CO)­(H), featuring an unsubstituted amide substituent that is similar in size to the Me group in **28**, revealed almost identical structural parameters to the P-NEt_2_ compound **21** and a ν­(CO) that is still higher than that of **28** (Table 3). Thus, the greater π-accepting ability of the Me-substituted diamidophosphine ligand in **12** is not a sterically derived phenomenon.

### Comparison of Catalytic Activity of **3** and **13**–**20**

With a thorough understanding of the comparative donor/acceptor properties of the pincer ligands in compounds **3** and **13**–**20** in hand, the activity of these compounds as precatalysts for alkene hydroboration was first evaluated using styrene as a model substrate. Using conditions modeled after optimized conditions reported for precatalyst **3**,^64^ each reaction was performed in duplicate in C_6_H_6_ at room temperature (23 °C) using 1.1 equiv HBpin, 1.0 mol % catalyst loading (11.3 mM in C_6_H_6_), and 2.0 mol % KBEt_3_H as an activator. As previously reported for **3**, the linear anti-Markovnikov product was observed exclusively for precatalyst derivatives **13**–**20**. Aliquots of the styrene hydroboration reactions were collected every 5 min over the course of 1 h, diluted, and analyzed via GC. Unconsumed alkene and boronate ester product peaks observed by GC were integrated and their relative composition within the reaction mixture (%) was plotted against time to generate reaction profiles (). Plots of ln­(styrene %) vs time revealed linear behavior consistent with first-order kinetics, allowing extraction of the observed reaction rate, *k*_*obs*_, for each precatalyst derivative (Table 5).

**Table 5:**
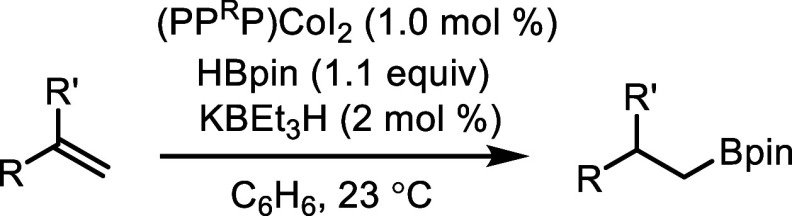
Measured Rate Constants (*k_obs_*) for the Catalytic Hydroboration of Styrene and α-Methylstyrene Using Precatalysts **3** and **13**–**20** (1.0 mol %) at 23 °C in the Presence of KBEt_3_H Activator (2.0 mol %)

**Precatalyst**	*k*_***obs***_ **(s** ^**–1**^)^a^ (**R = Ph, R′ = H)**	*k*_***obs***_ **(s** ^**–1**^)^a^ (**R = Ph, R′ = Me)**
(PP^NEt2^P)CoI_2_ (**13**)	1.2 × 10^–3^	2.6 × 10^–6^
(PP^N*i*Pr2^P)CoI_2_ (**14**)	7.8 × 10^–5^	2.4 × 10^–7^
(PP^OEt^P)CoI_2_ (**15**)	2.3 × 10^–3^	3.2 × 10^–5^
(PP^O*i*Pr^P)CoI_2_ (**16**)	1.3 × 10^–3^	1.7 × 10^–5^
(PP^OCH2CF3^P)CoI_2_ (**17**)	>6 × 10^–2^	4.2 × 10^–5^
(PP^OCH(CF3)2^P)CoI_2_ (**18**)	3.7 × 10^–3^	2.6 × 10^–6^
(PP^menthoxide^P)CoI_2_ (**19**)	1.9 × 10^–4^	4.0 × 10^–6^
(PP^Me^P)CoI_2_ (**20**)	3.0 × 10^–3^	2.4 × 10^–5^
(PP^CF3^P)CoI_2_ (**3**)	2.4 × 10^–3^	1.9 × 10^–4^

a The average of two trials.

The catalytic styrene hydroboration reactions using precatalysts **3**, **14**–**16**, and **18**–**20** could be reliably tracked over 1 h and compared among themselves (Figure 4A, Table 5). The N^*i*^Pr_2_- and (−)-menthoxide-substituted catalysts **14** and **19** were, by far the least active, likely owing to their steric hindrance impeding substrate approach at the cobalt center. With the exception of **19**, the alkoxy-substituted derivatives outperformed the amide-substituted derivatives, implying that a more electron-poor cobalt center was beneficial for catalytic turnover. This is corroborated by the increased performance of the perfluoroalkoxy-substituted precatalysts **17** and **18** compared to their direct analogues **15** and **16**. In fact, at 1.0 mol % catalyst loading, the hydroboration reaction using the trifluoroethoxy complex **17** was too rapid to obtain a *k*_*obs*_ value. The styrene was completely consumed within 5 min under these conditions, only allowing us to conclude that *k*_*obs*_ is greater than 6 × 10^–2^ s^–1^. Additional experiments at 0.1 mol % loading were conducted with both **15** and **17** to allow a more accurate comparison and provide information about the impact of fluorination of the central phosphorus substituent on catalytic activity. These results revealed the more electron-poor trifluoroethoxy compound **17** to be 3–4 times faster than the ethoxy compound **15** (Table 6, Figure 4B). The previously reported CF_3_-substituted compound **3** was found to be more active than the amide or alkoxy compounds **13**–**16**, but less active than the fluoroalkoxy compounds **17** and **18**. At first, the catalytic activity appears to track directly with the trend in ligand σ-donor strength and electron density at cobalt as indicated by ^1^ *J*_P–Se_ in **39**–**46** and ν­(CO) in **21**–**29**, respectively, with weaker σ-donating ligands and more electron-poor cobalt centers leading to enhanced catalytic activity. Counter to this trend, the Me-substituted compound **20**, which contains the most basic ligand, was found to be similar in catalytic activity to the CF_3_ compound **3**, which should have a less electron-rich cobalt center. This may be the result of steric effects, as CH_3_ is significantly smaller in size than CF_3_ (see %V_bur_ values in Table 2). Overall, the catalytic styrene hydroboration activity of the precatalysts increases in the following order as a function of the central phosphorus substituent: N^*i*^Pr_2_ ≤ (−)-menthoxide < NEt_2_ ≤ O^*i*^Pr < OEt ≤ CF_3_ ≈ CH_3_ < OCH­(CF_3_)_2_ < OCH_2_CF_3_.

**Figure 4:**
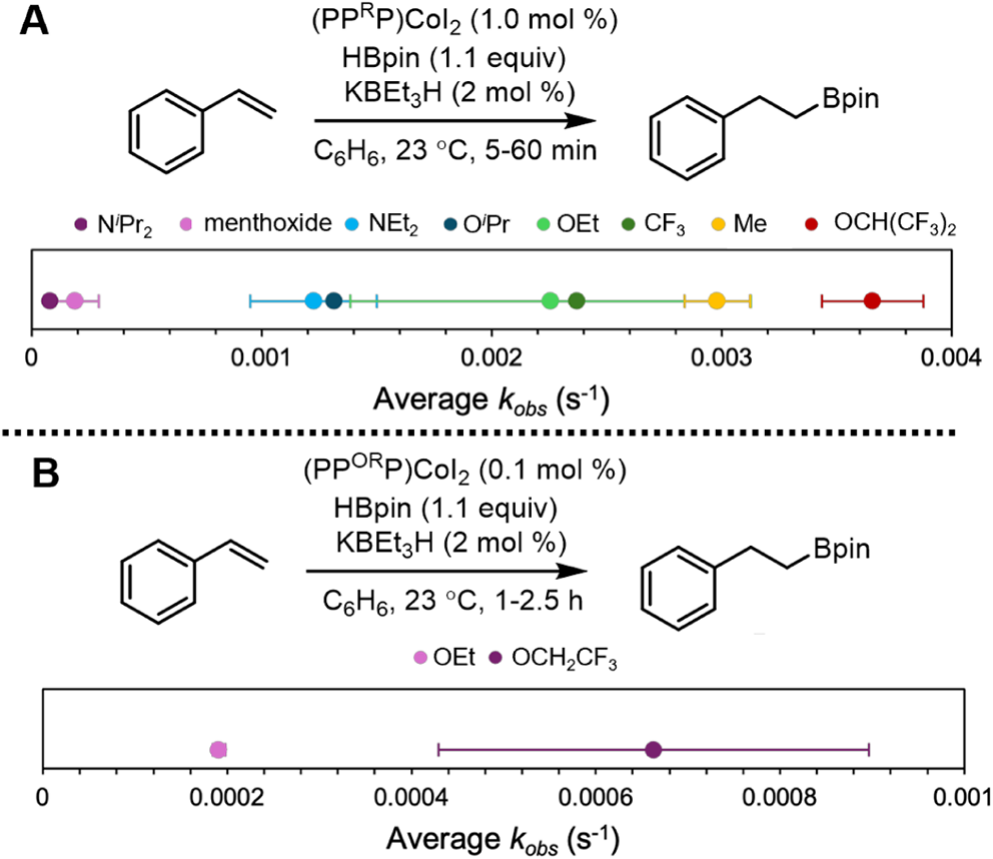
(A) Plot of *k_obs_* values (avg of two trials with standard deviations shown) for the hydroboration of styrene at 23 °C in C_6_H_6_ using 1.0 mol % loading of precatalysts **3**, **14**–**16**, and **18**–**20**. (B) Plot of *k_obs_* values (avg of two trials with standard deviations shown) for the hydroboration of styrene at 23 °C in C_6_H_6_ using 0.1 mol % loading of precatalysts **15** and **17**.

**Table 6:**
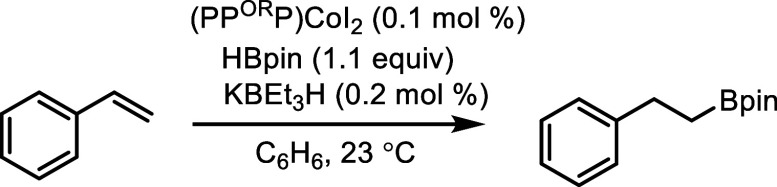
Measured Rate Constants (*k_obs_*) for the Catalytic Hydroboration of Styrene Using Precatalysts **15** and **17** (0.1 mol %) at 23 °C in the Presence of KBEt_3_H Activator (0.2 mol %)

Precatalyst	*k*_*obs*_ (s^–1^)^a^
(PP^OEt^P)CoI_2_ (**15**)	2.0 × 10^–4^
(PP^OCH2CF3^P)CoI_2_ (**17**)	6.8 × 10^–4^

a The average of two trials.

To ascertain whether the same structure/function relationships hold for a different alkene substrate, catalytic hydroboration reactions were also screened using α-methylstyrene as a more challenging alkene substrate. Reactions were performed in C_6_H_6_ at 23 °C using 1.0 mol % loading of precatalysts **3** and **13**–**20**, 2.0 mol % KBEt_3_H, and 1.1 equiv HBpin and analyzed as previously described for the styrene reactions. Under these conditions, the α-methylstyrene hydroboration reactions were universally more sluggish than observed with styrene, so time points were collected over the course of hours rather than minutes (). The *k*_*obs*_ values for each precatalyst, obtained as an average of two trials, are listed in Table 5 and plotted in Figure 5, revealing different trends in catalytic activity as a function of central phosphorus substituent compared to the styrene hydroboration reactions. With the more sterically hindered 1,1′-disubstituted alkene substrate, steric effects appear to play a much larger role in dictating catalytic activity, as the lowest *k*_*obs*_ values were observed for the precatalysts with the most bulky substituents: N^*i*^Pr_2_ (**13**), NEt_2_ (**14**), OCH­(CF_3_)_2_ (**18**), and (−)-menthoxide (**19**). While the trifluoroethoxy-substituted compound **17** remains more active than the ethoxy compound **15**, the isopropoxy compound **16** is significantly more active than its fluorinated analogue **18**. We hypothesize that this is related to the interplay between steric and electronic factors, with steric factors outweighing electronic considerations in the case of the bulkier OCH­(CF_3_)_2_ derivative. Unlike the styrene hydroboration reaction, the CF_3_ derivative **3** was found to be significantly more active for the catalytic hydroboration of α-methylstyrene than the Me derivative **20**. In this case, the presence of a more electron-poor cobalt center in **3** apparently outweighs the steric differences between CH_3_ and CF_3_. Overall, the *k*_*obs*_ value measured for the catalytic hydroboration of α-methylstyrene by precatalysts **3** and **13**–**20** increases in the following order as a function of central phosphorus substituent: N^*i*^Pr_2_ < NEt_2_ = OCH­(CF_3_)_2_ = (−)-menthoxide < O^*i*^Pr < Me < OEt < OCH_2_CF_3_ < CF_3_.

**Figure 5:**
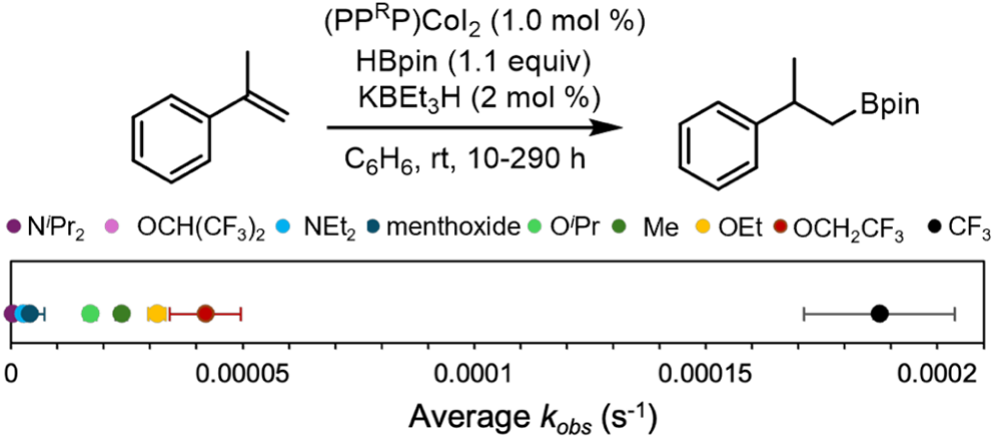
Plot of *k_obs_* values (avg of two trials with standard deviations shown) for the hydroboration of α-methylstyrene at 23 °C in C_6_H_6_ using 1.0 mol % loading of precatalysts **3** and **13**–**20**.

## Conclusions

In summary, a series of eight (PP^R^P)­CoI_2_ new derivatives of the previously reported (PP^CF3^P)­CoI_2_ hydroboration precatalyst have been synthesized and characterized (R = NEt_2_, N^*i*^Pr_2_, OEt, O^*i*^Pr, OCH_2_CF_3_, OCH­(CF_3_)_2_, (−)-menthoxide, Me). The differences in electron density at the cobalt center imparted by the varying σ-donor and π-acceptor properties of the central diamidophosphine, diamido phosphite, or triamido phosphine moiety were assessed by comparing the reduction potentials of the (PP^R^P)­CoI_2_ compounds measured using cyclic voltammetry as well as the computed ν­(CO) stretching frequencies of hypothetical (PP^R^P)­Co­(CO)­(H) compounds. In general, it was found that the amide substituents led to more electron density at the cobalt center than the alkoxy substituents, and that fluorination of the phosphorus substituent resulted in a more electron-poor cobalt center. Trends in Co–P bond distance indicated that shorter bond distances did not correlate with electron density at cobalt, indicating that cobalt-to-phosphorus π-backbonding contributes significantly to the Co–P bonding. By comparing the ^1^ *J*_P–Se_ coupling constants of a series of oxidized phosphine selenide derivatives, the relative basicity, which is directly related to σ-donor strength, of the central phosphorus atoms as a function of the R substituent was determined, revealing a trend that generally tracks well with the trend in electron density at the cobalt center, but does not correlate with Co–P bond distance. For example, the shortest Co–P distances are found in the derivatives containing the least basic phosphorus centers with OCH_2_CF_3_ and OCH­(CF_3_)_2_ substituents. The P-Me derivative was found to be the best σ-donor in the series but the resulting (PP^Me^P)Co compounds were found to be less electron-rich than the (PP^NR2^P)Co compounds, suggesting that the Me-substituted ligand derivative may be π-backbonding more strongly than the amide derivatives, perhaps as the result of steric constraints.

All nine (PP^R^P)­CoI_2_ compounds were found to be active precatalysts for the hydroboration of styrene and α-methylstyrene resulting in selective conversion to the anti-Markovnikov product. Comparing the rate constants for catalysis under identical conditions (*k*_*obs*_), it was generally found that more electron-poor cobalt compounds with more weakly donating and/or stronger π-accepting ligands led to faster catalytic turnover. Although the catalytic reaction mechanism has not been firmly elucidated and is currently under investigation, the observation that more electron-poor catalysts perform better is an indication that the rate-determining step does not involve oxidative addition and that σ-bond metathesis is more likely the rate-determining product formation step.^73^ Of note, the fluorinated alkoxy derivatives (PP^OCH2CF3^P)­CoI_2_ and (PP^OCH(CF3)2^P)­CoI_2_ significantly outperform the previously reported (PP^CF3^P)­CoI_2_ compound as precatalysts for the catalytic hydroboration of styrene. With the bulkier 1,1′-disubstituted alkene, catalytic activity shows a stronger dependence on steric effects, with the P-CF_3_ derivative significantly outperforming the other derivatives.

This study lends insight into the design of first-row transition metal pincer catalysts for alkene hydrofunctionalization, demonstrating that increasing the donor ability of the ligand is not always the optimal strategy for enhancing catalyst performance. Moreover, this work demonstrates the utility of diamidophosphine-containing fragments as ligands with tunable π-acceptor properties.

## Experimental Section

### General Considerations

Unless otherwise noted, all manipulations were carried out under an inert atmosphere using a nitrogen-filled glovebox or standard Schlenk techniques. Glassware was oven-dried before use. All proteo solvents were degassed by sparging with ultrahigh-purity argon and dried via passage through columns of drying agents using a Glass Contours solvent purification system from Pure Process Technologies. Benzene-*d*_6_ was degassed via repeated freeze–pump–thaw cycles and dried over 3 Å molecular sieves before use. CD_2_Cl_2_ was dried over CaH_2_, vacuum transferred, and degassed via repeated freeze–pump–thaw cycles. KBEt_3_H was purchased from Sigma-Aldrich as a 1 M solution in THF and used without further purification. Triethylamine, diethylamine, and diisopropylamine were distilled, degassed via repeated freeze–pump–thaw cycles, and dried over 3 Å molecular sieves before use. Ethanol, isopropanol, and styrene were degassed via repeated freeze–pump–thaw cycles and dried over 3 Å molecular sieves before use. (PP^Cl^P) (**4**), *N*,*N*-bis­(2-fluorophenyl)­ethane-1,2-diamine, and (PP^CF3^P)­CoI_2_ were synthesized according to literature procedures.^64,66^ NMR spectra were recorded at ambient temperature on a Bruker NEO 400 MHz instrument. ^1^H NMR and ^13^C­{^1^H} NMR chemical shifts were referenced to residual solvent resonances and are reported in ppm. ^31^P­{^1^H} and ^19^F­{^1^H} NMR chemical shifts (in ppm) were referenced to 85% H_3_PO_4_ (0 ppm) and neat trifluoroacetic acid (−76.55 ppm) external standards, respectively. All other reagents and solvents were obtained from commercial sources and used without further purification. GC-MS analysis was performed using an Agilent 7890B GC system equipped with the HP-5 Ultra Inert column (30 m, 0.25 mm, 0.25 μm) and a FID detector. For MS detection, an electron ionization system was used with an ionization energy of 70 eV.

### Synthesis of (PP^NEt2^P) (**5**)

*Method 1*: (PP^Cl^P) (**4**) (0.5115 g, 0.7929 mmol, 1 equiv) was dissolved in THF (10 mL) in a nitrogen-filled glovebox. Triethylamine (243.2 μL, 1.74 mmol, 2.2 equiv) was added and this solution was allowed to stir for 2 min. Diethylamine (82.0 μL, 0.793 mmol, 1.0 equiv) was added, causing the solution to become cloudy due to the precipitation of [HNEt_3_]­[Cl]. This solution was allowed to stir at 23 °C for 16 h, resulting in further precipitation of [HNEt_3_]­[Cl]. The resulting suspension was filtered through a Celite-packed pipet and the solvent was removed from the filtrate under vacuum. The resulting white solid was triturated with pentane (3 × 3 mL) to remove residual THF and triethylamine, yielding (PP^NEt2^P) (**5**) as a shiny white solid (0.341 g, 63.2% yield). *Method 2*: (PP^Cl^P) (**4**) (0.2380 g, 0.3690 mmol, 1 equiv) was dissolved in THF (4 mL) in a nitrogen-filled glovebox. Diethylamine (114.0 μL, 1.11 mmol, 3.0 equiv) was added, causing the solution to become cloudy due to the precipitation of [H_2_NEt_2_]­[Cl]. This solution was allowed to stir at 23 °C for 16 h, resulting in further precipitation of [H_2_NEt_2_]­[Cl]. The resulting suspension was filtered through a Celite-packed pipet, and the solvent was removed from the filtrate under vacuum. This resulted in the isolation of (PP^NEt2^P) (**5**) as a white powder (0.158 g, 63%). ^1^H NMR (400 MHz, benzene-*d*_6_): δ 7.57–7.48 (m, 4H), 7.40–7.53 (m, 6H), 7.19–7.17 (m, 2H), 7.14–6.95 (m, 14H), 6.87–6.78 (m, 2H), 3.95–3.84 (m, 2H), 3.16–3.06 (m, 2H), 2.98 (dq, ^3^ *J*_H–H_ = 7.1 Hz, ^3^ *J*_H–P_ = 9.4 Hz, 4H), 0.75 (t, ^3^ *J*_H–H_ = 7.1 Hz, 6H). ^31^P­{^1^H} NMR (162 MHz, C_6_D_6_): δ 104.62 (t, *J*_P–P_ = 64.1 Hz, 1P), −15.42 (d, *J*_P–P_ = 64.1 Hz, 2P). ^13^C­{^1^H} NMR (151 MHz, C_6_D_6_): 152.09 (m), 139.97 (m), 139.01 (m), 135.89 (s), 135.13 (m), 134.87 (m), 133.85 (m), 130.23 (s), 128.90–127.64 (overlapping signals with C_6_D_6_), 126.66 (m), 124.53 (m), 54.21 (m), 40.04 (dt, ^2^ *J*_C–P_ = 20.2 Hz, *J*_C–P_ = 2.1 Hz), 15.10 (s).

### Synthesis of (PP^N*i*Pr2^P) (**6**)

(PP^Cl^P) (**4**) (0.4340 g, 0.6728 mmol, 1 equiv) was dissolved in THF (10 mL) in a nitrogen-filled glovebox. To this solution was added diisopropylamine (282.9 μL, 2.02 mmol, 3.0 equiv), resulting in the gradual formation of a precipitate ([H_2_N^*i*^Pr_2_]­[Cl]). This suspension was allowed to stir for 16 h before filtration through a Celite-packed pipet to remove the precipitated salt and removal of the solvent from the filtrate in vacuo. The resulting white solid was triturated with pentane (3 × 3 mL) to remove residual THF and diisopropylamine. This resulted in the isolation of (PP^N*i*Pr2^P) (**6**) as a white powder (0.327 g, 68.4% yield). ^1^H NMR (400 MHz, benzene-*d*_6_): δ 7.63–7.52 (m, 6H), 7.42–7.36 (m, 4H), 7.24–7.17 (m, 2H), 7.13–6.95 (m, 14H), 6.83–6.78 (m, 2H), 4.14–3.92 (m, 2H), 3.68 (dsept, ^3^ *J*_H–H_ = 6.6 Hz, ^3^ *J*_H–P_ = 9.8 Hz, 2H), 2.97–2.80 (m, 2H), 1.11 (d, *J* = 6.7 Hz, 12H). ^31^P­{^1^H} NMR (162 MHz, benzene-*d*_6_): δ 93.82 (t, *J*_P–P_ = 64.8 Hz, 1P), −15.06 (d, *J*_P–P_ = 64.4 Hz, 2P). ^13^C­{^1^H} NMR (151 MHz, benzene-*d*_6_): δ 152.51 (m), 140.18 (m), 139.11 (m), 136.31 (s), 134.89–133.51 (overlapping signals), 129.94 (s), 129.00–127.67 (overlapping signals with C_6_D_6_), 126.81 (m), 124.07 (m), 54.38 (m), 45.18 (d, ^2^ *J*_C–P_ = 11.7 Hz), 25.10 (d, *J*_C–P_ = 6.8 Hz).

### Synthesis of (PP^OEt^P) (**7**)

(PP^Cl^P) (**4**) (0.3551 g, 0.5504 mmol, 1.0 equiv) was dissolved in THF (3 mL) in a nitrogen-filled glovebox. To this solution was added triethylamine (168.8 μL, 1.21 mmol, 2.2 equiv) and the mixture was allowed to stir for 2 min. Ethanol (32.1 μL, 0.550 mmol, 1.0 equiv) was added, causing immediate precipitation of a white solid ([HNEt_3_]­[Cl]). This suspension was allowed to stir for 16 h before filtration through a Celite-packed pipet to remove the precipitated salt and removal of solvent from the filtrate in vacuo. The resulting white solid was triturated with pentane (3 × 3 mL) to remove residual THF and triethylamine. This resulted in the isolation of (PP^OEt^P) (**7**) as a shiny white solid (0.279 g, 77.5% yield). ^1^H NMR (400 MHz, C_6_D_6_): δ 7.46–7.32 (m, 10H), 7.14–6.95 (m, 16H), 6.90–6.81 (m, 2H), 3.90–3.81 (m, 2H), 3.66 (quint, ^3^ *J*_H–H_ = 7.1 Hz, 2H), 3.32–3.22 (m, 2H), 0.91 (t, ^3^ *J*_H–H_ = 7.0 Hz, 3H). ^31^P­{^1^H} NMR (162 MHz, C_6_D_6_): δ 118.74 (t, *J*_P–P_ = 42.5 Hz, 1P), −15.21 (d, *J*_P–P_ = 42.7 Hz, 2P). ^13^C­{^1^H} NMR (151 MHz, C_6_D_6_): δ 150.54 (m), 138.94 (m), 138.72 (m), 136.59 (m), 135.21 (s), 134.61–134.01 (overlapping signals), 131.19 (s), 130.38 (s), 128.79–127.59 (signals overlapping with C_6_D_6_), 125.71 (m), 60.28 (dt, ^2^ *J*_C–P_ = 11.6 Hz, *J*_C–P_ = 2.9 Hz), 54.59 (m), 17.38 (d, ^3^ *J*_C–P_ = 4.7 Hz).

### Synthesis of (PP^O*i*Pr^P) (**8**)

(PP^Cl^P) (**4**) (0.3145 g, 0.4876 mmol, 1 equiv) was dissolved in THF (3 mL) in a nitrogen-filled glovebox. To this solution was added triethylamine (149.5 μL, 1.07 mmol, 2.2 equiv) and the mixture was allowed to stir for 2 min. Isopropanol (37.3 μL, 0.488 mmol, 1.0 equiv) was added, causing immediate precipitation of a white solid ([HNEt_3_]­[Cl]). This suspension was allowed to stir for 16 h before filtration through a Celite-packed pipet to remove the precipitated salt and removal of solvent from the filtrate under vacuo. The resulting white solid was triturated with pentane (3 × 3 mL) to remove residual solvents. This resulted in the isolation of (PP^O*i*Pr^P) (**8**) as a shiny white solid (0.261 g, 80.1% yield). ^1^H NMR (400 MHz, C_6_D_6_): δ 7.50–7.34 (m, 10H), 7.16–6.94 (m, 16H), 6.90–6.82 (m, 2H), 3.95 (dsept, ^3^ *J*_H–H_ = 6.2 Hz, ^3^ *J*_H–P_ = 9.0 Hz, 1H), 3.86–3.78 (m, 2H), 3.16–3.05 (m, 2H), 0.94 (d, *J* = 6.2 Hz, 6H). ^31^P­{^1^H} NMR (162 MHz, C_6_D_6_): δ 122.38 (t, *J*_P–P_ = 53.5 Hz, 1P), −15.93 (d, *J*_P–P_ = 53.4 Hz, 2P). ^13^C­{^1^H} NMR (151 MHz, C_6_D_6_): δ 150.52 (m), 139.42 (m), 138.78 (m), 136.08 (m), 135.35 (s), 134.74–134.05 (overlapping signals), 130.21 (s), 128.87–127.64 (overlapping signals with C_6_D_6_), 127.10 (m), 125.34 (m), 67.36 (d, ^2^ *J*_C–P_ = 20.6 Hz), 53.30 (m), 25.01 (d, ^3^ *J*_C–P_ = 3.8 Hz).

### Synthesis of (PP^OCH2CF3^P) (**9**)

(PP^Cl^P) (**4**) (0.2931 g, 0.4544 mmol, 1 equiv) was dissolved in THF (2 mL) in a nitrogen-filled glovebox. To this solution was added triethylamine (139.3 μL, 1.00 mmol, 2.2 equiv) and the mixture was allowed to stir for 2 min. 2,2,2-Trifluoroethanol (32.7 μL, 0.454 mmol, 1.0 equiv) was added, causing the immediate precipitation of a white solid ([HNEt_3_]­[Cl]). This suspension was allowed to stir for 16 h before filtration through a Celite-packed pipet to remove the precipitated salt. The solvent was removed from the filtrate in vacuo. The resulting white solid was triturated with pentane (3 × 3 mL) to remove residual THF and triethylamine. This resulted in the isolation of (PP^OCH2CF3^P) (**9**) as a shiny white solid (0.248 g, 77.1% yield). ^1^H NMR (400 MHz, C_6_D_6_): δ 7.43–7.27 (m, 10H), 7.14–6.98 (m, 16H), 6.89–6.81 (m, 2H), 3.84–3.57 (m, 4H), 3.22–3.10 (m, 2H). ^31^P­{^1^H} NMR (162 MHz, C_6_D_6_): δ 125.68 (tq, *J*_P–P_ = 40.1 Hz, *J*_P–F_ = 3.5 Hz, 1P), −15.91 (d, *J*_P–P_ = 40.3 Hz, 2P). ^19^F­{^1^H} NMR (377 MHz, C_6_D_6_): δ −75.84 (d, *J*_F–P_ = 3.5 Hz). ^13^C­{^1^H} NMR (151 MHz, C_6_D_6_): δ 149.15 (m), 138.29 (m), 136.67 (m), 135.07 (s), 134.38 (m), 130.53 (s), 128.83 (s), 128.46–127.68 (signals obscured by C_6_D_6_), 61.98 (m), 54.34 (m).

### Synthesis of (PP^OCH(CF3)2^P) (**10**)

(PP^Cl^P) (**4**) (0.4283 g, 0.6640 mmol, 1 equiv) was dissolved in THF (10 mL) in a nitrogen-filled glovebox. To this solution was added triethylamine (203.6 μL, 1.46 mmol, 2.2 equiv) and the solution was allowed to stir for 2 min. Hexafluoroisopropanol (69.9 μL, 0.664 mmol, 1.0 equiv) was added, causing the immediate precipitation of a white solid ([HNEt_3_]­[Cl]). This suspension was allowed to stir for 16 h before filtration through a Celite-packed pipet to remove the precipitated salt and removal of solvent from the filtrate in vacuo. The resulting white solid was triturated with pentane (3 × 3 mL) to remove residual THF and triethylamine. This resulted in the isolation of (PP^OCH(CF3)2^P) (**10**) as a shiny white solid (0.497 g, 96.5% yield). ^1^H NMR (400 MHz, C_6_D_6_): δ 7.47–7.34 (m, 8H), 7.31–7.22 (m, 2H), 7.15–6.99 (m, 16H), 6.88–6.81 (m, 2H), 4.23 (dsept, ^3^ *J*_H–F_ = 6.8 Hz, ^3^ *J*_H–P_ = 2.0 Hz, 1H), 3.76–3.51 (m, 2H), 3.11–2.89 (m, 2H). ^31^P­{^1^H} NMR (162 MHz, C_6_D_6_): δ 129.79 (tsept, *J*_P–P_ = 72.9 Hz, *J*_P–F_ = 7.5 Hz, 1P), −18.26 (d, ^3^ *J*_P–P_ = 72.6 Hz, 2P). ^19^F NMR (377 MHz, C_6_D_6_): δ −75.84 (d, *J*_F–P_ = 7.5 Hz). ^13^C­{^1^H} NMR (151 MHz, C_6_D_6_): δ 147.99 (m), 139.11 (m), 137.97 (m), 135.82–135.59 (overlapping signals), 135.56 (s), 134.39 (m), 130.44 (s), 128.91–127.73 (peaks obscured by C_6_D_6_ peaks), 127.33 (m), 126.13 (s), 122.27 (q, ^1^ *J*_C–F_ = 288.0 Hz), 70.71 (m), 53.14 (m).

### Synthesis of (PP^menthoxide^P) (**11**)

(PP^Cl^P) (**4**) (0.3560 g, 0.5510 mmol, 1.0 equiv) was dissolved in THF (2 mL) in a nitrogen-filled glovebox. To this solution was added triethylamine (168.8 μL, 1.21 mmol, 2.2 equiv) and was allowed to stir for 2 min. (−)-Menthol (0.0861 g, 0.551 mmol, 1.0 equiv) was dissolved in THF (2 mL) and added to the reaction solution, causing immediate precipitation of a white solid ([HNEt_3_]­[Cl]). This suspension was allowed to stir for 16 h before filtration through a Celite-packed pipet to remove the precipitated salt and removal of the solvent from the filtrate in vacuo. The resulting white solid was triturated with pentane (3 × 2 mL) to remove residual THF and triethylamine. This resulted in the isolation of (PP^menthoxide^P) (**11**) as a shiny white solid (0.315 g, 74.7% yield). ^1^H NMR (400 MHz, C_6_D_6_): δ 7.60–7.50 (m, 4H), 7.49–7.33 (m, 6H), 7.26–7.17 (m, 2H), 7.15–6.98 (m, 14H), 6.89–6.78 (m, 2H), 3.93–3.75 (m, 2H), 3.45–3.32 (m, 1H), 3.11–3.92 (m, 2H), 2.09–1.98 (m, 1H), 1.58–1.50 (m, 1H), 1.47–1.41 (m, 2H), 1.24–1.15 (m, 1H), 1.01–0.57 (overlapping peaks, 11H), 0.53–0.46 (d, ^3^ *J*_H–H_ = 6.9 Hz, 3H). ^31^P­{^1^H} NMR (162 MHz, C_6_D_6_): δ 122.73 (dd, *J*_P–P_ = 72.6 Hz, *J*_P–P_ = 72.4 Hz, 1P), −16.22 (dd, *J*_P–P_ = 73.6 Hz, *J*_P–P_ = 73.6 Hz, 1P), −17.38 (dd, *J*_P–P_ = 71.6 Hz, *J*_P–P_ = 71.6 Hz, 1P). ^13^C­{^1^H} NMR (151 MHz, benzene-*d*_6_): δ 151.78 (m), 150.73 (m), 140.43 (m), 139.92 (m), 138.98 (m), 136.61 (m), 135.94–134.21 (overlapping signals), 131.19 (s), 130.21 (s), 129.94 (s), 128.75–127.45 (signals overlapping with C_6_D_6_), 126.89 (m), 126.26 (m), 125.02 (s), 124.73 (s), 74.60 (d), 52.94 (m), 52.49 (m), 49.62 (m), 44.95 (m), 43.29 (s), 34.72 (s), 31.72 (s), 25.29 (s), 23.19 (s), 22.40 (s), 21.52 (s), 15.83 (s).

### Synthesis of (PP^Me^P) (**12**)

(PP^Cl^P) (**4**) (0.2743 g, 0.4252 mmol, 1 equiv) was dissolved in THF (4 mL) in a nitrogen-filled glovebox. This solution was allowed to stir while a solution of MeMgBr (303.7 μL, 1.4 M in THF, 0.43 mmol, 1.0 equiv) in THF (2 mL) was added dropwise. This solution was allowed to stir for 16 h before the addition of dioxane (4 mL). This resulted in the immediate precipitation of a fine white powder (MgBrCl). The cloudy solution was filtered through a Celite-packed pipet and residual THF and dioxane were removed from the filtrate under vacuum at 50 °C. The resulting oil was recrystallized in minimal Et_2_O (5 mL) at −35 °C, resulting in the isolation of (PP^Me^P) **(14**) as a white powder (0.173 g, 65.2% yield). ^1^H NMR (400 MHz, C_6_D_6_): δ 7.46–7.29 (m, 8H), 7.12–7.00 (m, 16H), 6.94–6.89 (m, 2H), 6.78–6.69 (m, 2H), 3.57–3.46 (m, 2H), 3.34–3.24 (m, 2H), 1.30 (dt, ^2^ *J*_H–P_ = 6.6 Hz, *J*_H–P_ = 1.9 Hz, 3H). ^31^P­{^1^H} NMR (162 MHz, C_6_D_6_): δ 108.41 (t, *J*_P–P_ = 86.7 Hz, 1P), −14.27 (d, *J*_P–P_ = 86.8 Hz, 2P). ^13^C­{^1^H} NMR (151 MHz, C_6_D_6_): δ 154.07 (m), 139.05–138.54 (overlapping signals), 135.55 (s), 132.36 (m), 130.37 (s), 128.97–127.70 (overlapping signals), 123.25 (s), 121.88 (m), 52.34 (m), 17.49 (dt, ^2^ *J*_C–P_ = 24.1 Hz, *J*_C–P_ = 3.4 Hz).

### Synthesis of (PP^NEt2^P)­CoI_2_ (**13**)

(PP^NEt2^P) (**5**) (0.1922 g, 0.2819 mmol, 1 equiv) was dissolved in THF (4 mL) in a nitrogen-filled glovebox. This solution was added to a stirring solution of CoI_2_ (0.1058 g, 0.3383 mmol, 1.2 equiv) in THF (3 mL). Upon addition, the bright blue solution immediately turned dark brown. This solution was allowed to stir for 24.5 h, resulting in a suspension of an orange powder in a blue-green solution. The reaction solution was filtered through glass microfiber filter paper using a pipet filter and the solid was collected via suspension in pentane (7 mL). Minimal dichloromethane (1 mL) was pushed through the pipet filter to collect any residual solid. Combination of the dichloromethane solution and pentane suspension resulted in the immediate formation of a brown, crystalline material. Removal of solvent under vacuum yielded (PP^NEt2^P)­CoI_2_ (**13**) as a deep brown crystalline solid (0.179 g, 63.9% yield). Single crystals suitable for X-ray analysis were grown via vapor diffusion of Et_2_O into a saturated solution of **13** in CH_2_Cl_2_. ^1^H NMR (400 MHz, CD_2_Cl_2_): δ 9.53 (br s), 8.57 (br s), 8.37 (br s), 7.56 (br s), 7.33 (br s), 5.91 (br s), 5.51 (br s), 4.96 (br s). Evans’ Method (CD_2_Cl_2_): μ_eff_ = 1.84 ± 0.06 μ_b_. Elemental analysis for C_42_H_42_CoI_2_N_3_P_3_•2CH_2_Cl_2_ (Calcd (Found)): %C, 45.39 (45.72); %H, 3.98 (4.57); %N, 3.61 (3.95).

### Synthesis of (PP^NiPr2^P)­CoI_2_ (**14**)

(PP^NiPr2^P) (**6**) (0.0684 g, 0.0964 mmol, 1 equiv) was dissolved in THF (2 mL) in a nitrogen-filled glovebox. This solution was added to a stirring solution of CoI_2_ (0.0390 g, 0.125 mmol, 1.3 equiv) in THF (2 mL). Upon addition, the bright blue solution immediately turned dark brown. This solution was allowed to stir for 23 h, resulting in a suspension of a brownish powder in a dark orange-brown solution. The reaction solution was allowed to cool at −35 °C for 1 h to encourage further precipitation. The solid was collected via filtration through glass microfiber filter paper using a pipet filter and subsequent suspension in pentane (5 mL). Removal of solvent from the pentane suspension under vacuum resulted in the isolation of (PP^NiPr2^P)­CoI_2_ (**14**) as a golden-brown powder (0.067 g, 68% yield). Single crystals suitable for X-ray analysis were grown via vapor diffusion of Et_2_O into a saturated solution of **14** in CH_2_Cl_2_. ^1^H NMR (400 MHz, CD_2_Cl_2_): δ 9.62 (br s), 9.37 (br s), 8.29 (br s), 7.73 (br s), 7.20 (br s), 6.27 (br s), 5.78 (br s), 5.57 (br s), 4.38 (br s). Evans’ Method (CD_2_Cl_2_): μ_*eff*_ = 1.79 ± 0.2 μ_B_. Elemental analysis for C_44_H_46_CoI_2_N_3_P_3_•CH_2_Cl_2_ (Calcd (Found)): %C, 48.80 (49.73); %H, 4.37 (4.86); %N, 3.79 (3.88).

### Synthesis of (PP^OEt^P)­CoI_2_ (**15**)

(PP^OEt^P) (**7**) (0.0871 g, 0.133 mmol, 1 equiv) was dissolved in THF (4 mL) in a nitrogen-filled glovebox. This solution was added to a stirring solution of CoI_2_ (0.0516 g, 0.165 mmol, 1.24 equiv) in THF (3 mL). Upon addition, the bright blue solution immediately turned dark red-brown. This solution was allowed to stir for 24.5 h, resulting in a suspension of a bright red powder in a dark orange-brown solution. The reaction solution was filtered through glass microfiber filter paper using a pipet filter and the solid was collected via suspension in pentane (7 mL). Residual solid in the pipet filter was collected with minimal dichloromethane (1 mL). The pentane suspension and dichloromethane solution were combined and solvents were removed under vacuum to yield (PP^OEt^P)­CoI_2_ (**15**) as a bright red-orange powder (0.095 g, 74% yield). Single crystals suitable for X-ray analysis were grown by slow evaporation of a saturated CH_2_Cl_2_ solution at −35 °C. ^1^H NMR (400 MHz, CD_2_Cl_2_): δ 9.34 (br s), 8.57 (br s), 8.24 (br s), 7.64 (br s), 5.63 (br s), 5.48 (br s), 3.31 (br s), 2.43 (br s), 1.05 (br s). Evans’ Method (CD_2_Cl_2_): μ_*eff*_ = 1.91 ± 0.05 μ_B_. Elemental analysis for C_40_H_37_CoI_2_N_2_OP_3_•CH_2_Cl_2_ (Calcd (Found)): %C, 46.80 (47.73); %H, 3.74 (4.49); %N, 2.66 (2.38).

### Synthesis of (PP^O*i*Pr^P)­CoI_2_ (**16**)

(PP^O*i*Pr^P) (**8**) (0.1137 g, 0.1700 mmol, 1 equiv) was dissolved in THF (4 mL) in a nitrogen-filled glovebox. This solution was added to a stirring solution of CoI_2_ (0.0636 g, 0.203 mmol, 1.2 equiv) in THF (3 mL). Upon addition, the bright blue solution immediately turned dark orange-brown. This solution was allowed to stir for 24.5 h, resulting in a suspension of a dark red powder in a dark orange-brown solution. The reaction solution was filtered through glass microfiber filter paper using a pipet filter and the solid was collected via suspension in pentane. Residual solid in the filter pipet was collected with minimal CH_2_Cl_2_ (1 mL). The pentane suspension and dichloromethane solution were combined and solvents were removed under vacuum to yield (PP^O*i*Pr^P)­CoI_2_ (**16**) as a bright orange powder (0.131 g, 78.3% yield). Single crystals suitable for X-ray analysis were grown by slow evaporation of a concentrated CH_2_Cl_2_ solution. ^1^H NMR (400 MHz, CD_2_Cl_2_): δ 9.45 (br s), 8.29 (br s), 8.19 (br s), 7.86 (br s), 5.58 (br s), 3.28 (br s), 1.62 (br s). Evans’ Method (CD_2_Cl_2_): μ_*eff*_ = 1.83 ± 0.1 μ_B_. Elemental analysis for C_41_H_39_CoI_2_N_2_OP_3_•1.5 CH_2_Cl_2_ (Calcd (Found)): %C, 46.04 (46.06); %H, 3.82 (4.28); %N, 2.53 (2.38).

### Synthesis of (PP^OCH2CF3^P)­CoI_2_ (**17**)

(PP^OCH2CF3^P) (**9**) (0.1342 g, 189.4 μmol, 1 equiv) was dissolved in THF (5 mL) in a nitrogen-filled glovebox. This solution was added to a stirring solution of CoI_2_ (0.0709 g, 227 μmol, 1.2 equiv) in THF (5 mL). Upon addition, the bright blue solution immediately turned dark brown. This solution was allowed to stir for 25 h, resulting in a suspension of an orange powder in a dark brown solution. The reaction solution was filtered through glass microfiber filter paper using a pipet filter and the solid was collected via suspension in pentane (10 mL). Removal of pentane under vacuum resulted in the isolation of (PP^OCH2CF3^P)­CoI_2_ (**17**) as a red-orange powder (0.149 g, 77.1% yield). Single crystals suitable for X-ray analysis were grown by slow evaporation of a saturated CH_2_Cl_2_ solution at −35 °C. ^1^H NMR (400 MHz, CD_2_Cl_2_): δ 9.36 (br s), 8.41 (br s), 8.29 (br s), 8.19 (br s), 7.61 (br s), 5.67 (br s), 5.42 (br s), 3.22 (br s), 0.50 (br s). Evans’ Method (CD_2_Cl_2_): μ_*eff*_ = 1.88 ± 0.1 μ_B_. Elemental analysis for C_40_H_34_CoF_3_I_2_N_2_OP_3_•2CH_2_Cl_2_ (Calcd (Found)): %C, 42.35 (42.40); %H, 3.22 (3.94); %N, 2.35 (2.07).

### Synthesis of (PP^OCH(CF3)2^P)­CoI_2_ (**18**)

(PP^OCH(CF3)2^P) (**10**) (0.2276 g, 0.2931 mmol, 1 equiv) was dissolved in THF (4 mL) in a nitrogen-filled glovebox. This solution was added to a stirring solution of CoI_2_ (0.1097 g, 0.3508 mmol, 1.2 equiv) in THF (3 mL). Upon addition, the bright blue solution immediately turned dark red-brown. This solution was allowed to stir for 25 h, then equal amounts of pentane (7 mL) were layered on top of the THF reaction mixture. Crystalline material formed after 72 h at −35 °C. The blue-green mother liquor was decanted, leaving dark brown crystals, orange powder, and blue powder. This mixture was divided into two portions and relayered with THF/pentane (5 mL/5 mL) and again stored at −35 °C for 72 h. Decanting of the blue-green mother liquor and removal of residual solvent under vacuum resulted in a black crystalline material, (PP^OCH(CF3)2^P)­CoI_2_ (**18**) (0.322 g, >99% yield). Single crystals suitable for X-ray analysis were grown by slow evaporation of a saturated CH_2_Cl_2_ solution at −35 °C. ^1^H NMR (400 MHz, CD_2_Cl_2_): δ 9.56 (br s), 8.18 (br s), 8.08 (br s), 7.88 (br s), 5.69 (br s), 5.22 (br s), 4.99 (br s), 3.31 (br s), 2.26 (br s). Evans’ Method (CD_2_Cl_2_): μ_eff_ = 2.11 ± 0.07 μ_B_. Elemental analysis for C_41_H_33_CoF_6_I_2_N_2_OP_3_•4CH_2_Cl_2_ (Calcd (Found)): %C, 37.82 (37.44); %H, 2.89 (3.64); %N, 1.96 (1.84).

### Synthesis of (PP^menthoxide^P)­CoI_2_ (**19**)

(PP^menthoxide^P) (**11**) (0.1103 g, 0.1442 mmol, 1 equiv) was dissolved in THF (4 mL) in a nitrogen-filled glovebox. This solution was added to a stirring solution of CoI_2_ (0.0540 g, 0.173 mmol, 1.2 equiv) in THF (3 mL). Upon addition, the bright blue solution immediately turned dark red. This solution was allowed to stir for 24.5 h, then equal amounts of pentane (7 mL) were layered on top of the THF reaction mixture. Crystalline material formed after 72 h at −35 °C. The blue-green mother liquor was decanted, leaving dark red crystals and orange powder. Residual solvent was removed under vacuum, resulting in the isolation of (PP^menthoxide^P)­CoI_2_ (**19**) as both a dark red crystalline material and orange powder (0.054 g, 35% yield). Single crystals suitable for X-ray analysis were grown from a concentrated solution of **19** in C_6_D_6_. ^1^H NMR (400 MHz, CD_2_Cl_2_): δ 11.74 (br s), 9.96 (br s), 9.49 (br s), 8.70 (br s), 8.40 (br s), 8.19 (br s), 8.05 (br s), 7.84 (br s), 6.52 (br s), 6.01 (br s), 5.54 (br s), 5.48 (br s), 4.08 (br s), 3.00 (br s), 2.07 (br s), 1.60 (br s), 0.82 (br s), 0.60 (br s), 0.46 (br s), 0.09 (br s). Evans’ Method (CD_2_Cl_2_): μ_eff_ = 2.56 ± 0.03 μ_b_. Elemental analysis for C_48_H_51_CoI_2_N_2_OP_3_•4CH_2_Cl_2_ (Calcd (Found)): %C, 44.07 (44.19); %H, 4.20 (4.14); %N, 1.98 (2.24).

### Synthesis of (PP^Me^P)­CoI_2_ (**20**)

(PP^Me^P) (**12**) (0.0533 g, 0.0854 mmol, 1 equiv) was dissolved in THF (2 mL) in a nitrogen-filled glovebox. This solution was added to a stirring solution of CoI_2_ (0.032 g, 0.10 mmol, 1.2 equiv) in THF (2 mL). Upon addition, the bright blue solution immediately turned a forest green color and became heterogeneous. This solution was allowed to stir for 16 h before being cooled to −35 °C for 24 h to encourage further solid precipitation. The reaction solution was filtered through a filter pipet and the green solid was collected via suspension in pentane (5 mL). Pentane was removed under vacuum resulting in the isolation of (PP^Me^P)­CoI_2_ (**20**) as an army green powder (0.078 g, 97% yield). Single crystals suitable for X-ray analysis were grown by layer diffusion of pentane into a saturated solution of THF at room temperature. ^1^H NMR (400 MHz, CD_2_Cl_2_): δ 8.93 (br s), 8.06 (br s), 7.31 (br s), 7.00 (br s), 5.67 (br s), 4.79 (br s), 4.07 (br s), 1.99 (br s), 1.62 (br s). Evans’ Method (CD_2_Cl_2_): μ_eff_ = 1.88 ± 0.06 μ_b_. Elemental analysis for C_39_H_35_CoI_2_N_2_P_3_•CH_2_Cl_2_ (Calcd (Found)): %C, 47.00 (47.86); %H, 3.65 (4.31); %N, 2.74 (2.42).

### Synthesis of (FP^Cl^F) (**30**)

*N*,*N*-bis­(2-fluorophenyl)­ethane-1,2-diamine (1.2888 g, 5.19 mmol, 1 equiv) was dissolved in THF (20 mL) in a nitrogen-filled glovebox. This suspension was allowed to stir while NEt_3_ (1.92 mL, 13.8 mmol, 2.66 equiv) was added dropwise via syringe. PCl_3_ (603.9 mL, 6.90 mmol, 1.33 equiv) was added dropwise via syringe, causing a color change from colorless to yellow and the precipitation of a white solid ([HNEt_3_]­[Cl]). The mixture was allowed to stir for 20 h before filtration through a sintered glass frit. The yellow filtrate was concentrated under vacuum and layered with equal parts pentane. Crystallization occurred over 48 h at −35 °C, resulting in a white crystalline substance. The solid was washed with pentane (3 × 1 mL) and subsequently placed under vacuum to remove residual solvent and volatile reagents to afford **30** as a white powder (1.183 g, 72.9% yield). ^1^H NMR (400 MHz, C_6_D_6_): δ 7.10–6.95 (m, 2H), 6.87–6.70 (m, 4H), 6.69–6.58 (m, 2H), 3.50–3.35 (m, 2H), 3.26–3.10 (m, 2H). ^31^P­{^1^H} NMR (162 MHz, C_6_D_6_): δ 143.39 (t, *J*_P–F_ = 110.6 Hz, 1P). ^19^F NMR (377 MHz, C_6_D_6_): δ −119.63 (m, *J*_F–P_ = 110.9 Hz, 2F). ^13^C­{^1^H} NMR (151 MHz, C_6_D_6_): δ 155.53 (dd, ^1^ *J*_C–F_ = 246.7 Hz, ^3^ *J*_C–P_ = 3.7 Hz), 130.70 (m), 124.96 (m), 124.5 (m), 122.17 (m), 116.80 (m), 49.19 (m).

### Synthesis of (FP^NEt2^F) (**31**)

(FP^Cl^F) (**30**) (0.3562 g, 1.14 mmol, 1 equiv) was dissolved in THF (4 mL) in a nitrogen-filled glovebox. The solution was allowed to stir while HNEt_2_ (353.5 mL, 3.42 mmol, 3.00 equiv) was added via micropipette, resulting in the gradual precipitation of a white powder ([H_2_NEt_2_]­[Cl]). The reaction stirred for a total of 22.5 h before filtration through a Celite-packed pipet and concentration of the filtrate under vacuum. Concentration resulted in the precipitation of a white powder that was subsequently triturated with pentane (3 × 2 mL) to remove residual solvent, yielding pure **31** as a white powder (0.349 g, 87.8% yield). ^1^H NMR (400 MHz, C_6_D_6_): δ 7.20–7.12 (m, signals overlapping with C_6_D_5_H, 2H), 7.01–6.82 (m, 4H), 6.70–6.58 (m, 2H), 3.78–3.64 (m, 2H), 3.26–3.11 (m, 2H), 2.94 (dq, ^3^ *J*_H–H_ = 7.1 Hz, ^3^ *J*_H–P_ = 8.4 Hz, 4H), 0.75 (t, ^3^ *J*_H–H_ = 7.1 Hz, 6H). ^31^P­{^1^H} NMR (162 MHz, C_6_D_6_): δ 105.15 (t, *J*_P–F_ = 87.6 Hz, 1P). ^19^F NMR (377 MHz, C_6_D_6_): δ −119.74 (m, *J*_P–F_ = 87.4 Hz, 2F). ^13^C­{^1^H} NMR (151 MHz, C_6_D_6_): δ 155.55 (dd, ^1^ *J*_C–F_ = 244.4 Hz, ^3^ *J*_C–P_ = 2.5 Hz), 134.74 (m), 124.65 (m), 122.03 (m), 121.55 (m), 116.89 (d, ^2^ *J*_C–P_ = 21.5 Hz), 48.86 (m), 40.08 (d, ^2^ *J*_C–P_ = 20.2 Hz), 14.76 (d, ^3^ *J*_C–P_ = 2.76 Hz).

### Synthesis of (FP^N*i*Pr2^F) (**32**)

(FP^Cl^F) (**30**) (0.3384 g, 1.08 mmol, 1 equiv) was dissolved in THF (4 mL) in a nitrogen-filled glovebox. The solution was allowed to stir while HN^*i*^Pr_2_ (458.2 mL, 3.25 mmol, 3.0 equiv) was added via micropipette, resulting in the gradual precipitation of a white powder ([NH_2_^*i*^Pr_2_]­[Cl]). The reaction was stirred for a total of 22.5 h before filtration through a Celite-packed pipet and concentration of the filtrate under vacuum. The resultant brown oil was layered with 1 mL of pentane and cooled to −35 °C for 24 h. This resulted in the precipitation of an off-white powder that was subsequently triturated with pentane (3 × 2 mL) to remove residual solvent and left under vacuum for 4 h, yielding **32** as a white powder (0.386 g, 94.5% yield). ^1^H NMR (400 MHz, C_6_D_6_): δ 7.47–7.36 (m, 2H), 7.00–6.81 (m, overlapping signals, 4H), 6.70–6.58 (m, 2H), 4.04–3.90 (m, 2H), 3.30 (dsept, ^3^ *J*_H–H_ = 6.9 Hz, ^3^ *J*_H–P_ = 9.8 Hz, 2H), 3.16–3.02 (m, 2H), 0.96 (d, ^3^ *J*_H–H_ = 6.9 Hz, 12H). ^31^P­{^1^H} NMR (162 MHz, C_6_D_6_): δ 93.39 (t, *J*_P–F_ = 56.1 Hz, 1P). ^19^F NMR (377 MHz, C_6_D_6_): δ −120.00 (m, *J*_P–F_ = 56.4 Hz, 2F). ^13^C­{^1^H} NMR (151 MHz, C_6_D_6_): δ 156.67 (dd, ^1^ *J*_C–F_ = 245.3 Hz, ^3^ *J*_C–P_ = 2.7 Hz), 134.59 (m), 124.37 (m), 122.20 (m), 116.73 (d, ^2^ *J*_C–P_ = 21.3 Hz), 49.51 (m), 45.0 (d, ^2^ *J*_C–P_ = 10.9 Hz), 24.40 (d, ^3^ *J*_C–P_ = 7.1 Hz).

### Synthesis of (FP^OEt^F) (**33**)

(FP^Cl^F) (**30**) (0.3700 g, 968.7 mmol, 1 equiv) was dissolved in THF (5 mL) in a nitrogen-filled glovebox. To this was added NEt_3_ (362.9 mL, 2.13 mmol, 2.2 equiv) via micropipette. This solution was allowed to stir for 2 min before the addition of EtOH (76.0 mL,1.07 mmol, 1.1 equiv) by micropipette, which resulted in the immediate precipitation of a white powder ([NHEt_3_]­[Cl]). The reaction was stirred for a total of 20.5 h before filtration through a Celite-packed pipet. Concentration of the filtrate under vacuum provided a yellow oil that was cooled to −35 °C for 30 min. This encouraged the precipitation of a white powder that was subsequently triturated with pentane (3 × 2 mL) and placed under vacuum for 4 h to remove residual solvent, yielding pure **33** (0.339 g, 88.9% yield). ^1^H NMR (400 MHz, C_6_D_6_): δ 7.10–6.75 (m, overlapping signals, 6H), 6.70–6.57 (m, 2H), 3.71 (dq, ^3^ *J*_H–H_ = 7.0 Hz, ^3^ *J*_H–P_ = 7.7 Hz, 2H), 3.55–3.40 (m, 2H), 3.31–3.16 (m, 2H), 0.94 (t, ^3^ *J*_H–H_ = 7.0 Hz, 3H). ^31^P­{^1^H} NMR (162 MHz, C_6_D_6_): δ 116.59 (t, *J*_P–F_ = 104.4 Hz, 1P). ^19^F NMR (377 MHz, C_6_D_6_): δ −119.93 (m, *J*_P–F_ = 104.4 Hz, 2F). ^13^C­{^1^H} NMR (151 MHz, C_6_D_6_): δ 155.24 (dd, ^1^ *J*_C–F_ = 244.8, ^3^ *J*_C–P_ = 2.8 Hz), 133.86 (m), 124.82 (m), 122.20 (m), 121.08 (m), 116.63 (d, ^2^ *J*_C–P_ = 20.5 Hz), 60.34 (d, ^2^ *J*_C–P_ = 10.8 Hz), 48.92 (m), 17.18 (d, ^3^ *J*_C–P_ = 4.2 Hz).

### Synthesis of (FP^O*i*Pr^F) (**34**)

(FP^Cl^F) (**30**) (0.3029 g, 968.7 mmol, 1 equiv) was dissolved in THF (4 mL) in a nitrogen-filled glovebox. To this was added NEt_3_ (297.1 mL, 2.13 mmol, 2.2 equiv) via micropipette. This solution was allowed to stir for 2 min before the addition of ^*i*^PrOH (81.5 mL,1.07 mmol, 1.1 equiv) by micropipette, which resulted in the immediate precipitation of a white powder ([NHEt_3_]­[Cl]). The reaction was stirred for a total of 20.5 h before filtration through a Celite-packed pipet and concentration of the filtrate under vacuum, resulting in an off-white powder. This powder was triturated with pentane (3 × 3 mL), removing residual solvent and providing clean **34** (0.310 g, 95.3% yield). ^1^H NMR (400 MHz, C_6_D_6_): δ 7.06–6.77 (m, overlapping signals, 6H), 6.71–6.56 (m, 2H), 4.28 (dsept, ^3^ *J*_H–H_ = 6.3 Hz, ^3^ *J*_H–P_ = 9.2 Hz, 1H), 3.58–3.42 (m, 2H), 3.22–3.08 (m, 2H), 0.98 (d, ^3^ *J*_H–H_ = 6.1 Hz, 6H). ^31^P­{^1^H} NMR (162 MHz, C_6_D_6_): δ 119.41 (t, *J*_P–F_ = 115.9 Hz, 1P). ^19^F NMR (377 MHz, C_6_D_6_): δ −119.75 (m, *J*_P–F_ = 117.3 Hz, 2F). ^13^C­{^1^H} NMR (151 MHz, C_6_D_6_): δ 155.00 (dd, ^1^ *J*_C–F_ = 244.1, ^3^ *J*_C–P_ = 2.4 Hz), 133.99 (m), 124.77 (m), 122.80 (m), 120.71 (m), 116.54 (d, ^2^ *J*_C–P_ = 20.9 Hz), 68.12 (d, ^2^ *J*_C–P_ = 17.7 Hz), 48.17 (m), 24.76 (d, ^3^ *J*_C–P_ = 3.2 Hz).

### Synthesis of (FP^OCH2CF3^F) (**35**)

(FP^Cl^F) (**30**) (0.2068 g, 661.4 mmol, 1 equiv) was dissolved in THF (4 mL) in a nitrogen-filled glovebox. To this was added NEt_3_ (202.8 mL, 1.46 mmol, 2.2 equiv) via micropipette. This solution was allowed to stir for 2 min before the addition of trifluoroethanol (76.9 mL, 1.07 mmol, 1.61 equiv) by micropipette, which resulted in the immediate precipitation of a white powder ([NHEt_3_]­[Cl]). The reaction continued to stir for a total of 21 h before filtration through a Celite-packed pipet and concentration of the filtrate under vacuum, resulting in an off-white powder. This powder was triturated with pentane (3 × 3 mL), removing residual solvent and providing clean **35** (0.213 g, 85.5% yield). ^1^H NMR (400 MHz, C_6_D_6_): δ 6.96–6.74 (m, overlapping signals, 6H), 6.71–6.58 (m, 2H), 3.84 (dq, ^3^ *J*_H–F_ = 7.3 Hz, ^3^ *J*_H–P_ = 8.5 Hz, 2H), 3.40–3.21 (m, 2H), 3.16–3.00 (m, 2H). ^31^P­{^1^H} NMR (162 MHz, C_6_D_6_): δ 123.56 (t, *J*_P–F_ = 111.9 Hz, 1P). ^19^F NMR (377 MHz, C_6_D_6_): δ −73.95 (m, ^4^ *J*_F–P_ = 9.0 Hz, 3F), −120.33 (m, *J*_F–P_ = 111.9 Hz, 2F). ^13^C­{^1^H} NMR (151 MHz, C_6_D_6_): δ 155.23 (dd, ^1^ *J*_C–F_ = 244.3 Hz, ^3^ *J*_C–P_ = 3.3 Hz), 132.76 (m), 124.89 (m), 124.42 (dq, ^1^ *J*_C–F_ = 276.2 Hz, ^3^ *J*_C–P_ = 5.8 Hz), 122.93 (m), 121.16 (m), 116.51 (d, *J*_C–P_ = 20.7 Hz), 62.25 (dq, ^2^ *J*_C–F_ = 35.7 Hz, ^2^ *J*_C–P_ = 15.4 Hz), 48.46 (m).

### Synthesis of (FP^OCH(CF3)2^F) (**36**)

(FP^Cl^F) (**30**) (0.3947 g, 1.26 mmol, 1 equiv) was dissolved in THF (4 mL) in a nitrogen-filled glovebox. To this was added NEt_3_ (387.1 mL, 2.78 mmol, 2.2 equiv). This solution was allowed to stir for 2 min before the addition of hexafluoroisopropanol (146.8 mL, 1.39 mmol, 1.1 equiv), which resulted in the immediate precipitation of a white powder ([NHEt_3_]­[Cl]). The reaction was allowed to stir for a total of 20.5 h before filtration through a Celite-packed pipet and concentration of the filtrate under vacuum, resulting in an off-white powder. This powder was triturated with pentane (3 × 3 mL), removing residual solvent and providing clean **36** (0.423 g, 75.4% yield). ^1^H NMR (400 MHz, C_6_D_6_): δ 6.99–6.73 (m, overlapping signals, 6H), 6.72–6.60 (m, 2H), 4.50 (dsept, ^3^ *J*_H–F_ = 6.0 Hz, ^3^ *J*_H–P_ = 8.5 Hz, 2H), 3.46–3.32 (m, 2H), 3.01–2.85 (m, 2H). ^31^P­{^1^H} NMR (162 MHz, C_6_D_6_): δ 123.56 (tsept, *J*_P–F_ = 126.6 Hz, ^4^ *J*_P–F_ = 7.2 Hz, 1P). ^19^F NMR (377 MHz, C_6_D_6_): δ −73.26 (m, 6F), −119.8 (d, *J*_F–P_ = 128.0 Hz, 2F). ^13^C­{^1^H} NMR (151 MHz, C_6_D_6_): δ 155.23 (dd, ^1^ *J*_C–F_ = 244.8, ^3^ *J*_C–P_ = 2.8 Hz), 131.99 (m), 124.87 (m), 123.61 (m), 121.90 (q, ^1^ *J*_C–F_ = 283.4 Hz), 121.70 (m), 116.44 (d, ^2^ *J*_C–P_ = 20.1 Hz), 71.10 (dsept, ^2^ *J*_C–F_ = 33.1 Hz, ^2^ *J*_C–P_ = 18.4 Hz), 48.19 (m).

### Synthesis of (FP^Me^F) (**37**)

(FP^Cl^F) (**30**) (0.3130 g, 1.00 mmol, 1 equiv) was dissolved in THF (4 mL) in a nitrogen-filled glovebox. This was allowed to stir while MeMgBr (786.5 mL, 1.4 M in 1:3 THF:toluene, 1.1 mmol, 1.1 equiv) was added dropwise via syringe. This resulted in no color change from the yellow reaction solution. The solution was allowed to stir for a total of 21 h before the addition of dioxane (3 mL) to precipitate MgBrCl salts. The suspension was filtered through a sintered glass frit to remove any MgBrCl and the remaining filtrate was concentrated to afford an orange-brown oil. Trituration of the oil with pentane (3 × 3 mL) resulting in the precipitation of a yellow-white powder. Further trituration was performed with pentane (1 × 3 mL), eventually resulting in **37** as an off-white powder (0.098 g, 33% yield). ^1^H NMR (400 MHz, C_6_D_6_): δ 6.97–6.68 (m, signals overlapping, 6H), 6.63–6.51 (m, 2H), 3.18 (s, 4H), 1.23 (dt, ^2^ *J*_H–P_ = 7.8 Hz, *J*_H–F_ = 1.5 Hz, 3H). ^31^P­{^1^H} NMR (162 MHz, C_6_D_6_): δ 111.72 (t, *J*_P–F_ = 120.4 Hz, 1P). ^19^F NMR (377 MHz, C_6_D_6_): δ −122.12 (m, *J*_P–F_ = 118.9 Hz, 2F). ^13^C­{^1^H} NMR (151 MHz, C_6_D_6_): δ 154.49 (dd, ^1^ *J*_C–F_ = 242.0, ^3^ *J*_C–P_ = 2.8 Hz), 135.84 (m), 124.75 (m), 120.73 (m), 119.37 (m), 116.26 (m), 48.12 (m), 18.72 (d, ^1^ *J*_C–P_ = 38.4 Hz).

### Synthesis of (FP^CF3^F) (**38**)

(FP^Cl^F) (**30**) (0.499 g, 1.60 mmol, 1 equiv) was suspended in a minimal amount of THF (4 mL) in a nitrogen-filled glovebox. To this stirring mixture, 18-crown-6 (84.36 mg, 0.319 mmol, 0.2 equiv) and KF (101.99 mg, 1.76 mmol, 1.1 equiv) were added in that respective order, and the mixture was allowed to stir for 15 min. TMSCF_3_ (295 mg, 2.07 mmol, 1.3 equiv) was then added to the stirring solution and the mixture was warmed to 40 °C. Reaction progress was monitored by ^19^F NMR spectroscopy for disappearance of TMSCF_3_ and the formation of **38**. After 5 d, the TMSCF_3_ was consumed but the formation of **38** was incomplete so additional TMSCF_3_ (295 mg, 2.07 mmol, 1.3 equiv) was added to the warmed stirring solution. The reaction was allowed to stir an additional 24 h before filtration through a Celite-packed pipet and concentration under vacuum. This resulted in the precipitation of a tan powder that was subsequently dissolved in minimal Et_2_O (1 mL) followed by the addition of pentane (10 mL) and stirring for 24 h. The mixture was then cooled to −35 °C for 24 h, resulting in the recrystallization of **38**. This recrystallization process was repeated two more times to afford pure **38** in a tan crystalline form (0.058 g, 10% yield). ^1^H NMR (400 MHz, C_6_D_6_): δ 6.87–6.68 (m, 6H), 6.64–6.51 (m, 2H), 3.44–3.29 (m, 2H), 3.14–2.99 (m, 2H). ^31^P­{^1^H} NMR (162 MHz, C_6_D_6_): δ 78.65 (tq, *J*_P–F_ = 125.6 Hz, ^2^ *J*_P–F_ = 48.2 Hz, 1P). ^19^F­{^1^H} NMR (377 MHz, C_6_D_6_): δ −64.50 (dt, ^2^ *J*_F–P_ = 48.0 Hz, *J*_F–F_ = 6.4 Hz, 3F), −120.22 (dq, *J*_F–P_ = 124.7 Hz, *J*_F–F_ = 6.3 Hz, 2F). ^13^C­{^1^H} NMR (151 MHz, C_6_D_6_): δ 154.89 (dd, ^1^ *J*_C–F_ = 244.1, ^3^ *J*_C–P_ = 2.2 Hz), 132.77 (m), 124.89 (m), 123.15 (m), 121.30 (m), 116.74 (m), 49.36 (m). Due to coupling to ^19^F and poor signal-to-noise, the CF_3_ carbon was not resolved in the ^13^C­{^1^H} NMR spectrum.

### Synthesis of (F­(Se=)­P^NEt2^F) (**39**)

Compound **31** (0.1161 g, 332.3 mmol, 1 equiv) was dissolved in CHCl_3_ (2 mL) in a nitrogen-filled glovebox. This solution was added to a suspension of Se^0^ (0.0314 g, 398 mmol, 1.2 equiv) in CHCl_3_ (2 mL). This suspension was allowed to stir at 50 °C for 48 h. The remaining Se^0^ was removed via filtration through a Celite-packed pipet, and the resulting filtrate was concentrated under vacuum. Trituration of the resultant oil with pentane gave an off-white powder that was recrystallized under ambient conditions via a layer diffusion of Et_2_O and pentane (2 mL, respectively) at −35 °C over 48 h. The remaining mother liquor was decanted from the white, crystalline solid. The solid was placed under vacuum to remove any residual solvent, providing **39** (0.073 g, 52% yield). ^1^H NMR (400 MHz, CDCl_3_): δ 8.11–7.95 (m, 2H), 7.20–7.02 (m, 6H), 4.04–3.91 (m, 2H), 3.81–3.70 (m, 2H), 3.30 (dq, ^3^ *J*_H–H_ = 7.2 Hz, ^3^ *J*_H–P_ = 15.0 Hz, 4H), 0.80 (t, ^3^ *J*_H–H_ = 7.2 Hz, 6H). ^31^P­{^1^H} NMR (162 MHz, CDCl_3_): δ 62.08 (s with d satellites, ^1^ *J*_P–Se_ = 832.5 Hz, 1P). ^19^F NMR (377 MHz, CDCl_3_): δ −119.57 (m, 2F). ^13^C­{^1^H} NMR (151 MHz, CDCl_3_): δ 158.16 (dd, ^1^ *J*_C–F_ = 247.8 Hz, ^3^ *J*_C–P_ = 4.5 Hz), 129.18 (m), 127.42 (m), 126.41 (m), 124.28 (m), 116.52 (d, ^2^ *J*_C–P_ = 5.1 Hz), 47.73 (m), 39.92 (d, ^2^ *J*_C–P_ = 20.7 Hz), 13.70 (d, ^3^ *J*_C–P_ = 2.2 Hz).

### Synthesis of (F­(Se=)­P^N*i*Pr2^F) (**40**)

Compound **32** (0.1426 g, 377.8 mmol, 1 equiv) was dissolved in CHCl_3_ (2 mL) in a nitrogen-filled glovebox. This solution was combined with Se^0^ (0.0356 g, 451 mmol, 1.2 equiv) suspended in CHCl_3_ (2 mL) and allowed to stir at 50 °C for 48 h. Following the 48 h reaction period, the solution was filtered through a Celite-packed pipet to remove excess Se^0^. The resulting filtrate was concentrated under vacuum to give an off-white powder. Under ambient conditions, minimal ethyl acetate was added (0.5 mL) and layered with pentane (2 mL). Recrystallization occurred at −35 °C over 48 h, providing **40** as a pinkish-white crystalline solid (0.126 g, 73.7% yield). A minor (<5%) impurity was observed in the ^1^H and ^31^P NMR spectra of **40**, but since its presence did not impact the measured ^1^ *J*_P–Se_, further purification steps were not performed. ^1^H NMR (400 MHz, CDCl_3_): δ 8.72–8.47 (m, 2H), 7.17–6.98 (m, 6H), 4.16–4.04 (m, 2H), 3.97 (dsept, ^3^ *J*_H–H_ = 7.0 Hz, ^3^ *J*_H–P_ = 20.1 Hz, 2H), 3.70–3.59 (m, 2H), 1.06 (d, ^3^ *J*_H–H_ = 6.8 Hz, 12H). ^31^P­{^1^H} NMR (162 MHz, CDCl_3_): δ 49.50 (s with d satellites, ^1^ *J*_P–Se_ = 824.6 Hz, 1P). ^19^F NMR (377 MHz, CDCl_3_): δ −119.47 (m, 2F). ^13^C­{^1^H} NMR (151 MHz, CDCl_3_): δ 157.70 (dd, ^1^ *J*_C–F_ = 248.1 Hz, ^3^ *J*_C–P_ = 6.2 Hz), 130.07 (m), 126.22 (m), 125.75 (m), 124.00 (m), 116.44 (d, ^2^ *J*_C–P_ = 20.5 Hz), 48.13 (d, ^2^ *J*_C–P_ = 6.4 Hz), 47.26 (m), 23.03 (d, ^3^ *J*_C–P_ = 2.0 Hz).

### Synthesis of (F­(Se=)­P^OEt^F) (**41**)

Compound **33** (0.1043 g, 323.6 mmol, 1 equiv) was dissolved in CHCl_3_ (2 mL) in a nitrogen-filled glovebox. This solution was combined with Se^0^ (0.0310 g, 392 mmol, 1.2 equiv) suspended in CHCl_3_ (2 mL) and allowed to stir at 50 °C for 48 h. Following the 48 h reaction period, the solution was filtered through a Celite-packed pipet to remove excess Se^0^. The resulting filtrate was concentrated under vacuum and triturated with pentane (3 × 3 mL) to give **41** as a fine white powder (0.100 g, 77.1% yield). ^1^H NMR (400 MHz, CDCl_3_): δ 7.68–7.56 (m, 2H), 7.26–7.09 (m, 6H), 4.08 (dq, ^3^ *J*_H–H_ = 6.9 Hz, ^3^ *J*_H–P_ = 11.0 Hz, 2H), 4.01–3.8 (m, 4H), 1.18 (t, ^3^ *J*_H–H_ = 7.0 Hz, 3H). ^31^P­{^1^H} NMR (162 MHz, CDCl_3_): δ 68.33 (s with d satellites, ^1^ *J*_P–Se_ = 905.8 Hz, 1P). ^19^F NMR (377 MHz, CDCl_3_): δ −118.69 (m, 2F). ^13^C­{^1^H} NMR (151 MHz, CDCl_3_): δ 158.60 (dd, ^1^ *J*_C–F_ = 249.1 Hz, ^3^ *J*_C–P_ = 4.7 Hz), 128.23 (m), 127.43 (m), 124.64 (m), 116.75 (d, ^2^ *J*_C–P_ = 20.3 Hz), 65.58 (d, ^2^ *J*_C–P_ = 7.6 Hz), 48.87 (m), 15.91 (d, ^3^J_C–P_ = 7.8 Hz).

### Synthesis of (F­(Se=)­P^O*i*Pr^F) (**42**)

Compound **34** (0.1139 g, 338.7 mmol, 1 equiv) was dissolved in CHCl_3_ (2 mL) in a nitrogen-filled glovebox. This solution was combined with Se^0^ (0.0321 g, 406 mmol, 1.2 equiv) suspended in CHCl_3_ (2 mL) and allowed to stir at 50 °C for 48 h. Following the 48 h reaction period, the solution was filtered through a Celite-packed pipet to remove excess Se^0^. The resulting filtrate was concentrated under vacuum and triturated with pentane (3 × 3 mL) to give **42** as a fine white powder (0.087 g, 62% yield). ^1^H NMR (400 MHz, CDCl_3_): δ 7.72–7.62 (m, 2H), 7.25–7.08 (m, 6H), 4.61 (dsept, ^3^ *J*_H–H_ = 6.4 Hz, ^3^ *J*_H–P_ = 12.7 Hz, 1H), 4.03–3.92 (m, 2H), 3.89–3.77 (m, 2H), 1.08 (d, ^3^ *J*_H–H_ = 6.2 Hz, 6H). ^31^P­{^1^H} NMR (162 MHz, CDCl_3_): δ 67.43 (s with d satellites, ^1^ *J*_P–Se_ = 897.1 Hz, 1P). ^19^F NMR (377 MHz, CDCl_3_): δ −117.81 (m, 2F). ^13^C­{^1^H} NMR (151 MHz, CDCl_3_): δ 158.96 (dd, ^1^ *J*_C–F_ = 249.8 Hz, ^3^ *J*_C–P_ = 4.5 Hz), 129.17 (m), 128.21 (m), 127.57 (m), 124.43 (m), 116.70 (d, ^2^ *J*_C–P_ = 20.3 Hz), 74.88 (d, ^2^ *J*_C–P_ = 6.6 Hz), 48.72 (m), 23.31 (d, ^3^ *J*_C–P_ = 5.0 Hz).

### Synthesis of (F­(Se=)­P^OCH2CF3^F) (**43**)

Compound **35** (0.0643 g, 171 mmol, 1 equiv) was dissolved in CHCl_3_ (2 mL) in a nitrogen-filled glovebox. This solution was combined with Se^0^ (0.0281 g, 357 mmol, 2.09 equiv) suspended in CHCl_3_ (1 mL) and allowed to stir at 50 °C for 162.5 h. Following the 162.5 h reaction period, the solution was filtered through a Celite-packed pipet to remove excess Se^0^. The resulting filtrate was concentrated under vacuum and triturated with pentane (1 × 3 mL) to give **43** as a fine white powder (0.072 g, 92% yield). A minor (<5%) impurity was observed in the ^1^H and ^19^F NMR spectra of **43**, but since its presence did not impact the measured ^1^ *J*_P–Se_, further purification steps were not performed. ^1^H NMR (400 MHz, CDCl_3_): δ 7.53–7.45 (m, 2H), 7.29–7.21 (m, overlapping with CHCl_3_ signal, 2H), 7.20–7.12 (m, 4H), 4.27 (dq, ^3^ *J*_H–F_ = 8.5 Hz, ^3^ *J*_H–P_ = 9.9 Hz, 2H), 4.04–3.86 (m, 4H). ^31^P­{^1^H} NMR (162 MHz, CDCl_3_): δ 72.38 (d, ^1^ *J*_P–Se_ = 933.6 Hz, 1P). ^19^F NMR (377 MHz, CDCl_3_): δ −73.24 (t, *J*_F–F_ = 1.7 Hz, 3F), −118.56 (m, 2F). ^13^C­{^1^H} NMR (151 MHz, CDCl_3_): δ 158.83 (dd, ^1^ *J*_C–F_ = 249.1 Hz, ^3^ *J*_C–P_ = 4.4 Hz), 128.81 (m), 127.58 (m), 124.79 (m), 116.82 (d, ^2^ *J*_C–P_ = 20.4 Hz), 65.54 (q, ^2^ *J*_C–F_ = 36.1 Hz), 49.04 (m).

### Synthesis of (F­(Se=)­P^OCH(CF3)2^F) (**44**)

Compound **36** (0.3573 g, 170.9 mmol, 1 equiv) was dissolved in CHCl_3_ (2 mL) in a nitrogen-filled glovebox. This solution was combined with Se^0^ (0.1016 g, 357.2 mmol, 2.09 equiv) suspended in CHCl_3_ (1 mL) and allowed to stir at 25 °C for 168 h. Following the 168 h reaction period, the solution was filtered through a Celite-packed pipet to remove excess Se^0^. The resulting filtrate was concentrated under vacuum and subjected to silica flash column chromatography under ambient conditions (hexanes/ethyl acetate gradient: 0–100% ethyl acetate). Fractions were collected and the solvent was removed under vacuum, yielding **44** as a fine white powder (0.095 g, 23% yield). ^1^H NMR (400 MHz, CDCl_3_): δ 7.68–7.56 (m, 2H), 7.35–7.27 (m, 2H), 6.21–7.10 (m, 4H), 5.25 (dsept, ^3^ *J*_H–F_ = 5.8 Hz, ^3^ *J*_H–P_ = 17.1 Hz, 1H), 4.22–4.01 (m, 2H), 3.87–3.64 (m, 2H). ^31^P­{^1^H} NMR (162 MHz, CDCl_3_): δ 74.87 (s with d satellites, ^1^ *J*_P–Se_ = 950.0 Hz, 1P). ^19^F NMR (377 MHz, CDCl_3_): δ −72.25 (s, 6F), −118.04 (s, 2F). ^13^C­{^1^H} NMR (151 MHz, CDCl_3_): δ 160.16 (dd, ^1^ *J*_C–F_ = 251.1 Hz, ^4^ *J*_C–P_ = 4.5 Hz), 131.55 (m), 129.33 (m), 126.50 (m), 124.79 (m), 116.72 (d, ^2^ *J*_C–P_ = 19.9 Hz), 73.46 (dsept, ^2^ *J*_C–F_ = 33.4 Hz, ^2^ *J*_C–P_ = 2.7 Hz), 49.71 (m).

### Synthesis of (F­(Se=)­P^Me^F) (**45**)

Compound **37** (0.1023 g, 350.0 mmol, 1 equiv) was dissolved in CHCl_3_ (2 mL) in a nitrogen-filled glovebox. This solution was combined with Se^0^ (0.0557 g, 706 mmol, 2.02 equiv) suspended in CHCl_3_ (1 mL) and allowed to stir at 50 °C for 162 h. Following the 162 h reaction period, the solution was filtered through a Celite-packed pipet to remove excess Se^0^. The resulting filtrate was concentrated under vacuum and triturated with pentane (1 × 3 mL) to give a white powder. The powder was subsequently dissolved in minimal Et_2_O (1.5 mL) and cooled at −35 °C for 24 h. The mother liquor was decanted away from the resulting white, crystalline material. The solid crystals were placed under vacuum to remove residual solvent and afford pure **45** (0.087 g, 67% yield). A minor (<5%) impurity was observed in the ^1^H and ^19^F NMR spectra of **45**, but since its presence did not impact the measured ^1^ *J*_P–Se_, further purification steps were not performed. ^1^H NMR (400 MHz, CDCl_3_): δ 7.74–7.59 (m, 2H), 7.26–7.09 (m, 6H), 3.93–3.77 (m, 4H), 2.21 (dt, ^2^ *J*_H–P_ = 13.3 Hz, *J*_H–F_ = 1.9 Hz). ^31^P­{^1^H} NMR (162 MHz, C_6_D_6_): δ 77.96 (s with d satellites, ^1^ *J*_P–Se_ = 807.0 Hz, 1P). ^19^F NMR (377 MHz, CDCl_3_): δ −118.25 (d, *J*_F–P_ = 2.1 Hz, 2F). ^13^C­{^1^H} NMR (151 MHz, CDCl_3_): δ 160.16 (dd, ^1^ *J*_C–F_ = 247.9 Hz, ^4^ *J*_C–P_ = 3.8 Hz), 129.04 (m), 128.63 (m), 127.63 (m), 124.84 (m), 116.65 (d, ^2^ *J*_C–P_ = 20.4 Hz), 73.46 (dsept, ^2^ *J*_C–F_ = 33.4 Hz, ^2^ *J*_C–P_ = 2.7 Hz), 49.18 (m), 29.37 (dt, ^1^ *J*_C–P_ = 72.8 Hz, *J*_C–F_ = 3.9 Hz).

### Synthesis of (F­(Se=)­P^CF3^F) (**46**)

Compound **38** (0.0581 g, 167.51 mmol, 1 equiv) was dissolved in CHCl_3_ (2 mL) in a nitrogen-filled glovebox. This solution was combined with Se^0^ (0.0268 g, 339.37 mmol, 2.03 equiv) suspended in CHCl_3_ (1 mL) and allowed to stir at 50 °C for 21 d. Following the 21 d reaction period, the solution was filtered through a Celite-packed pipet to remove excess Se^0^. The resulting filtrate was concentrated under vacuum and triturated with pentane (1 × 3 mL) to give a white powder. This was subsequently dissolved in minimal Et_2_O (1.5 mL) and cooled at −35 °C for 24 h. The mother liquor was decanted away from the now white, crystalline material. The solid crystals were placed under vacuum to remove residual solvent and provide **46** (0.052 g, 73% yield). ^1^H NMR (400 MHz, CDCl_3_): δ 7.64–7.53 (m, 2H), 7.38–7.27 (m, 2H), 7.24–7.09 (m, 4H), 4.14–3.96 (m, 2H), 3.94–3.78 (m, 2H). ^31^P­{^1^H} NMR (162 MHz, CDCl_3_): δ 55.37 (q with dq satellites, ^1^ *J*_P–Se_ = 912.5 Hz, ^2^ *J*_P–F_ = 102.3 Hz, 1P). ^19^F NMR (377 MHz, CDCl_3_): δ −72.1 (dt, ^2^ *J*_F–P_ = 101.8 Hz, *J*_F–F_ = 3.8 Hz, 3F), −119.6 (m, 2F). ^13^C­{^1^H} NMR (151 MHz, CDCl_3_): δ 160.08 (dd, ^1^ *J*_C–F_ = 250.9 Hz, ^3^ *J*_C–P_ = 4.1 Hz), 131.28 (m), 129.48 (m), 126.96 (m), 124.77 (m), 123.76 (dq, ^1^ *J*_C–F_ = 321.8 Hz, ^1^ *J*_C–P_ = 162.15 Hz), 116.96 (d, ^2^ *J*_C–P_ = 20.0 Hz), 50.00 (m).

### Cyclic Voltammetry

Cyclic voltammetry measurements were conducted in a nitrogen-filled glovebox using a 0.1 M [^n^Bu_4_N]­[PF_6_] electrolyte solution in THF using a CHI 620 E potentiostat (CH Instruments Inc., Austin, TX). A glassy carbon electrode was used for the working electrode, a platinum wire was used for the counter electrode, and a Ag/Ag^+^ reference electrode was used with 0.01 M AgNO_3_/0.1 M [^n^Bu_4_N]­[PF_6_] as the reference solution.

### EPR Spectroscopy

Low-temperature (40 K) continuous-wave (CW) X-band EPR spectra were collected using a Bruker EMXPlus spectrometer equipped with a ColdEdge cryogen-free helium cryostat and recirculation system and an Oxford Instruments MercuryITC temperature controller. Spectra were obtained under nonsaturating conditions at 0.5 mW using a microwave frequency of 9.37 GHz and a modulation frequency and amplitude of 100 kHz and 6 G, respectively. Room-temperature (298 K) CW X-band EPR spectra were collected using a Bruker ESR5000 spectrometer. Spectra were obtained under nonsaturating conditions at 10 mW using a microwave frequency of 9.45 GHz and a modulation frequency and amplitude of 100 kHz and 10 G, respectively. Background signals were removed by baseline subtraction using IGOR Pro 9.00 (Wavemetrics, Lake Oswego, OR). EPR spectral simulations were performed using the EasySpin (ver. 5.2.36) toolbox within MATLAB.^27^

### Computational Studies

All calculations were performed with the ORCA 5.0.4 software package^74,75^ using resources provided by the Ohio Supercomputer Center.^76^ The atomic coordinates from the solid-state structures of **3** ^64^ and **13**–**20** were used as a starting point and modified by deletion of the two cobalt-bound halides and the addition of a hydride and carbonyl ligand. Geometry optimizations and frequency calculations were performed using the ωB97X-D3 functional and def2-SVP basis set.^77–79^ Numerical frequency calculations were performed on all optimized geometries to ensure the absence of significant imaginary frequencies and to provide the ν­(CO) stretching frequency of interest.

### Single Crystal X-ray Diffraction

The single-crystal X-ray diffraction studies were carried out on a Bruker Kappa Photon III CPAD diffractometer equipped with Mo Kα radiation (λ = 0.71073 Å). Data were collected in a nitrogen gas stream at 100(2) K using ϕ and ϖ scans. The data were integrated using the Bruker SAINT software program and scaled using the SADABS software program. Solution by the dual-space method (SHELXT) produced a complete phasing model for refinement. %V_bur_ values for each ligand were obtained by analyzing the solid-state structures of **13**–**20** and **3** ^64^ in ChimeraX using the SEQCROW plug-in.^80,81^

### General Procedure for Kinetic Studies

A 20 mL scintillation vial was charged with (PP^R^P)­CoI_2_ precatalyst (0.1 or 1.0 mol %) and a stir bar in a N_2_-filled glovebox. To this were added C_6_H_6_, styrene (1 equiv), HBpin (1.1 equiv), and KBEt_3_H (0.2 or 2.0 mol %). Upon addition of KBEt_3_H, the reaction was allowed to stir and a timer was started. Aliquots for kinetic time points were collected by dipping the tip of a Pasteur pipet into a stirring reaction solution at the appropriate time as indicated by a running timer. This portion of the reaction solution was diluted into 2 mL of C_6_H_6_ and removed from the N_2_ glovebox. 1–3 drops of this new solution were added to a 2 mL GC-MS vial filled with C_6_H_6_.

## Supplementary Material





## Notes

Cartesian coordinates for DFT-optimized structures of **21**–**29** and (PP^NH2^P)­Co­(CO)­(H) (XYZ)
Spectroscopic characterization data for **5**–**20** and **30**–**46**, cyclic voltammetry data for **3** and **13**–**20**, EPR data for **3** and **13**–**20**, experimental procedures and detailed kinetic analysis of styrene and α-methylstyrene hydroboration reactions catalyzed by **3** and **13**–**20**, X-ray crystallography details for **13**-**20**, and additional details related to DFT calculations (PDF)
