# Supplementary material for: Synthesis and Characterization of a Series of (PPRP)Co Complexes and Comparison of their Electronic Properties and Catalytic Activities
Source: Organometallics. 2026 Jun 19;45(13):1554–70. doi: 10.1021/acs.organomet.6c00131 (PMC13371084; doi:10.1021/acs.organomet.6c00131)
Supplement: Supplementary file 2 [file om6c00131_si_002.pdf]

SUPPORTING INFORMATION

Synthesis and Characterization of a Series of $(PP^{R}P)Co$ Complexes and Comparison of their Electronic Properties and Catalytic Activities

Maria C. Seith, Matthew C. Fitzsimmons, Hanan A. Muhammad, Chris A. Nieto, Curtis E. Moore, Luke C. Lewis, and Christine M. Thomas*

Department of Chemistry and Biochemistry, The Ohio State University, 100 W. 18th Ave, Columbus OH 43210, USA

thomasc@chemistry.ohio-state.edu

Table of Contents	
**NMR Characterization Data for 5-20 and 30-46**	**S9-S110**
**Figure S1**: ^{1}H NMR spectrum of $PP^{NEt2}P$ (**5**).	S9
**Figure S2**: $^{31}P\{^{1}H\}$ NMR spectrum of $PP^{NEt2}P$ (**5**).	S10
**Figure S3**: $^{13}C\{^{1}H\}$ NMR spectrum of $PP^{NEt2}P$ (**5**).	S11
**Figure S4**: ^{1}H NMR spectrum of $(PP^{NiPr2}P)$ (**6**).	S12
**Figure S5**: $^{31}P\{^{1}H\}$ NMR spectrum of $(PP^{NiPr2}P)$ (**6**).	S13
**Figure S6:** $^{13}C\{^{1}H\}$ NMR spectrum of $(PP^{NiPr2}P)$ (**6**).	S14
**Figure S7**: ^{1}H NMR spectrum of $(PP^{OEt}P)$ (**7**).	S15
**Figure S8**: $^{31}P\{^{1}H\}$ NMR (spectrum of $(PP^{OEt}P)$ (**7**).	S16
**Figure S9:** $^{13}C\{^{1}H\}$ NMR spectrum of $(PP^{OEt}P)$ (**7**).	S17
**Figure S10:** ^{1}H NMR spectrum of $(PP^{OiPr}P)$ (**8**).	S18
**Figure S11**: $^{31}P\{^{1}H\}$ NMR spectrum of $(PP^{OiPr}P)$ (**8**).	S19
**Figure S12**: $^{13}C\{^{1}H\}$ NMR spectrum of $(PP^{OiPr}P)$ (**8**).	S20
**Figure S13:** ^{1}H NMR spectrum of $(PP^{OCH2CF3}P)$ (**9**).	S21
**Figure S14**: $^{31}P\{^{1}H\}$ NMR spectrum of $(PP^{OCH2CF3}P)$ (**9**).	S22
**Figure S15**: ^{19}F NMR spectrum of $(PP^{OCH2CF3}P)$ (**9**).	S23
**Figure S16**: $^{13}C\{^{1}H\}$ NMR spectrum of $(PP^{OCH2CF3}P)$ (**9**).	S24
**Figure S17:** ^{1}H NMR spectrum of $(PP^{OCH(CF3)2}P)$ (**10**).	S25
**Figure S18**: $^{31}P\{^{1}H\}$ NMR spectrum of $(PP^{OCH(CF3)2}P)$ (**10**).	S26
**Figure S19**: ^{19}F NMR spectrum of $(PP^{OCH(CF3)2}P)$ (**10**).	S27
**Figure S20:** $^{13}C\{^{1}H\}$ NMR spectrum of $(PP^{OCH(CF3)2}P)$ (**10**).	S28
**Figure S21:** ^{1}H NMR spectrum of $(PP^{menthoxide}P)$ (**11**).	S29
**Figure S22**: $^{31}P\{^{1}H\}$ NMR spectrum of $(PP^{menthoxide}P)$ (**11**).	S30
**Figure S23:** $^{13}C\{^{1}H\}$ NMR spectrum of $(PP^{menthoxide}P)$ (**11**).	S31
**Figure S24:** ^{1}H NMR spectrum of $(PP^{Me}P)$ (**12**).	S32
**Figure S25**: $^{31}P\{^{1}H\}$ NMR spectrum of $(PP^{Me}P)$ (**12**).	S33
**Figure S26:** $^{13}C\{^{1}H\}$ NMR spectrum of $(PP^{Me}P)$ (**12**).	S34

**Figure S27:** ^{1}H NMR spectrum of (PP^{NEt2}P)CoI$_2$ (**13**).	S35
**Figure S28:** ^{1}H NMR spectrum of (PP^{NiPr2}P)CoI$_2$ (**14**).	S36
**Figure S29:** ^{1}H NMR spectrum of (PP^{OEt}P)CoI$_2$ (**15**).	S37
**Figure S30:** ^{1}H NMR spectrum of (PP^{OiPr}P)CoI$_2$ (**16**).	S38
**Figure S31:** ^{1}H NMR spectrum of (PP^{OCH2CF3}P)CoI$_2$ (**17**).	S39
**Figure S32:** ^{1}H NMR spectrum of (PP$^{OCH(CF3)2}$P)CoI$_2$ (**18**).	S40
**Figure S33:** ^{1}H NMR spectrum of (PP^{menthoxide}P)CoI$_2$ (**19**).	S41
**Figure S34:** ^{1}H NMR spectrum of (PP^{Me}P)CoI$_2$ (**20**).	S42
**Figure S35**: ^{1}H NMR spectrum of (FP^{Cl}F) (**30**).	S43
**Figure S36**: ^{31}P{^{1}H} NMR spectrum of (FP^{Cl}F) (**30**).	S44
**Figure S37**: ^{19}F NMR spectrum of (FP^{Cl}F) (**30**).	S45
**Figure S38**: ^{13}C{^{1}H} NMR spectrum of (FP^{Cl}F) (**30**).	S46
**Figure S39**: ^{1}H NMR spectrum of (FP^{NEt2}F) (**31**).	S47
**Figure S40**: ^{31}P{^{1}H} NMR spectrum of (FP^{NEt2}F) (**31**).	S48
**Figure S41**: ^{19}F NMR spectrum of (FP^{NEt2}F) (**31**).	S49
**Figure S42**: ^{13}C{^{1}H} NMR spectrum of (FP^{NEt2}F) (**31**).	S50
**Figure S43**: ^{1}H NMR spectrum of (FP$^{N\textit{i}Pr2}$F) (**32**).	S51
**Figure S44**: ^{31}P{^{1}H} NMR spectrum of (FP$^{N\textit{i}Pr2}$F) (**32**).	S52
**Figure S45**: ^{19}F NMR spectrum of (FP$^{N\textit{i}Pr2}$F) (**32**).	S53
**Figure S46**: ^{13}C{^{1}H} NMR spectrum of (FP$^{N\textit{i}Pr2}$F) (**32**).	S54
**Figure S47**: ^{1}H NMR spectrum of (FP^{OEt}F) (**33**).	S55
**Figure S48**: ^{31}P{^{1}H} NMR spectrum of (FP^{OEt}F) (**33**).	S56
**Figure S49**: ^{19}F NMR spectrum of (FP^{OEt}F) (**33**).	S57
**Figure S50**: ^{13}C{^{1}H} NMR spectrum of (FP^{OEt}F) (**33**).	S58
**Figure S51**: ^{1}H NMR spectrum of (FP$^{O\textit{i}Pr}$F) (**34**).	S59
**Figure S52**: ^{31}P{^{1}H} NMR spectrum of (FP$^{O\textit{i}Pr}$F) (**34**).	S60
**Figure S53**: ^{19}F NMR spectrum of (FP$^{O\textit{i}Pr}$F) (**34**).	S61
**Figure S54**: ^{13}C{^{1}H} NMR spectrum of (FP$^{O\textit{i}Pr}$F) (**34**).	S62
**Figure S55**: ^{1}H NMR spectrum of (FP^{OCH2CF3}F) (**35**).	S63
**Figure S56**: ^{31}P{^{1}H} NMR spectrum of (FP^{OCH2CF3}F) (**35**).	S64
**Figure S57**: ^{19}F NMR spectrum of (FP^{OCH2CF3}F) (**35**).	S65
**Figure S58**: ^{13}C{^{1}H} NMR spectrum of (FP^{OCH2CF3}F) (**35**).	S66
**Figure S59**: ^{1}H NMR spectrum of (FP$^{OCH(CF3)2}$F) (**36**).	S67
**Figure S60**: ^{31}P{^{1}H} NMR spectrum of (FP$^{OCH(CF3)2}$F) (**36**).	S68
**Figure S61**: ^{19}F NMR spectrum of (FP$^{OCH(CF3)2}$F) (**36**).	S69
**Figure S62**: ^{13}C{^{1}H} NMR spectrum of (FP$^{OCH(CF3)2}$F) (**36**).	S70
**Figure S63**: ^{1}H NMR spectrum of (FP^{Me}F) (**37**).	S71
**Figure S64**: ^{31}P{^{1}H} NMR spectrum of (FP^{Me}F) (**37**).	S72
**Figure S65**: ^{19}F NMR spectrum of (FP^{Me}F) (**37**).	S73
**Figure S66**: ^{13}C{^{1}H} NMR spectrum of (FP^{Me}F) (**37**).	S74
**Figure S67**: ^{1}H NMR spectrum of (FP^{CF3}F) (**38**).	S75
**Figure S68**: ^{31}P{^{1}H} NMR spectrum of (FP^{CF3}F) (**38**).	S76
**Figure S69**: ^{19}F NMR spectrum of (FP^{CF3}F) (**38**).	S77
**Figure S70**: ^{13}C{^{1}H} NMR spectrum of (FP^{CF3}F) (**38**).	S78
**Figure S71**: ^{1}H NMR spectrum of (F(Se=)P^{NEt2}F) (**39**).	S79

**Figure S72**: $^{31}P\{^1H\}$ NMR spectrum of (F(Se=)P^{NEt2}F) (**39**).	S80
**Figure S73**: ^{19}F NMR spectrum of (F(Se=)P^{NEt2}F) (**39**).	S81
**Figure S74**: $^{13}C\{^1H\}$ NMR spectrum of (F(Se=)P^{NEt2}F) (**39**).	S82
**Figure S75**: ^1H NMR spectrum of (F(Se=)$P^{N\textit{i}Pr2}$F) (**40**).	S83
**Figure S76**: $^{31}P\{^1H\}$ NMR spectrum of (F(Se=)$P^{N\textit{i}Pr2}$F) (**40**).	S84
**Figure S77**: ^{19}F NMR spectrum of (F(Se=)$P^{N\textit{i}Pr2}$F) (**40**).	S85
**Figure S78**: $^{13}C\{^1H\}$ NMR spectrum of (F(Se=)$P^{N\textit{i}Pr2}$F) (**40**).	S86
**Figure S79**: ^1H NMR spectrum of (F(Se=)P^{OEt}F) (**41**).	S87
**Figure S80**: $^{31}P\{^1H\}$ NMR spectrum of (F(Se=)P^{OEt}F) (**41**).	S88
**Figure S81**: ^{19}F NMR spectrum of (F(Se=)P^{OEt}F) (**41**).	S89
**Figure S82**: $^{13}C\{^1H\}$ NMR spectrum of (F(Se=)P^{OEt}F) (**41**).	S90
**Figure S83**: ^1H NMR spectrum of (F(Se=)$P^{O\textit{i}Pr}$F) (**42**).	S91
**Figure S84**: $^{31}P\{^1H\}$ NMR spectrum of (F(Se=)$P^{O\textit{i}Pr}$F) (**42**).	S92
**Figure S85**: ^{19}F NMR spectrum of (F(Se=)$P^{O\textit{i}Pr}$F) (**42**).	S93
**Figure S86**: $^{13}C\{^1H\}$ NMR spectrum of (F(Se=)$P^{O\textit{i}Pr}$F) (**42**).	S94
**Figure S87**: ^1H NMR spectrum of (F(Se=)$P^{OCH2CF3}$F) (**43**).	S95
**Figure S88**: $^{31}P\{^1H\}$ NMR spectrum of (F(Se=)$P^{OCH2CF3}$F) (**43**).	S96
**Figure S89**: ^{19}F NMR spectrum of (F(Se=)$P^{OCH2CF3}$F) (**43**).	S97
**Figure S90**: $^{13}C\{^1H\}$ NMR spectrum of (F(Se=)$P^{OCH2CF3}$F) (**43**).	S98
**Figure S91**: ^1H NMR spectrum of (F(Se=)$P^{OCH(CF3)2}$F) (**44**).	S99
**Figure S92**: $^{31}P\{^1H\}$ NMR spectrum of (F(Se=)$P^{OCH(CF3)2}$F) (**44**).	S100
**Figure S93**: ^{19}F NMR spectrum of (F(Se=)$P^{OCH(CF3)2}$F) (**44**).	S101
**Figure S94**: $^{13}C\{^1H\}$ NMR spectrum of (F(Se=)$P^{OCH(CF3)2}$F) (**44**).	S102
**Figure S95**: ^1H NMR spectrum of (F(Se=)P^{Me}F) (**45**).	S103
**Figure S96**: $^{31}P\{^1H\}$ NMR spectrum of (F(Se=)P^{Me}F) (**45**).	S104
**Figure S97**: ^{19}F NMR spectrum of (F(Se=)P^{Me}F) (**45**).	S105
**Figure S98**: $^{13}C\{^1H\}$ NMR spectrum of (F(Se=)P^{Me}F) (**45**).	S106
**Figure S99**: ^1H NMR spectrum of (F(Se=)P^{CF3}F) (**46**).	S107
**Figure S100**: $^{31}P\{^1H\}$ NMR spectrum of (F(Se=)P^{CF3}F) (**46**).	S108
**Figure S101**: ^{19}F NMR spectrum of (F(Se=)P^{CF3}F) (**46**).	S109
**Figure S102**: $^{13}C\{^1H\}$ NMR spectrum of (F(Se=)P^{CF3}F) (**46**).	S110
**Cyclic Voltammetry Data**	**S111-S119**
**Figure S103**: Cyclic voltammograms of ($PP^{NEt2}P$)CoI_2 (**13**).	S111
**Figure S104**: Cyclic voltammograms of ($PP^{NiPr2}P$)CoI_2 (**14**).	S112
**Figure S105**: Cyclic voltammogram*s* of ($PP^{OEt}P$)CoI_2 (**15**).	S113
**Figure S106**: Cyclic voltammogram*s* of ($PP^{O\textit{i}Pr}P$)CoI_2 (**16**).	S114
**Figure S107**: Cyclic voltammogram*s* of ($PP^{OCH2CF3}P$)CoI_2 (**17**).	S115
**Figure S108**: Cyclic voltammogram*s* of ($PP^{OCH(CF3)2}P$)CoI_2 (**18**).	S116
**Figure S109**: Cyclic voltammogram*s* of ($PP^{menthoxide}P$)CoI_2 (**19**).	S117
**Figure S110**: Cyclic voltammogram*s* of ($PP^{Me}P$)CoI_2 (**20**).	S118
**Figure S111**: Cyclic voltammogram*s* of ($PP^{CF3}P$)CoI_2 (**3**).	S119
**EPR Data**	**S120-S131**
**Table S1**: g_1, g_2, g_3, and g_{iso} values for compounds **3** and **13-20** obtained from simulation of low temperature (40 K) EPR spectra.	S120
**Figure S112**. X-band EPR spectra of **14**, **16**, **18**, and **20** collected at 40 K.	S121

**Figure S113**. Experimental and simulated X-band EPR spectra of **13** and **14** collected at 40 K.	S122
**Figure S114**. Experimental and simulated X-band EPR spectra of **15**, **16**, and **19** collected at 40 K.	S123
**Figure S115**. Experimental and simulated X-band EPR spectra of **17** and **18** collected at 40 K.	S124
**Figure S116**. Experimental and simulated X-band EPR spectra of **3** and **20** collected at 40 K.	S125
**Table S2**: g_{iso} and A_{iso} (^{59}Co) values for compounds **3**, **13**, **15-18**, and **20** obtained from simulation of room temperature (298 K) EPR spectra.	S126
**Figure S117**. X-band EPR spectra of **13**, **16**, **18**, and **20** collected at 298 K.	S127
**Figure S118**. Experimental and simulated X-band EPR spectra of **13** collected at 298 K.	S128
**Figure S119**. Experimental and simulated X-band EPR spectra of **15** and **16** collected at 298 K.	S129
**Figure S120**. Experimental and simulated X-band EPR spectra of **17** and **18** collected at 298 K.	S130
**Figure S121**. Experimental and simulated X-band EPR spectra of **20** collected at 298 K.	S131
**Kinetic Studies: Hydroboration of Styrene**	**S132-S153**
Hydroboration of styrene with 1.0 mol % ($PP^{NEt2}P$)CoI_2 (**13**), trial one.	S132
**Figure S122**: Plotted timepoints and first order kinetics analysis of formation of linearly hydroborated styrene (red) and consumption of styrene versus time for 1.0 mol % loading of ($PP^{NEt2}P$)CoI_2 (**13**).	S132
Hydroboration of styrene with 1.0 mol % ($PP^{NEt2}P$)CoI_2 (**13**), trial two.	S133
**Figure 123**: Plotted timepoints and first order kinetics analysis of formation of linearly hydroborated styrene (red) and consumption of styrene versus time for 1.0 mol % loading of ($PP^{NEt2}P$)CoI_2 (**13**).	S133
Hydroboration of styrene with 1.0 mol % ($PP^{N\textit{i}Pr2}P$)CoI_2 (**14**), trial one.	S134
**Figure S124**: Plotted timepoints and first order kinetics analysis of formation of linearly hydroborated styrene and consumption of styrene versus time for 1.0 mol % loading of ($PP^{N\textit{i}Pr2}P$)CoI_2 (**14**).	S134
Hydroboration of styrene with 1.0 mol % ($PP^{N\textit{i}Pr2}P$)CoI_2 (**14**), trial two.	S135
**Figure S125**: Plotted timepoints and first order kinetics analysis of formation of linearly hydroborated styrene and consumption of styrene versus time for 1.0 mol % loading of ($PP^{N\textit{i}Pr2}P$)CoI_2 (**14**).	S135
Hydroboration of styrene with 1.0 mol % ($PP^{OEt}P$)CoI_2 (**15**), trial one.	S136
**Figure 126**: Plotted timepoints and first order kinetics analysis of formation of linearly hydroborated styrene and consumption of styrene versus time for 1.0 mol % loading of ($PP^{OEt}P$)CoI_2 (**15**).	S136
Hydroboration of styrene with 1.0 mol % ($PP^{OEt}P$)CoI_2 (**15**), trial two.	S137
**Figure S127**: Plotted timepoints and first order kinetics analysis of formation of linearly hydroborated styrene and consumption of styrene versus time for 1.0 mol % loading of ($PP^{OEt}P$)CoI_2 (**15**).	S137
Hydroboration of styrene with 1.0 mol % ($PP^{O\textit{i}Pr}P$)CoI_2 (**16**), trial one.	S138

**Figure S128**: Plotted timepoints and first order kinetics analysis of formation of linearly hydroborated styrene and consumption of styrene versus time for 1.0 mol % loading of $(PP^{O\textit{i}Pr}P)CoI_2$ (**16**).	S138
Hydroboration of styrene with 1.0 mol % $(PP^{O\textit{i}Pr}P)CoI_2$ (**16**), trial two.	S139
**Figure S129**: Plotted timepoints and first order kinetics analysis of formation of linearly hydroborated styrene and consumption of styrene versus time for 1.0 mol % loading of $(PP^{O\textit{i}Pr}P)CoI_2$ (**16**).	S139
Hydroboration of styrene with 1.0 mol % $(PP^{OCH2CF3}P)CoI_2$ (**17**), trial one.	S140
**Figure S130.** GC-FID of reaction mixture of catalysis carried out with 1.0 mol % **17** after 5 minutes.	S140
Hydroboration of styrene with 1.0 mol % $(PP^{OCH2CF3}P)CoI_2$ (**17**), trial two.	S141
**Figure S131.** GC-FID of reaction mixture of catalysis carried out with 1.0 mol % **17** after 5 minutes.	S141
Hydroboration of styrene with 1.0 mol % $(PP^{OCH(CF3)2}P)CoI_2$ (**18**), trial one.	S142
**Figure S132**: Plotted timepoints and first order kinetics analysis of formation of linearly hydroborated styrene and consumption of styrene versus time for 1.0 mol % loading of $(PP^{OCH(CF3)2}P)CoI_2$ (**18**).	S142
Hydroboration of styrene with 1.0 mol % $(PP^{OCH(CF3)2}P)CoI_2$ (**18**), trial two.	S143
**Figure S133**: Plotted timepoints and first order kinetics analysis of formation of linearly hydroborated styrene and consumption of styrene versus time for 1.0 mol % loading of $(PP^{OCH(CF3)2}P)CoI_2$ (**18**).	S143
Hydroboration of styrene with 1.0 mol % $(PP^{nenthoxide}P)CoI_2$ (**19**), trial one.	S144
**Figure S134**: Plotted timepoints and first order kinetics analysis of formation of linearly hydroborated styrene (red) and consumption of styrene versus time for 1.0 mol % loading of $(PP^{menthoxide}P)CoI_2$ (**19**).	S144
Hydroboration of styrene with 1.0 mol % $(PP^{menthoxide}P)CoI_2$ (**19**), trial two.	S145
**Figure S135**: Plotted timepoints and first order kinetics analysis of formation of linearly hydroborated styrene and consumption of styrene versus time for 1.0 mol % loading of $(PP^{menthoxide}P)CoI_2$ (**19**).	S145
Hydroboration of styrene with 1.0 mol % $(PP^{Me}P)CoI_2$ (**20**), trial one.	S146
**Figure S136**: Plotted timepoints and first order kinetics analysis of formation of linearly hydroborated styrene and consumption of styrene versus time for 1.0 mol % loading of $(PP^{Me}P)CoI_2$ (**20**).	S146
Hydroboration of styrene with 1.0 mol % $(PP^{Me}P)CoI_2$ (**20**), trial two.	S147
**Figure S137**: Plotted timepoints and first order kinetics analysis of formation of linearly hydroborated styrene and consumption of styrene versus time for 1.0 mol % loading of $(PP^{Me}P)CoI_2$ (**20**).	S147
Hydroboration of styrene with 1.0 mol % $(PP^{CF3}P)CoI_2$ (**3**), trial one.	S148
**Figure S138**: Plotted timepoints and first order kinetics analysis of formation of linearly hydroborated styrene and consumption of styrene versus time for 1.0 mol % loading of $(PP^{CF3}P)CoI_2$ (**3**).	S148
Hydroboration of styrene with 1.0 mol % $(PP^{CF3}P)CoI_2$ (**3**), trial two.	S149
**Figure S139**: Plotted timepoints and first order kinetics analysis of formation of linearly hydroborated styrene and consumption of styrene versus time for 1.0 mol % loading of $(PP^{CF3}P)CoI_2$ (**3**).	S149
Hydroboration of styrene with 0.1 mol % $(PP^{OEt}P)CoI_2$ (**15**), trial one.	S150

**Figure 140**: Plotted timepoints and first order kinetics analysis of formation of linearly hydroborated styrene and consumption of styrene versus time for 0.1 mol % loading of $(PP^{OEt}P)CoI_2$ (**15**).	S150
Hydroboration of styrene with 0.1 mol % $(PP^{OEt}P)CoI_2$ (**15**), trial two.	S151
**Figure 141**: Plotted timepoints and first order kinetics analysis of formation of linearly hydroborated styrene and consumption of styrene versus time for 0.1 mol % loading of $(PP^{OEt}P)CoI_2$ (**15**).	S151
Hydroboration of styrene with 0.1 mol % $(PP^{OCH2CF3}P)CoI_2$ (**17**), trial one.	S152
**Figure 142**: Plotted timepoints and first order kinetics analysis of formation of linearly hydroborated styrene and consumption of styrene versus time for 0.1 mol % loading of $(PP^{OCH2CF3}P)CoI_2$ (**17**).	S152
Hydroboration of styrene with 0.1 mol % $(PP^{OCH2CF3}P)CoI_2$ (**17**), trial two.	S153
**Figure 143**: Plotted timepoints and first order kinetics analysis of formation of linearly hydroborated styrene and consumption of styrene versus time for 0.1 mol % loading of $(PP^{OCH2CF3}P)CoI_2$ (**17**).	S153
**Kinetic Studies: Hydroboration of α-methylstyrene**	**S154-S171**
Hydroboration of α-methylstyrene with 1.0 mol % $(PP^{NEt2}P)CoI_2$ (**13**), trial one.	S154
**Figure 144**: Plotted timepoints and first order kinetics analysis of formation of linearly hydroborated *α*-methylstyrene and consumption of *α*-methylstyrene versus time for 1.0 mol % loading of $(PP^{NEt2}P)CoI_2$ (**13**).	S154
Hydroboration of α-methylstyrene with 1.0 mol % $(PP^{NEt2}P)CoI_2$ (**13**), trial two.	S155
**Figure 145**: Plotted timepoints and first order kinetics analysis of formation of linearly hydroborated *α*-methylstyrene and consumption of *α*-methylstyrene versus time for 1.0 mol % loading of $(PP^{NEt2}P)CoI_2$ (**13**).	S155
Hydroboration of α-methylstyrene with 1.0 mol % $(PP^{NiPr2}P)CoI_2$ (**14**), trial one.	S156
**Figure 146**: Plotted timepoints and first order kinetics analysis of formation of linearly hydroborated α-methylstyrene and consumption of α-methylstyrene versus time for 1.0 mol % loading of $(PP^{NiPr2}P)CoI_2$ (**14**).	S156
Hydroboration of α-methylstyrene with 1.0 mol % $(PP^{NiPr2}P)CoI_2$ (**14**), trial two.	S157
**Figure 147**: Plotted timepoints and first order kinetics analysis of formation of linearly hydroborated *α*-methylstyrene and consumption of *α*-methylstyrene versus time for 1.0 mol % loading of $(PP^{NiPr2}P)CoI_2$ (**14**).	S157
Hydroboration of α-methylstyrene with 1.0 mol % $(PP^{OEt}P)CoI_2$ (**15**), trial one.	S158
**Figure 148**: Plotted timepoints and first order kinetics analysis of formation of linearly hydroborated α-methylstyrene and consumption of α-methylstyrene versus time for 1.0 mol % loading of $(PP^{OEt}P)CoI_2$ (**15**).	S158
Hydroboration of α-methylstyrene with 1.0 mol % $(PP^{OEt}P)CoI_2$ (**15**), trial two.	S159
**Figure 149**: Plotted timepoints and first order kinetics analysis of formation of linearly hydroborated *α*-methylstyrene and consumption of *α*-methylstyrene versus time for 1.0 mol % loading of $(PP^{OEt}P)CoI_2$ (**15**).	S159
Hydroboration of α-methylstyrene with 1.0 mol % $(PP^{OiPr}P)CoI_2$ (**16**), trial one.	S160
**Figure 150**: Plotted timepoints and first order kinetics analysis of formation of linearly hydroborated α-methylstyrene and consumption of α-methylstyrene versus time for 1.0 mol % loading of $(PP^{OiPr}P)CoI_2$ (**16**).	S160
Hydroboration of α-methylstyrene with 1.0 mol % $(PP^{OiPr}P)CoI_2$ (**16**), trial two.	S161

**Figure 151**: Plotted timepoints and first order kinetics analysis of formation of linearly hydroborated α-methylstyrene and consumption of α-methylstyrene versus time for 1.0 mol % loading of $(PP^{O\textit{i}Pr}P)CoI_2$ (**16**).	S161
Hydroboration of α-methylstyrene with 1.0 mol % $(PP^{OCH2CF3}P)CoI_2$ (**17**), trial one.	S162
**Figure 152**: Plotted timepoints and first order kinetics analysis of formation of linearly hydroborated α-methylstyrene and consumption of α-methylstyrene versus time for 1.0 mol % loading of $(PP^{OCH2CF3}P)CoI_2$ (**17**).	S162
Hydroboration of α-methylstyrene with 1.0 mol % $(PP^{OCH2CF3}P)CoI_2$ (**17**), trial two.	S163
**Figure 153**: Plotted timepoints and first order kinetics analysis of formation of linearly hydroborated *α*-methylstyrene and consumption of *α*-methylstyrene versus time for 1.0 mol % loading of $(PP^{OCH2CF3}P)CoI_2$ (**17**).	S163
Hydroboration of α-methylstyrene with 1.0 mol % $(PP^{OCH(CF3)2}P)CoI_2$ (**18**), trial one.	S164
**Figure 154**: Plotted timepoints and first order kinetics analysis of formation of linearly hydroborated α-methylstyrene and consumption of α-methylstyrene versus time for 1.0 mol % loading of $(PP^{OCH(CF3)2}P)CoI_2$ (**18**).	S164
Hydroboration of α-methylstyrene with 1.0 mol % $(PP^{OCH(CF3)2}P)CoI_2$ (**18**), trial two.	S165
**Figure 155**: Plotted timepoints and first order kinetics analysis of formation of linearly hydroborated *α*-methylstyrene and consumption of *α*-methylstyrene versus time for 1.0 mol % loading of $(PP^{OCH(CF3)2}P)CoI_2$ (**18**).	S165
Hydroboration of α-methylstyrene with 1.0 mol % $(PP^{menthoxide}P)CoI_2$ (**19**), trial one.	S166
**Figure 156**: Plotted timepoints and first order kinetics analysis of formation of linearly hydroborated α-methylstyrene and consumption of α-methylstyrene versus time for 1.0 mol % loading of $(PP^{menthoxide}P)CoI_2$ (**19**).	S166
Hydroboration of α-methylstyrene with 1.0 mol % $(PP^{menthoxide}P)CoI_2$ (**19**), trial two.	S167
**Figure 157**: Plotted timepoints and first order kinetics analysis of formation of linearly hydroborated α-methylstyrene and consumption of α-methylstyrene versus time for 1.0 mol % loading of $(PP^{menthoxide}P)CoI_2$ (**19**).	S167
Hydroboration of α-methylstyrene with 1.0 mol % $(PP^{Me}P)CoI_2$ (**20**), trial one.	S168
**Figure 158**: Plotted timepoints and first order kinetics analysis of formation of linearly hydroborated α-methylstyrene and consumption of α-methylstyrene versus time for 1.0 mol % loading of $(PP^{Me}P)CoI_2$ (**20**).	S168
Hydroboration of α-methylstyrene with 1.0 mol % $(PP^{Me}P)CoI_2$ (**20**), trial two.	S169
**Figure 159**: Plotted timepoints and first order kinetics analysis of formation of linearly hydroborated *α*-methylstyrene and consumption of *α*-methylstyrene versus time for 1.0 mol % loading of $(PP^{Me}P)CoI_2$ (**20**).	S169
Hydroboration of α-methylstyrene with 1.0 mol % $(PP^{CF3}P)CoI_2$ (**3**), trial one.	S170
**Figure 160**: Plotted timepoints and first order kinetics analysis of formation of linearly hydroborated *α*-methylstyrene and consumption of *α*-methylstyrene versus time for 1.0 mol % loading of $(PP^{CF3}P)CoI_2$ (**3**).	S170
Hydroboration of α-methylstyrene with 1.0 mol % $(PP^{CF3}P)CoI_2$ (**3**), trial two.	S171

**Figure 161**: Plotted timepoints and first order kinetics analysis of formation of linearly hydroborated α-methylstyrene and consumption of α-methylstyrene versus time for 1.0 mol % loading of $(PP^{CF3}P)CoI_2$ (**3**).	S171
**X-Ray Crystallography Data Collection and Refinement Details**	**S172-S190**
**Figure S162**. Fully labelled displacement ellipsoid representation of $(PP^{NEt2}P)CoI_2$ (**13**).	S172
Data Collection and Refinement Details for **13**.	S172
**Figure S163**. Fully labelled displacement ellipsoid representation of $(PP^{N\textit{i}Pr2}P)CoI_2$ (**14**).	S174
Data Collection and Refinement Details for **14**.	S174
**Figure S164.** Fully labelled displacement ellipsoid representation of $(PP^{OEt}P)CoI_2$ (**15**).	S176
Data Collection and Refinement Details for **15**.	S176
**Figure S165**. Fully labelled displacement ellipsoid representation of $(PP^{O\textit{i}Pr}P)CoI_2$ (**16**).	S178
Data Collection and Refinement Details for **16**.	S178
**Figure S166**. Fully labelled displacement ellipsoid representation of $(PP^{OCH2CF3}P)CoI_2$ (**17**).	S180
Data Collection and Refinement Details for **17**.	S180
**Figure S167**. Fully labelled displacement ellipsoid representation of $(PP^{OCH(CF3)2}P)CoI_2$ (**18**).	S182
Data Collection and Refinement Details for **18**.	S182
**Figure S168**. Fully labelled displacement ellipsoid representation of $(PP^{menthoxide}P)CoI_2$ (**19**).	S184
Data Collection and Refinement Details for **19**.	S184
**Figure S169**. Fully labelled displacement ellipsoid representation of $(PP^{Me}P)CoI_2$ (**20**).	S186
Data Collection and Refinement Details for **20**.	S186
**Table S3.** Crystal data and structure refinement details for **13**•1.5(CH_2Cl_2),0.25(Et_2O), **14**, and **15**•2.5(CH_2Cl_2).	S188
**Table S4.** Crystal data and structure refinement details for **16**•(CH_2Cl_2), **17**•2(CH_2Cl_2), and **18**•(CH_2Cl_2).	S189
**Table S5.** Crystal data and structure refinement details for **19**•2.5(C_6H_6) and **20**•2.5(THF).	S190
**DFT Calculations of 21-29**	S190-192
**Figure S170**. DFT-optimized geometries of $(PP^{R}P)Co(H)(CO)$ compounds **21-29**.	S191
**Table S6**. Selected natural charges and Wiberg bond indices (WBIs) calculated for **21-29**.	192

NMR Characterization Data for 5-46

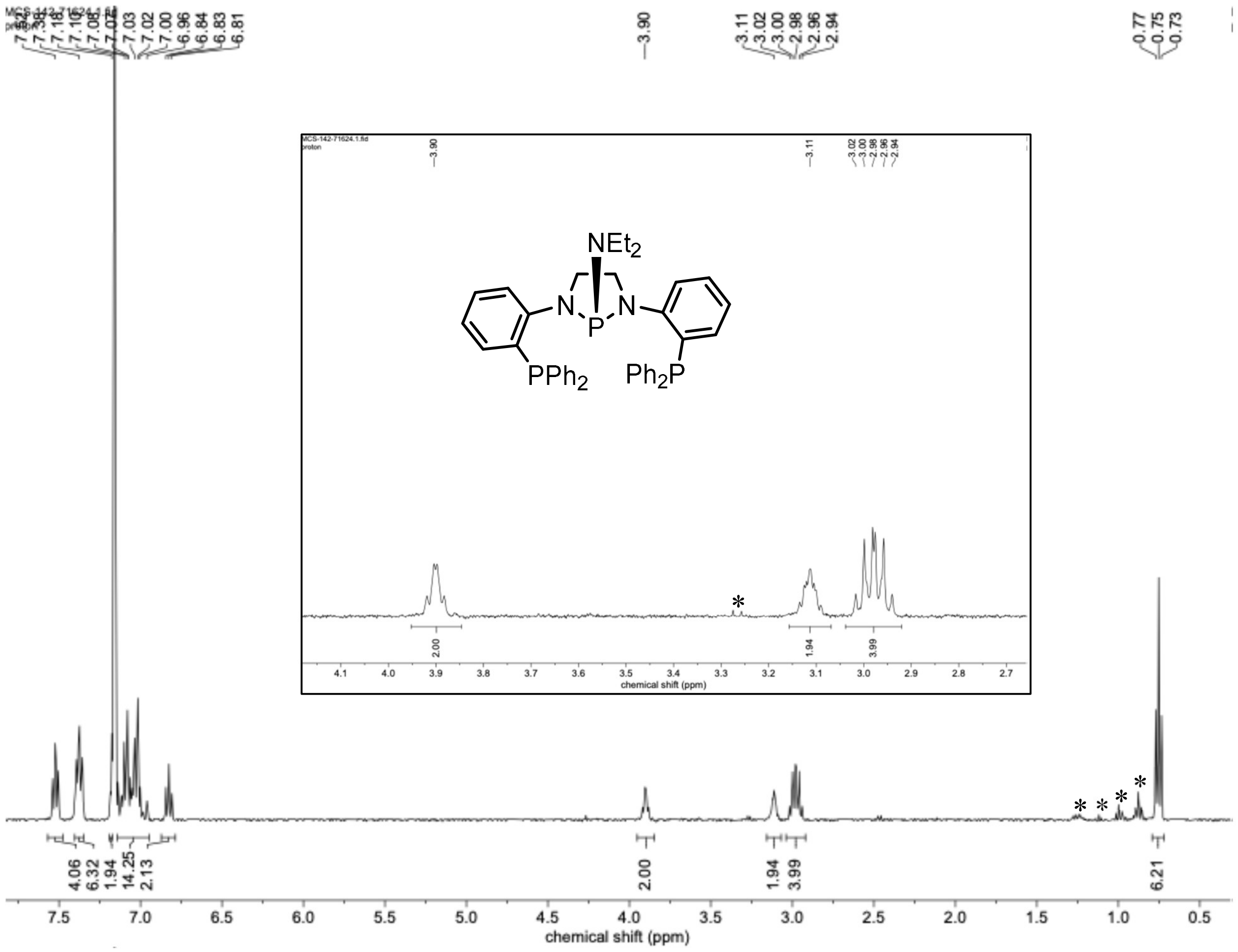

Figure S1: ^{1}H NMR (C_6D_6, 400 MHz) spectrum of ($PP^{NEt2}P$) (**5**). Residual solvent denoted with an asterisk (*).

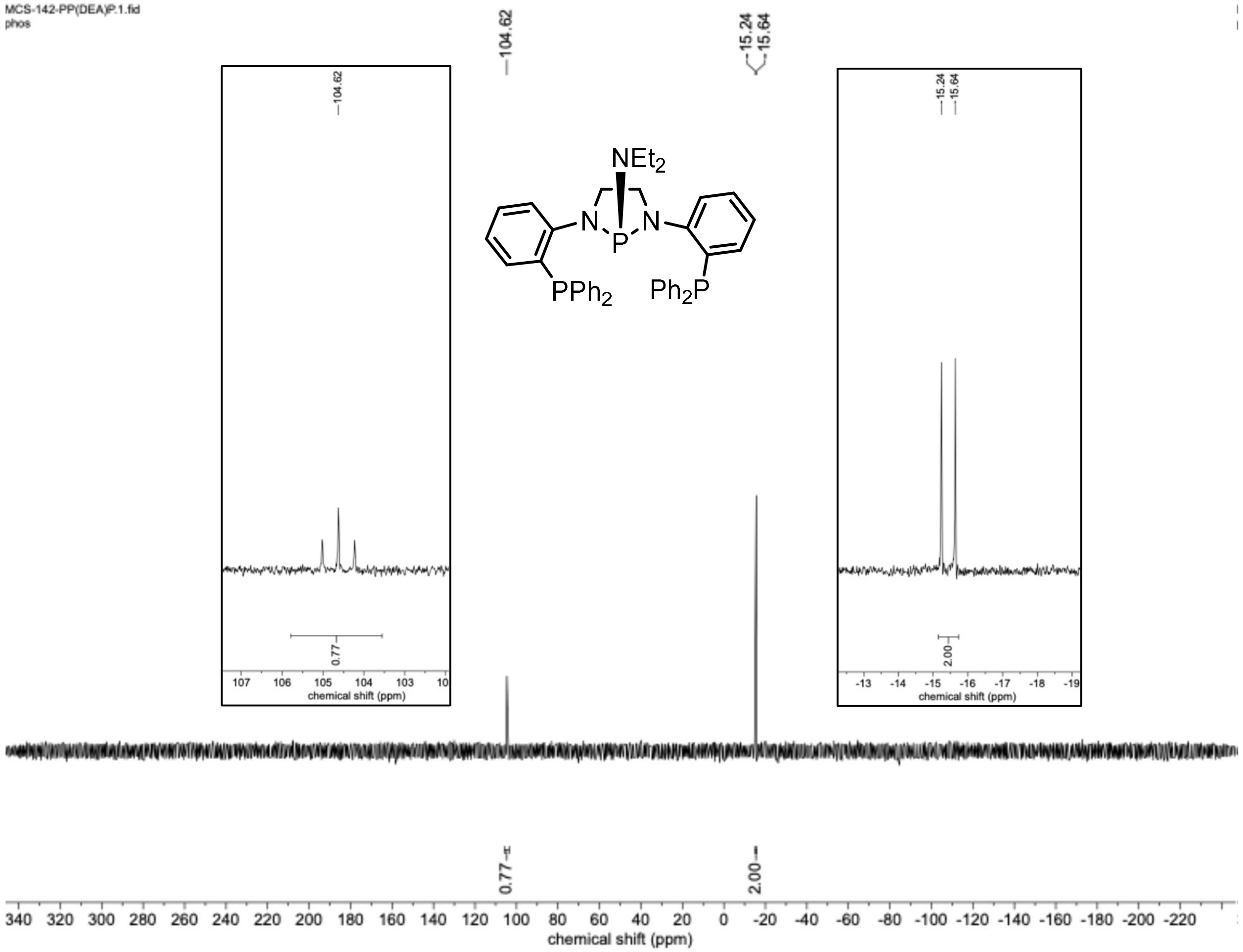

Figure S2: $^{31}P\{^{1}H\}$ NMR (C_6D_6, 162 MHz) spectrum of ($PP^{NEt2}P$) (**5**).

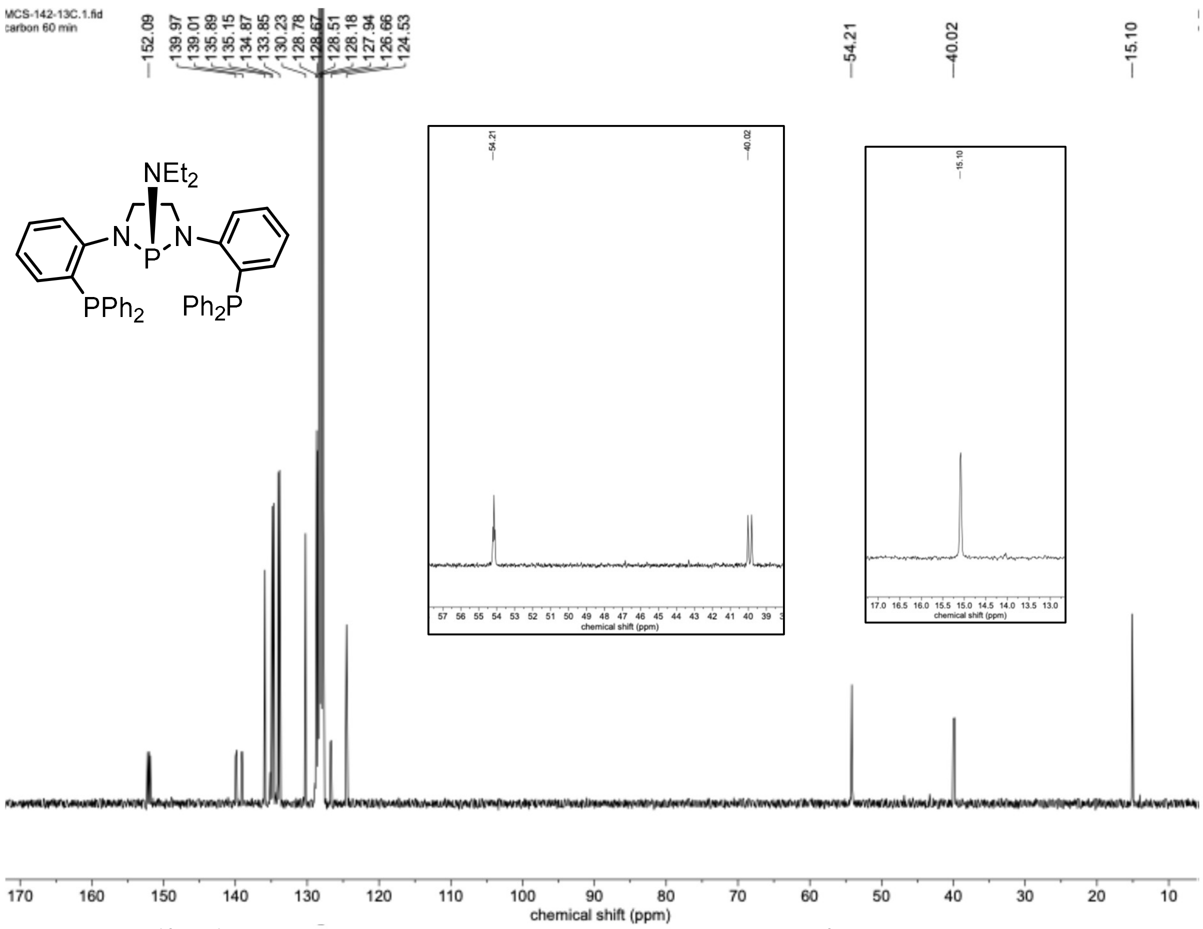

Figure S3: $^{13}C\{^1H\}$ NMR (C_6D_6, 151 MHz) spectrum of ($PP^{NEt2}P$) (**5**).

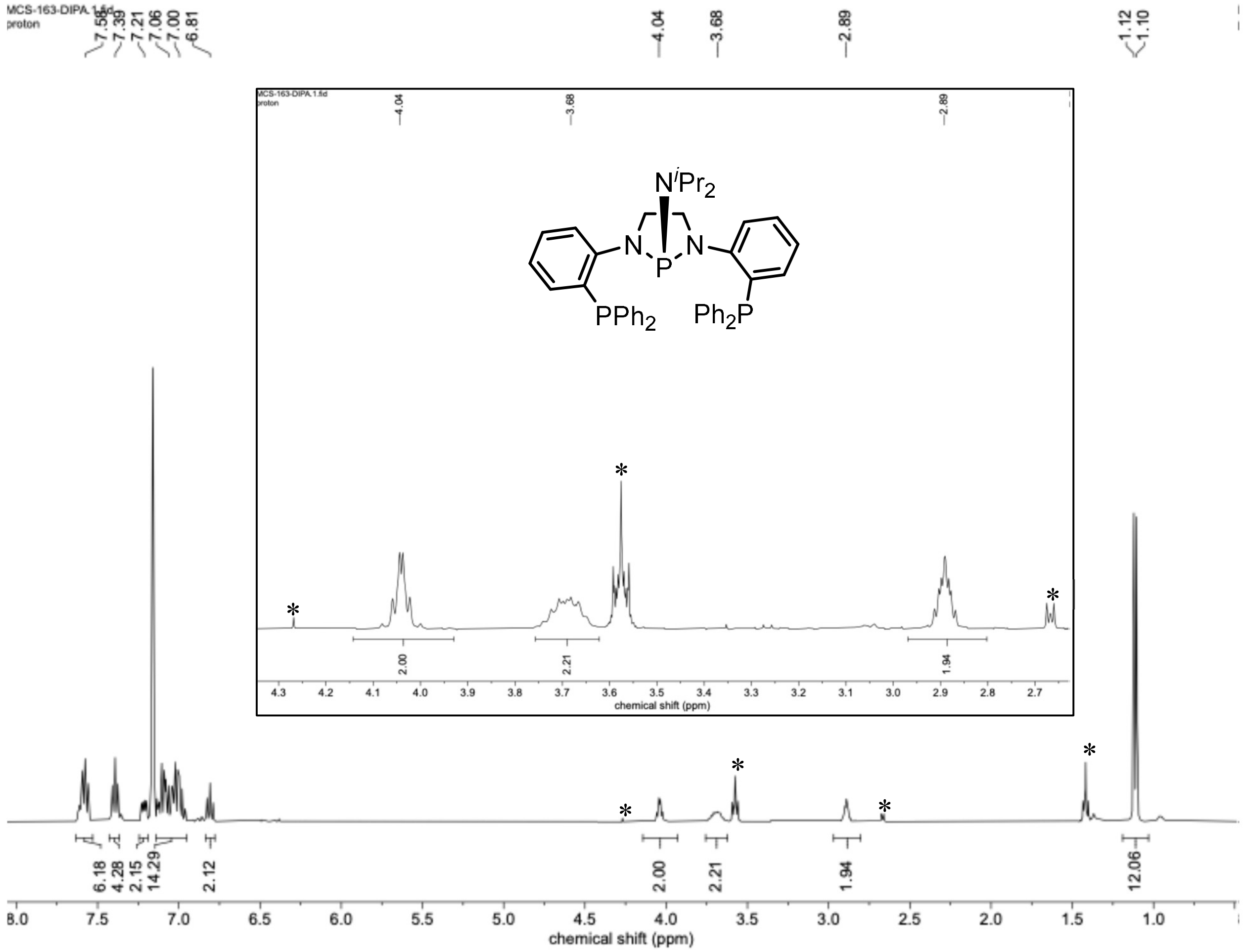

Figure S4: ^{1}H NMR (C_6D_6, 400 MHz) spectrum of (PP$^{Ni\text{Pr}2}$P) (**6**). Residual solvent denoted with an asterisk (*).

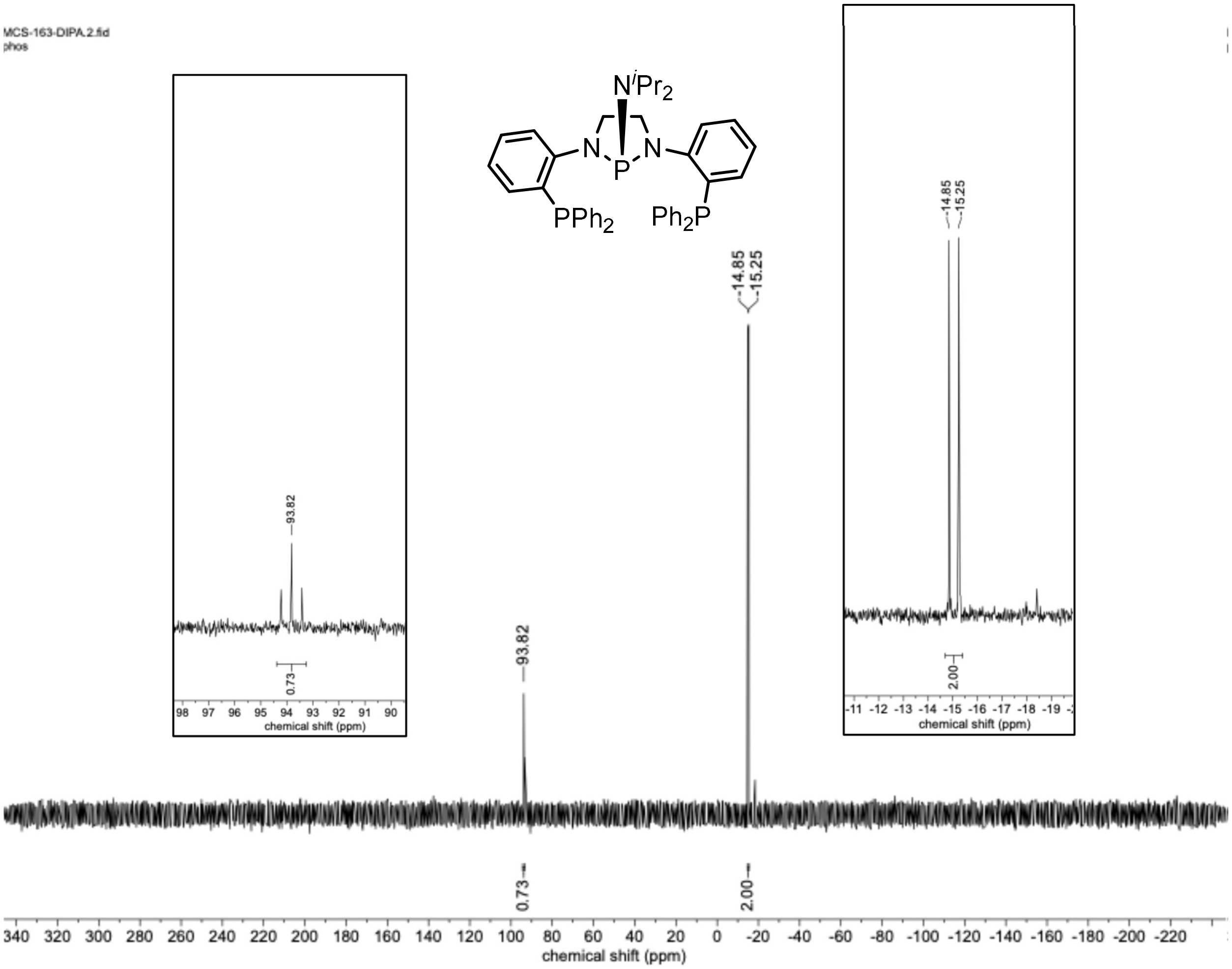

Figure S5: $^{31}P\{^{1}H\}$ NMR (C_6D_6, 162 MHz) spectrum of ($PP^{N\mathit{i}Pr2}P$) (**6**).

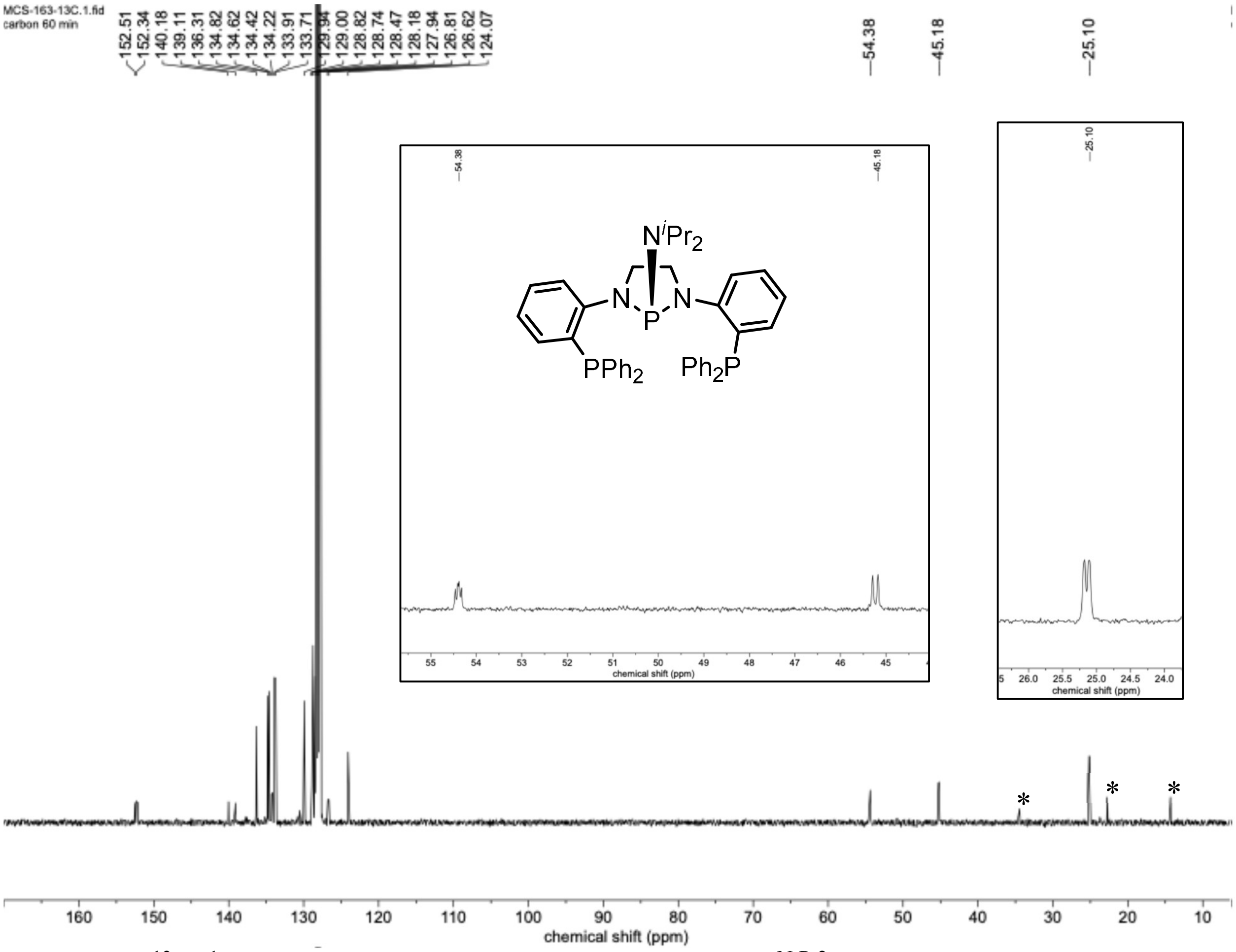

Figure S6: $^{13}C\{^{1}H\}$ NMR (C_6D_6, 151 MHz) spectrum of (PP$^{N\textit{i}Pr2}$P (**6**). Residual solvent denoted with an asterisk (*).

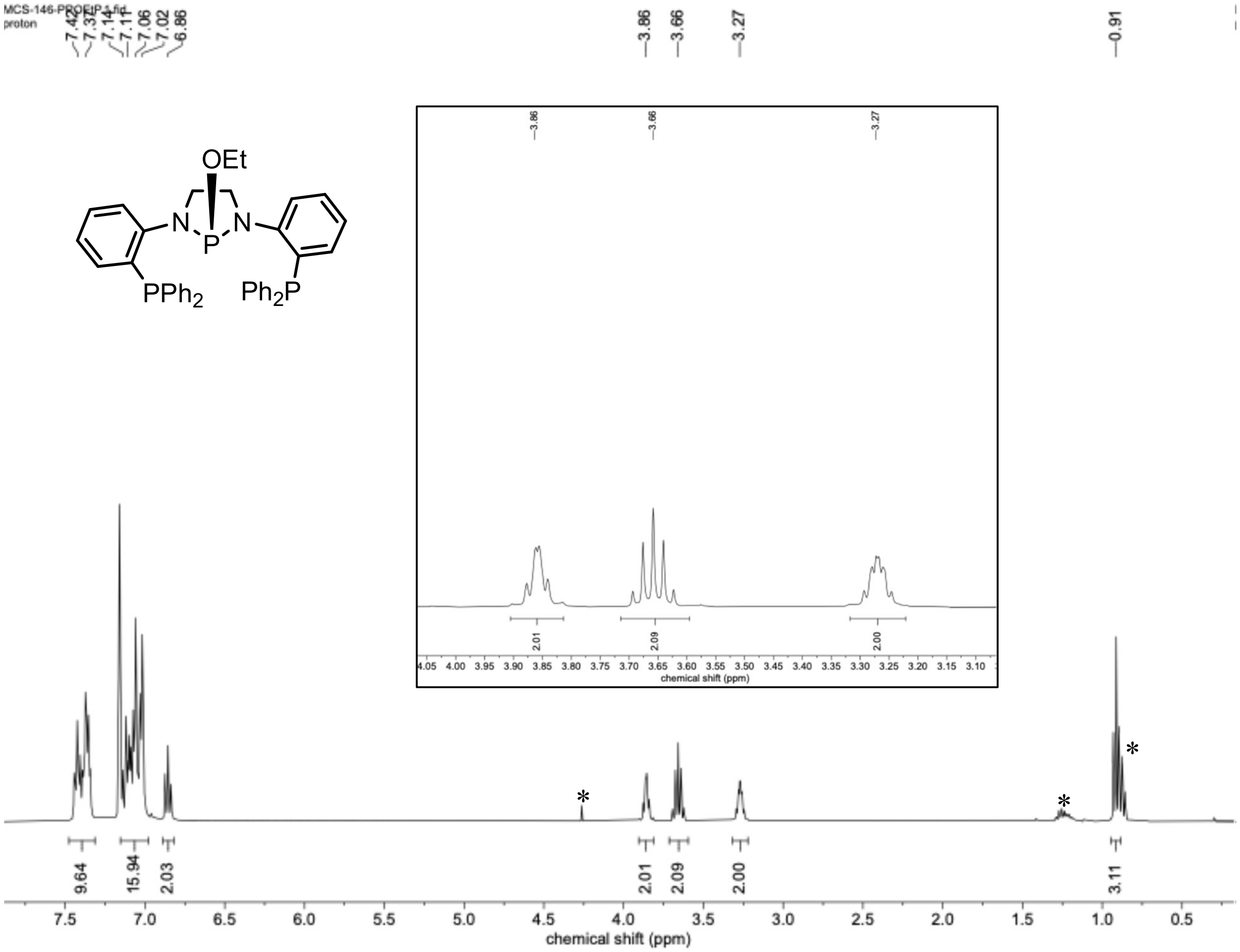

Figure S7: ^{1}H NMR (C_6D_6, 400 MHz) spectrum of ($PP^{OEt}P$) (**7**). Residual solvent denoted with an asterisk (*).

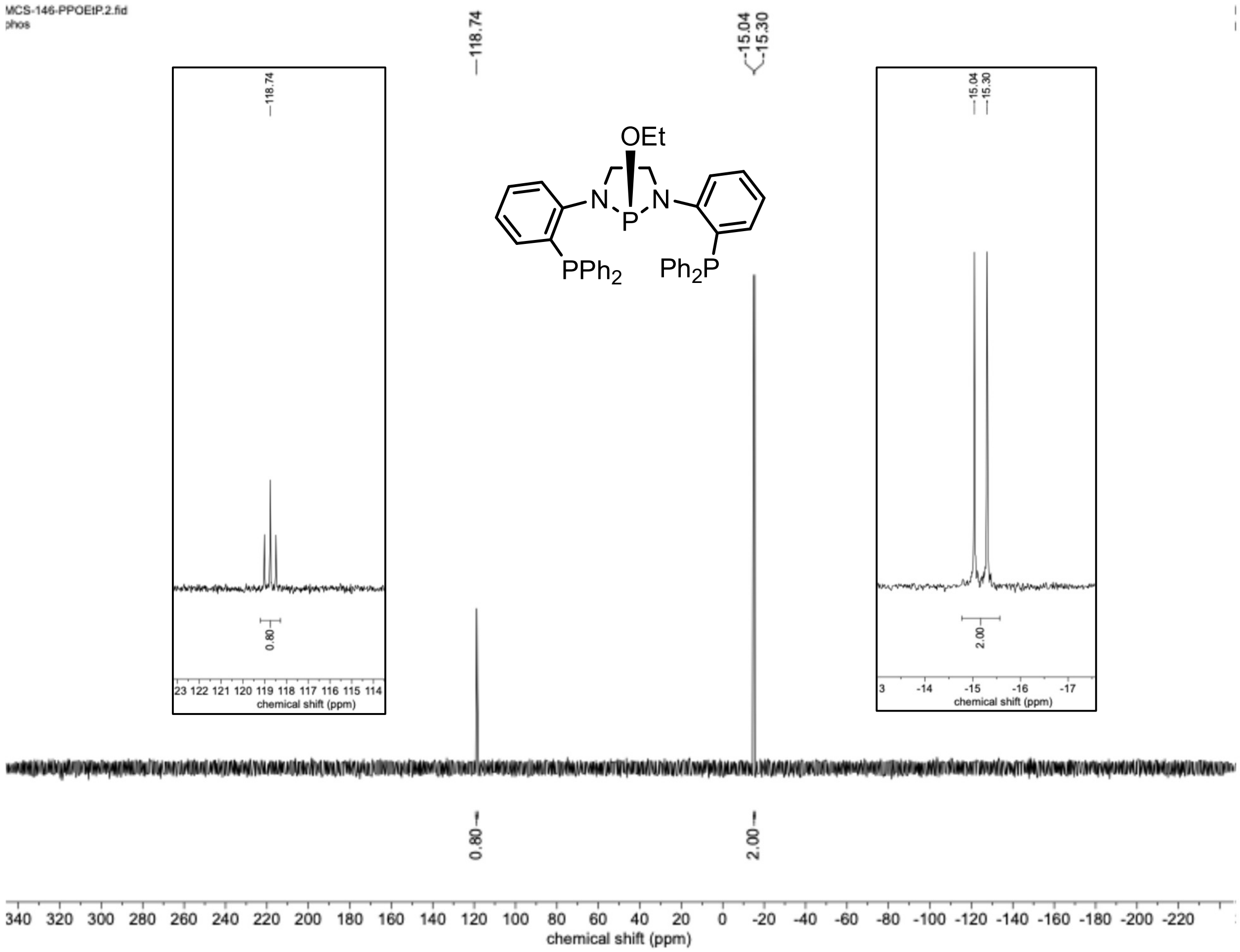

Figure S8: $^{31}P\{^{1}H\}$ NMR (C_6D_6, 162 MHz) spectrum of ($PP^{OEt}P$) (**7**).

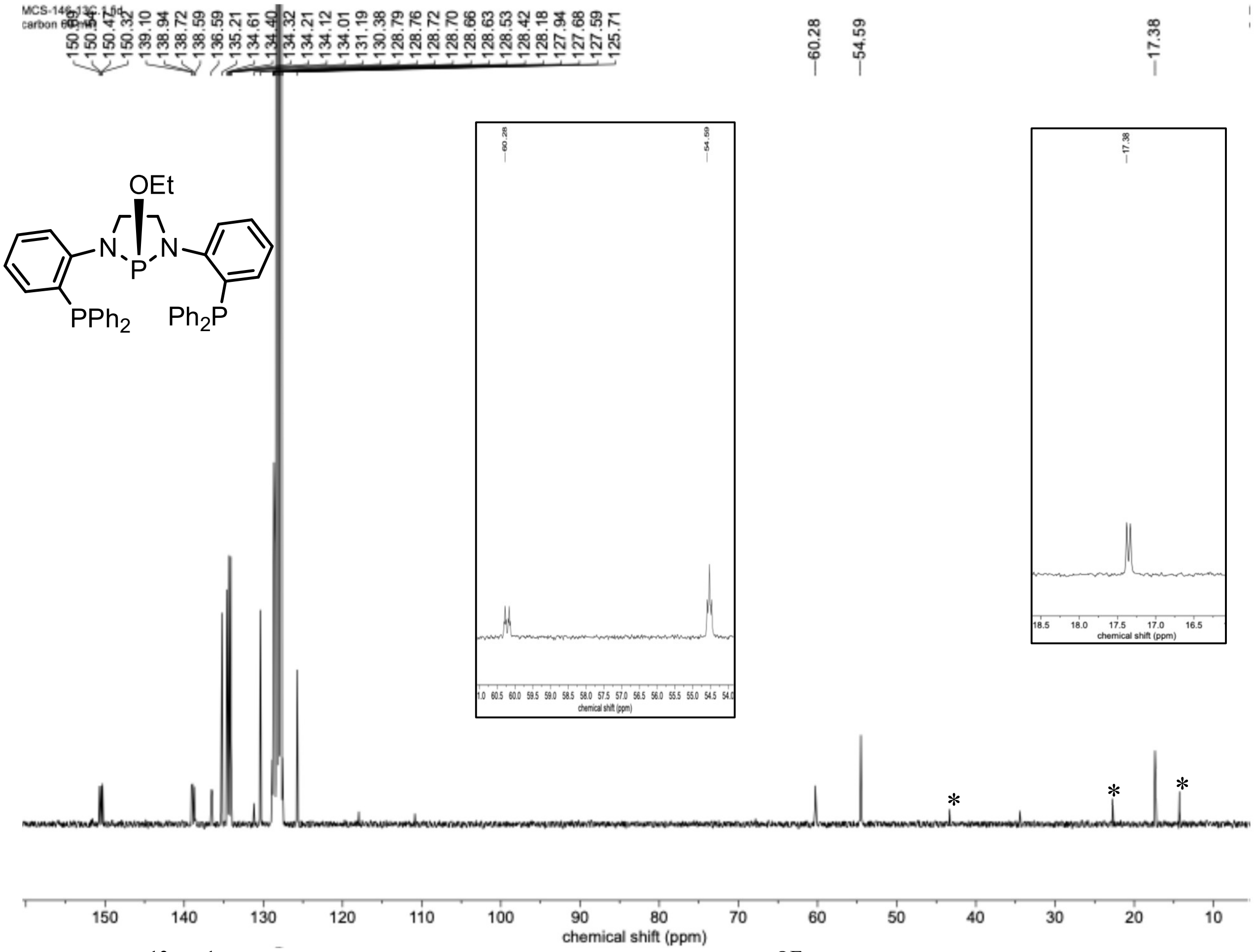

Figure S9: $^{13}C\{^1H\}$ NMR (C_6D_6, 151 MHz) spectrum of ($PP^{OEt}P$) (**7**). Residual solvent denoted with an asterisk (*).

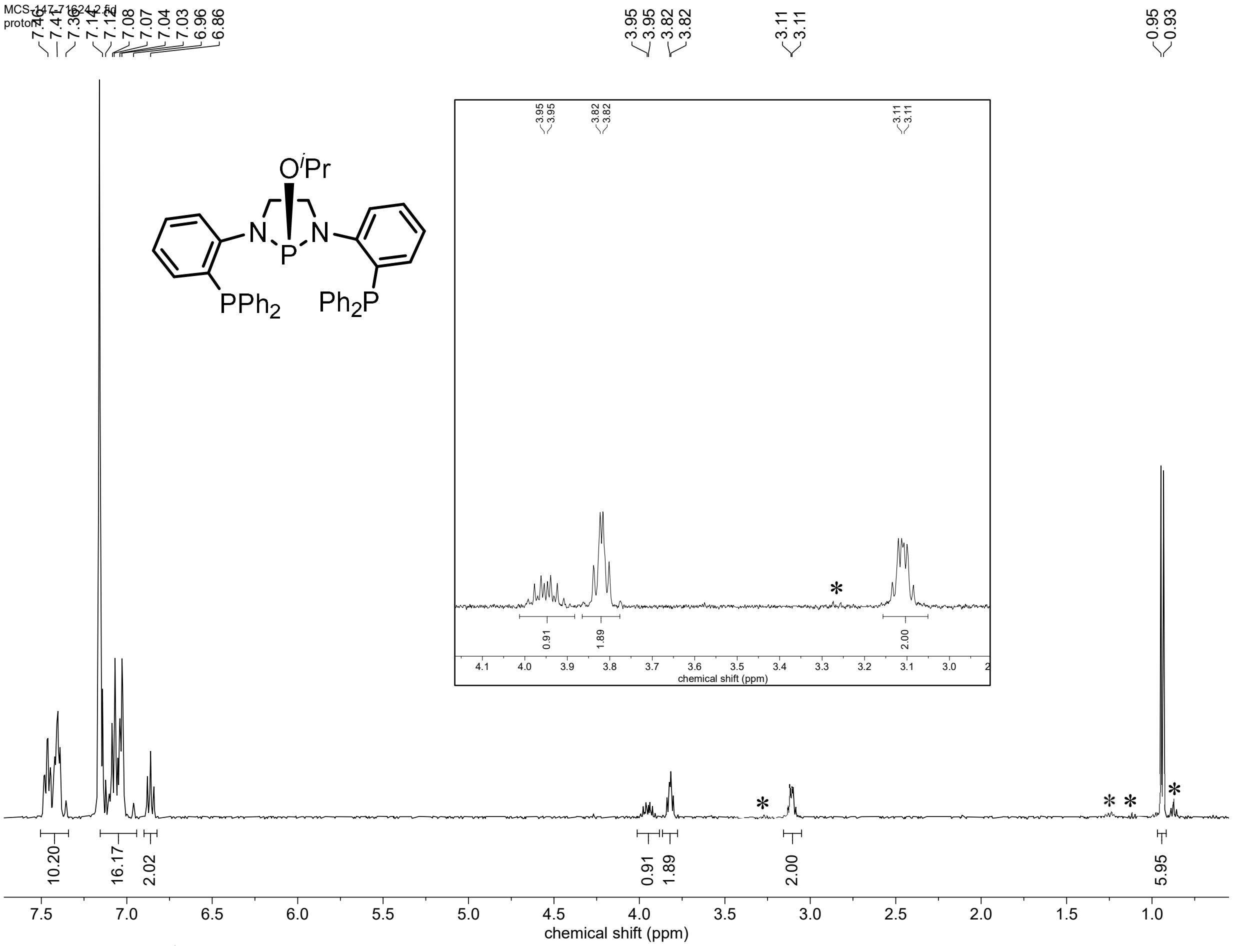

Figure S10: ^{1}H NMR (C_6D_6, 400 MHz) spectrum of ($PP^{O\mathit{i}Pr}P$) (**8**). Residual solvent denoted with an asterisk (*).

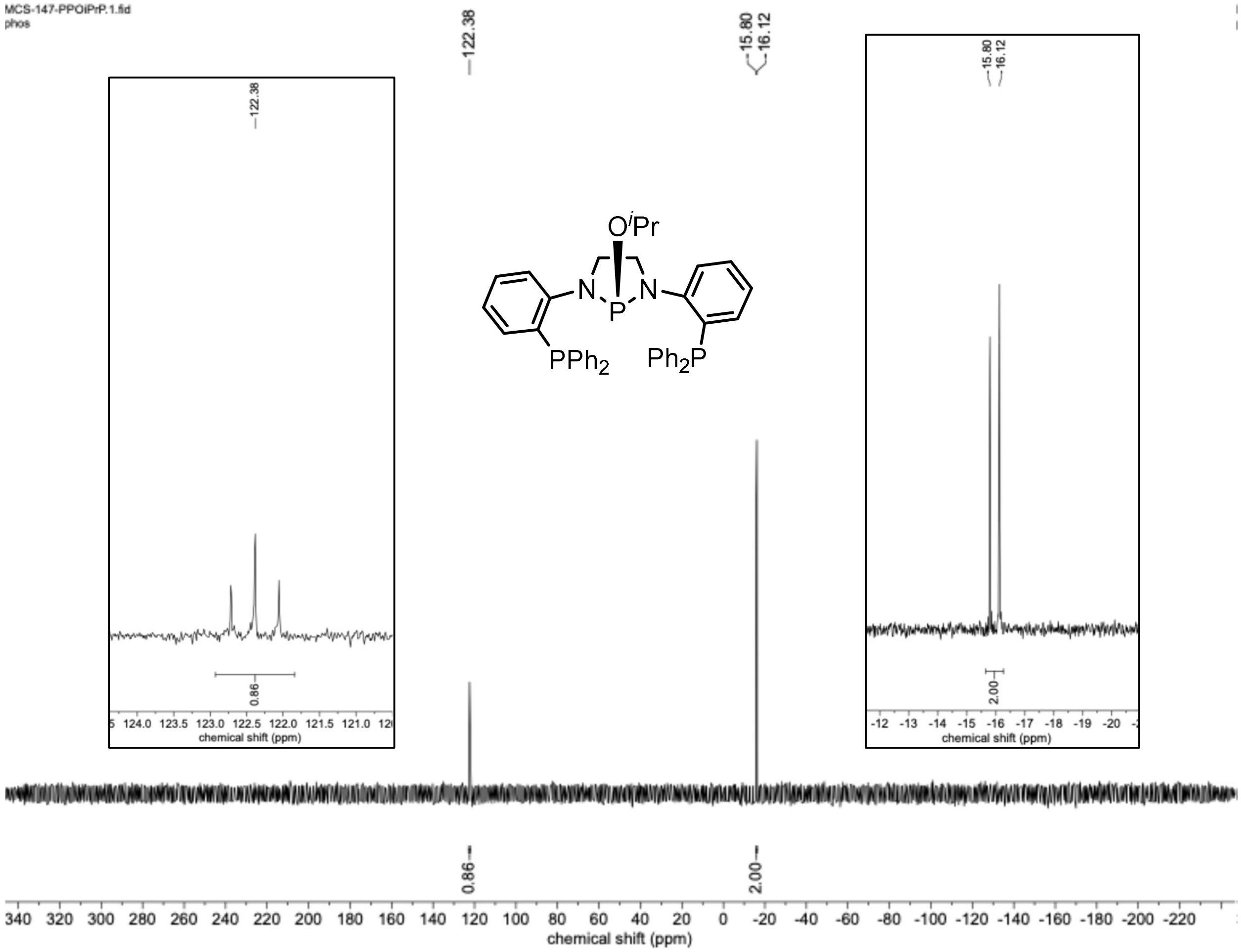

Figure S11: $^{31}P\{^{1}H\}$ NMR (C_6D_6, 162 MHz) spectrum of ($PP^{O\textit{i}Pr}P$) (**8**).

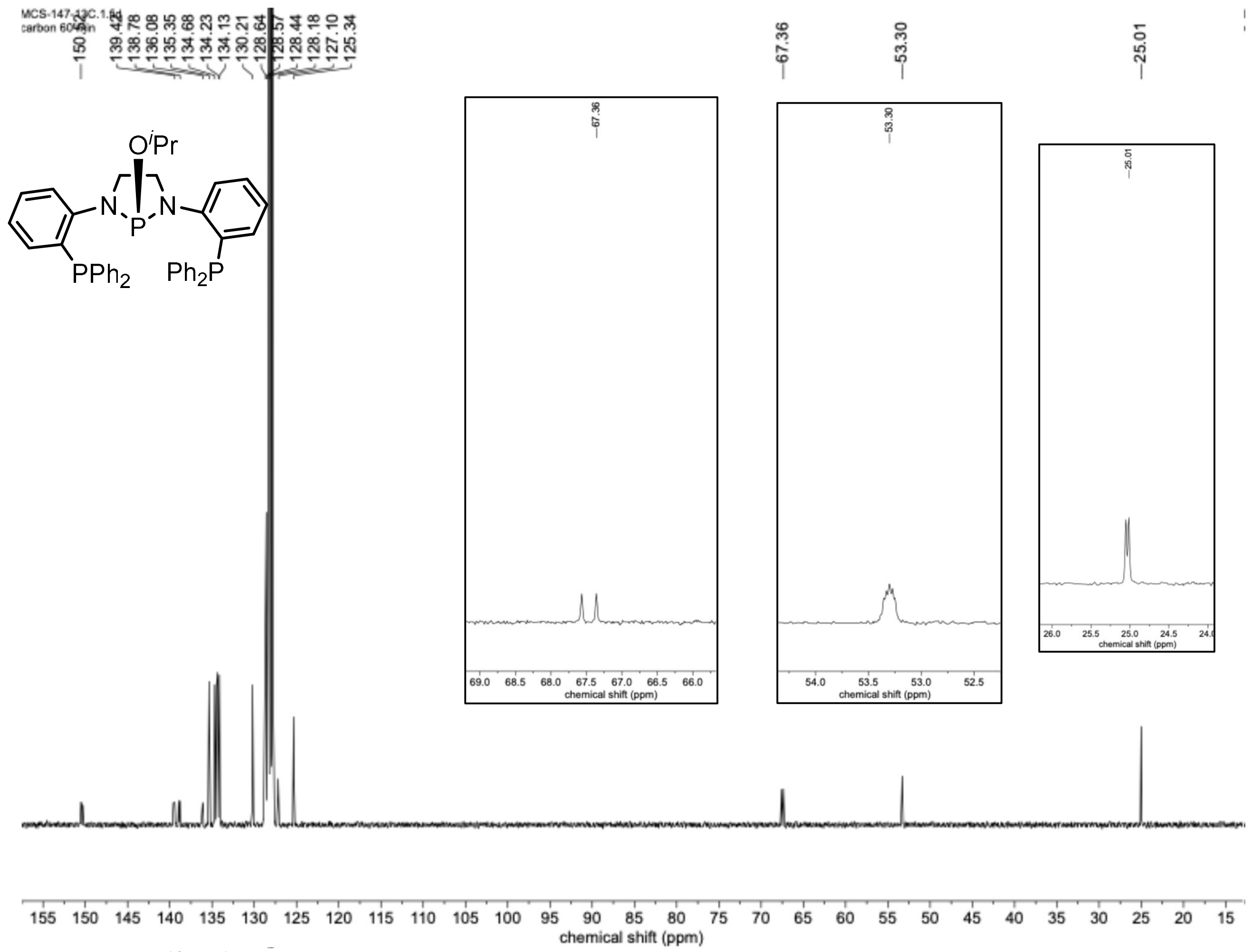

Figure S12: $^{13}C\{^1H\}$ NMR (C_6D_6, 151 MHz) spectrum of ($PP^{OiPr}P$) (**8**).

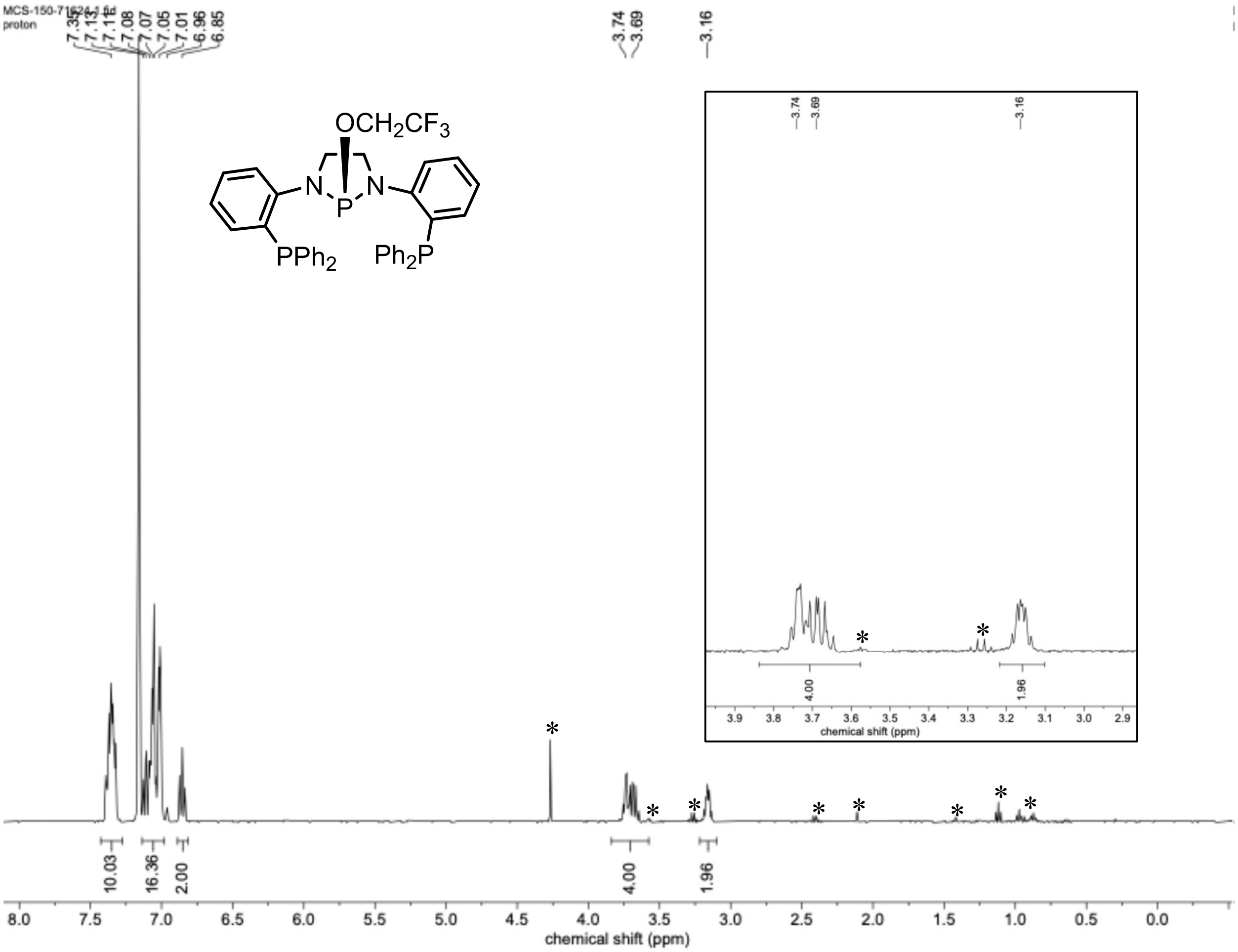

Figure S13: ^{1}H NMR (C_6D_6, 400 MHz) spectrum of ($PP^{OCH2CF3}P$) (**9**). Residual solvent denoted with an asterisk (*).

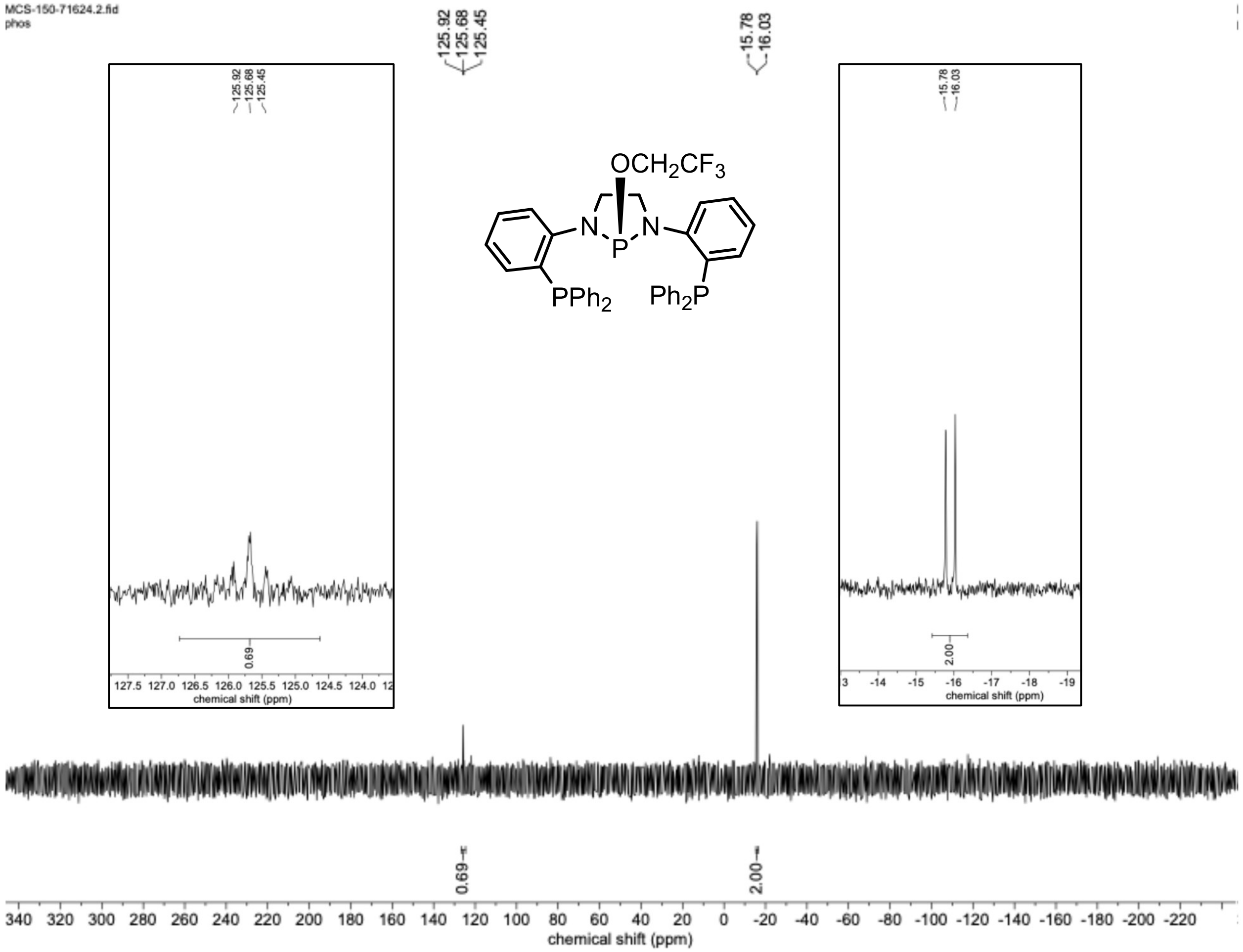

Figure S14: $^{31}P\{^{1}H\}$ NMR (C_6D_6, 162 MHz) spectrum of ($PP^{OCH2CF3}P$) (**9**).

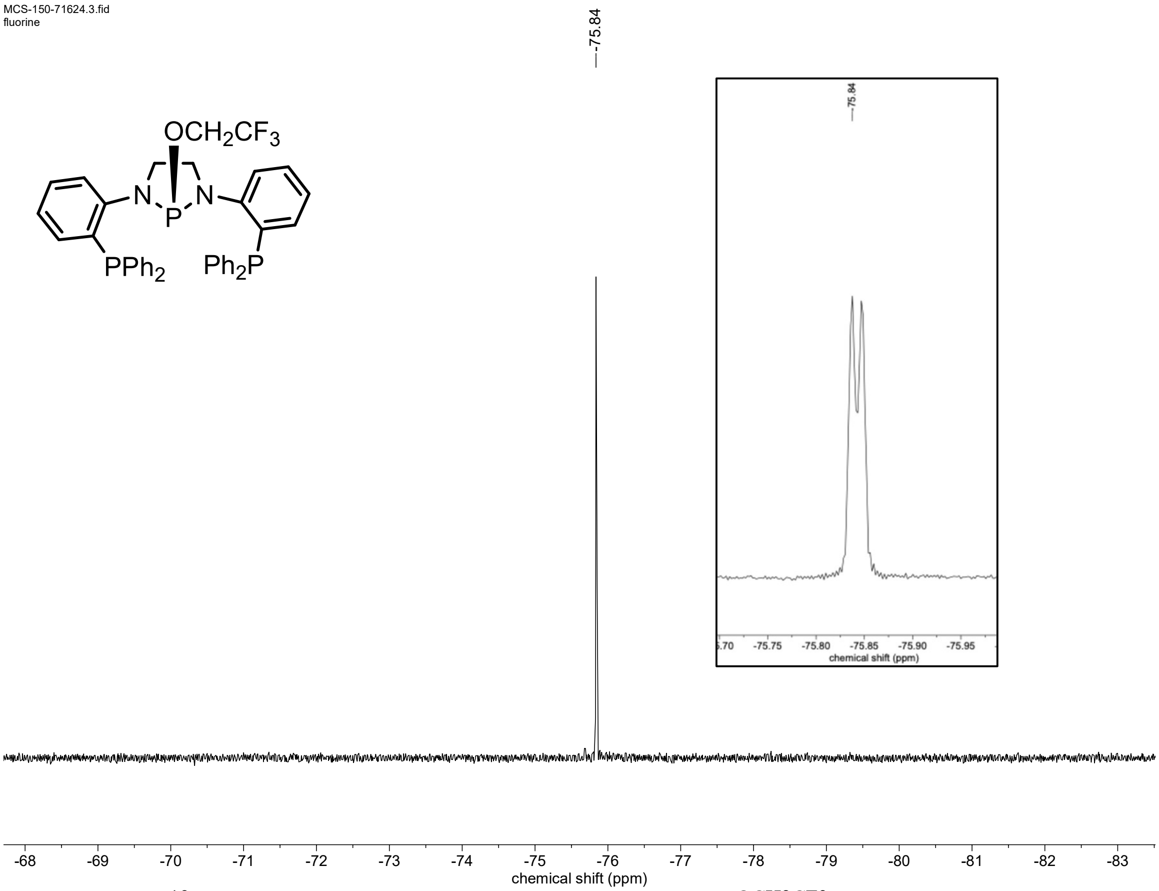

Figure S15: ^{19}F NMR (C_6D_6, 377 MHz) spectrum of ($PP^{OCH2CF3}P$) (**9**).

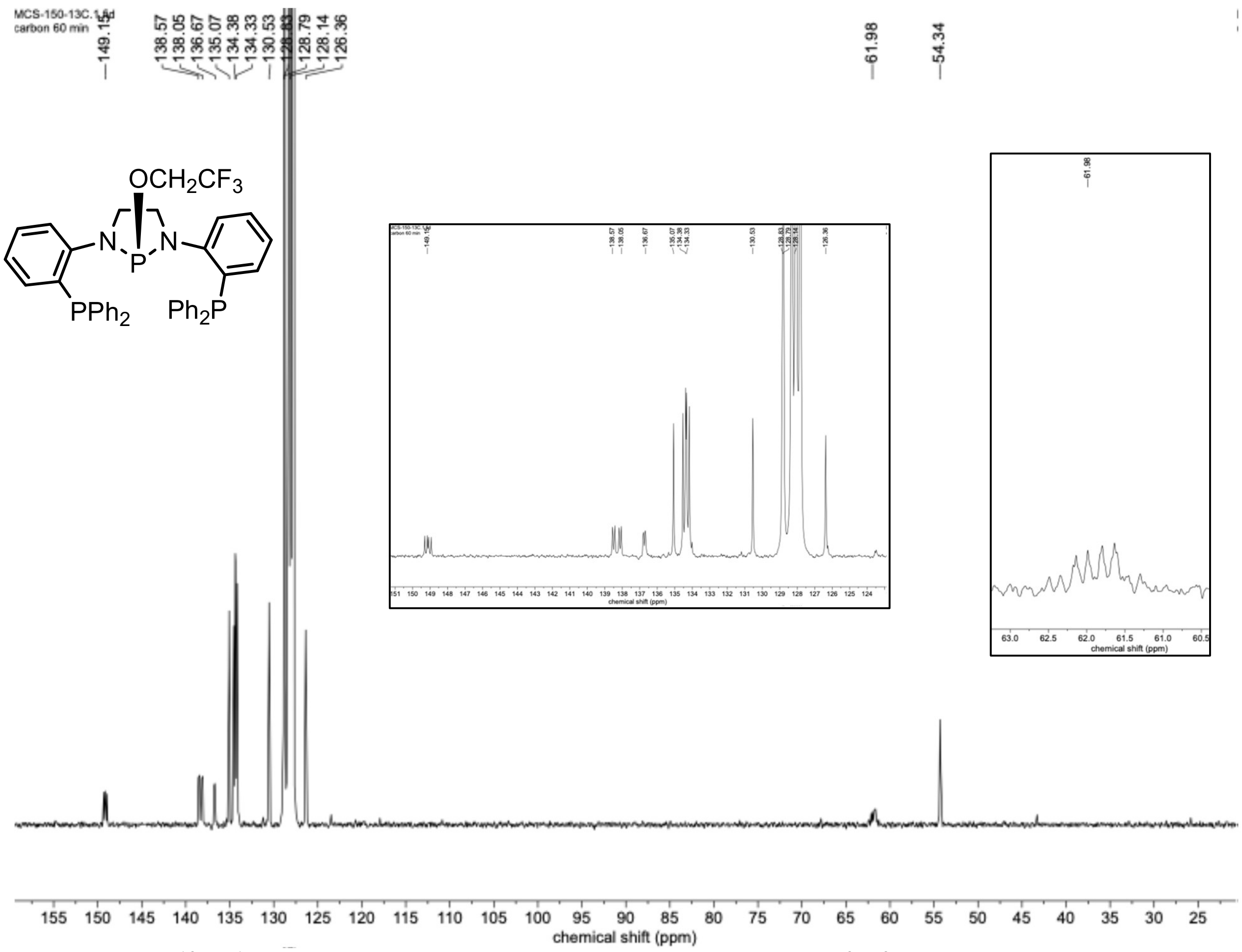

Figure S16: $^{13}C\{^{1}H\}$ NMR (C_6D_6, 151 MHz) spectrum of ($PP^{OCH2CF3}P$) (**9**).

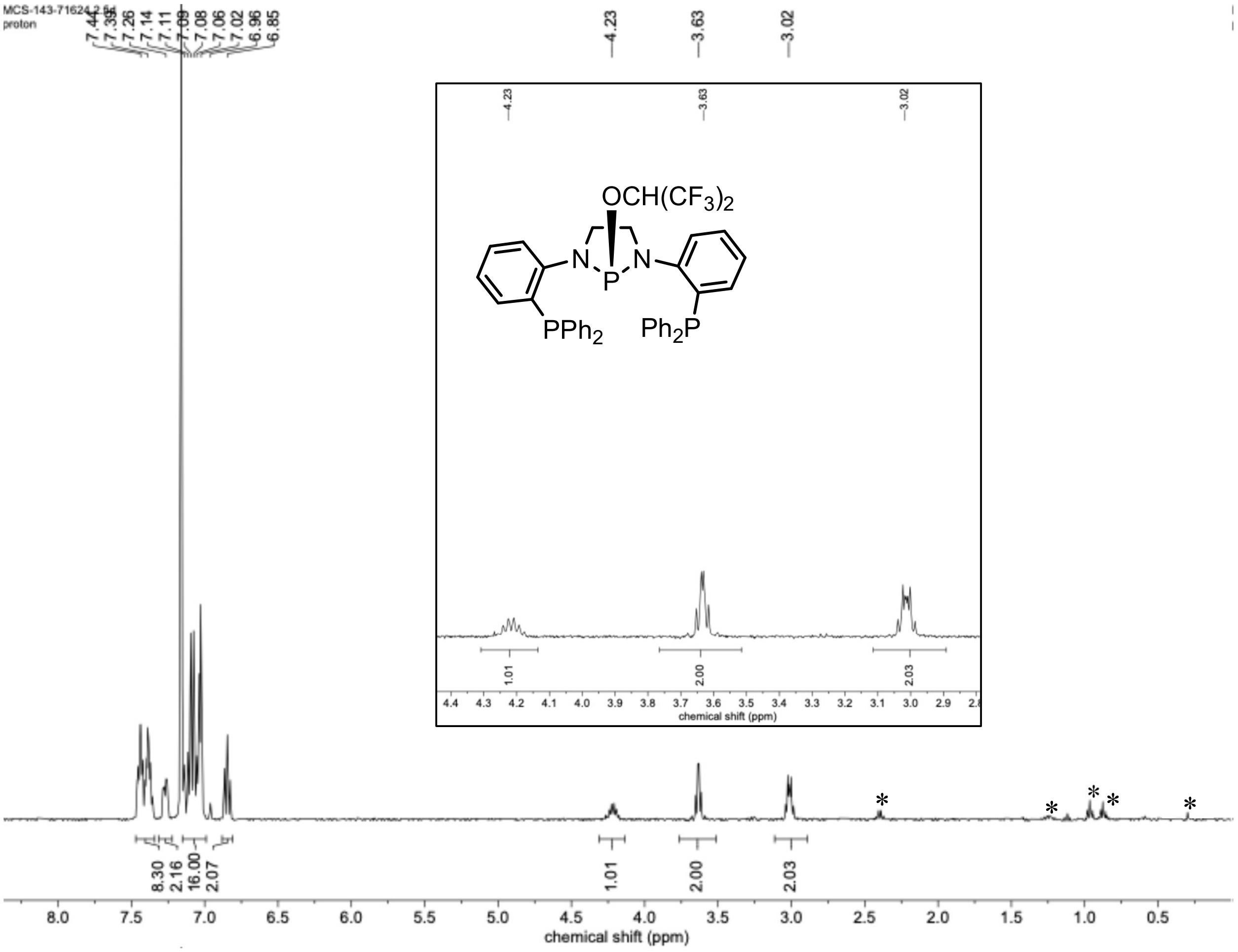

Figure S17: ^{1}H NMR (C_6D_6, 400 MHz) spectrum of ($PP^{OCH(CF3)2}P$) (**10**). Residual solvent denoted with an asterisk (*).

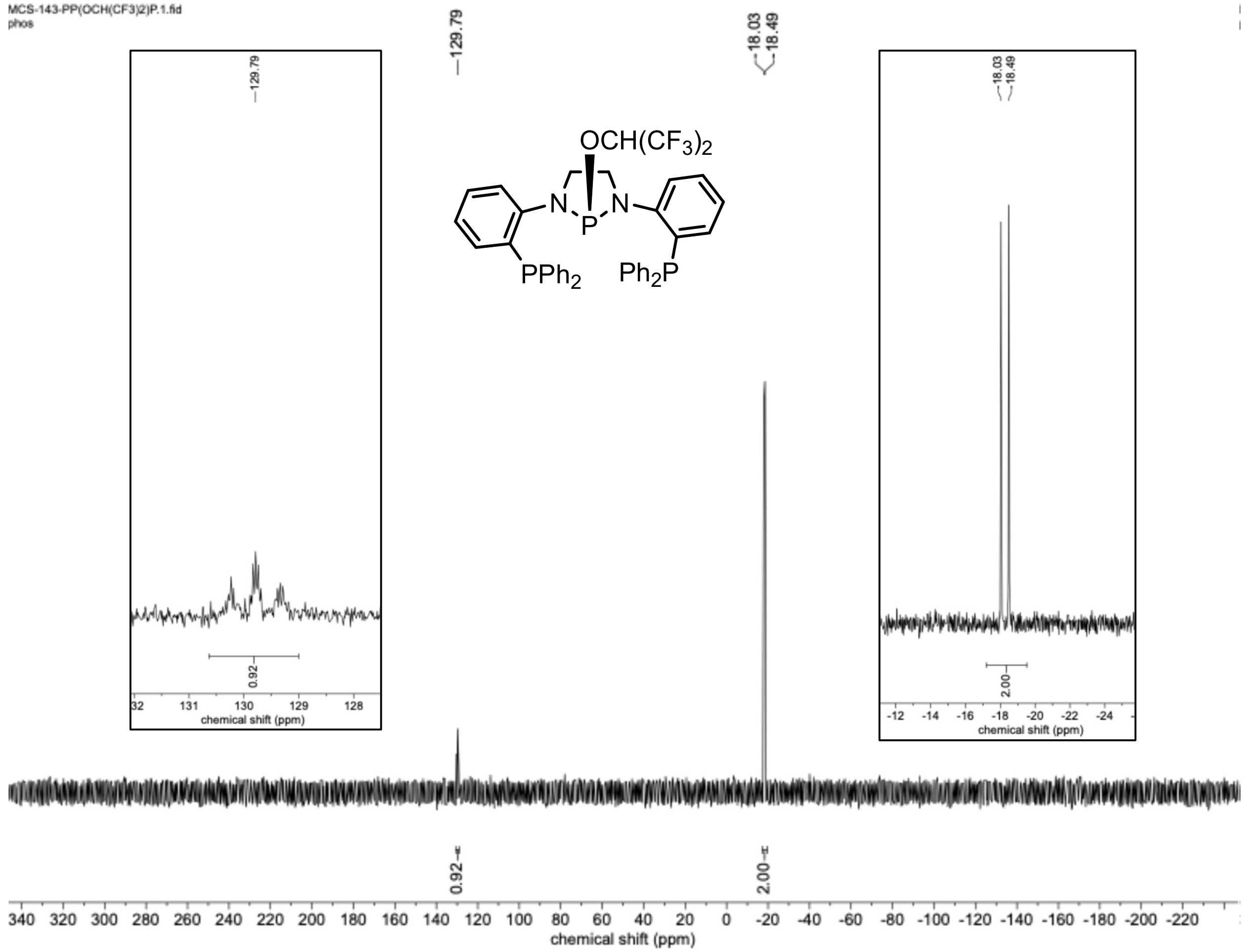

Figure S18: $^{31}P\{^{1}H\}$ NMR (C_6D_6, 162 MHz) spectrum of ($PP^{OCH(CF3)2}P$) (**10**).

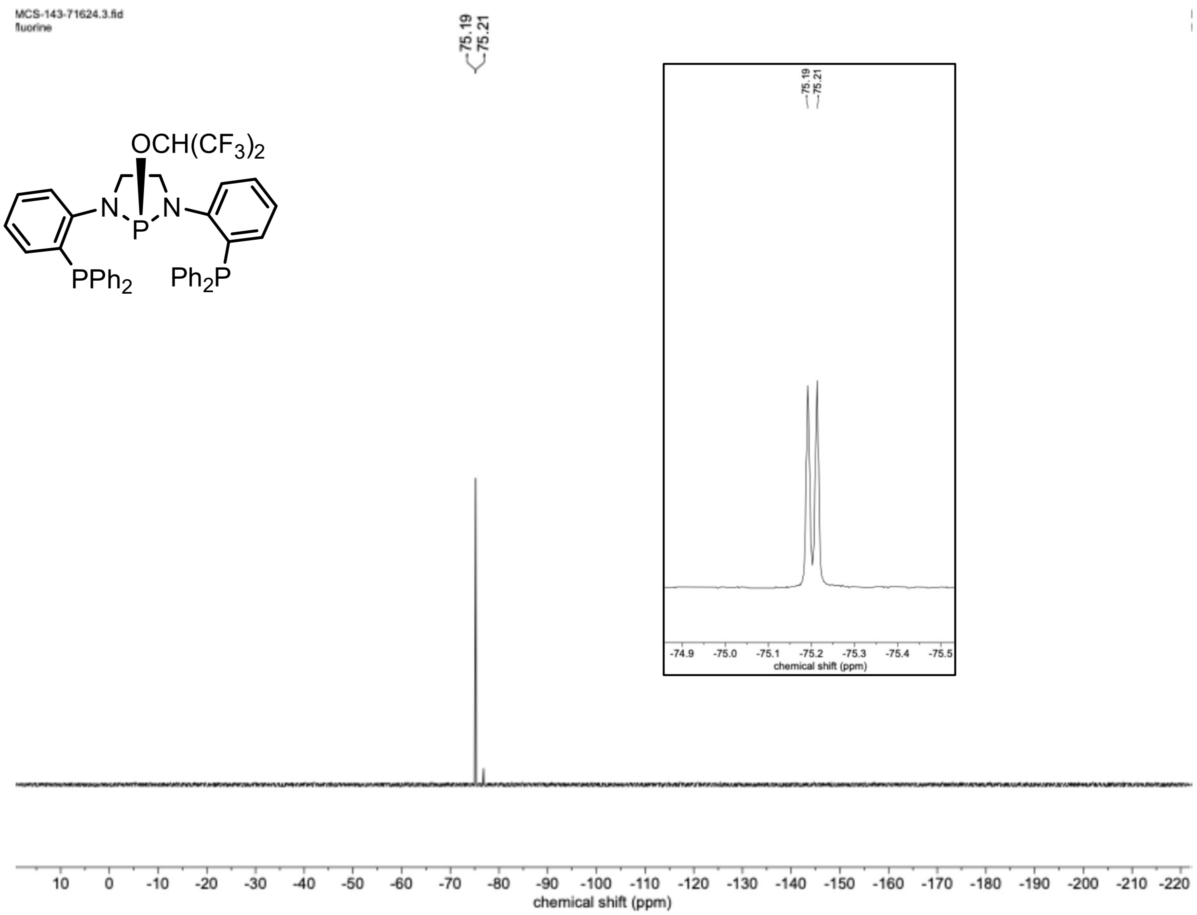

Figure S19: ^{19}F NMR (C_6D_6, 377 MHz) spectrum of ($PP^{OCH(CF3)2}P$) (**10**).

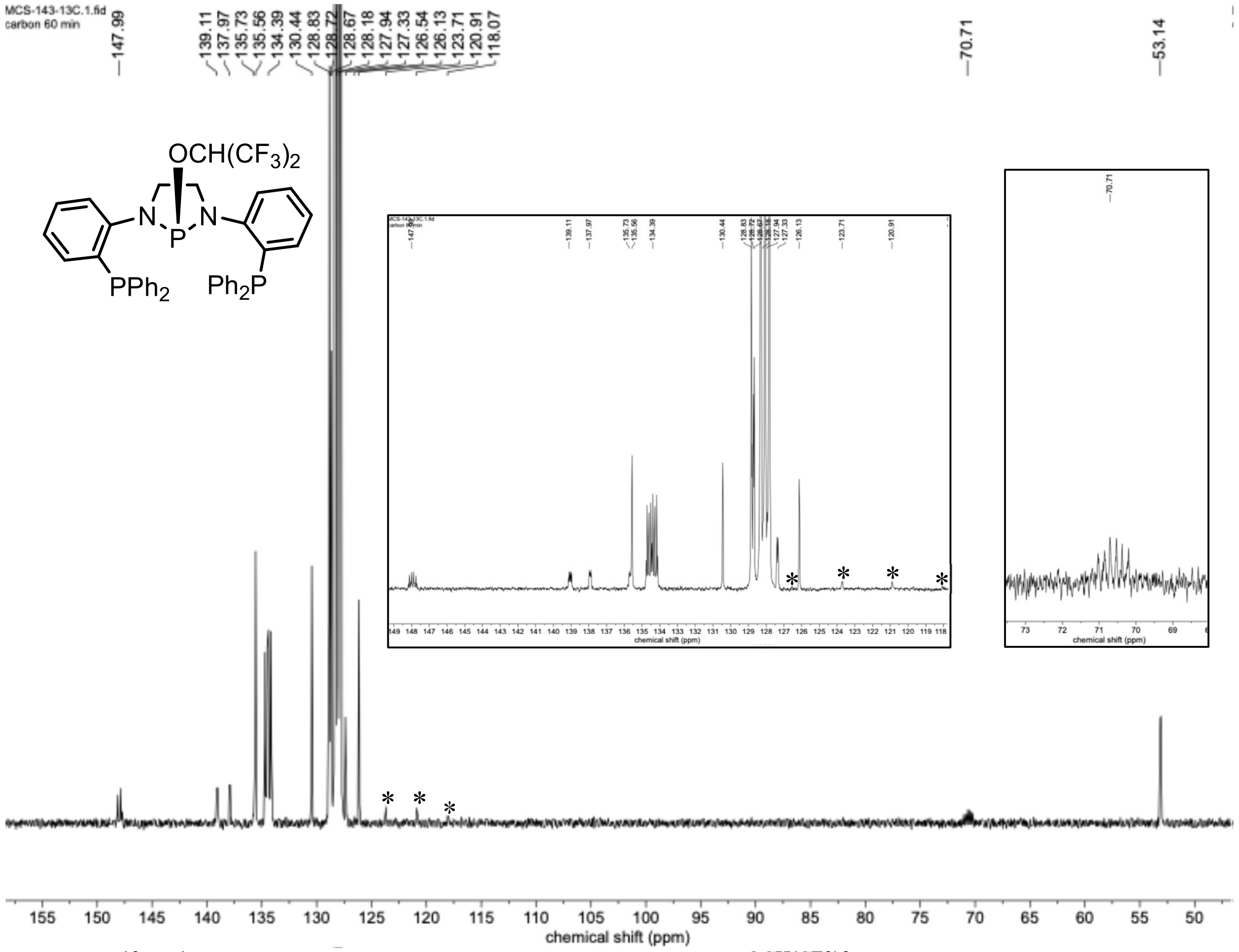

Figure S20: $^{13}C\{^1H\}$ NMR (C_6D_6, 151 MHz) spectrum of ($PP^{OCH(CF3)2}P$) (**10**). CF_3 quartet signal denoted with asterisks (*).

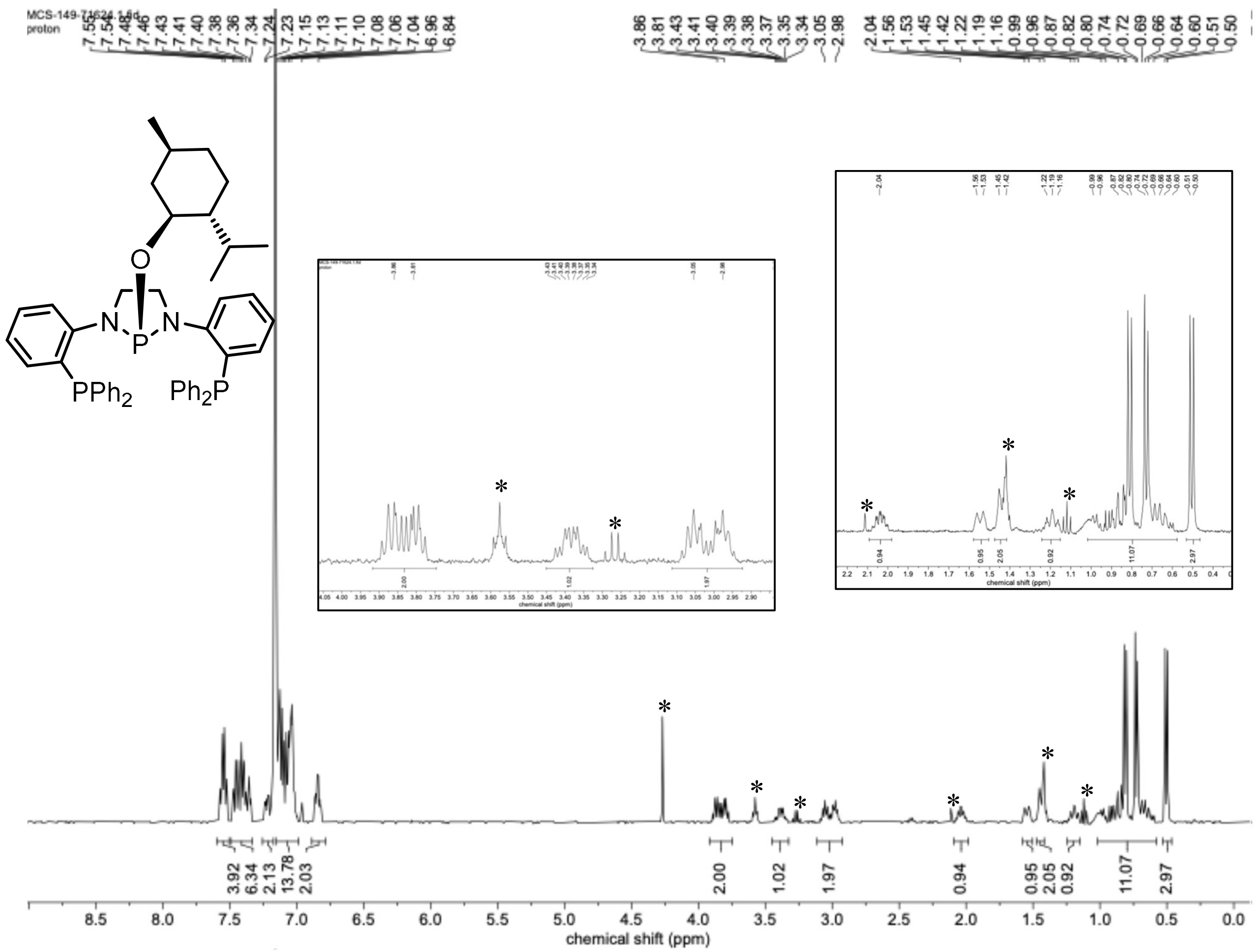

Figure S21: ^{1}H NMR (C_6D_6, 400 MHz) spectrum of ($PP^{menthoxide}P$) (**11**). Residual solvent denoted with an asterisk (*).

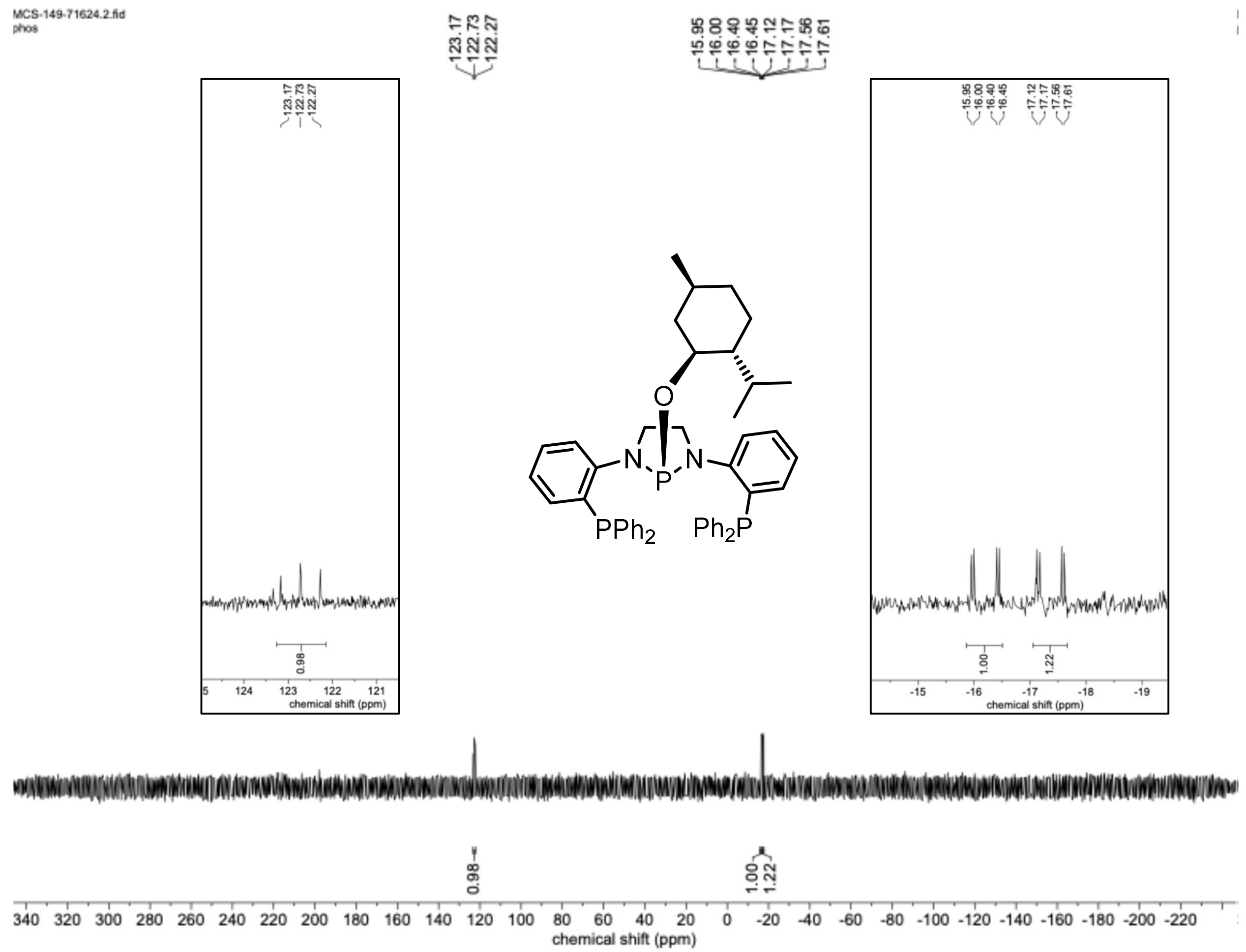

Figure S22: $^{31}P\{^{1}H\}$ NMR (C_6D_6, 162 MHz) spectrum of ($PP^{menthoxide}P$) (**11**).

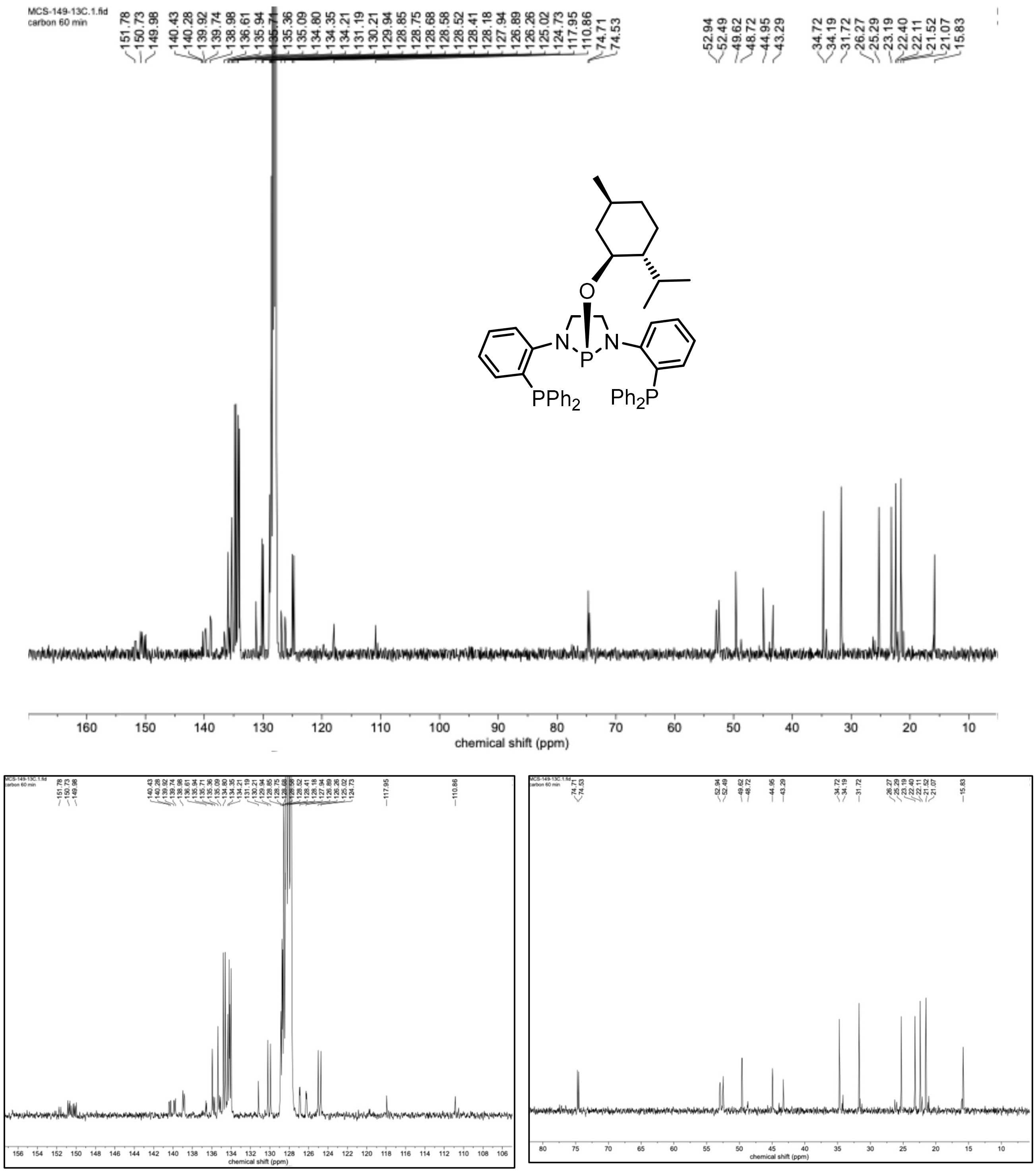

Figure S23: $^{13}C\{^{1}H\}$ NMR (C_6D_6, 151 MHz) spectrum of (PP^{menthoxide}P) (**11**). Residual solvent denoted with an asterisk (*).

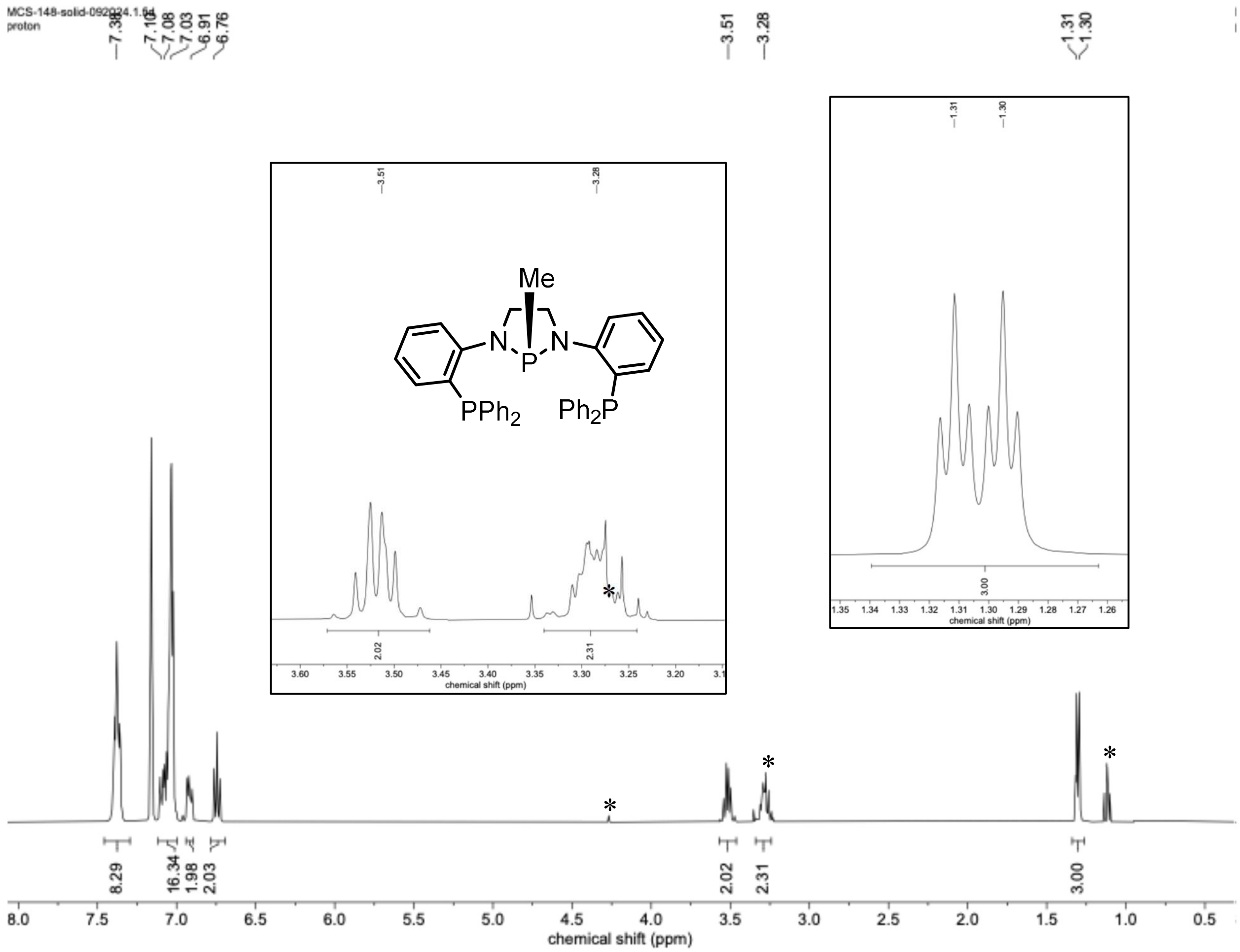

Figure S24: ^{1}H NMR (C_6D_6, 400 MHz) spectrum of ($PP^{Me}P$) (**12**). Residual solvent denoted with an asterisk (*).

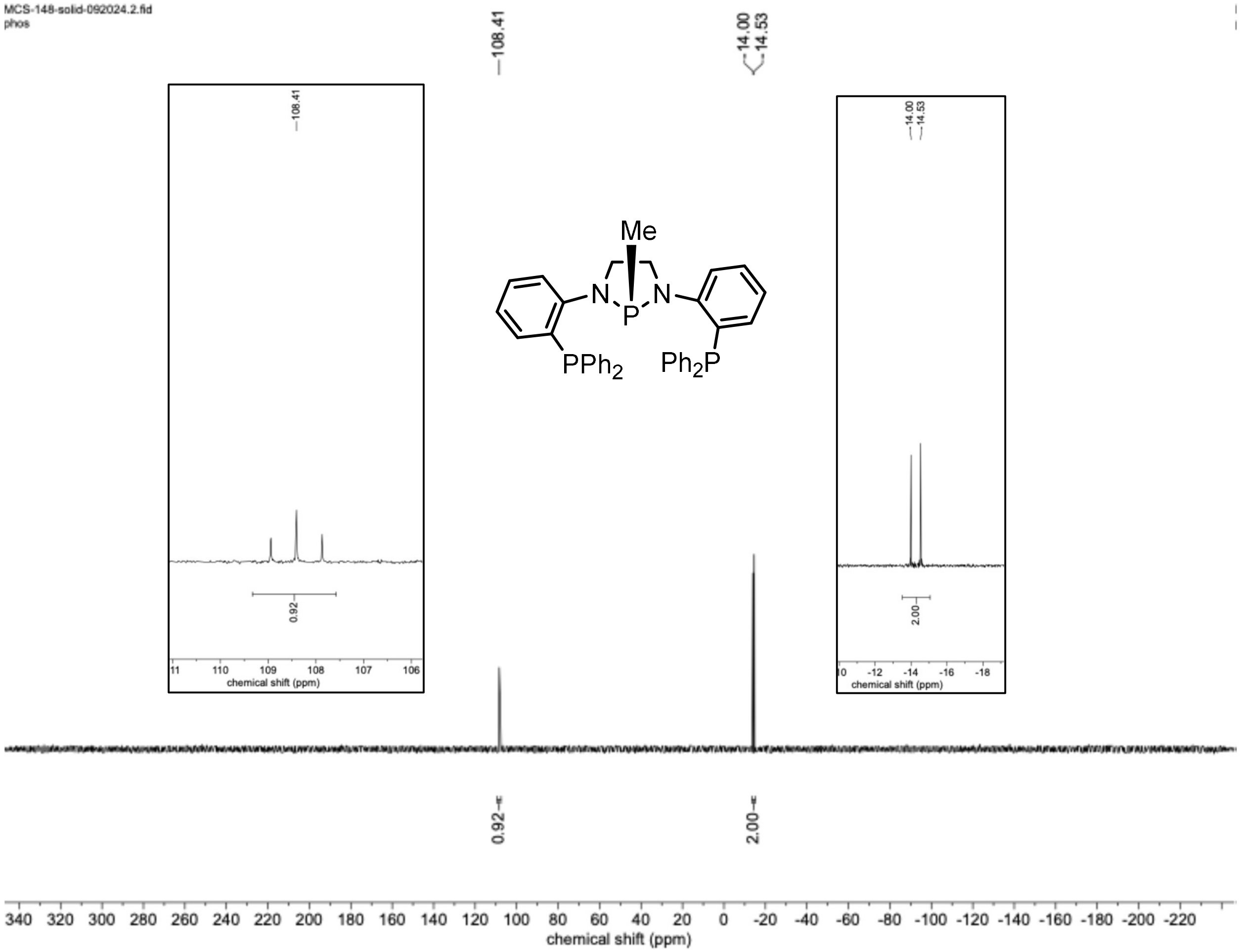

Figure S25: $^{31}P\{^{1}H\}$ NMR (C_6D_6, 162 MHz) spectrum of ($PP^{Me}P$) (**12**).

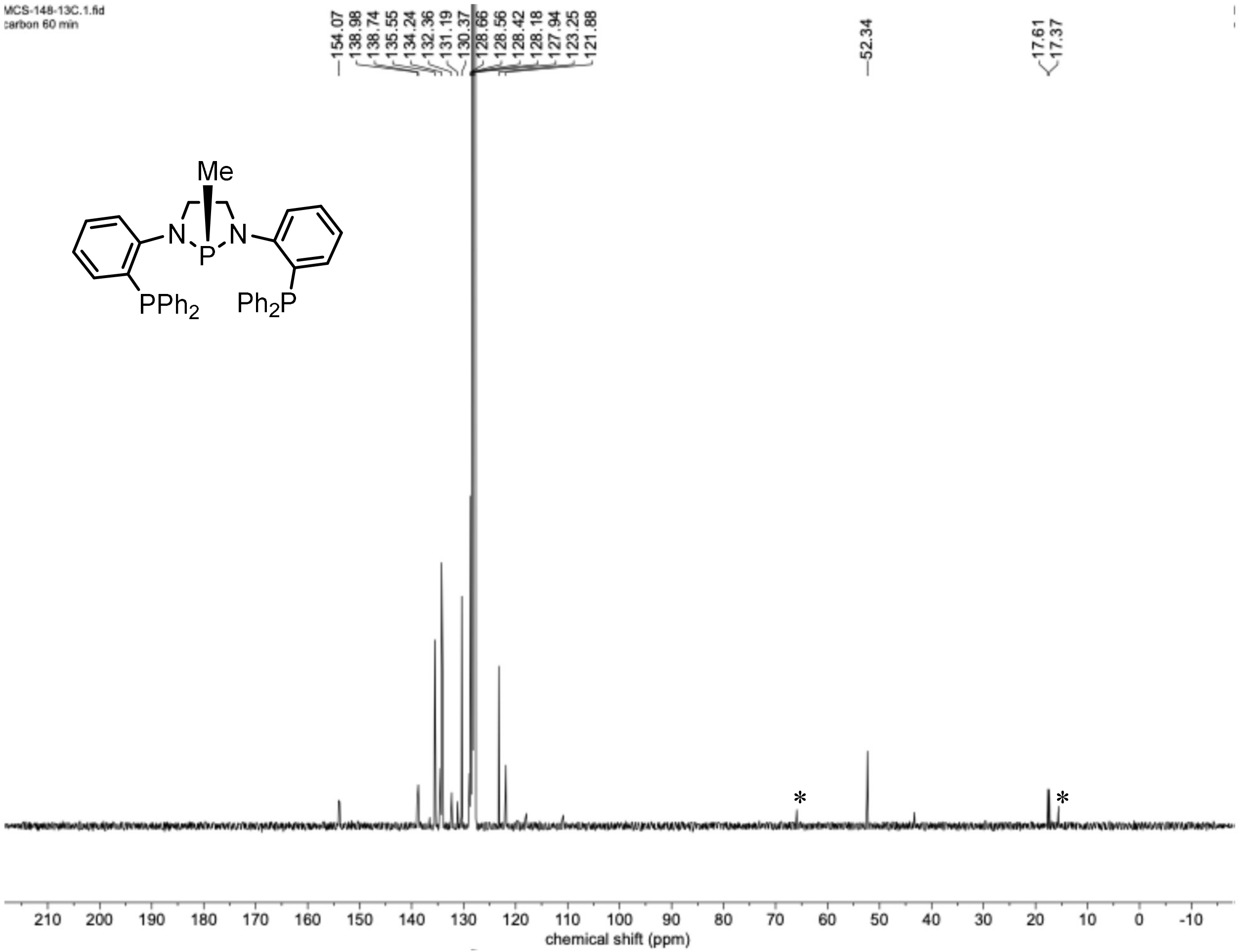

Figure S26: $^{13}C\{^1H\}$ NMR (C_6D_6, 151 MHz) spectrum of ($PP^{Me}P$) (**12**). Residual solvent denoted with an asterisk (*).

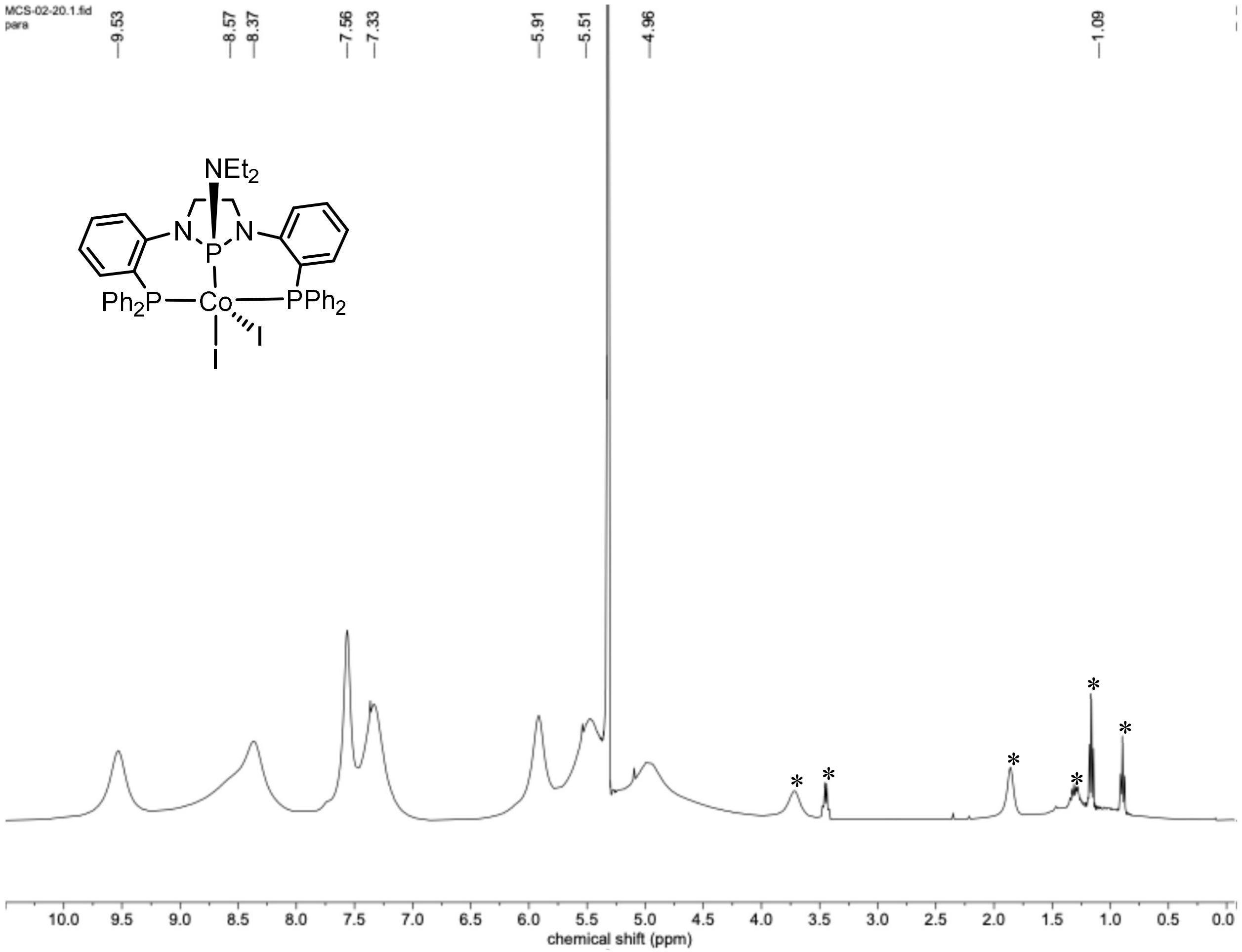

Figure S27: ^{1}H NMR (CD_2Cl_2, 400 MHz) spectrum of ($PP^{NEt2}P$)CoI_2 (**13**). Residual solvent denoted with an asterisk (*).

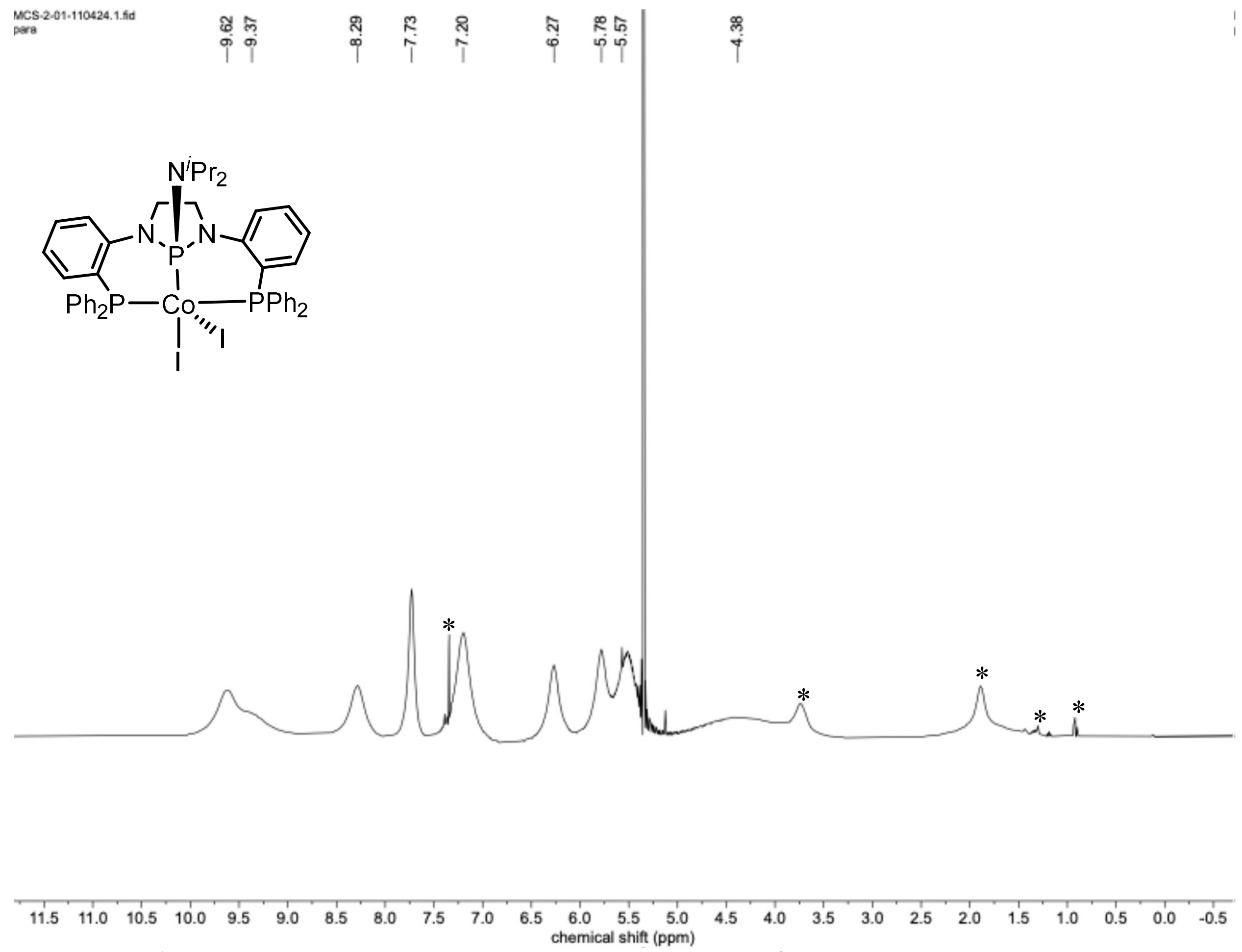

Figure S28: ^{1}H NMR (CD_2Cl_2, 400 MHz) spectrum of (PP$^{N\textit{i}Pr2}$P)CoI_2 (**14**). Residual solvent denoted with an asterisk (*).

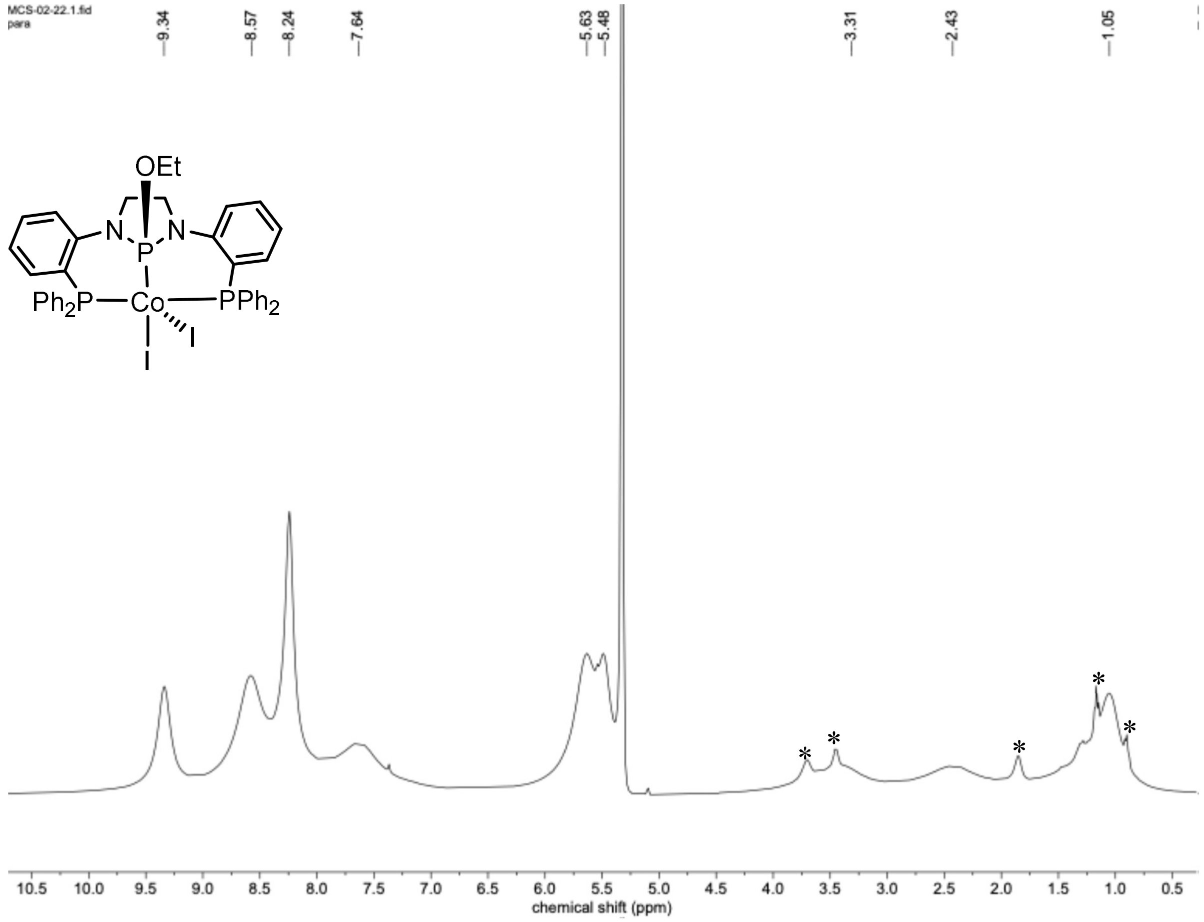

Figure S29: ^{1}H NMR (CD_2Cl_2, 400 MHz) spectrum of $(PP^{OEt}P)CoI_2$ (**15**). Residual solvent denoted with an asterisk (*).

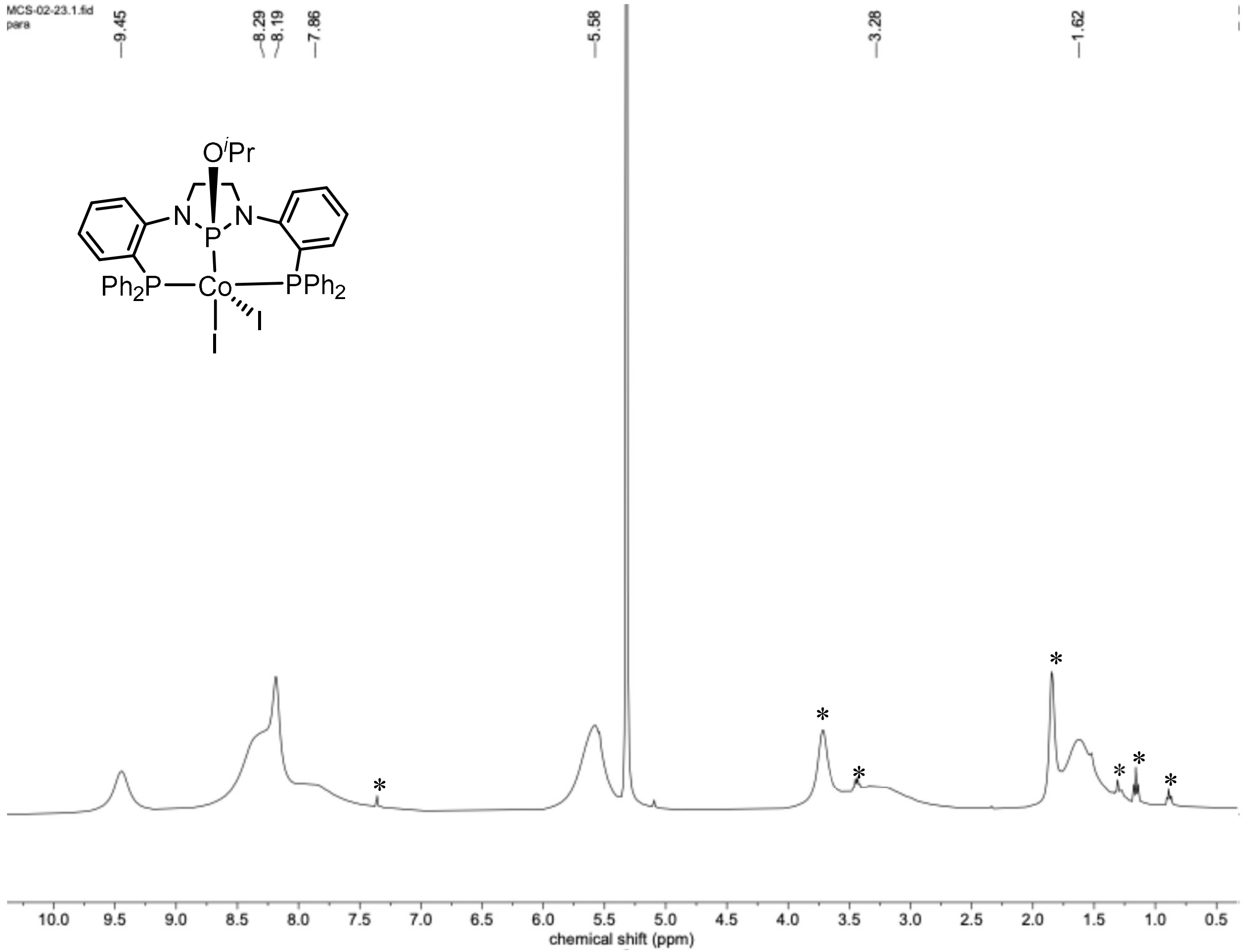

Figure S30: ^{1}H NMR (CD_2Cl_2, 400 MHz) spectrum of (PP^{OiPr}P)CoI_2 (**16**). Residual solvent denoted with an asterisk (*).

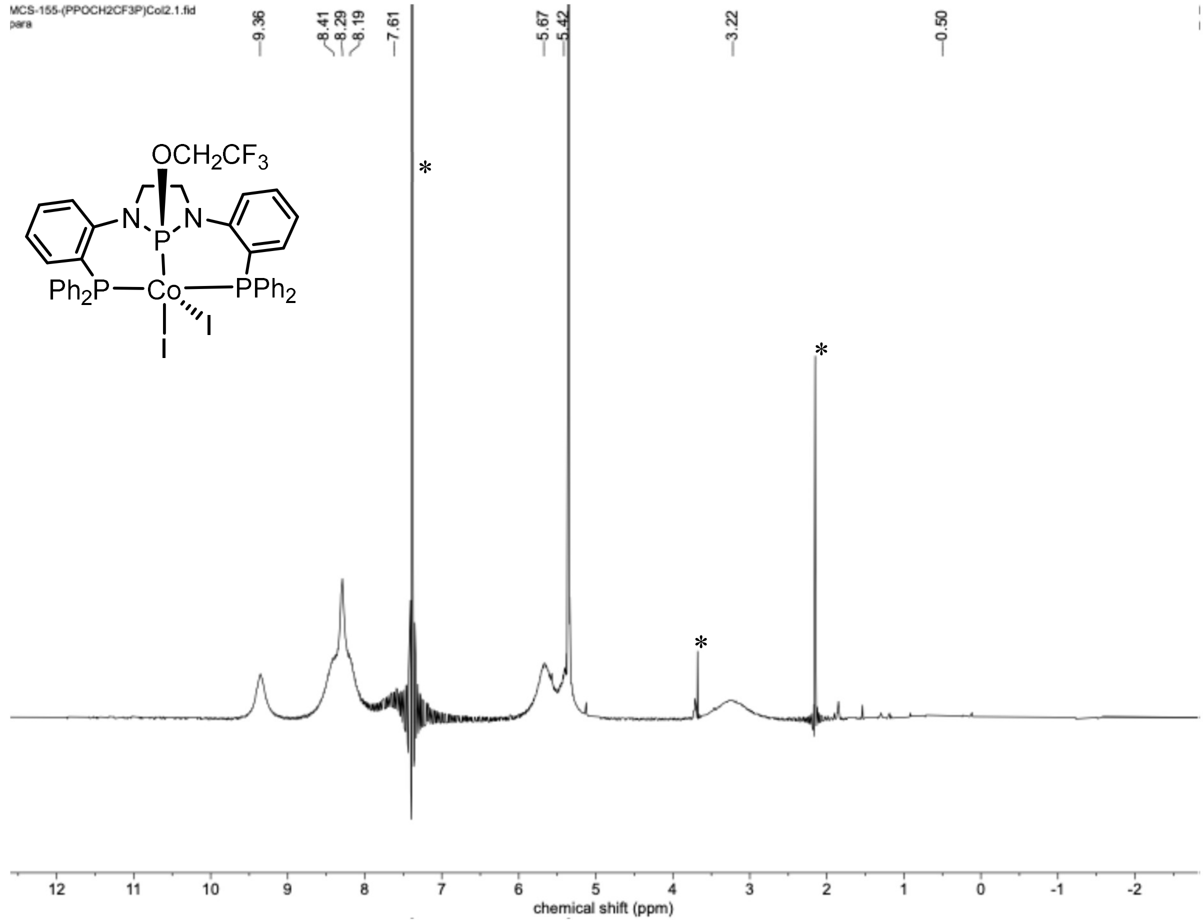

Figure S31: ^{1}H NMR (CD_2Cl_2, 400 MHz) spectrum of ($PP^{OCH2CF3}P$)CoI_2 (**17**). Residual solvent denoted with an asterisk (*).

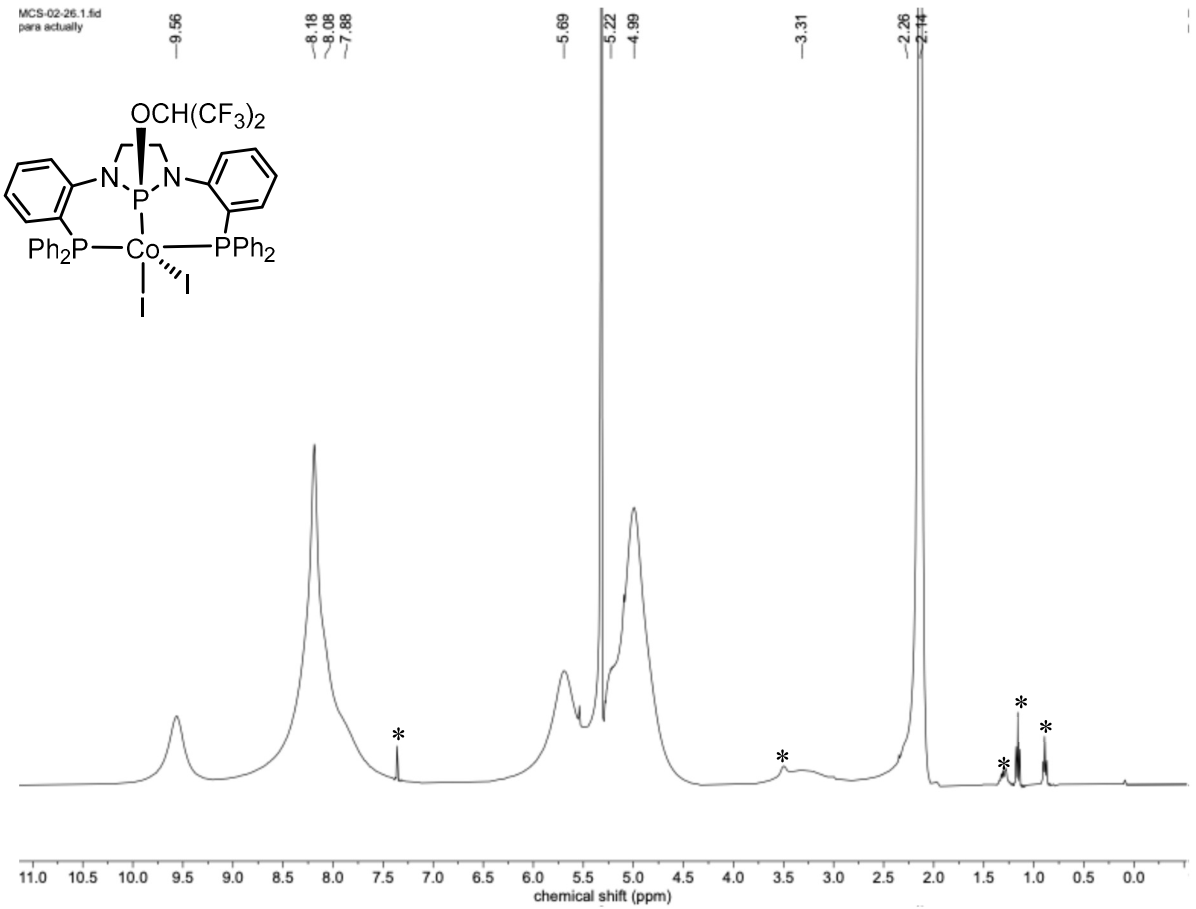

Figure S32: ^{1}H NMR (CD_2Cl_2, 400 MHz) spectrum of ($PP^{OCH(CF3)2}P$)CoI_2 (**18**). Residual solvent denoted with an asterisk (*).

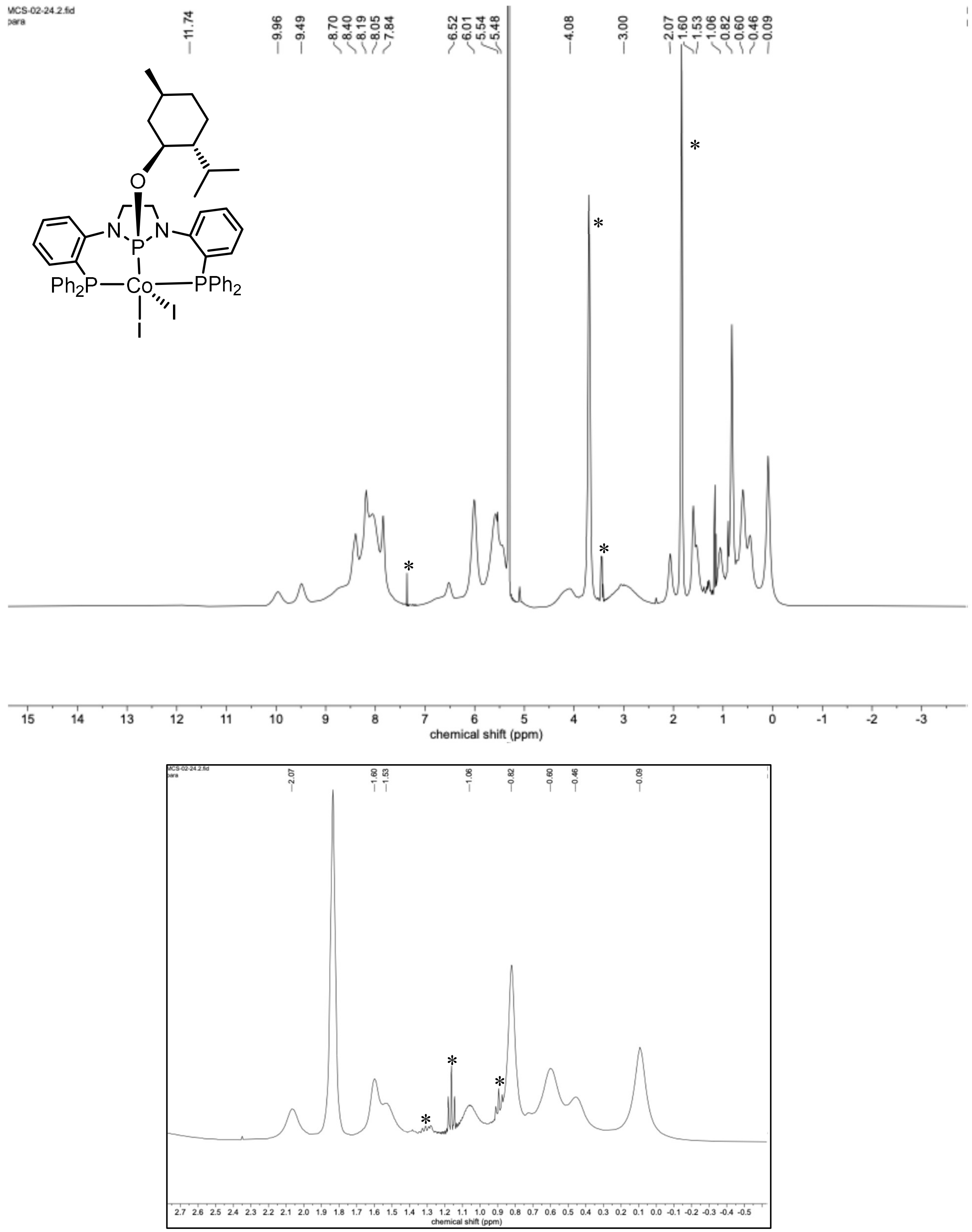

Figure S33: ^{1}H NMR (CD_2Cl_2, 400 MHz) spectrum of (PP^{menthoxide}P)CoI_2 (**19**). Residual solvent denoted with an asterisk (*).

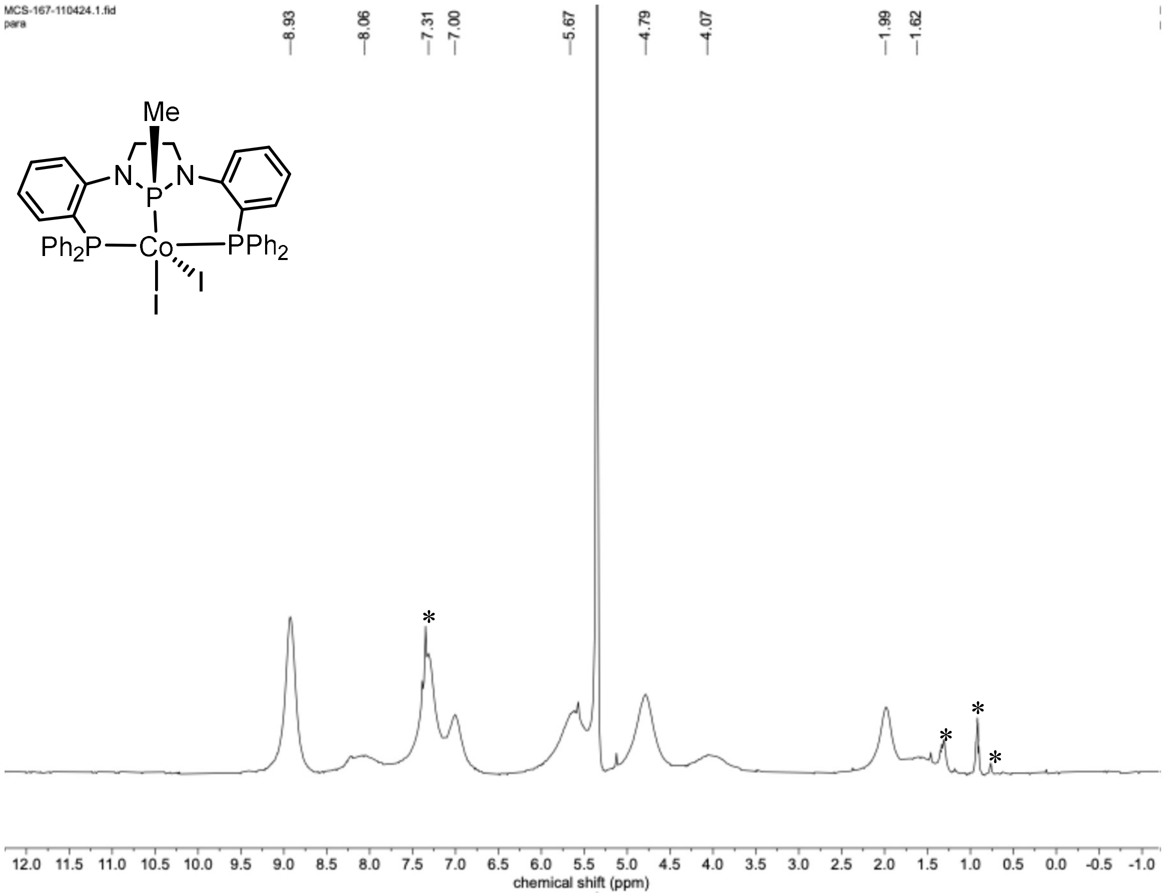

Figure S34: ^{1}H NMR (CD_2Cl_2, 400 MHz) spectrum of $(PP^{Me}P)CoI_2$ (**20**). Residual solvent denoted with an asterisk (*).

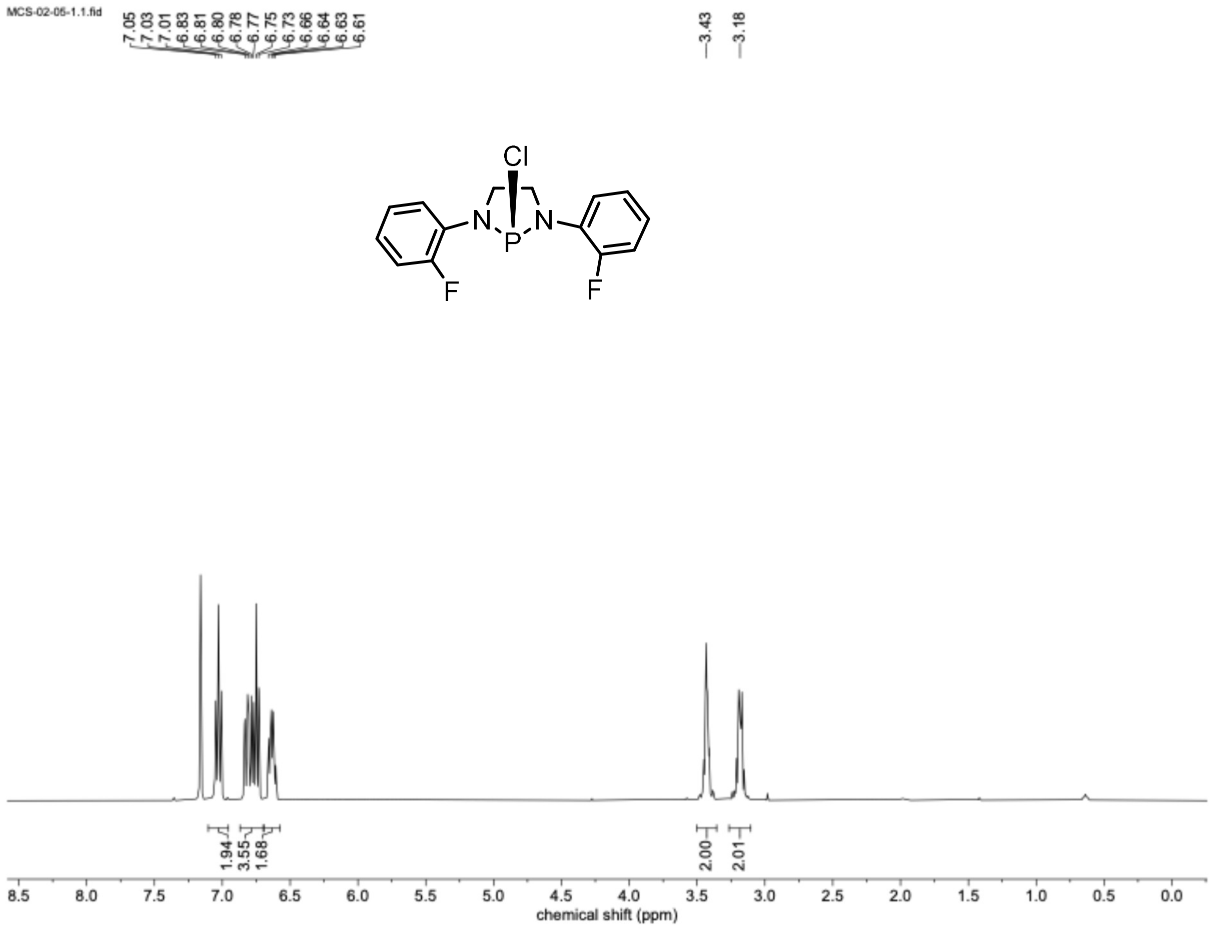

Figure S35: ^{1}H NMR (C_6D_6, 400 MHz) spectrum of ($FP^{Cl}F$) (**30**).

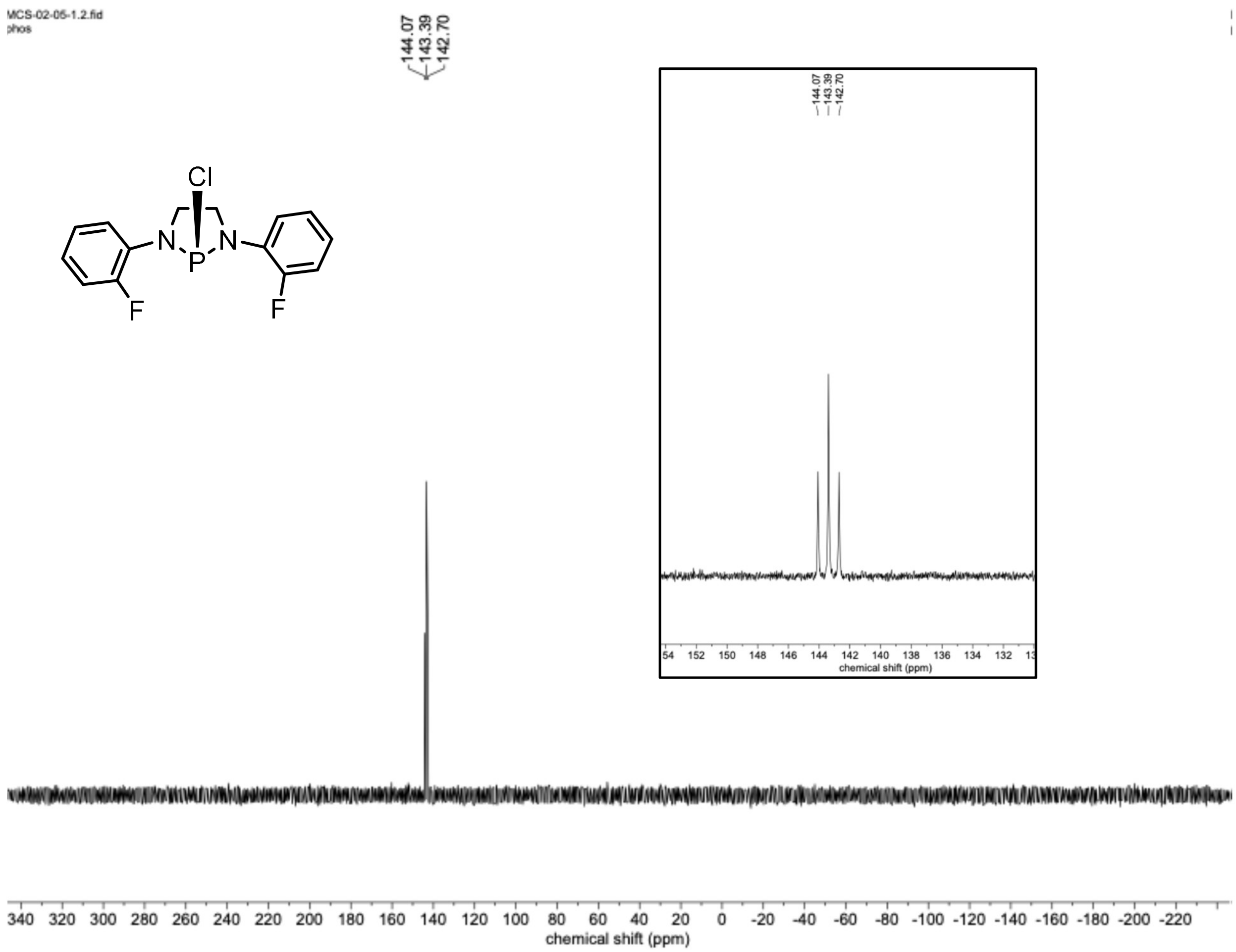

Figure S36: $^{31}P\{^{1}H\}$ NMR (C_6D_6, 162 MHz) spectrum of ($FP^{Cl}F$) (**30**).

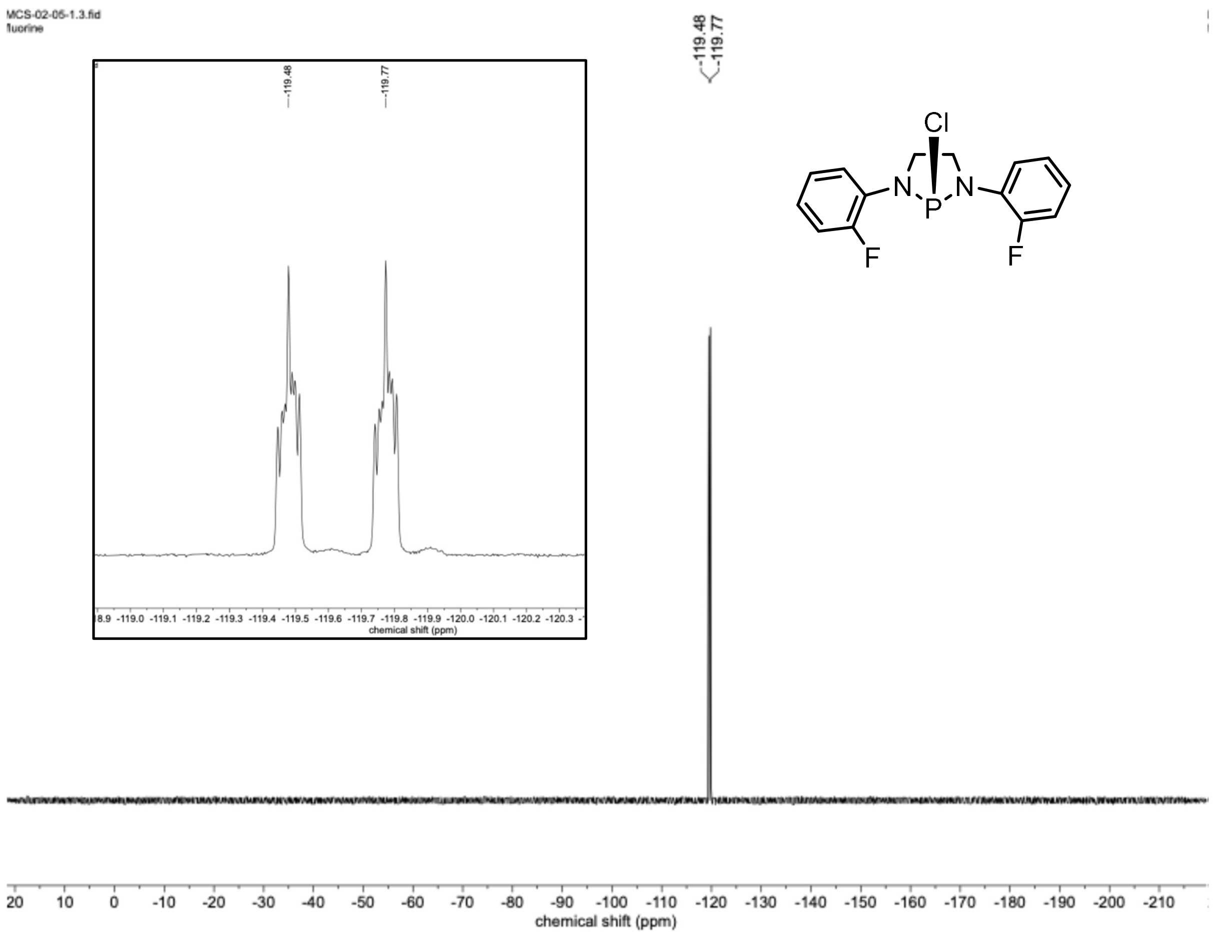

Figure S37: ^{19}F NMR (C_6D_6, 377 MHz) spectrum of ($FP^{Cl}F$) (**30**).

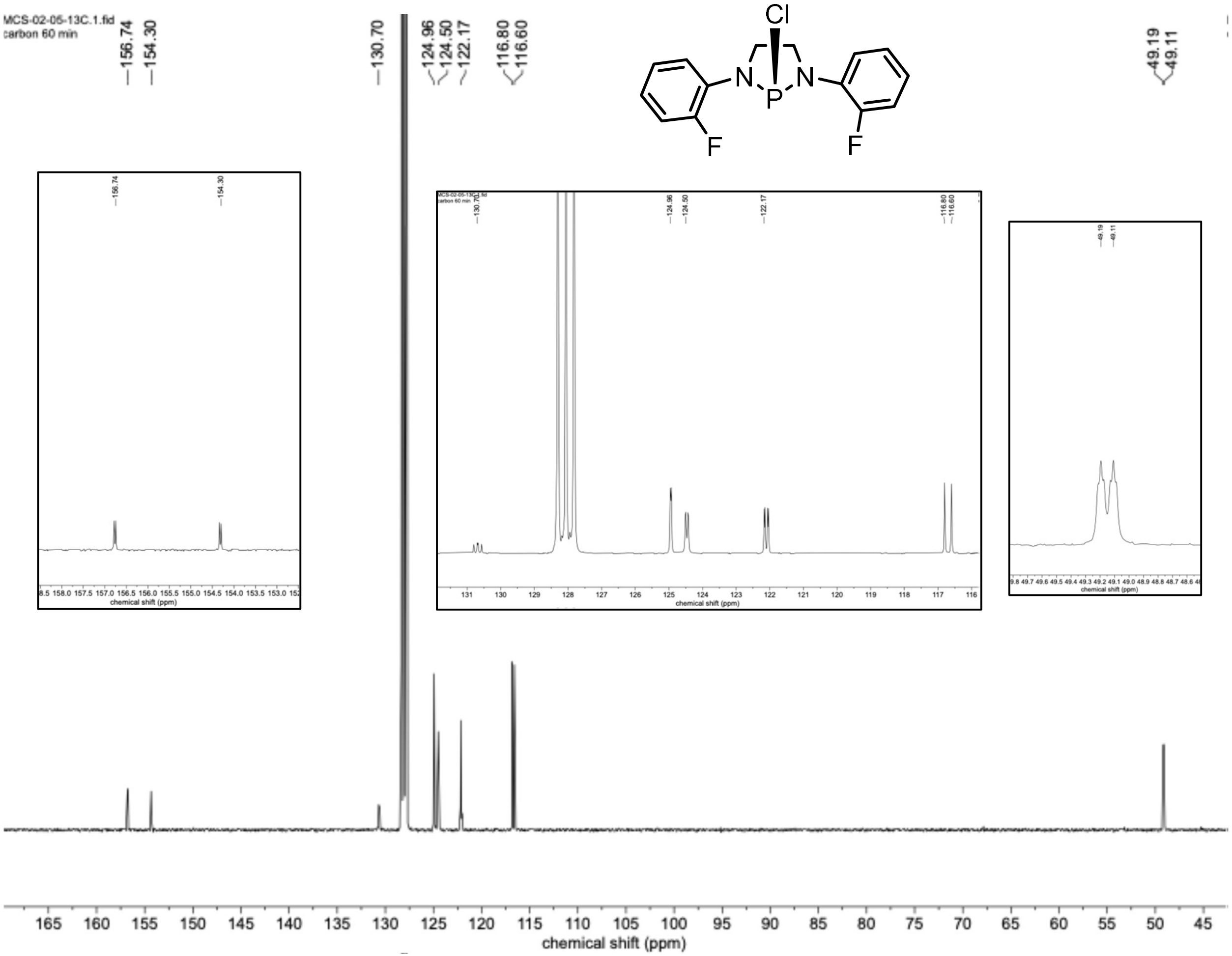

Figure S38: $^{13}C\{^1H\}$ NMR (C_6D_6, 151 MHz) spectrum of ($FP^{Cl}F$) (**30**).

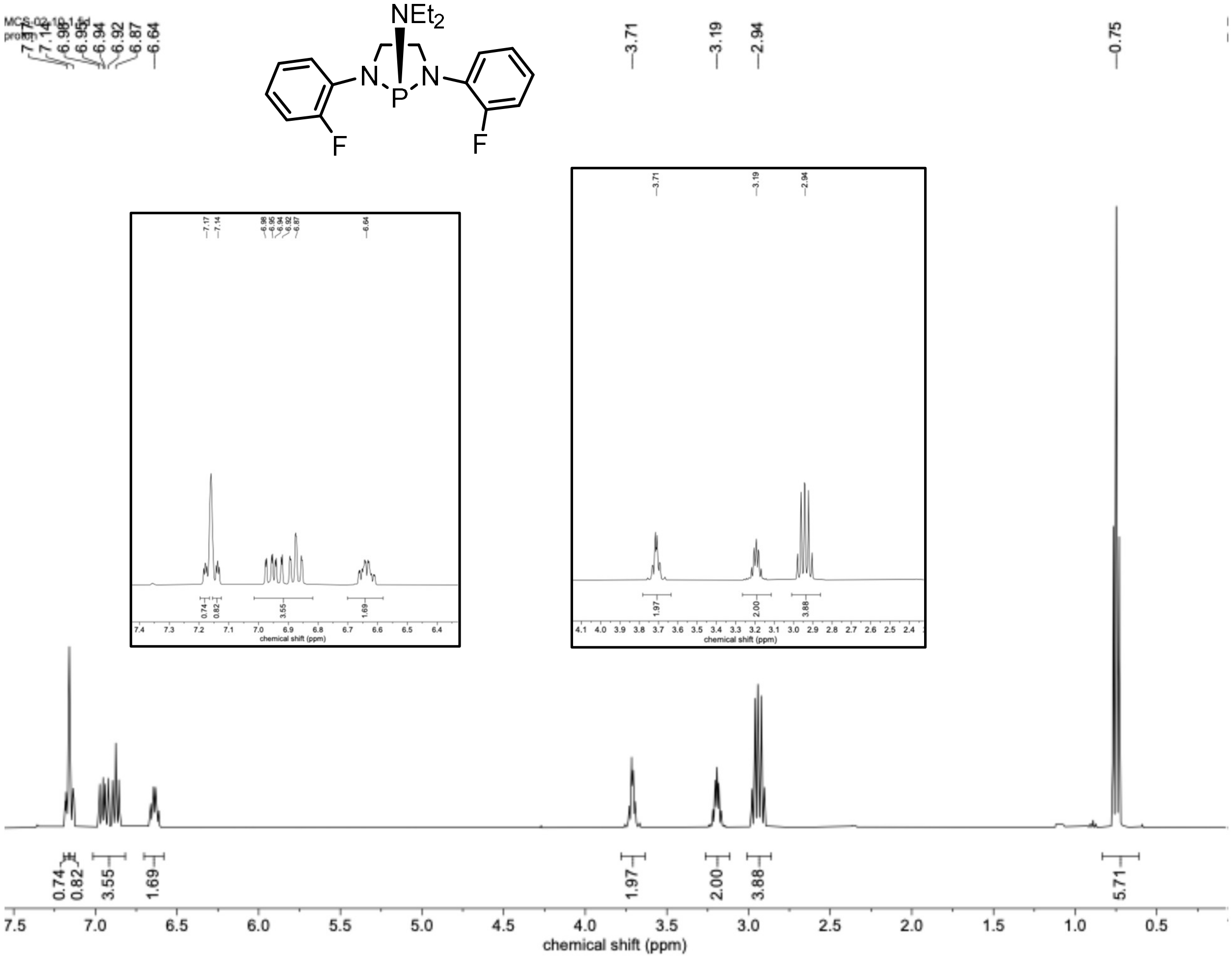

Figure S39: ^{1}H NMR (C_6D_6, 400 MHz) spectrum of ($FP^{NEt2}F$) (**31**).

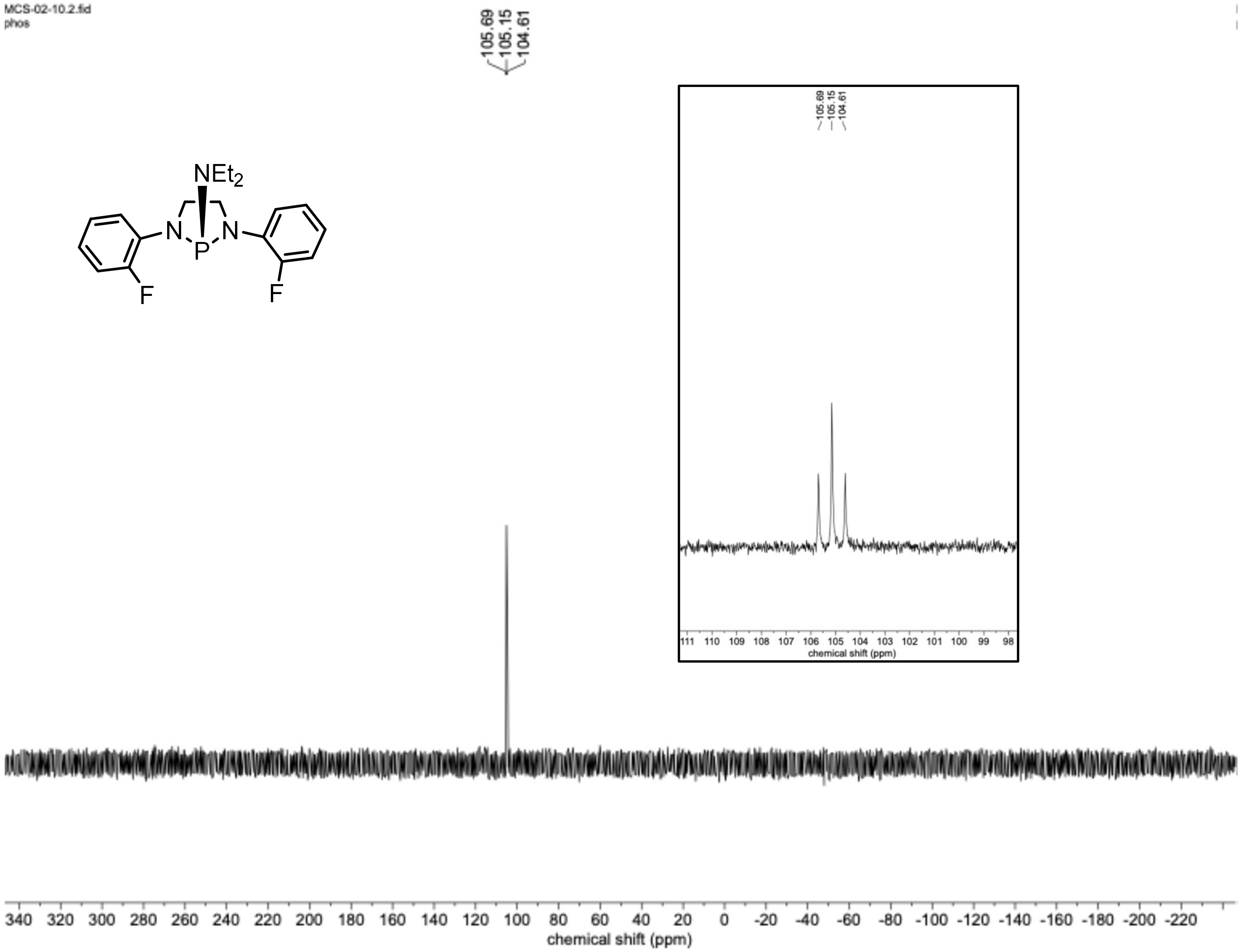

Figure S40: $^{31}P\{^{1}H\}$ NMR (C_6D_6, 162 MHz) spectrum of ($FP^{NEt2}F$) (**31**).

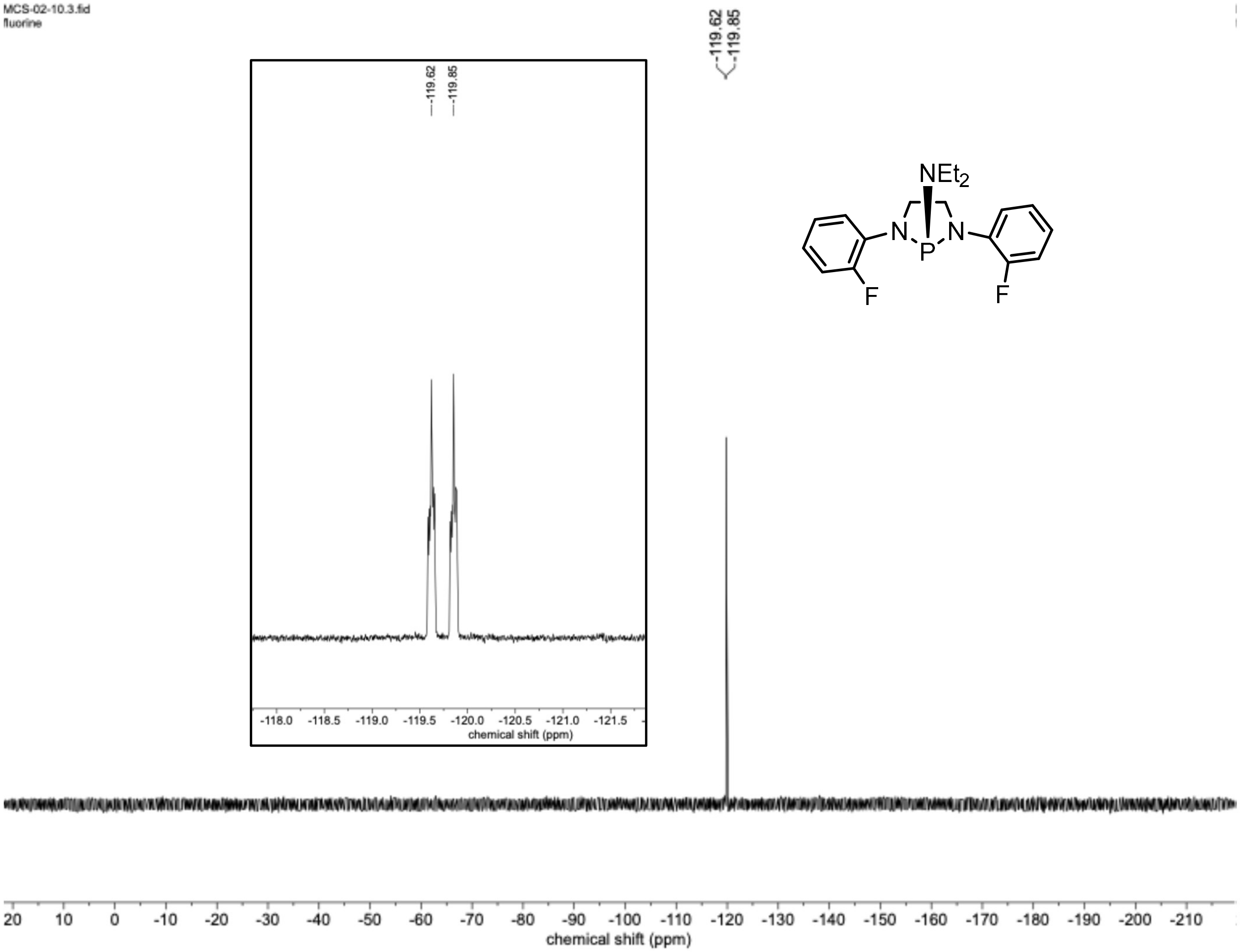

Figure S41: ^{19}F NMR (C_6D_6, 377 MHz) spectrum of ($FP^{NEt2}F$) (**31**).

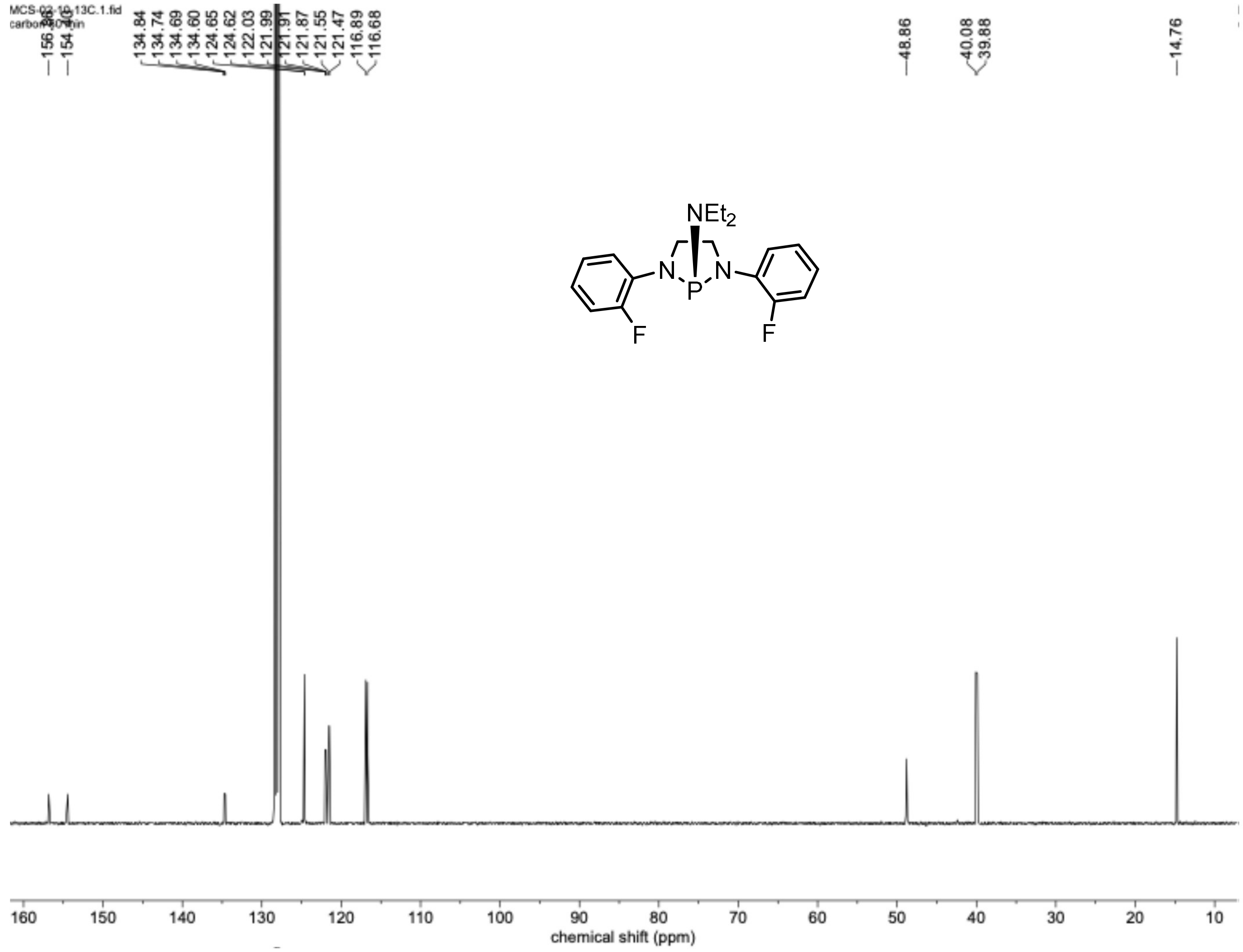

Figure S42: $^{13}C\{^{1}H\}$ NMR (C_6D_6, 151 MHz) spectrum of ($FP^{NEt2}F$) (**31**).

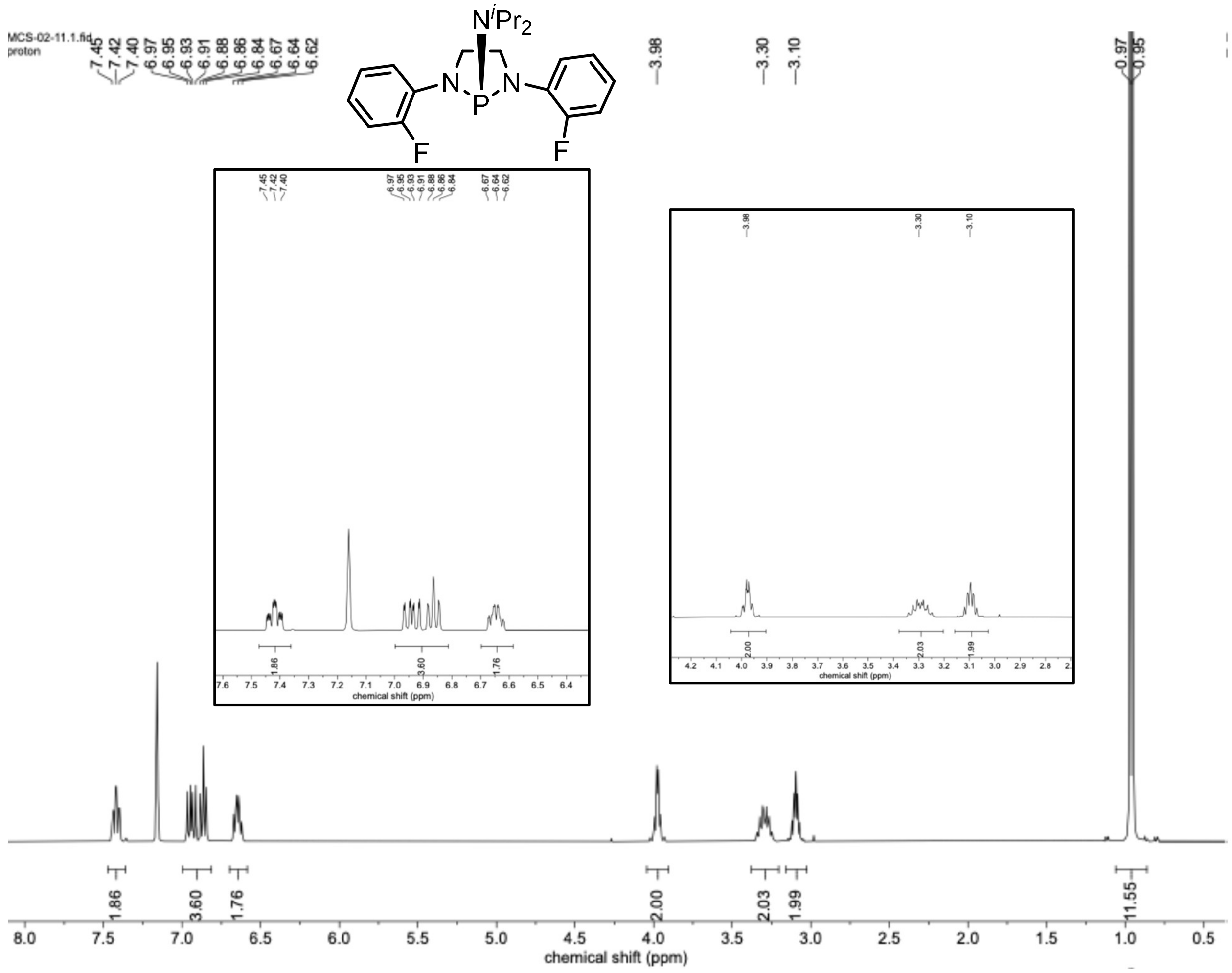

Figure S43: ^{1}H NMR (C_6D_6, 400 MHz) spectrum of ($FP^{NiPr2}F$) (**32**).

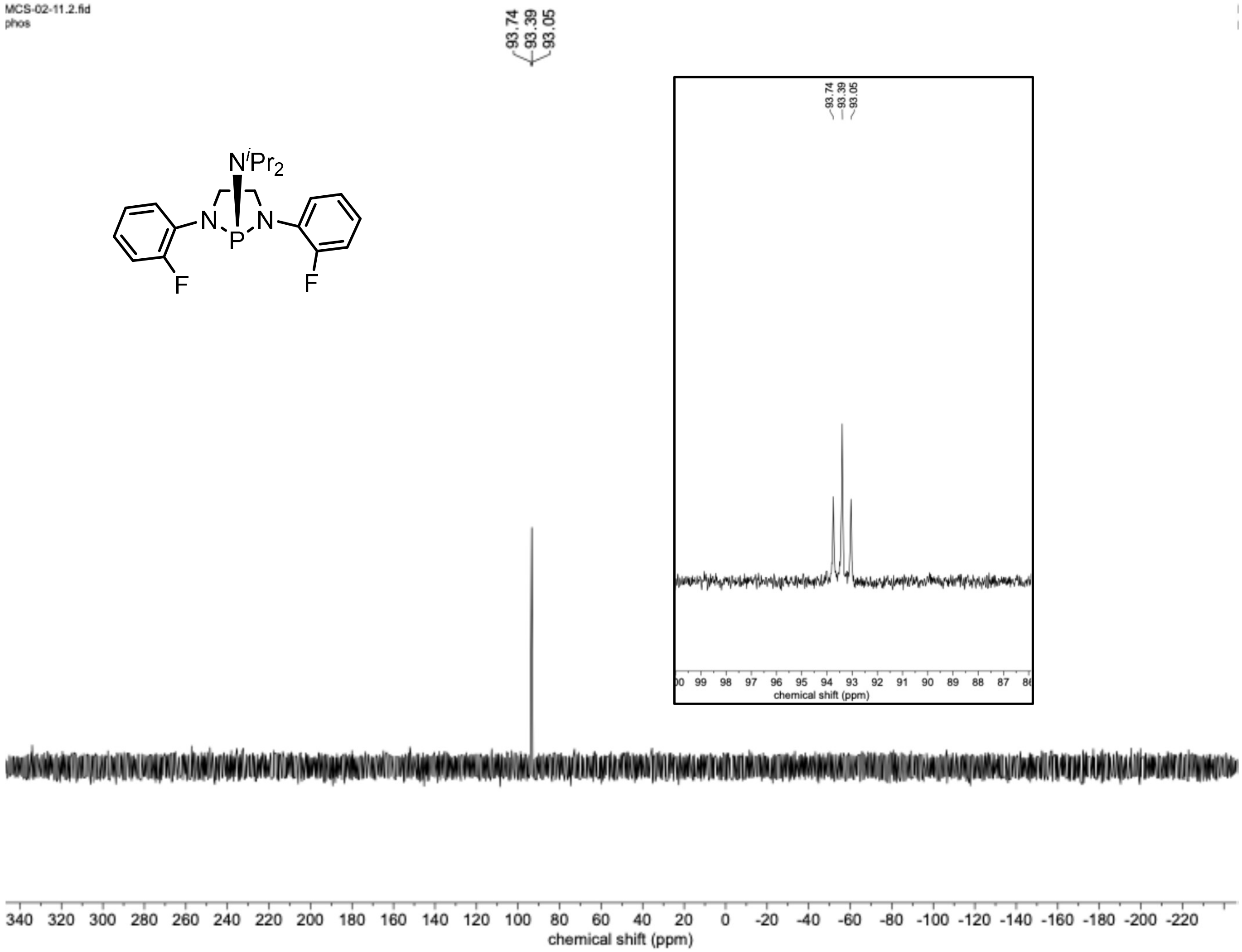

Figure S44: $^{31}P\{^1H\}$ NMR (C_6D_6, 162 MHz) spectrum of ($FP^{N\textit{i}Pr2}F$) (**32**).

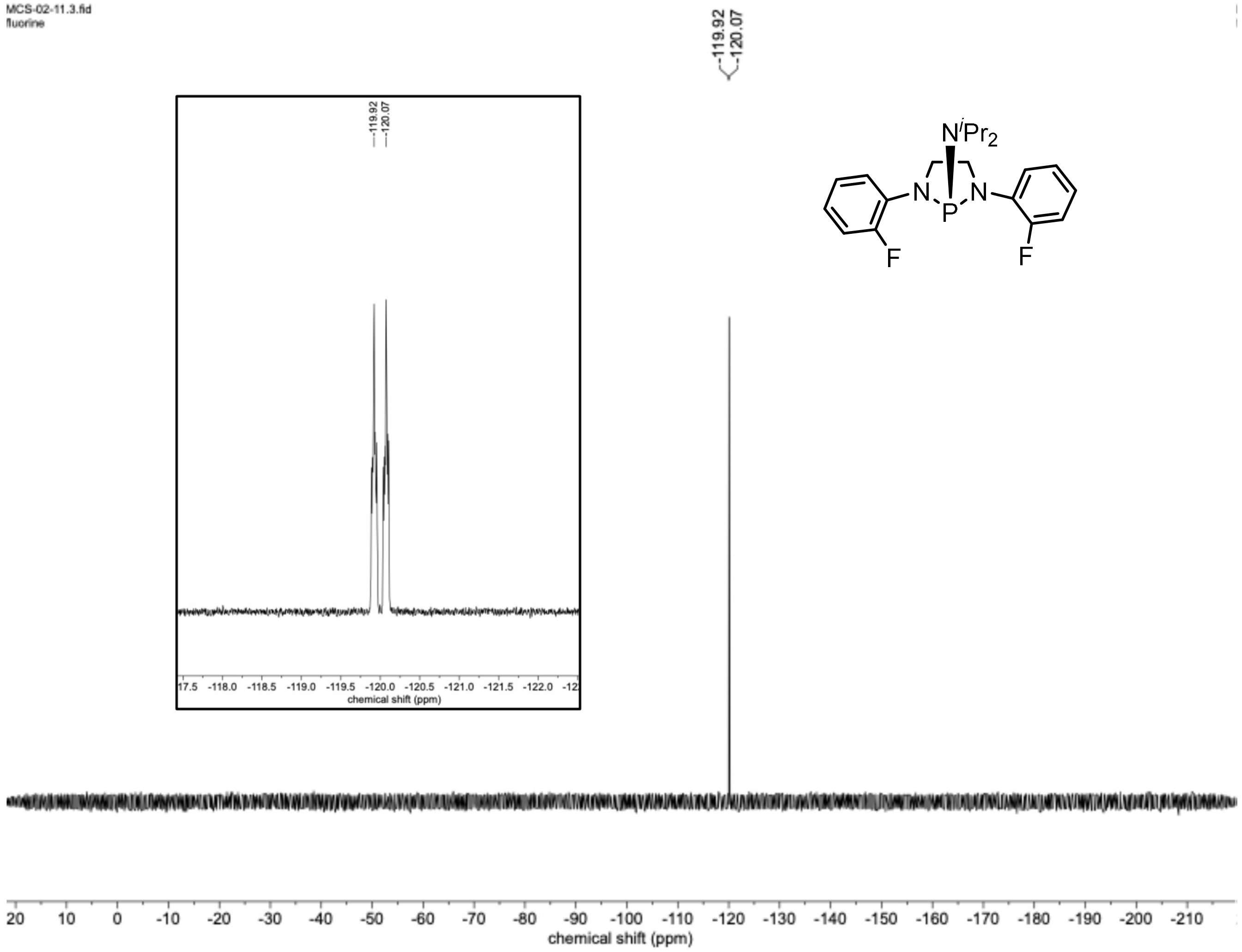

Figure S45: ^{19}F NMR (C_6D_6, 377 MHz) spectrum of ($FP^{N\mathit{i}Pr2}F$) (**32**).

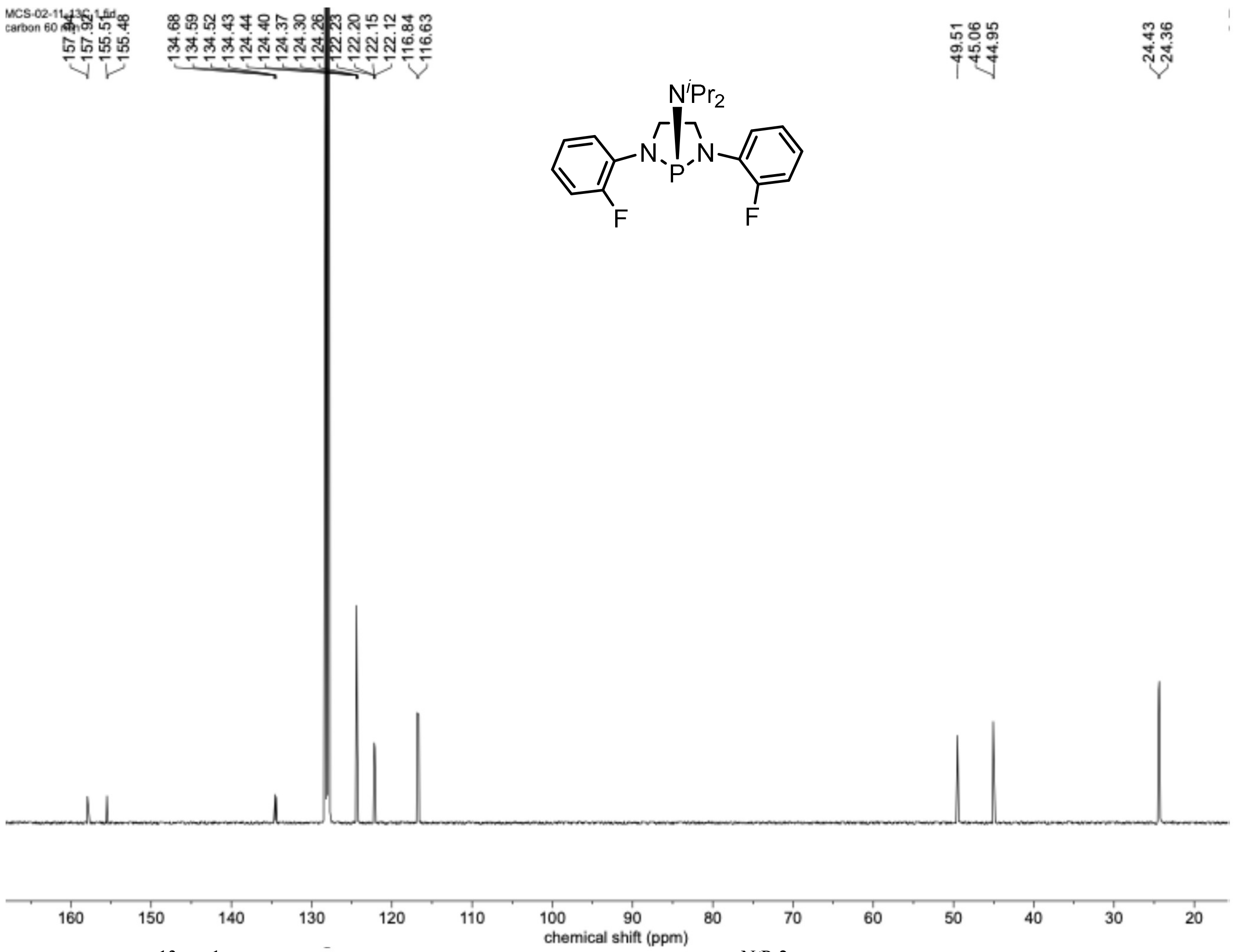

Figure S46: $^{13}C\{^1H\}$ NMR (C_6D_6, 151 MHz) spectrum of ($FP^{N\mathit{i}Pr2}F$) (**32**).

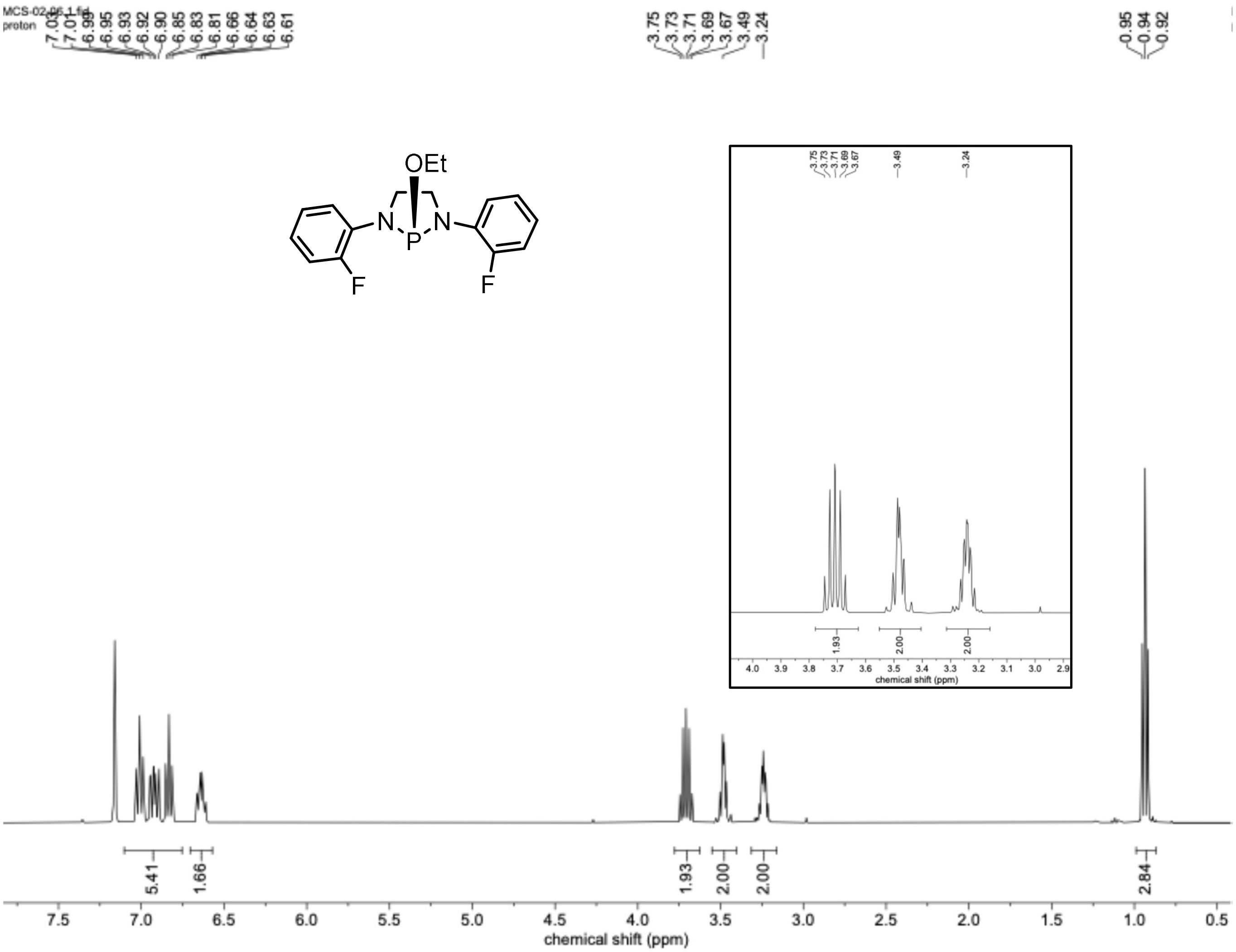

Figure S47: ^{1}H NMR (C_6D_6, 400 MHz) spectrum of (FP^{OEt}F) (**33**).

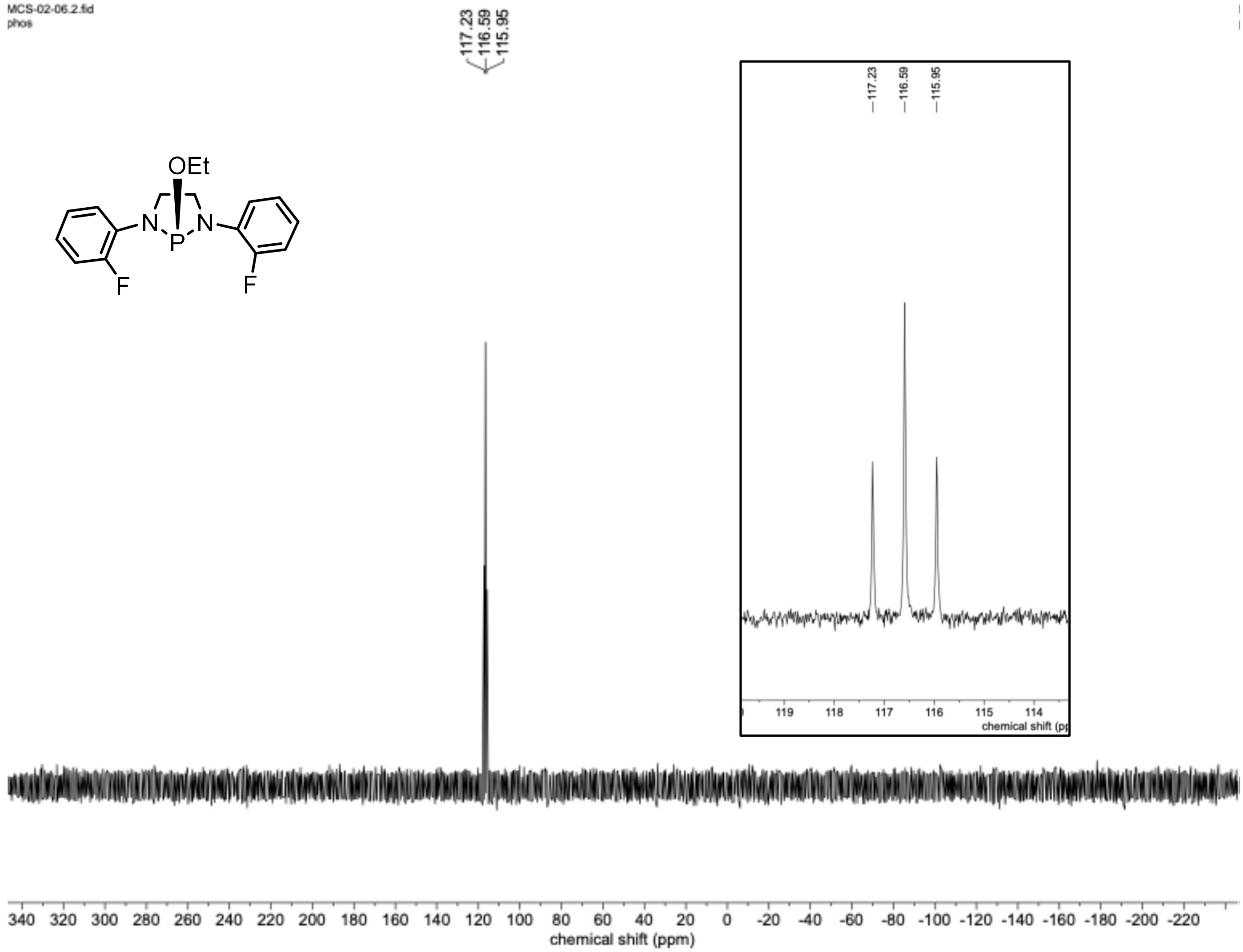

Figure S48: $^{31}P\{^{1}H\}$ NMR (C_6D_6, 162 MHz) spectrum of ($FP^{OEt}F$) (**33**).

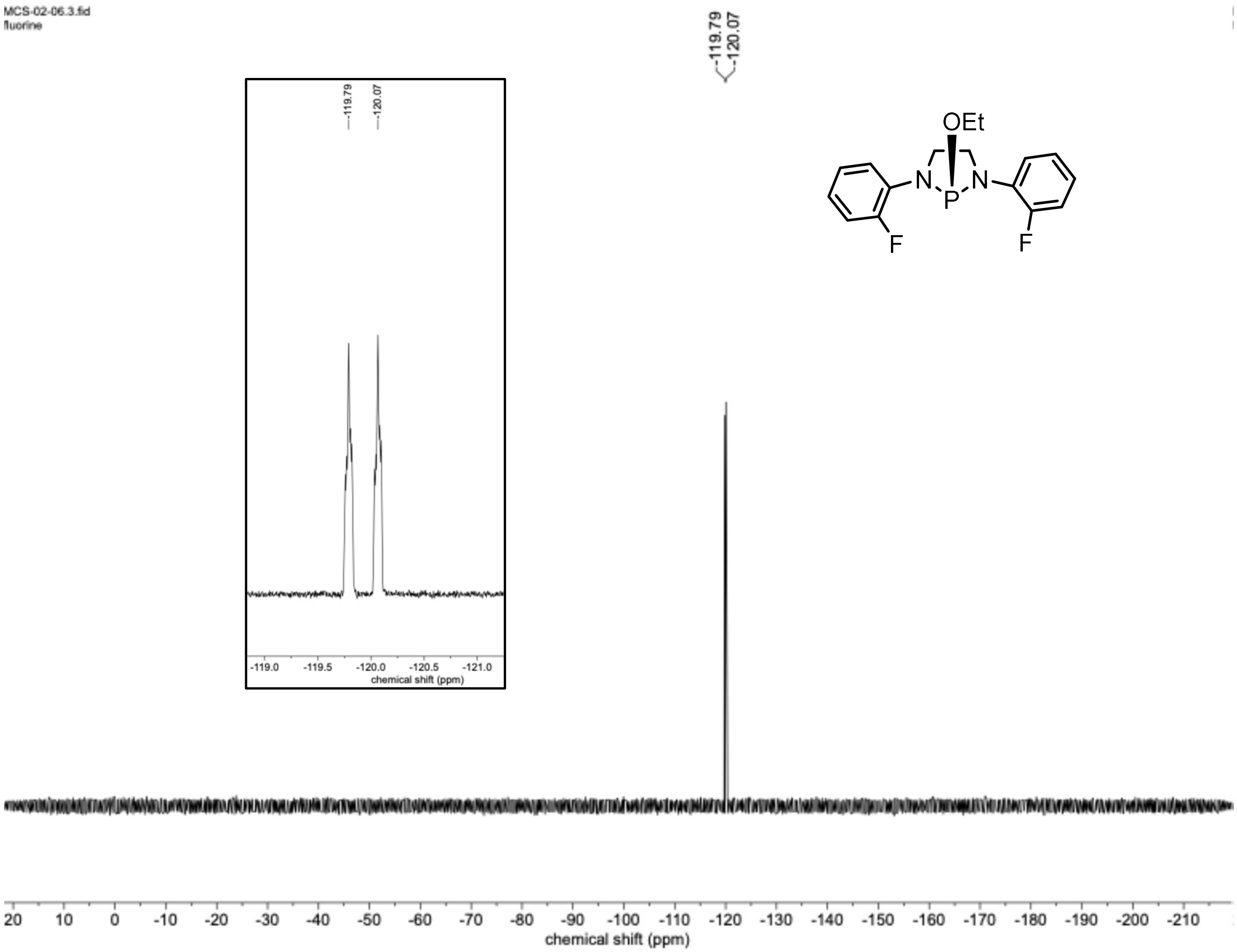

Figure S49: ^{19}F NMR (C_6D_6, 377 MHz) spectrum of ($FP^{OEt}F$) (**33**).

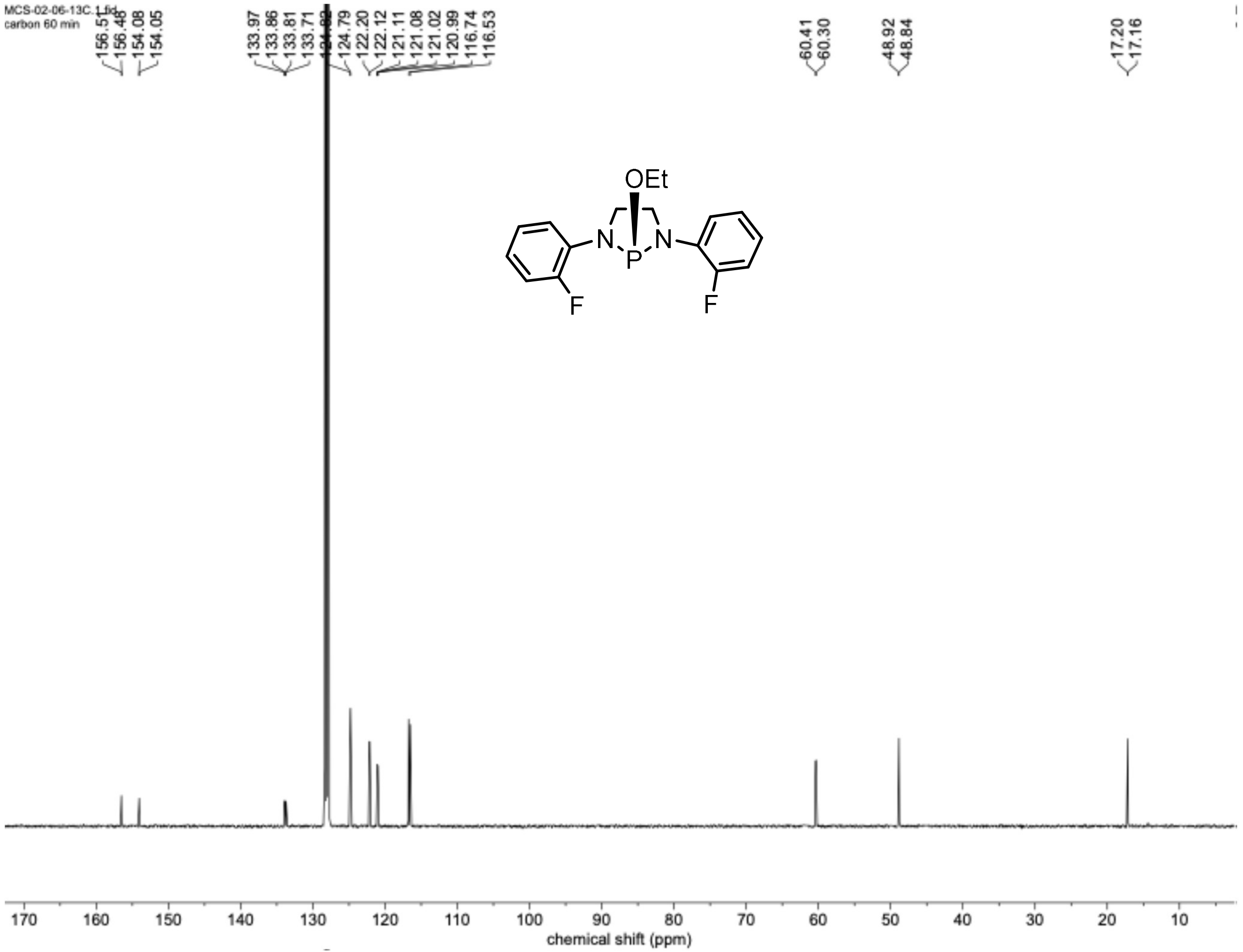

Figure S50: $^{13}C\{^1H\}$ NMR (C_6D_6, 151 MHz) spectrum of ($FP^{OEt}F$) (**33**).

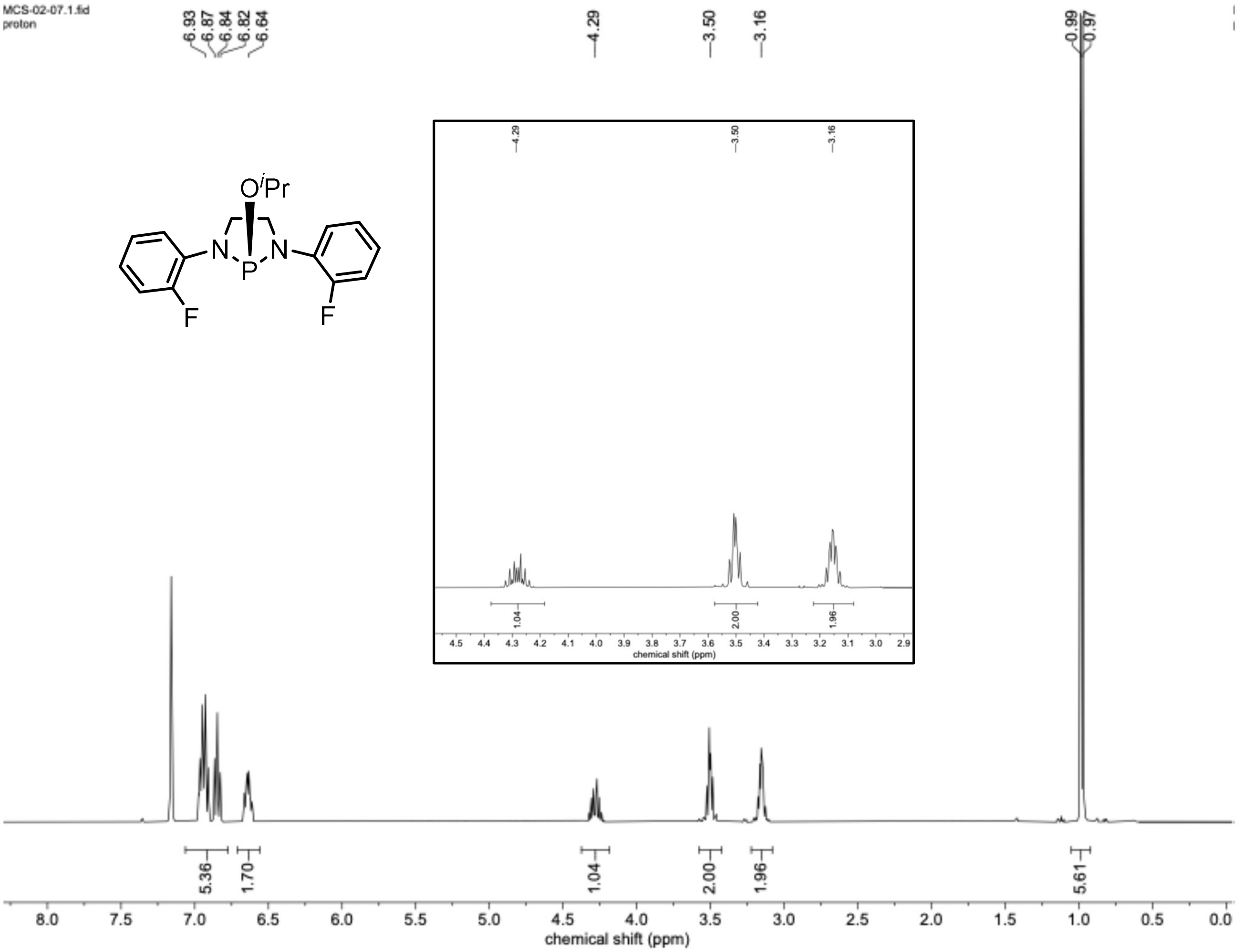

Figure S51: ^{1}H NMR (C_6D_6, 400 MHz) spectrum of (FP^{OiPr}F) (**34**).

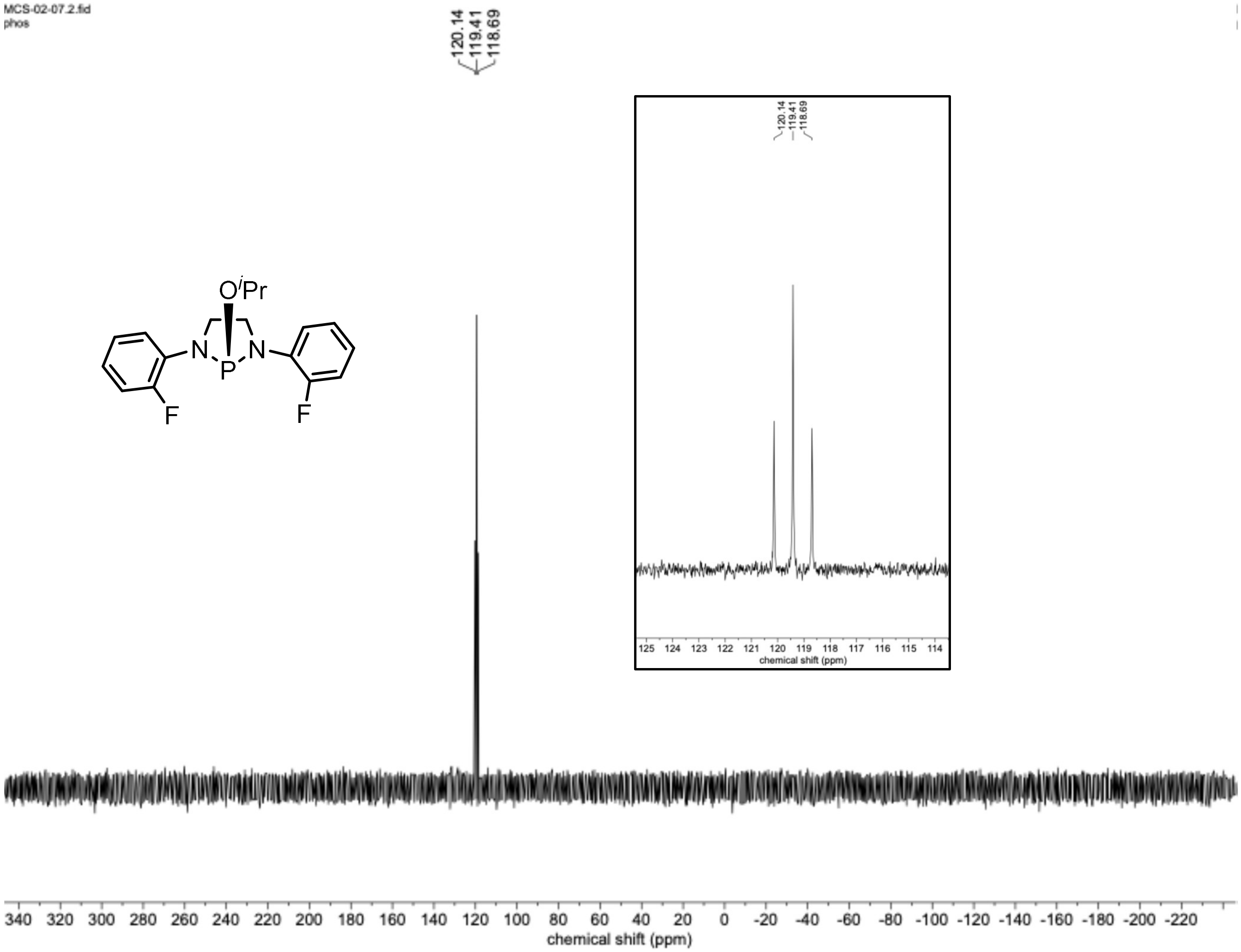

Figure S52: $^{31}P\{^1H\}$ NMR (C_6D_6, 162 MHz) spectrum of ($FP^{O\textit{i}Pr}F$) (**34**).

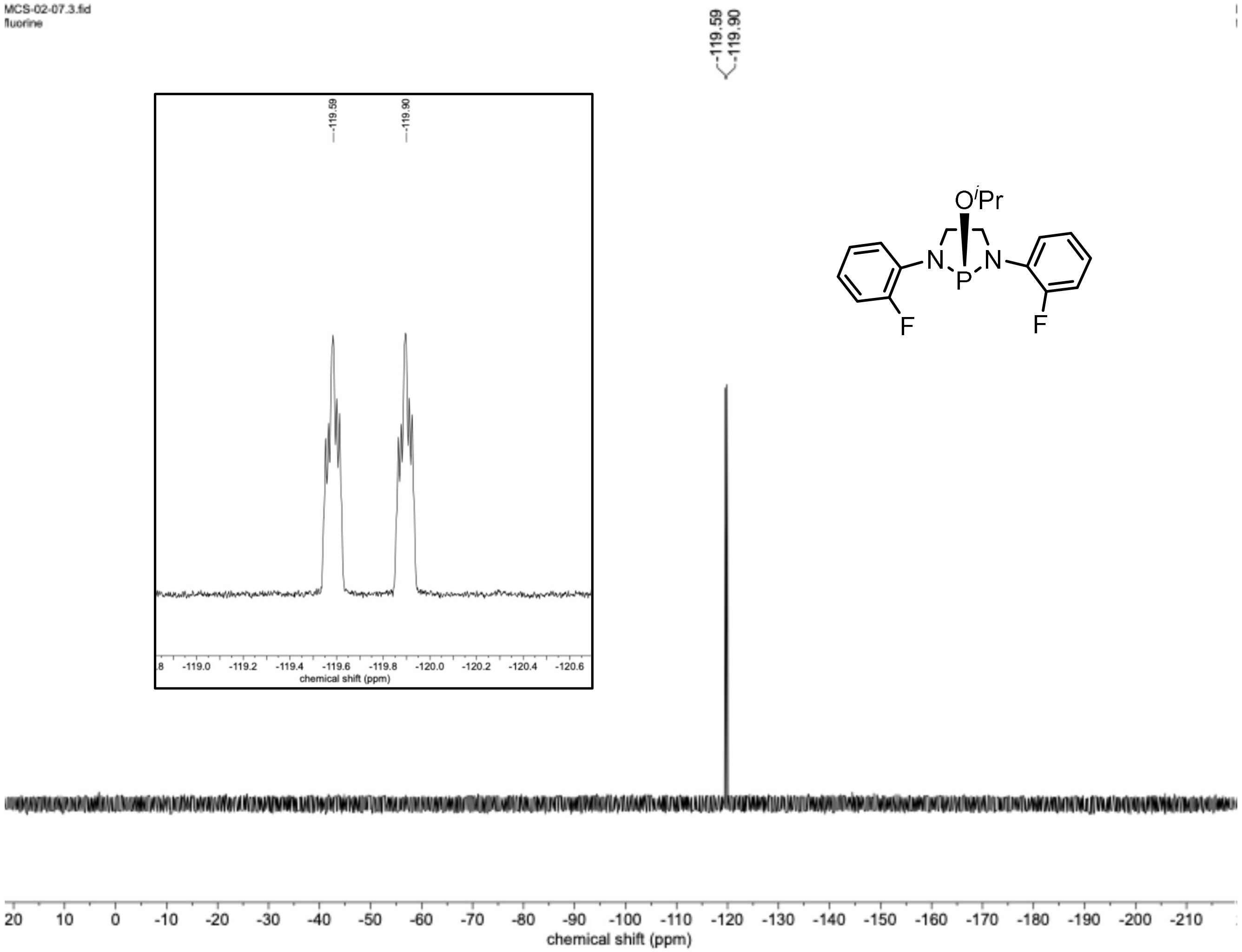

Figure S53: ^{19}F NMR (C_6D_6, 377 MHz) spectrum of (FP$^{\mathrm{O}i\mathrm{Pr}}$F) (**34**).

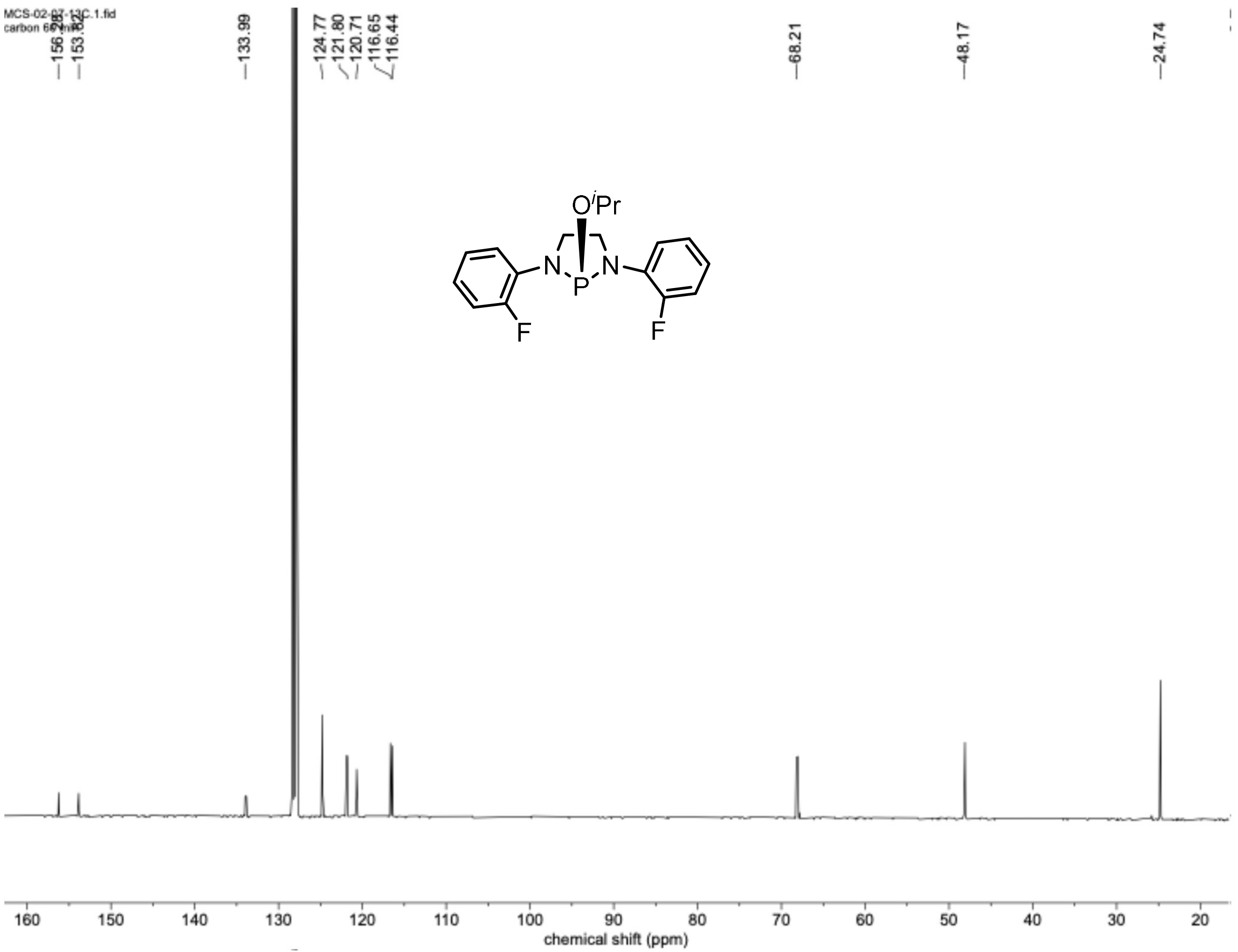

Figure S54: $^{13}C\{^{1}H\}$ NMR (C_6D_6, 151 MHz) spectrum of ($FP^{O\mathit{i}Pr}F$) (**34**).

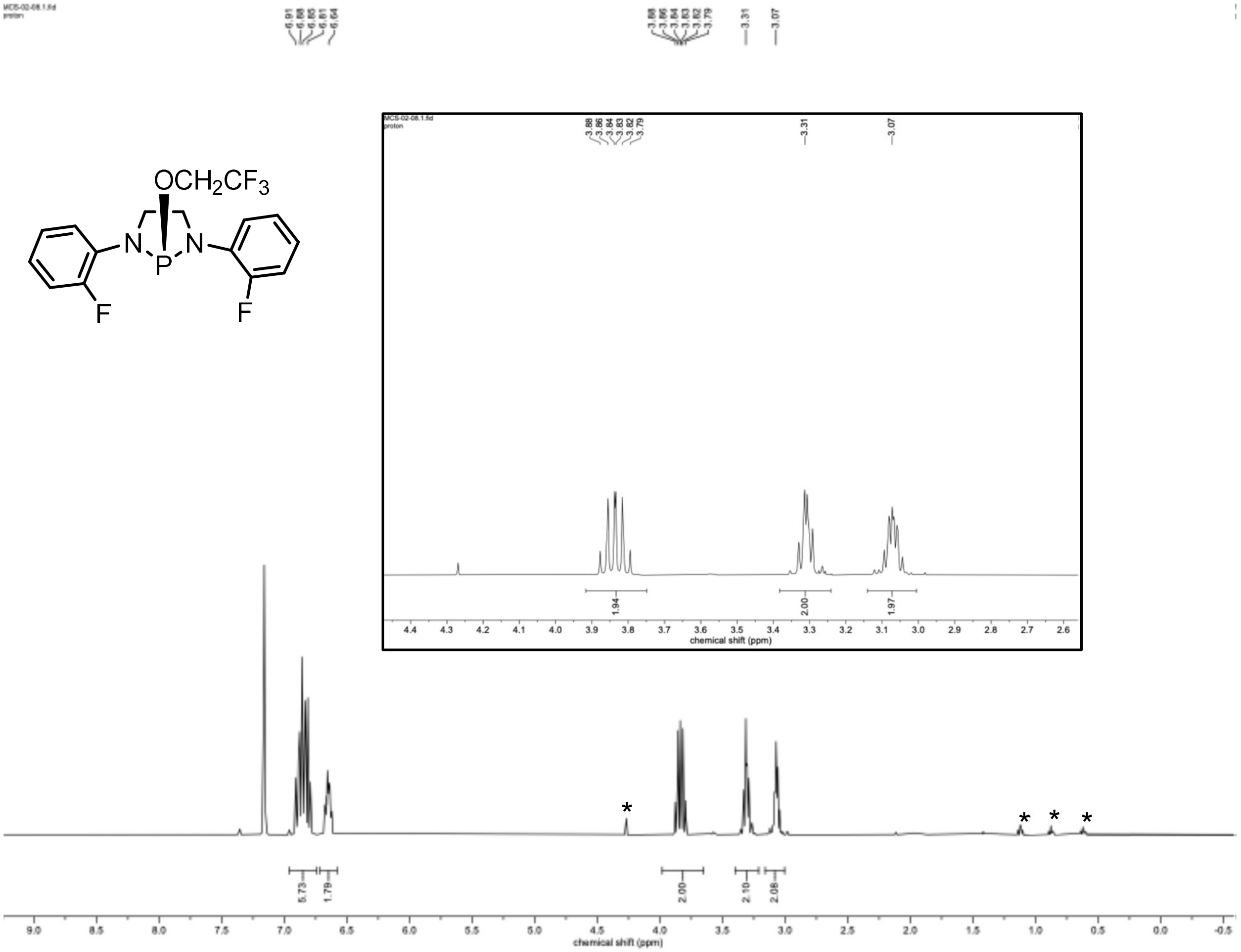

Figure S55: ^{1}H NMR (C_6D_6, 400 MHz) spectrum of ($FP^{OCH2CF3}F$) (**35**). Residual solvent denoted with an asterisk (*).

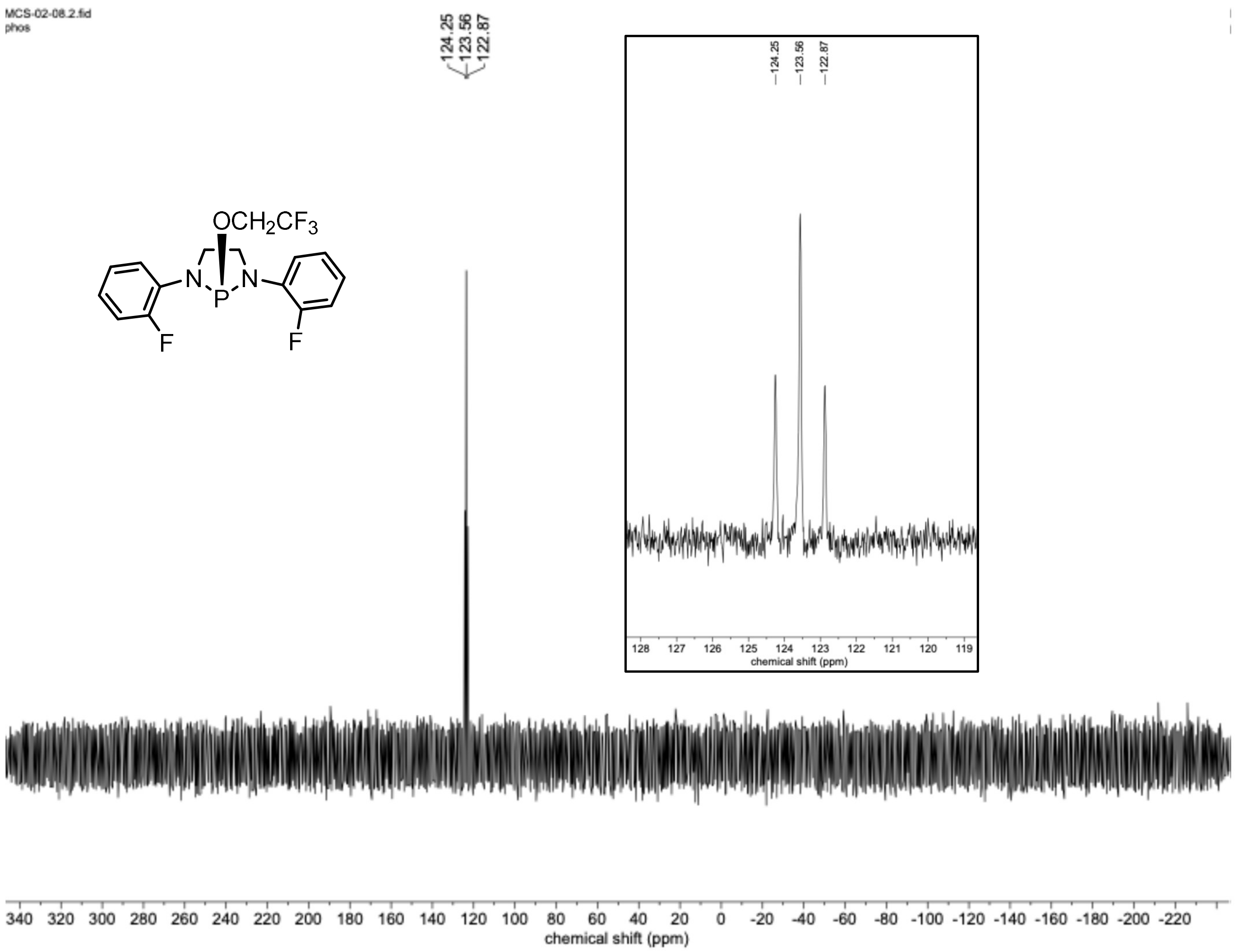

Figure S56: $^{31}P\{^1H\}$ NMR (C_6D_6, 162 MHz) spectrum of ($FP^{OCH2CF3}F$) (**35**).

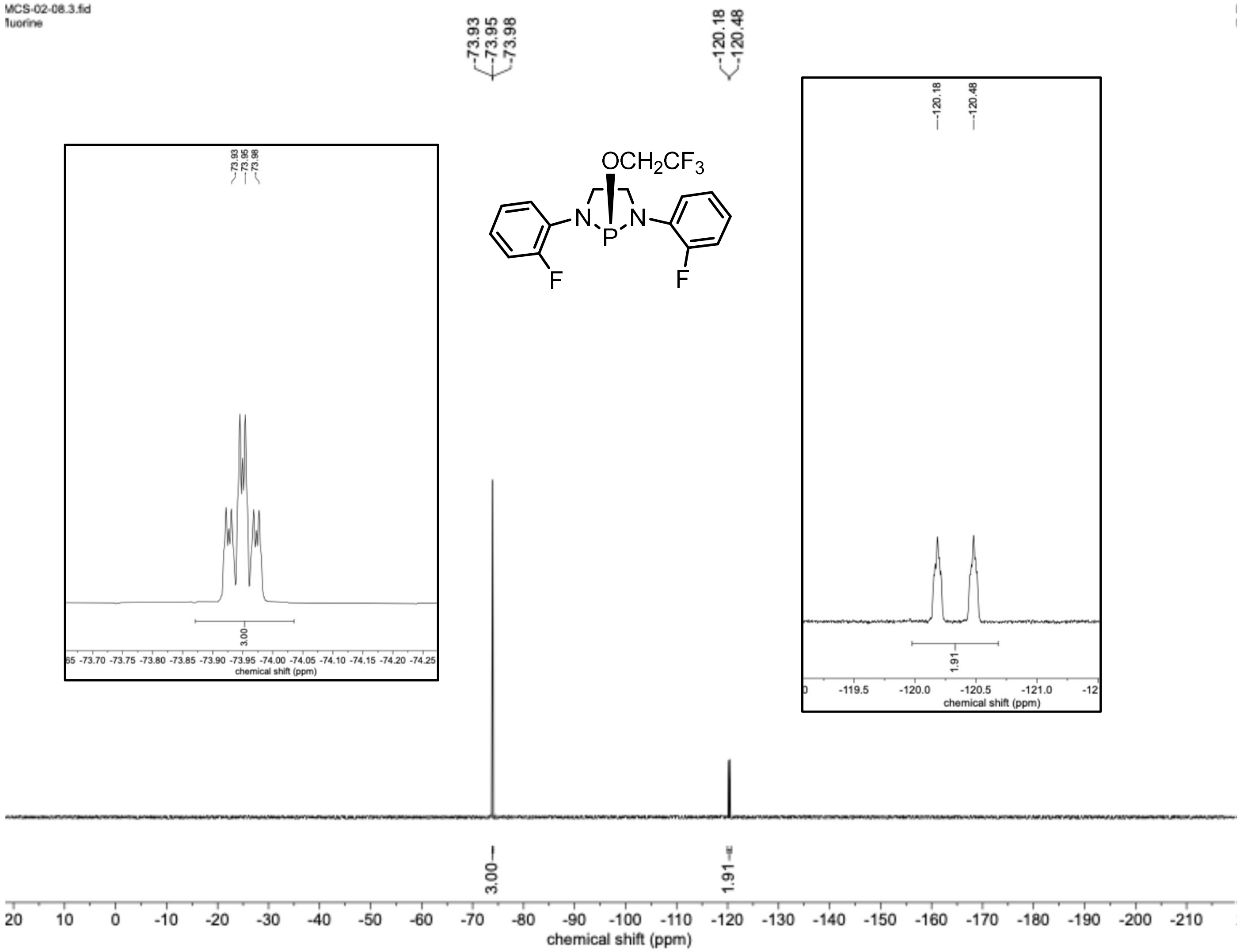

Figure S57: ^{19}F NMR (C_6D_6, 377 MHz) spectrum of ($FP^{OCH2CF3}F$) (**35**).

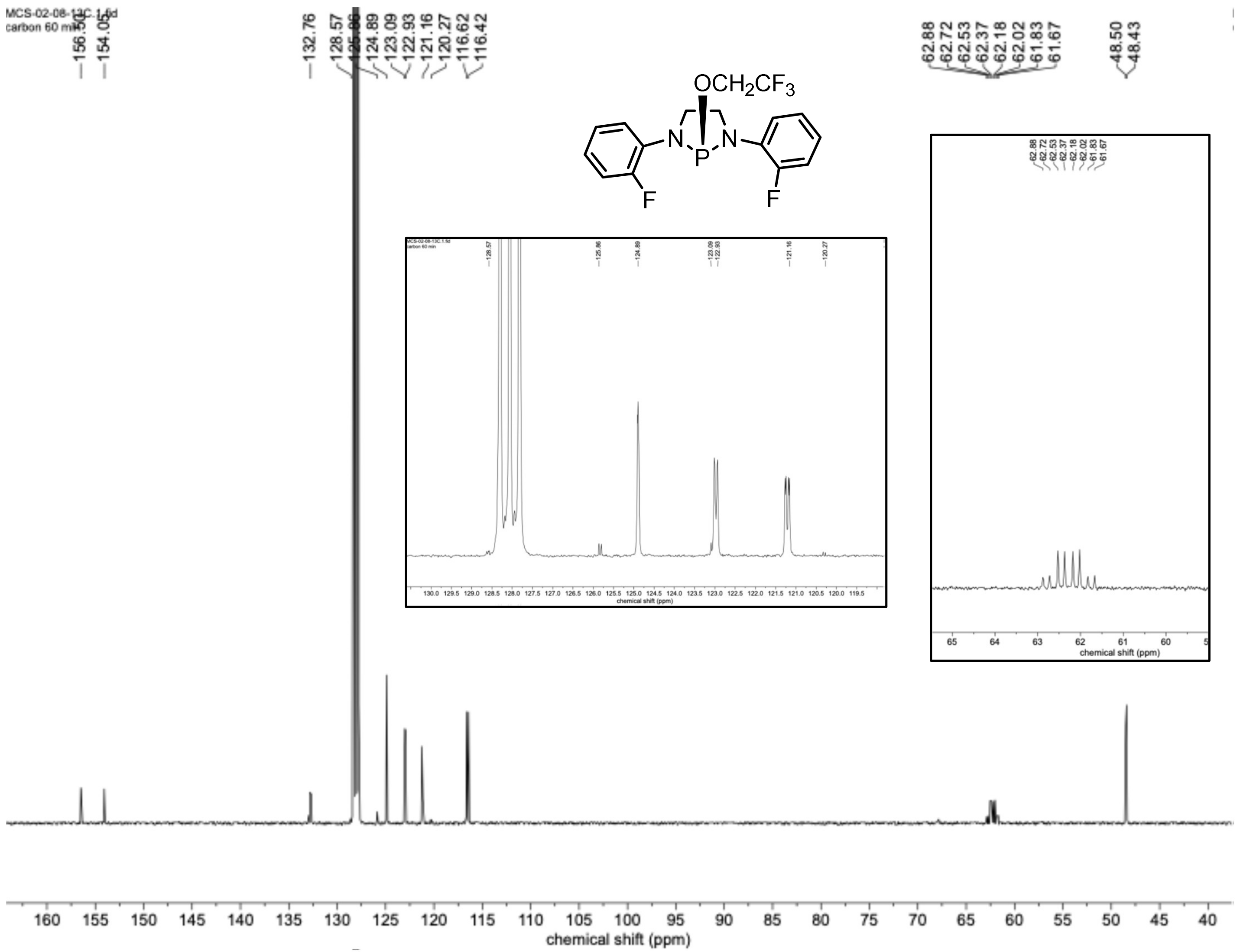

Figure S58: $^{13}C\{^1H\}$ NMR (C_6D_6, 151 MHz) spectrum of ($FP^{OCH2CF3}F$) (**35**).

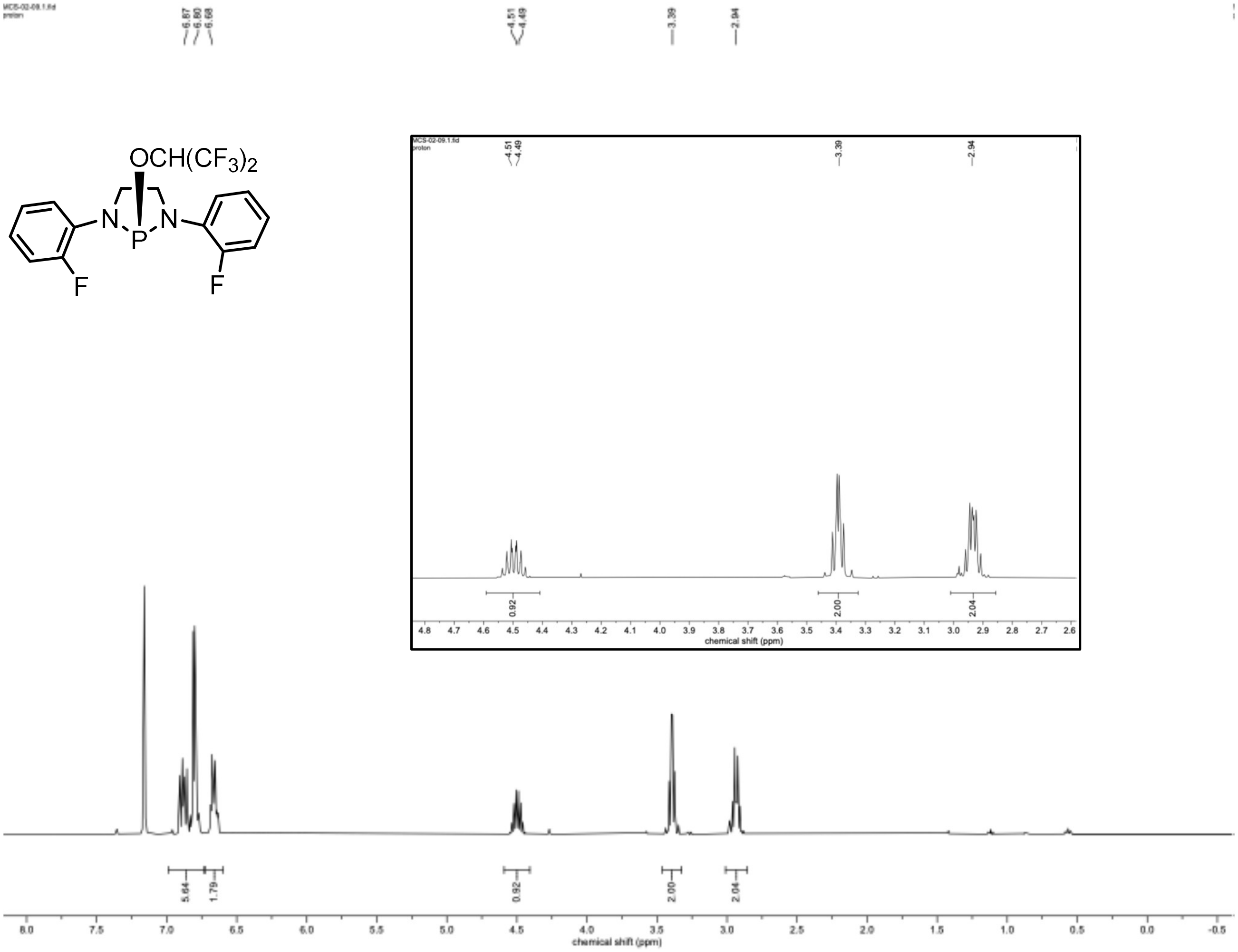

Figure S59: ^{1}H NMR (C_6D_6, 400 MHz) spectrum of ($FP^{OCH(CF3)2}F$) (**36**).

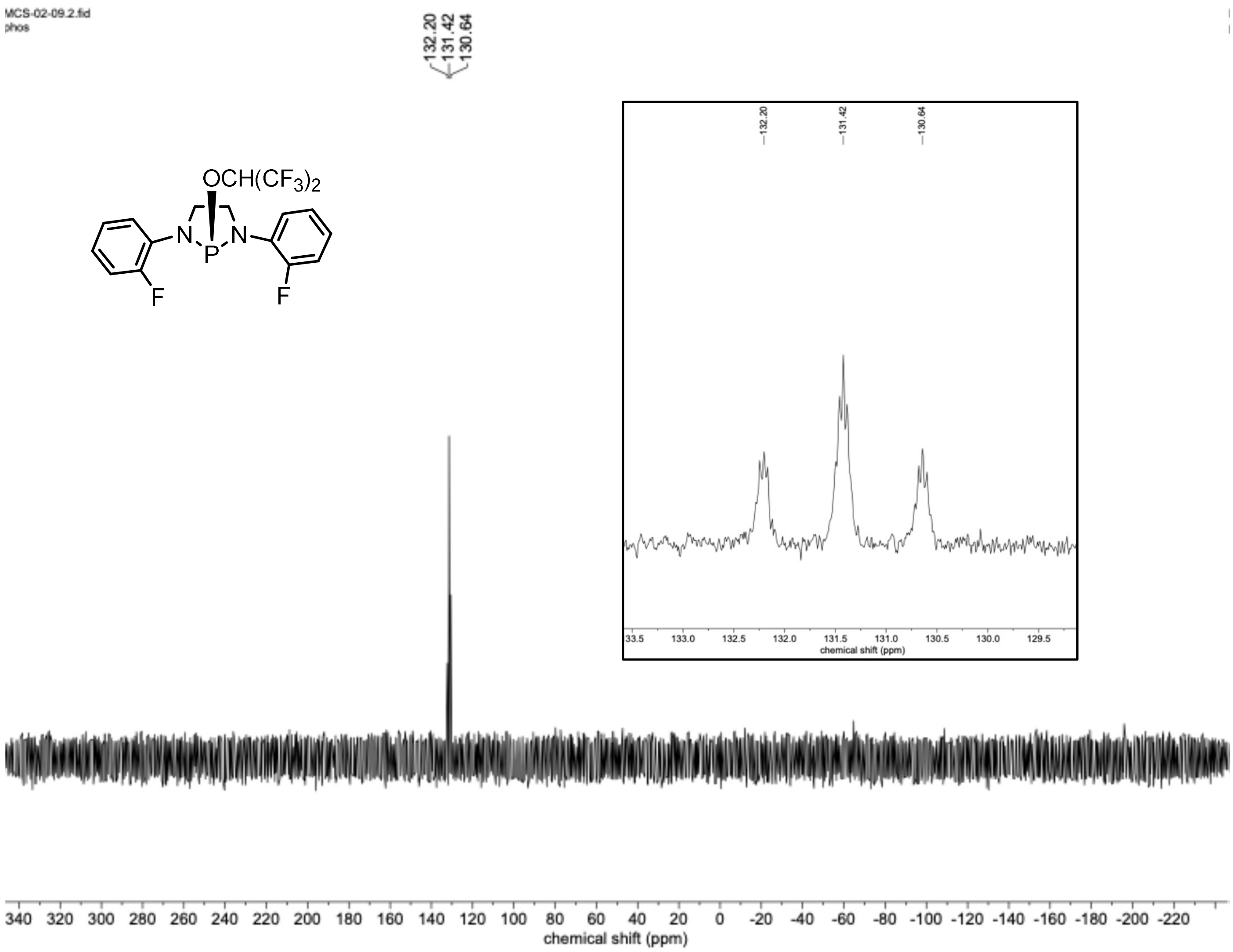

Figure S60: $^{31}P\{^{1}H\}$ NMR (C_6D_6, 162 MHz) spectrum of ($FP^{OCH(CF3)2}F$) (**36**).

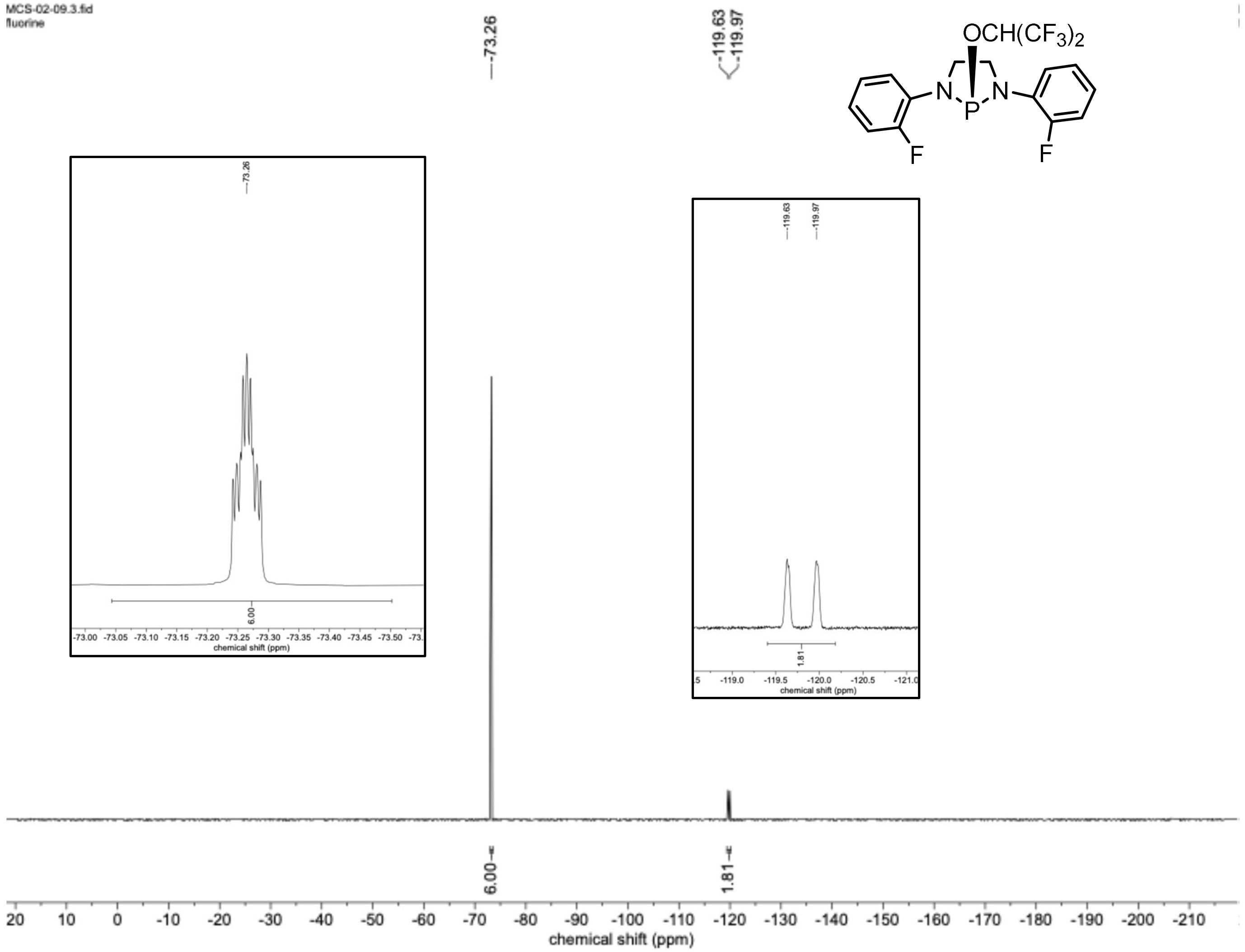

Figure S61: ^{19}F NMR (C_6D_6, 377 MHz) spectrum of (FP$^{OCH(CF3)2}$F) (**36**).

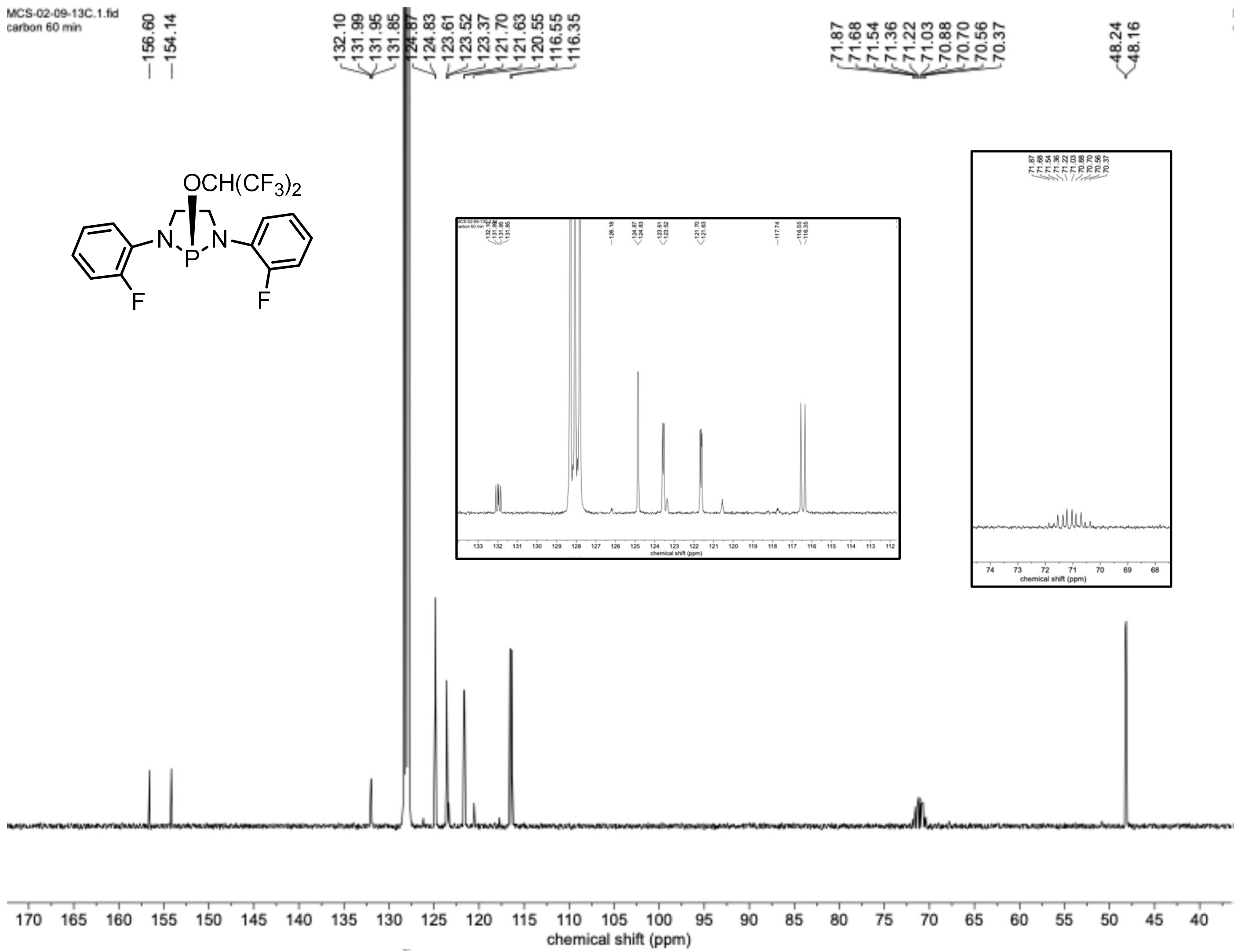

Figure S62: $^{13}C\{^{1}H\}$ NMR (C_6D_6, 151 MHz) spectrum of ($FP^{OCH(CF3)2}F$) (**36**).

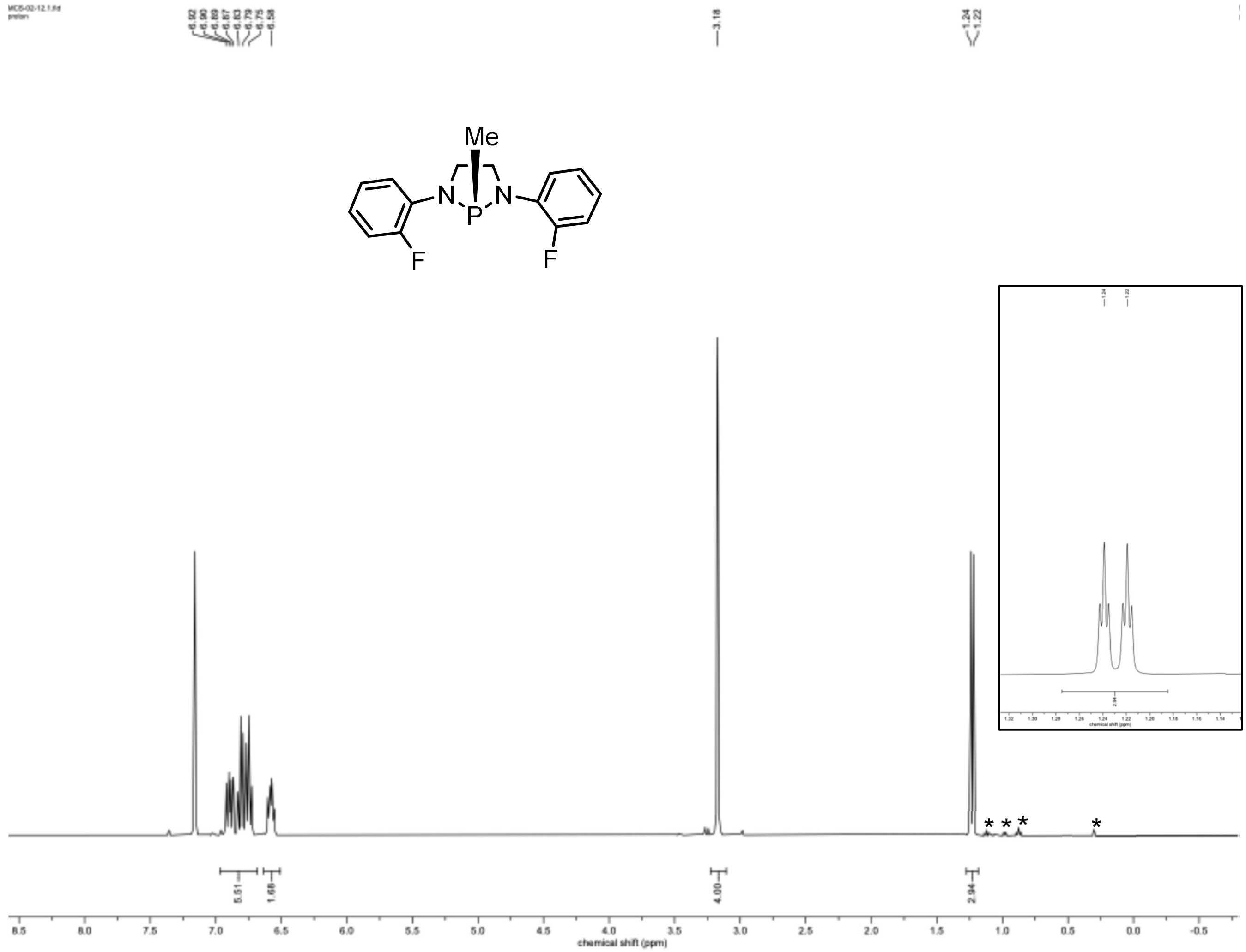

Figure S63: ^{1}H NMR (C_6D_6, 400 MHz) spectrum of (FP^{Me}F) (**37**). Residual solvent denoted with an asterisk (*)

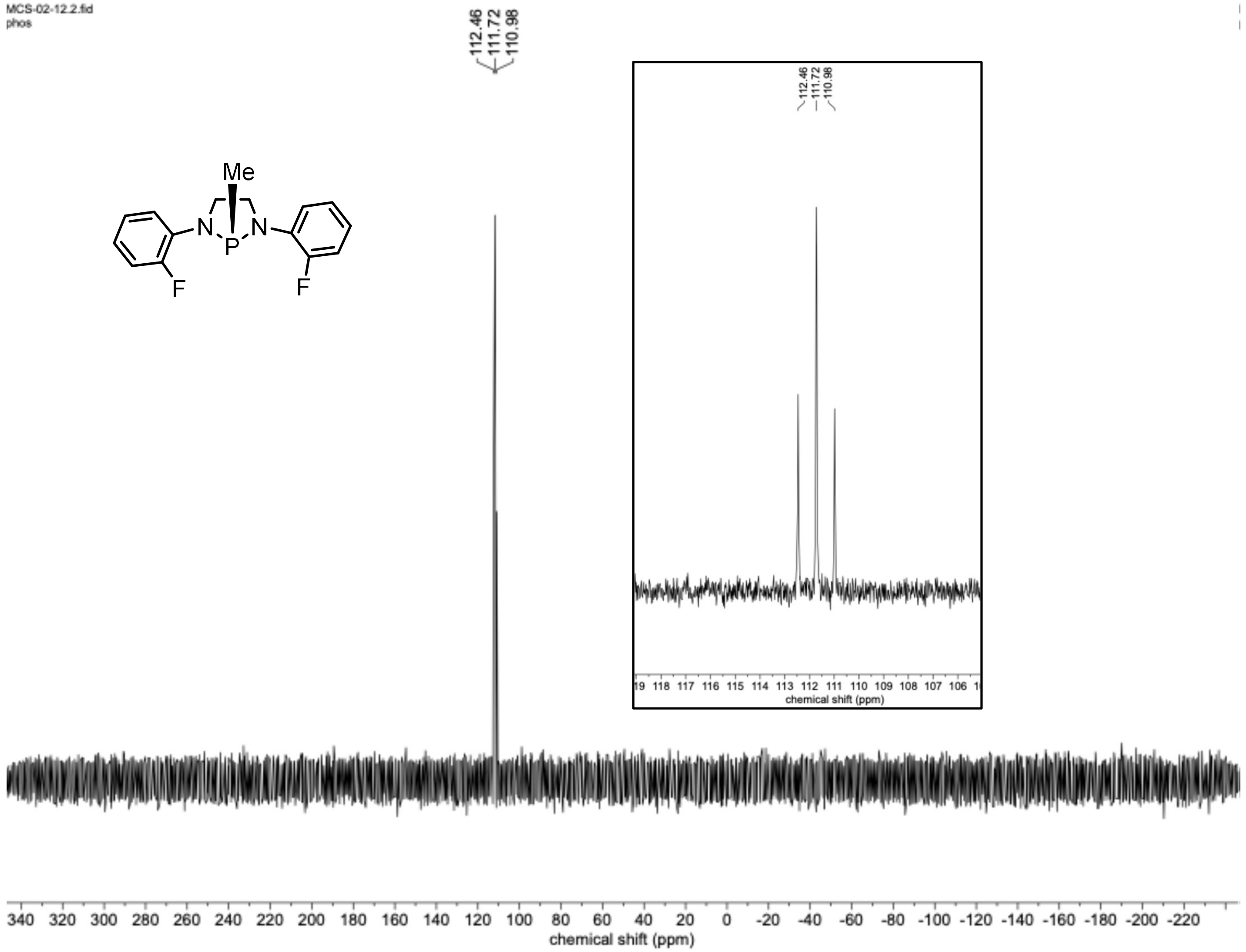

Figure S64: $^{31}P\{^1H\}$ NMR (C_6D_6, 162 MHz) spectrum of ($FP^{Me}F$) (**37**).

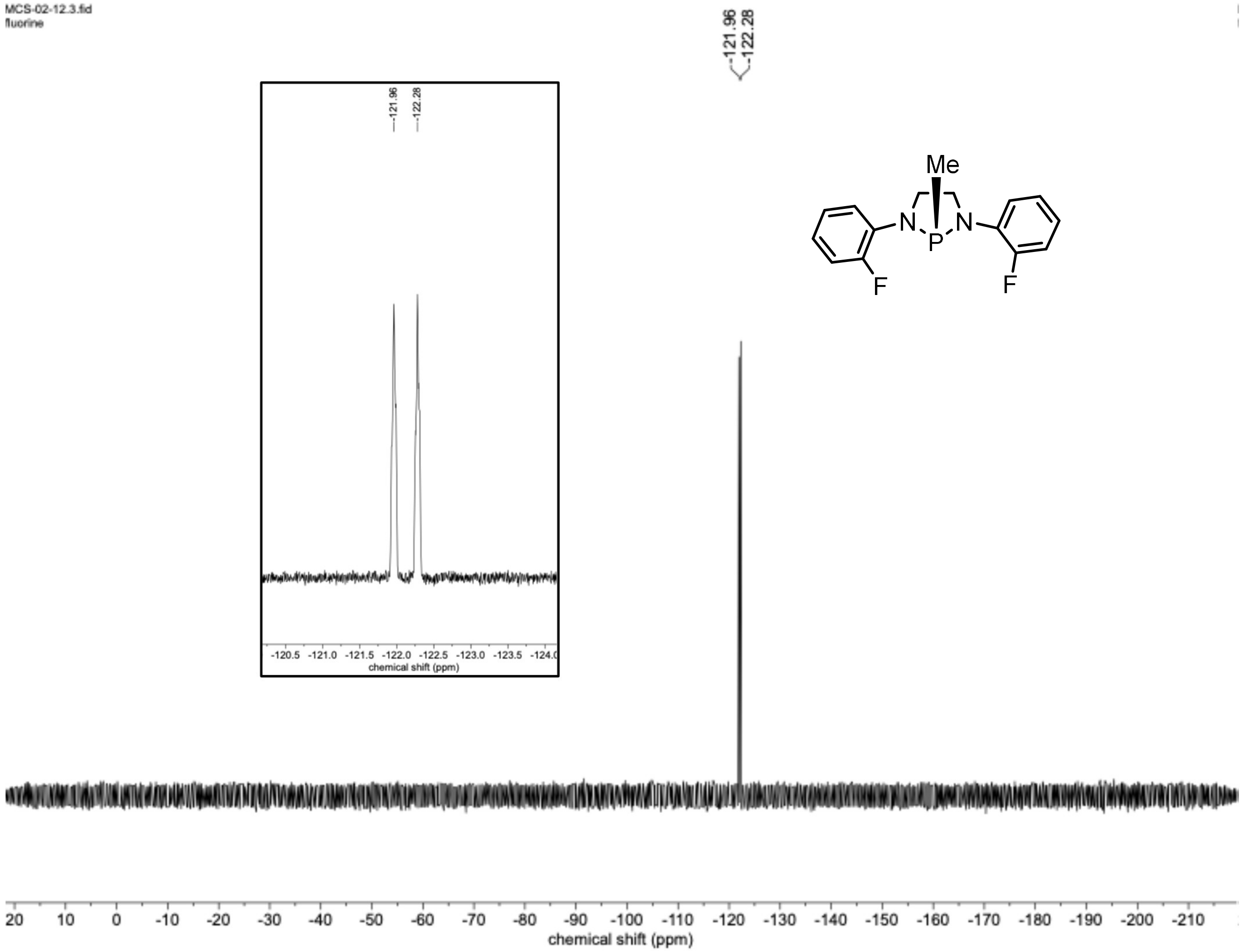

Figure S65: ^{19}F NMR (C_6D_6, 377 MHz) spectrum of ($FP^{Me}F$) (**37**).

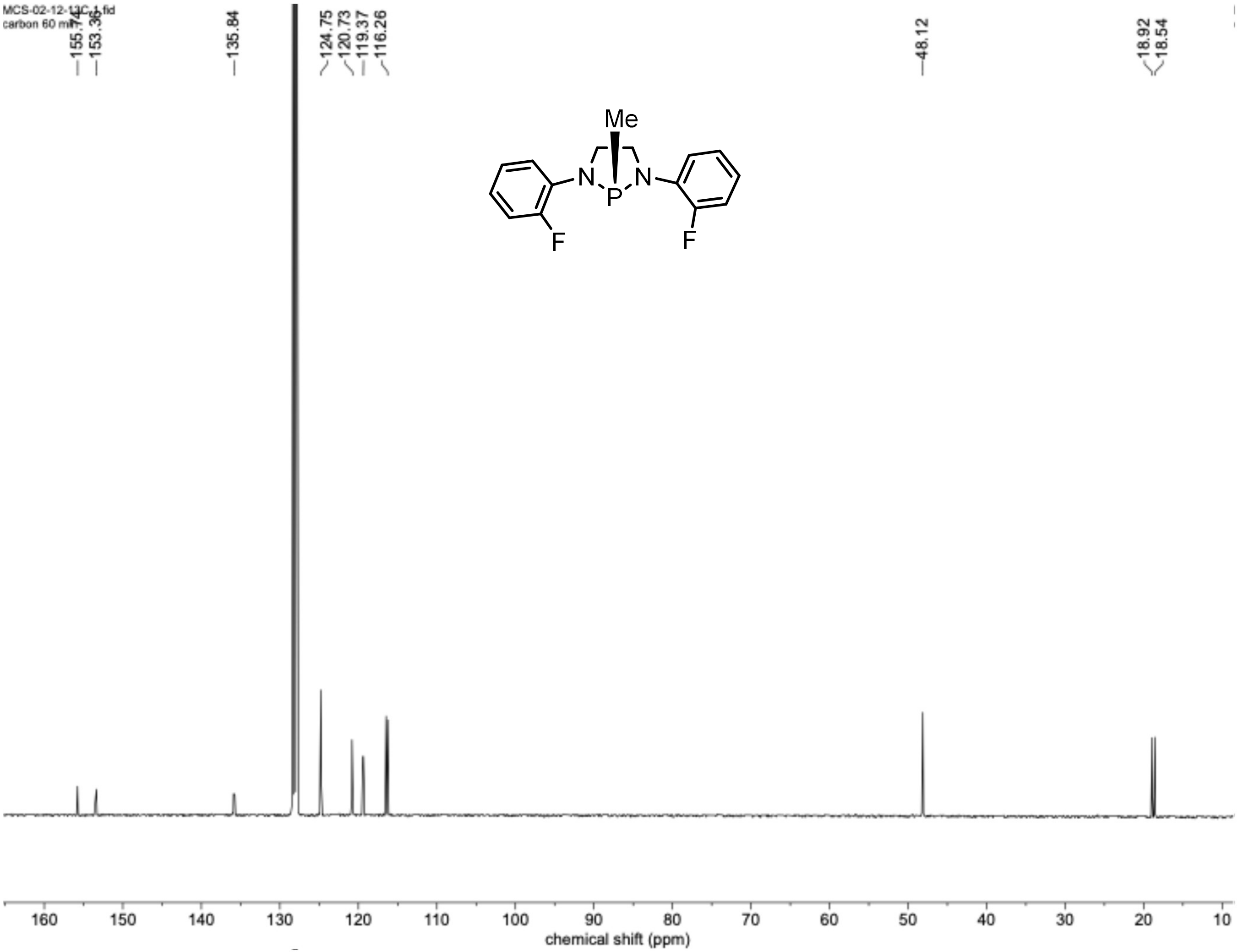

Figure S66: $^{13}C\{^1H\}$ NMR (C_6D_6, 151 MHz) spectrum of ($FP^{Me}F$) (**37**).

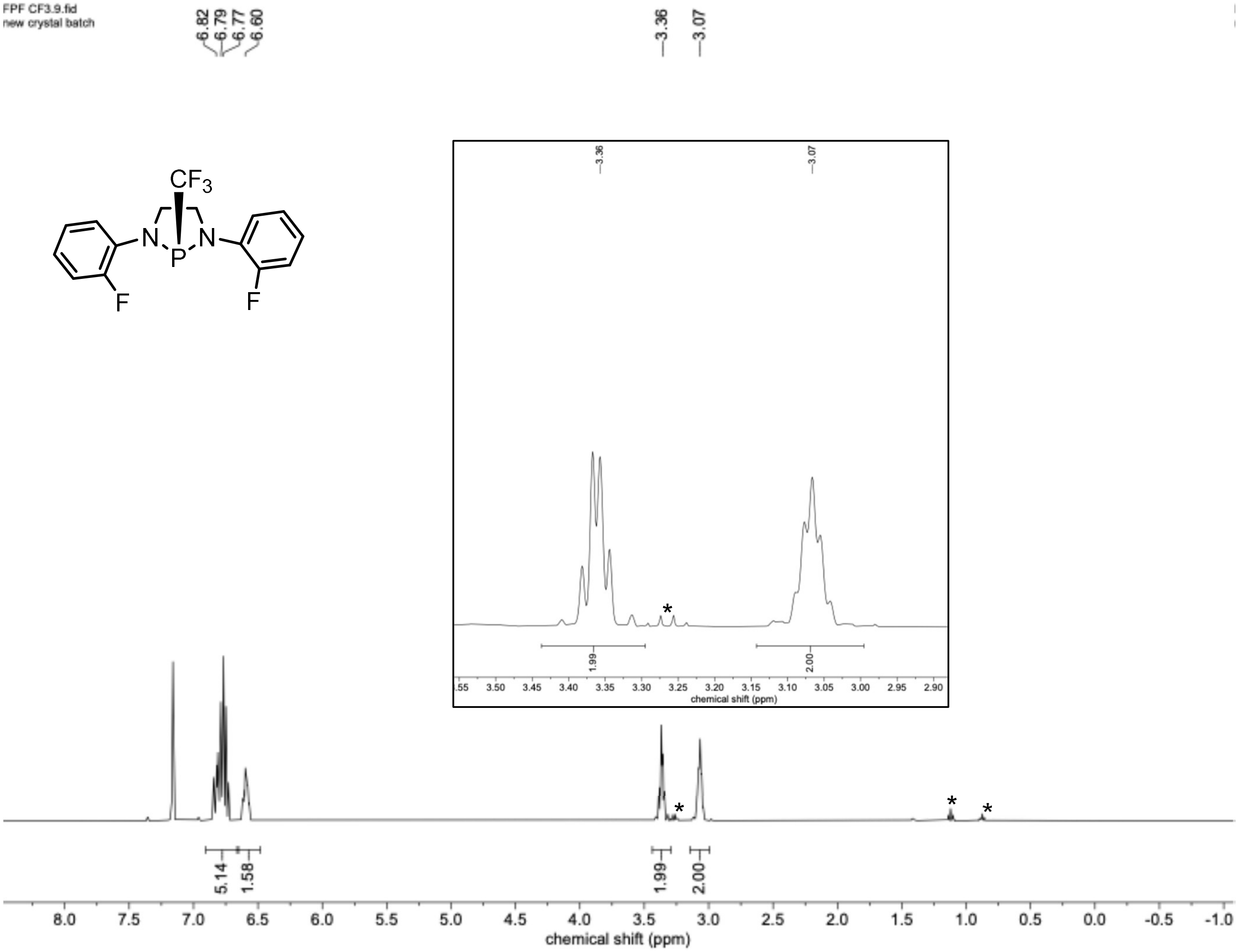

Figure S67: ^{1}H NMR (C_6D_6, 400 MHz) spectrum of ($FP^{CF3}F$) (**38**). Residual solvent denoted with an asterisk (*).

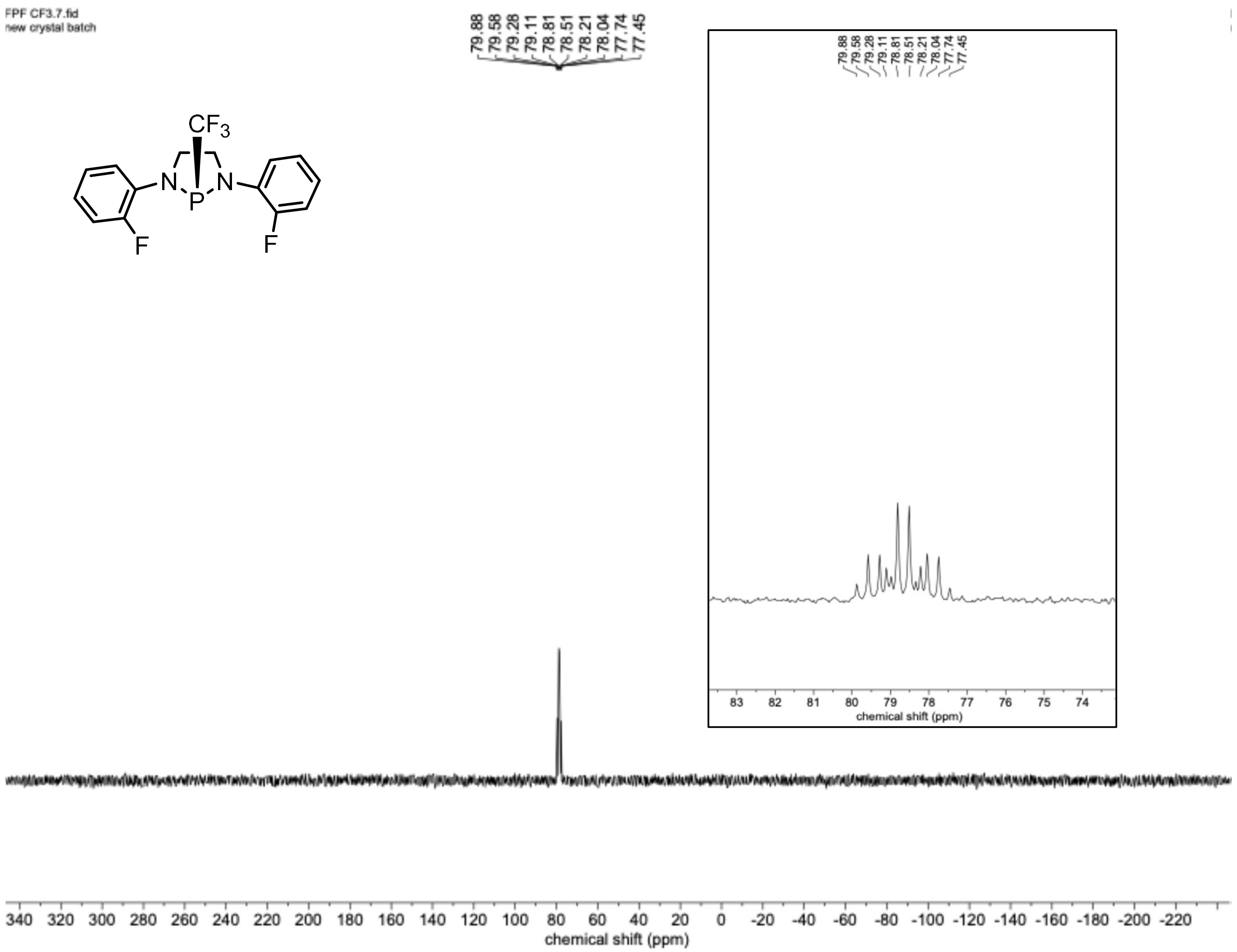

Figure S68: $^{31}P\{^1H\}$ NMR (C_6D_6, 162 MHz) spectrum of ($FP^{CF3}F$) (**38**).

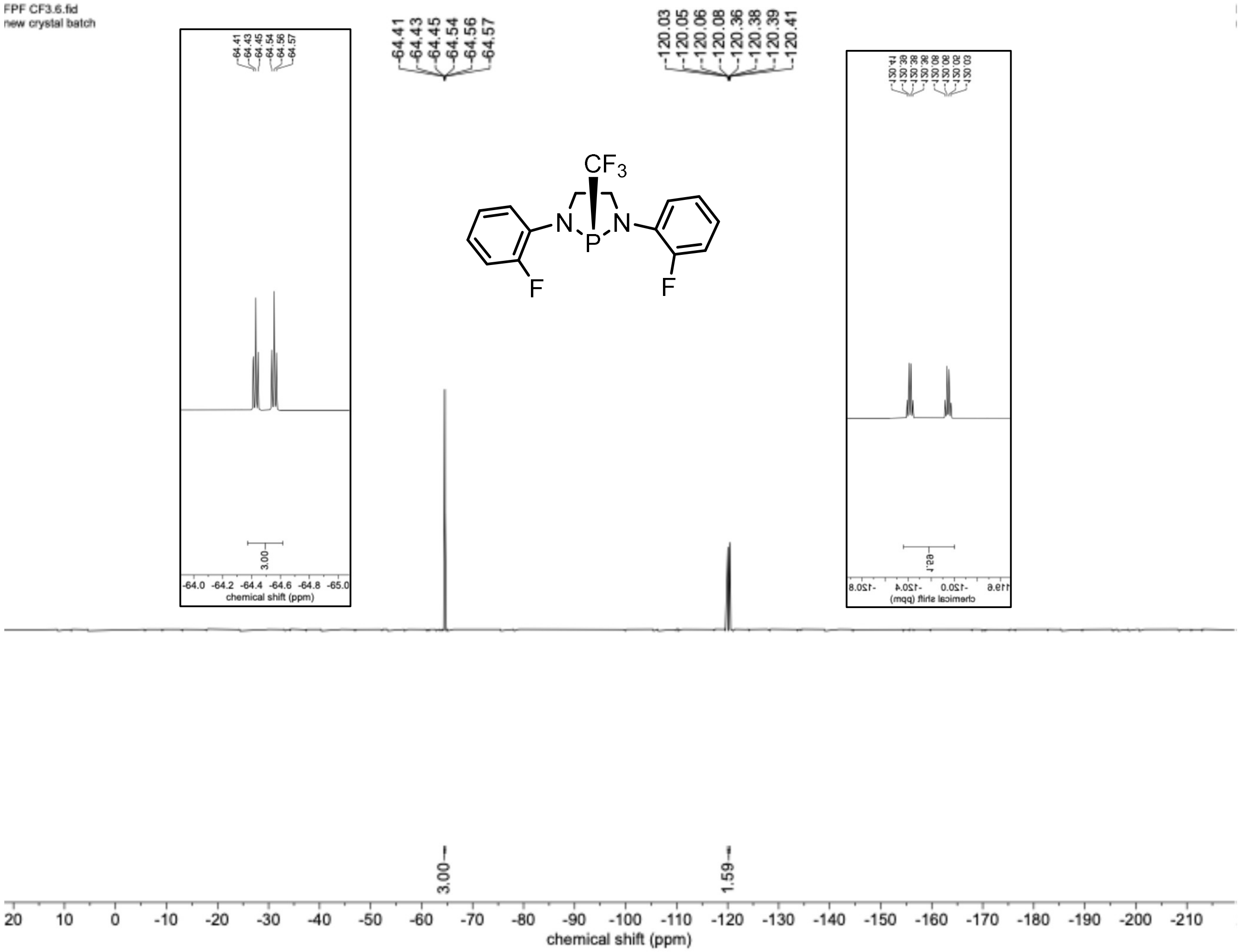

Figure S69: ^{19}F NMR (C_6D_6, 377 MHz) spectrum of ($FP^{CF3}F$) (**38**).

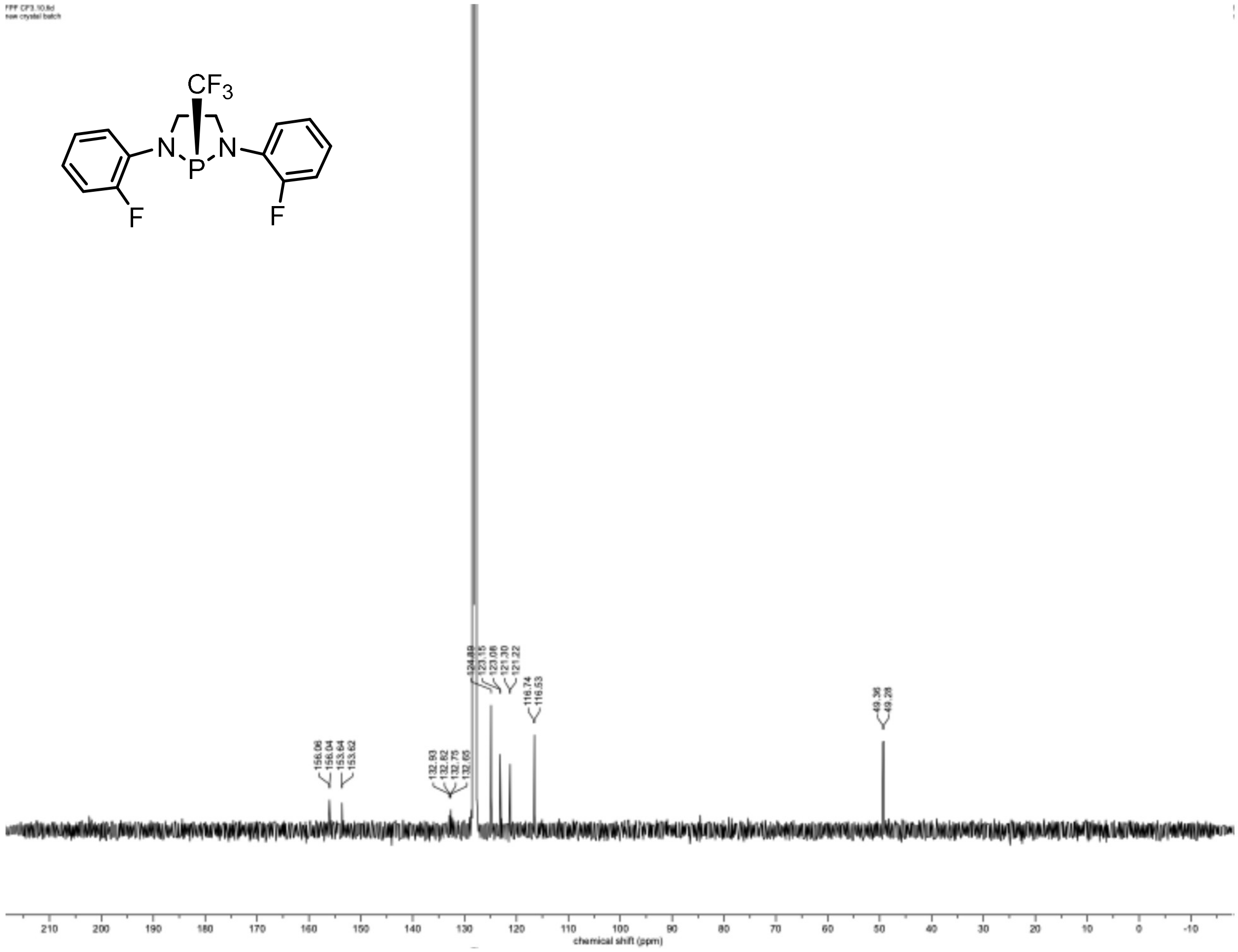

Figure S70: $^{13}C\{^1H\}$ NMR (C_6D_6, 151 MHz) spectrum of ($FP^{CF3}F$) (**38**).

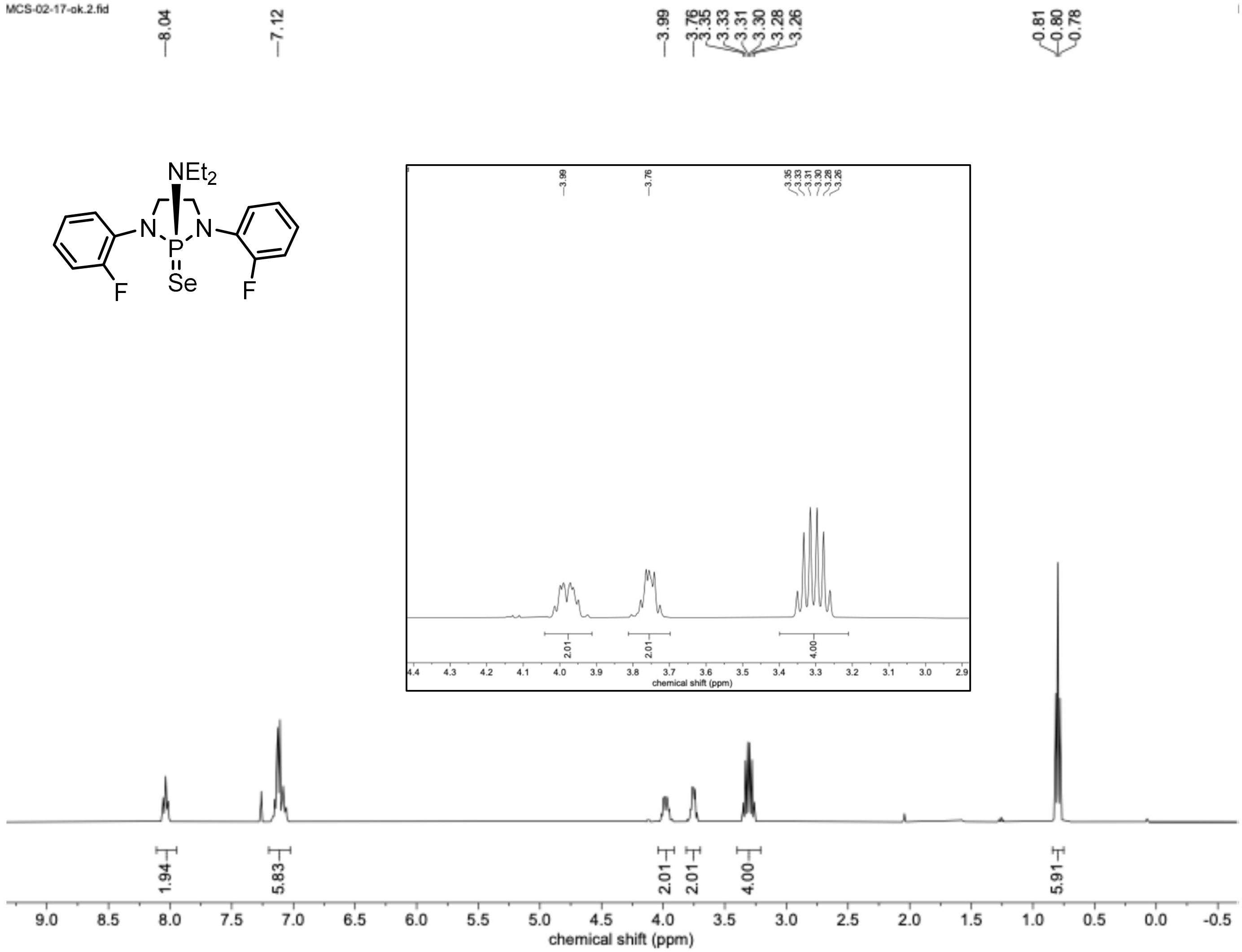

Figure S71: ^{1}H NMR ($CDCl_3$, 400 MHz) spectrum of ($F(Se{=})P^{NEt2}F$) (**39**).

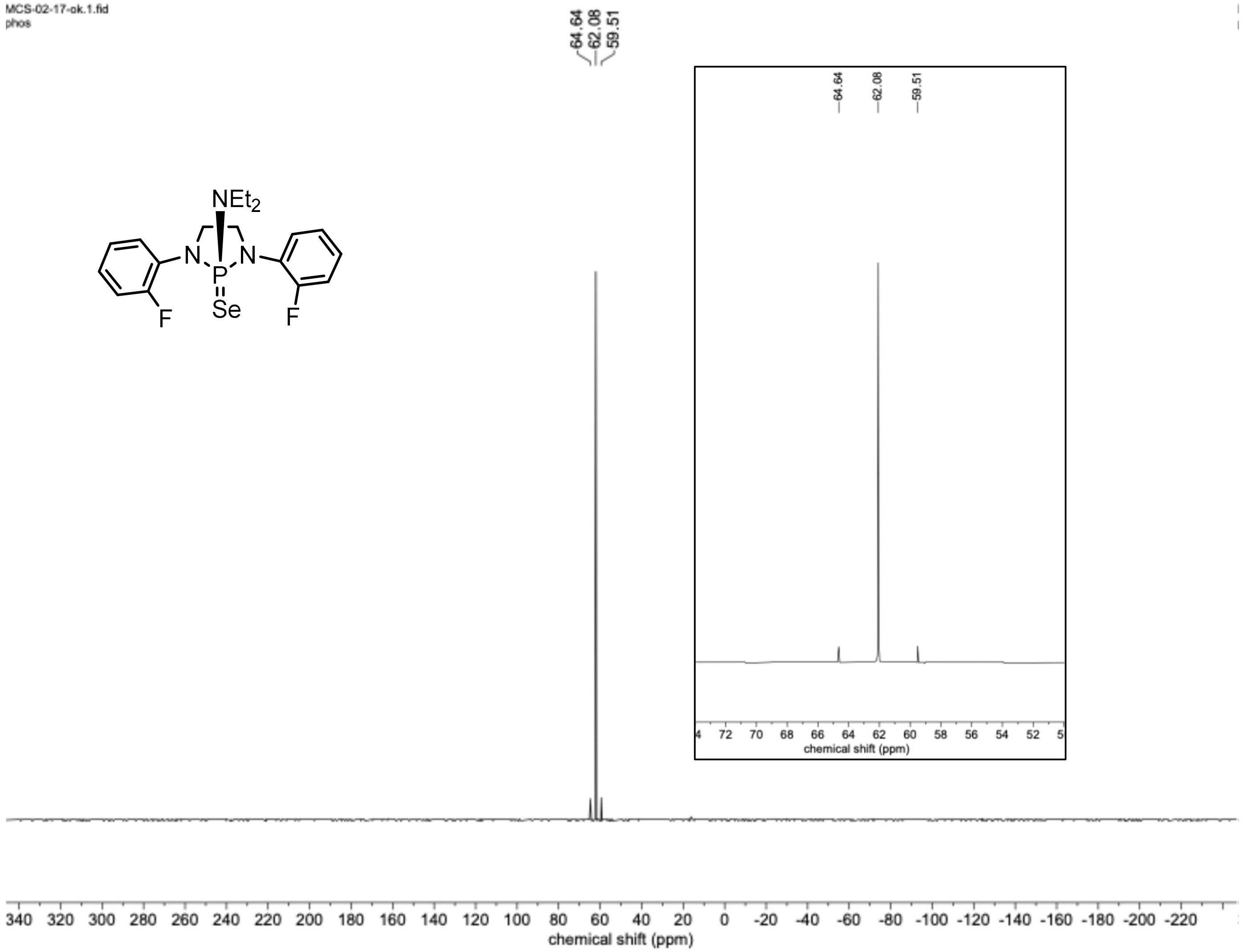

Figure S72: $^{31}P\{^1H\}$ NMR ($CDCl_3$, 162 MHz) spectrum of (F(Se=)P^{NEt2}F) (**39**).

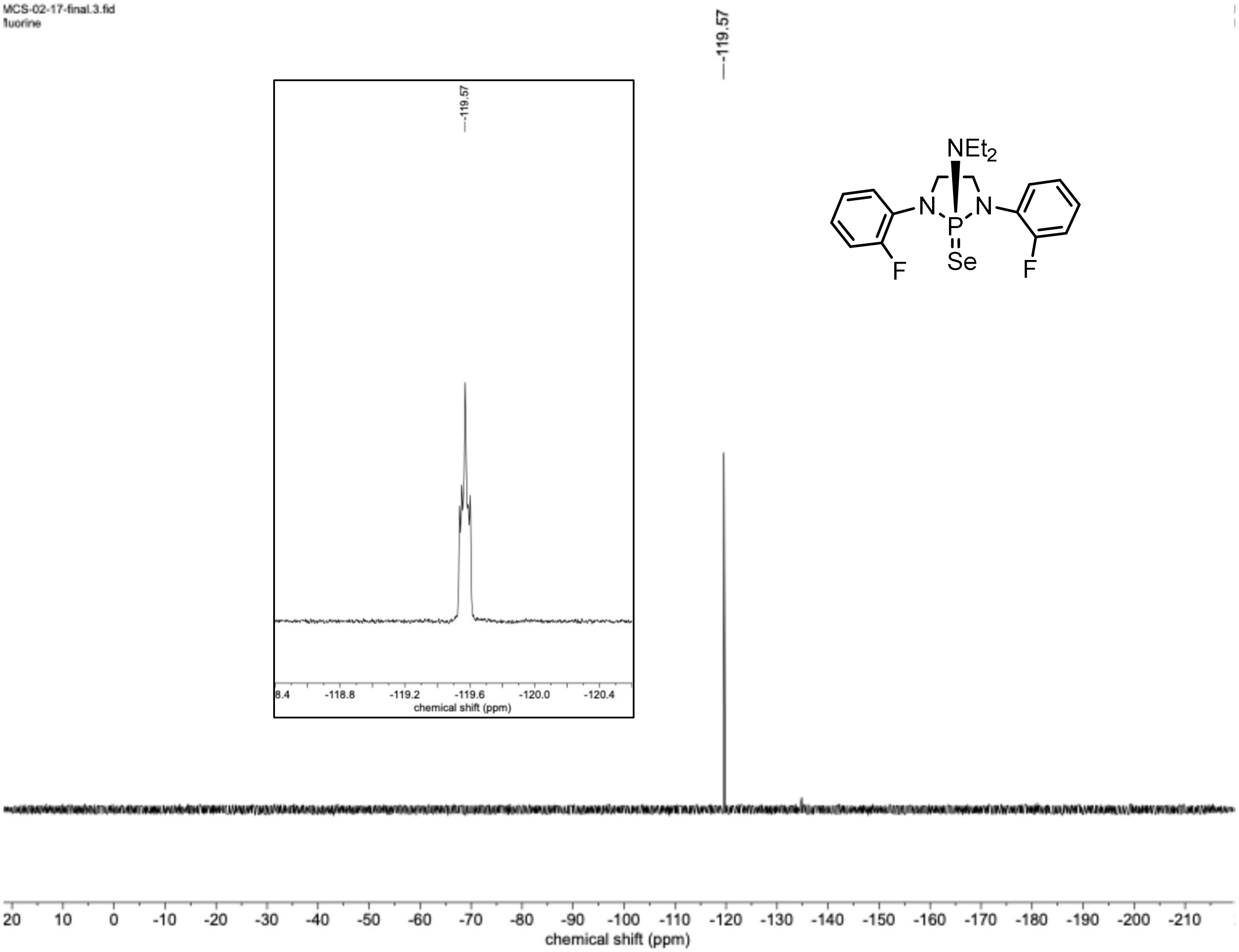

Figure S73: ^{19}F NMR ($CDCl_3$, 377 MHz) spectrum of (F(Se=)P^{NEt2}F) (**39**).

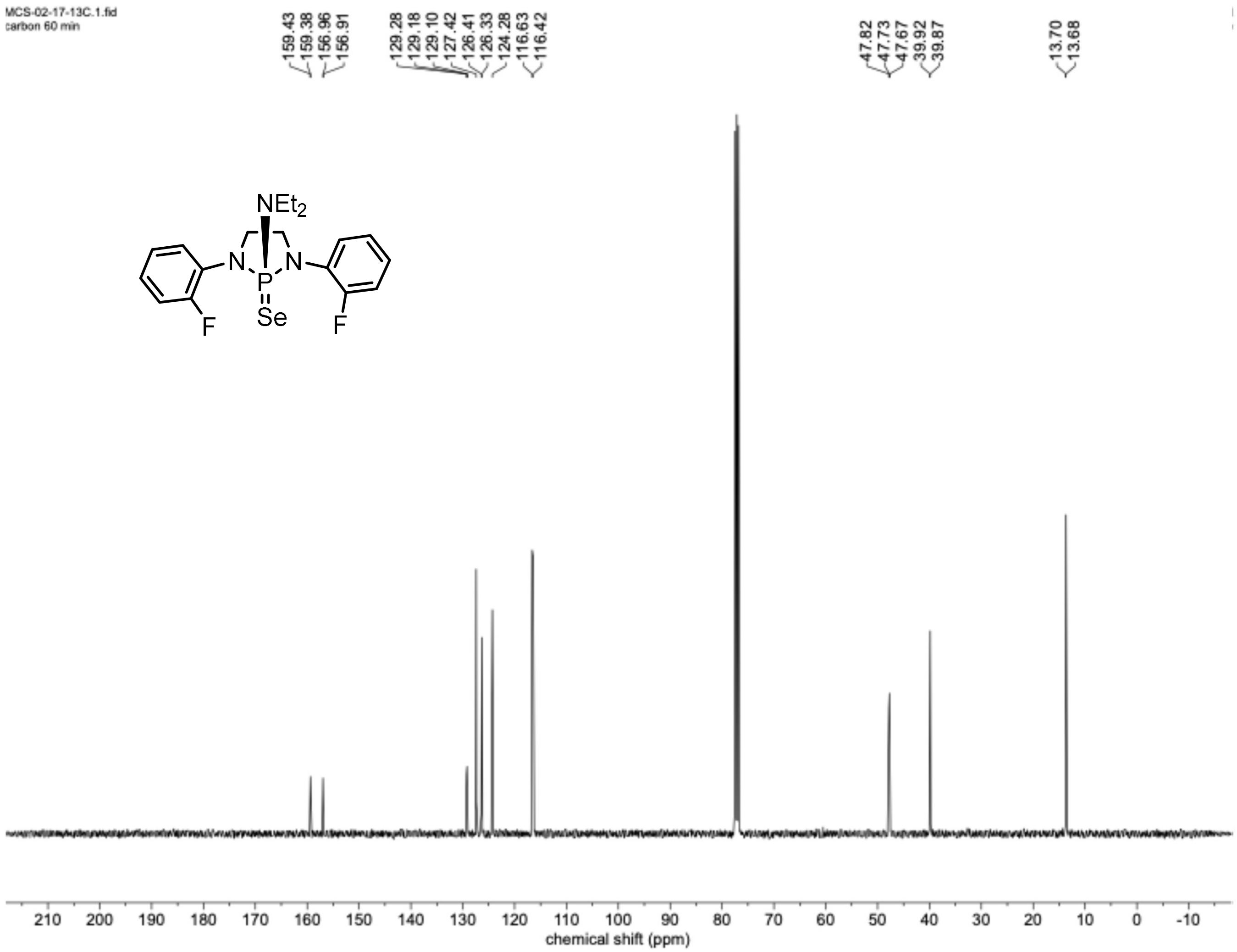

Figure S74: $^{13}C\{^1H\}$ NMR ($CDCl_3$, 151 MHz) spectrum of (F(Se=)P^{NEt2}F) (**39**).

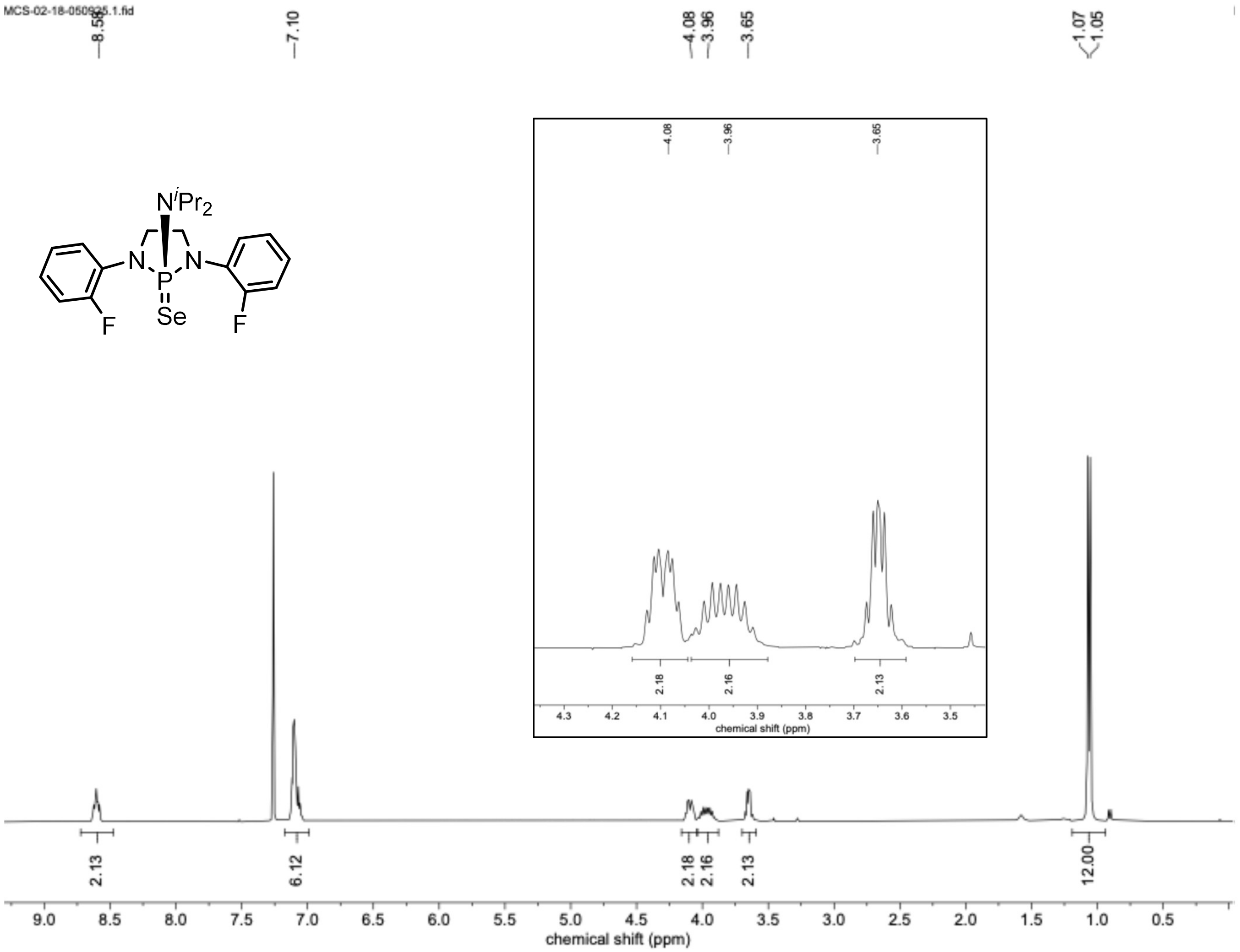

Figure S75: ^{1}H NMR ($CDCl_3$, 400 MHz) spectrum of (F(Se=)P^{NiPr2}F) (**40**).

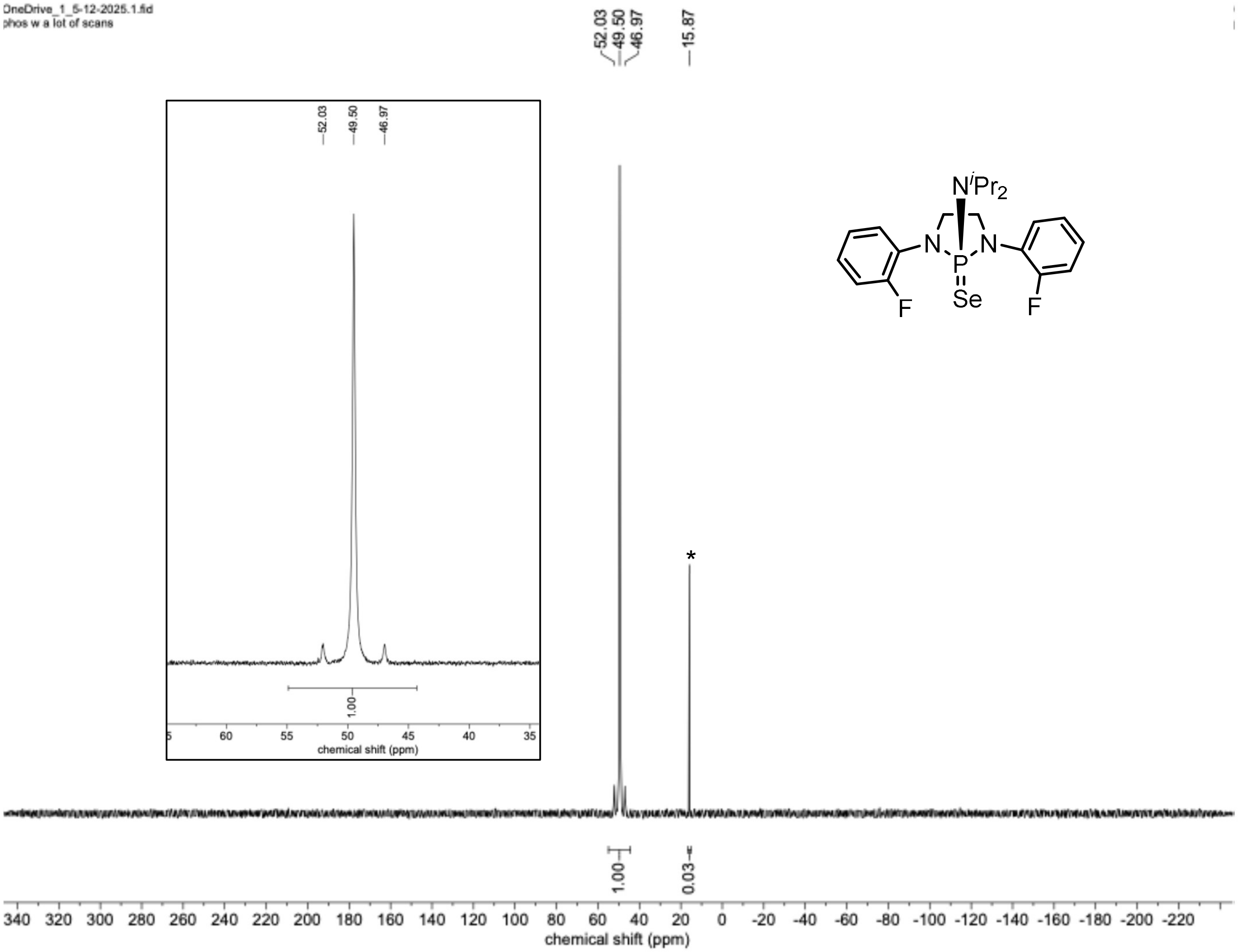

Figure S76: $^{31}P\{^1H\}$ NMR ($CDCl_3$, 162 MHz) spectrum of (F(Se=)$P^{N\mathit{i}Pr2}$F) (**40**). Minor Unidentified phosphorous-containing impurity denoted with an asterisk (*).

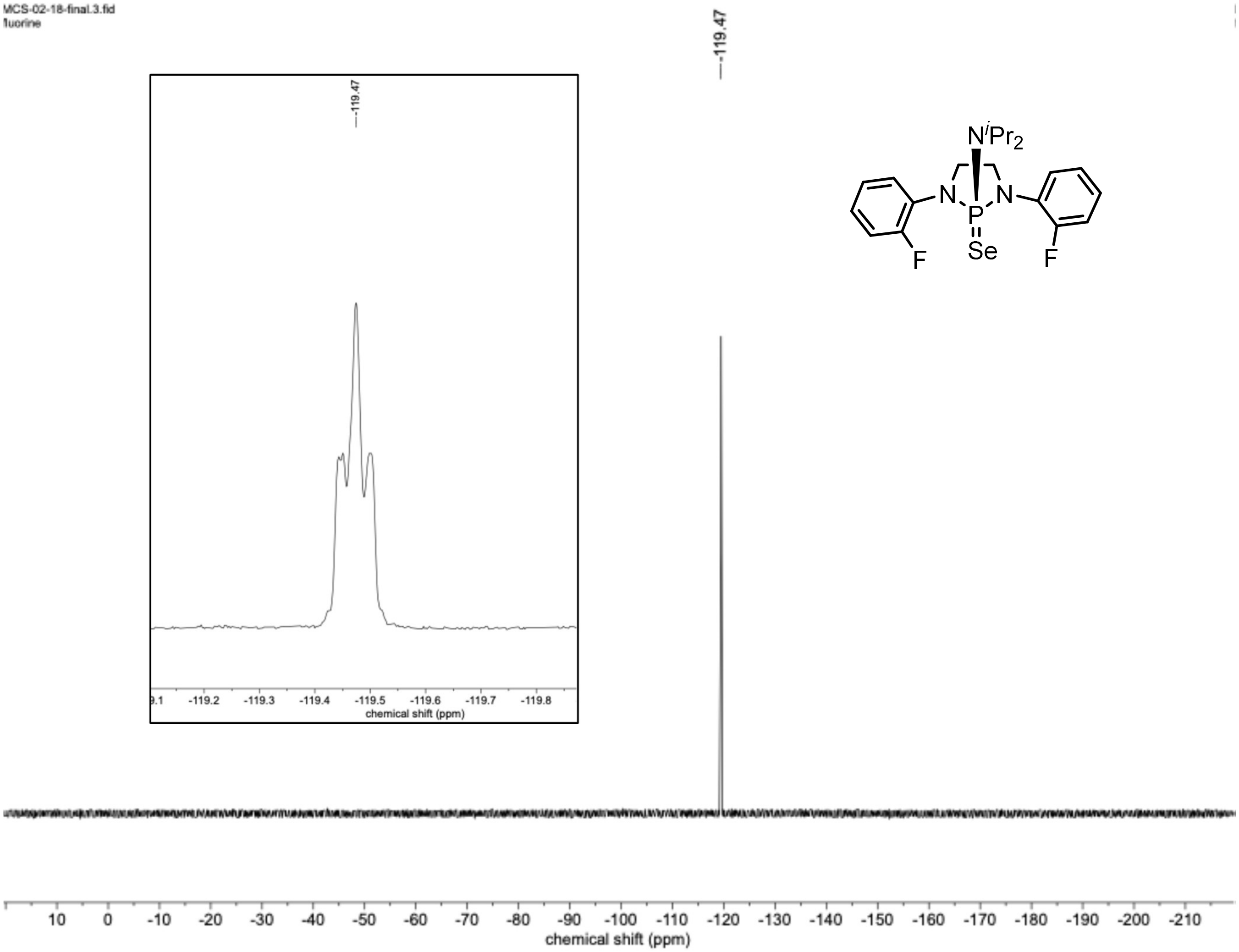

Figure S77: ^{19}F NMR (CDCl$_3$, 377 MHz) spectrum of (F(Se=)P^{NiPr2}F) (**40**).

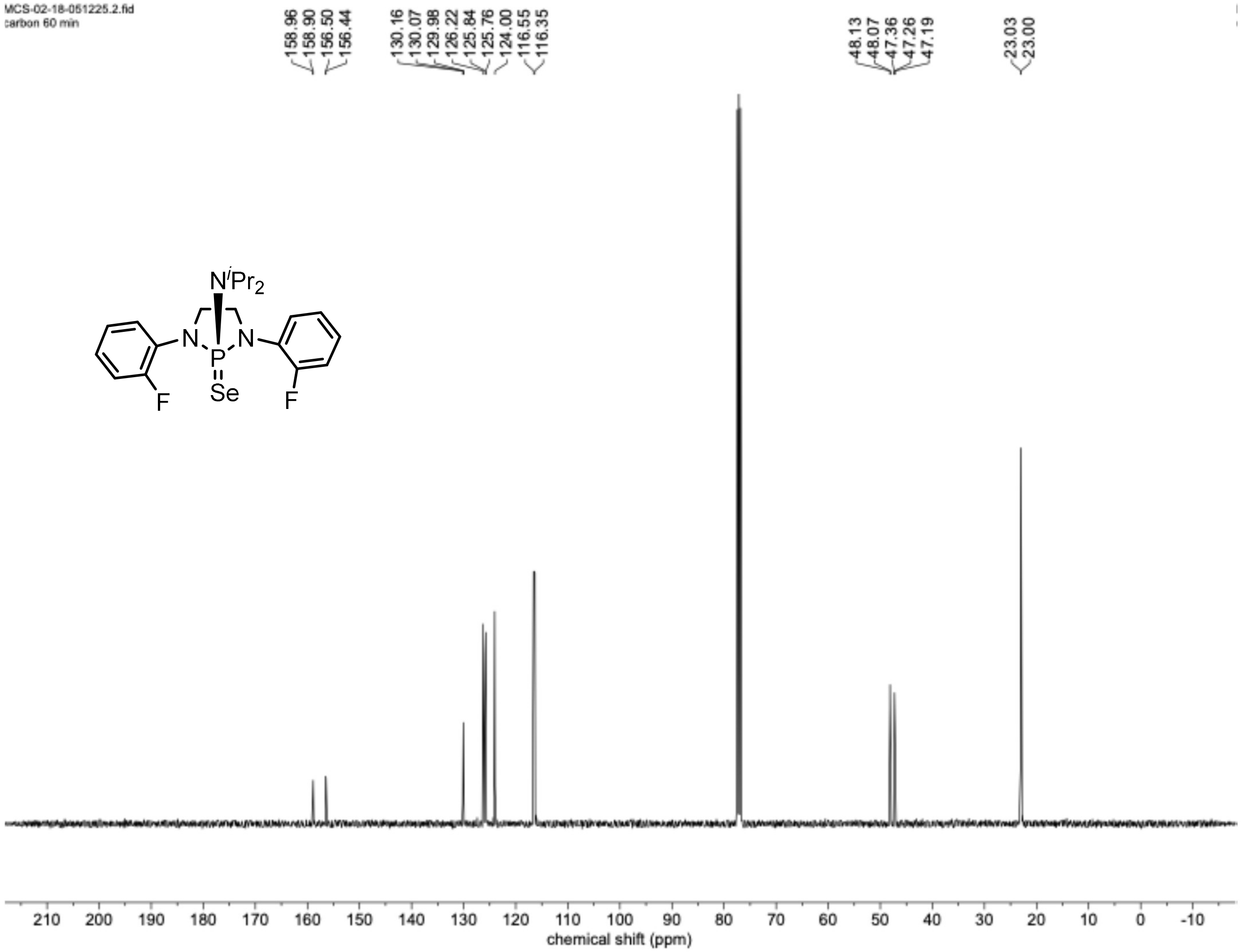

Figure S78: $^{13}C\{^1H\}$ NMR ($CDCl_3$, 151 MHz) spectrum of (F(Se=)$P^{N\textit{i}Pr2}$F) (**40**).

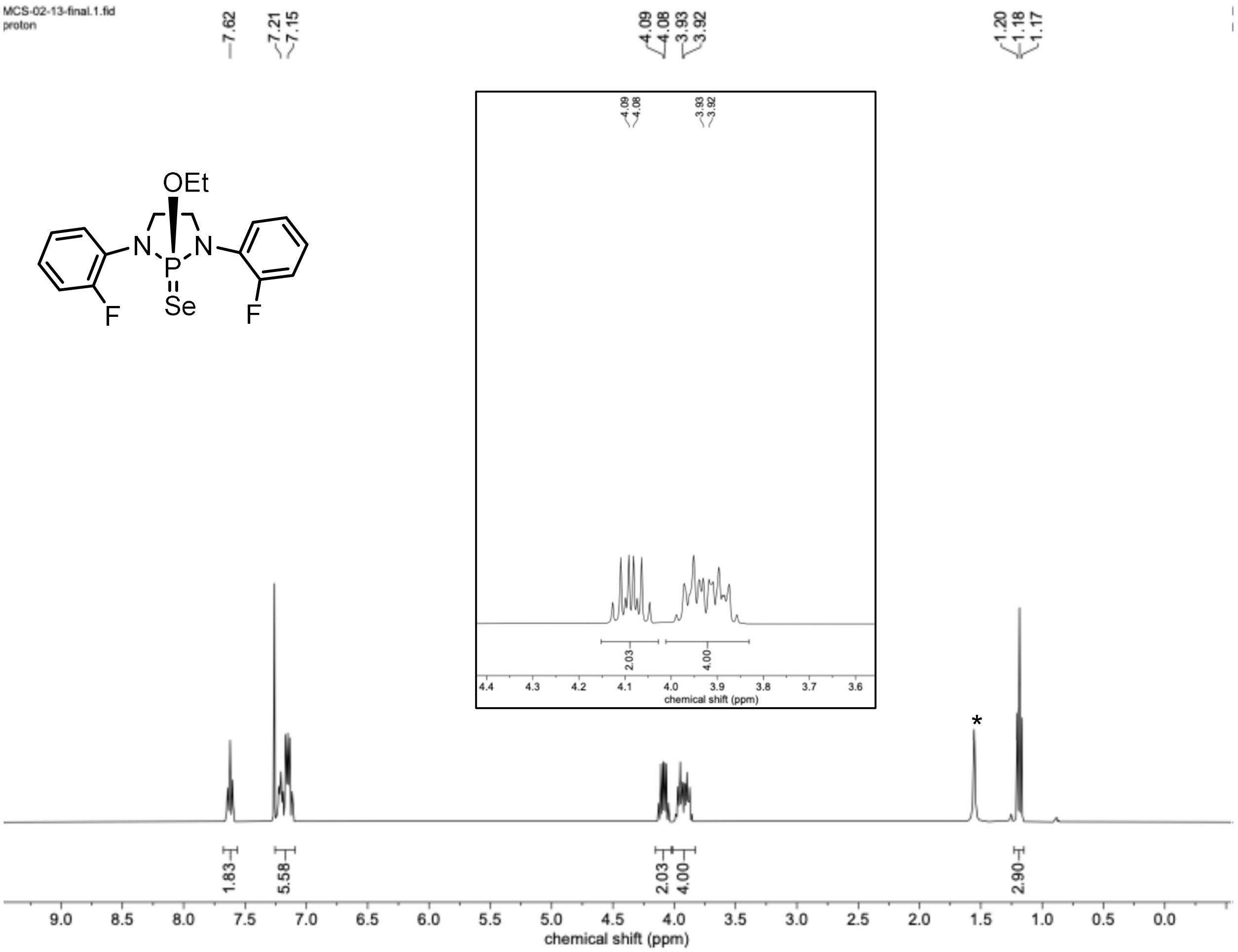

Figure S79: ^{1}H NMR ($CDCl_3$, 400 MHz) spectrum of (F(Se=)P^{OEt}F) (**41**). Water denoted with an asterisk (*)

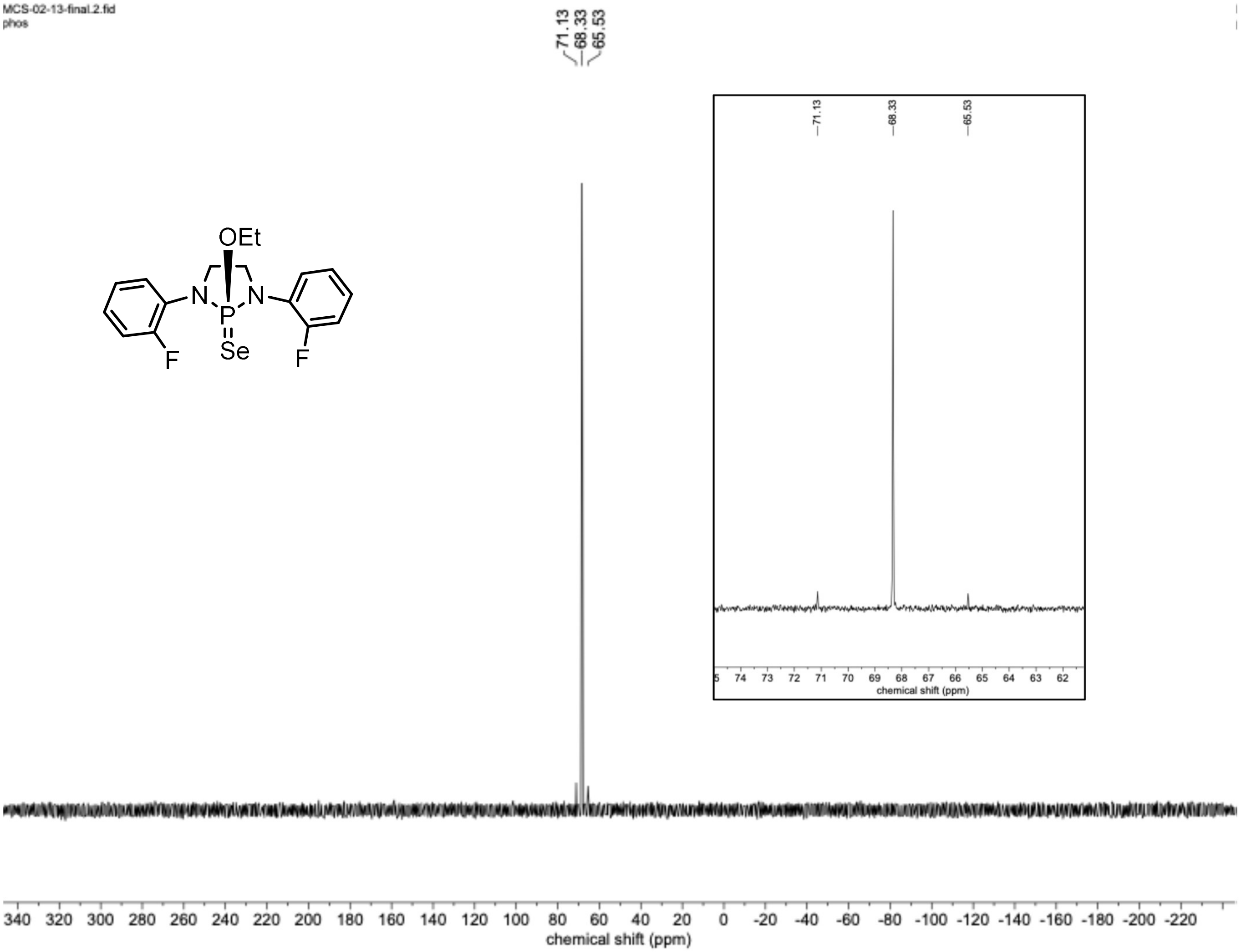

Figure S80: $^{31}P\{^{1}H\}$ NMR ($CDCl_3$, 162 MHz) spectrum of (F(Se=)P^{OEt}F) (**41**).

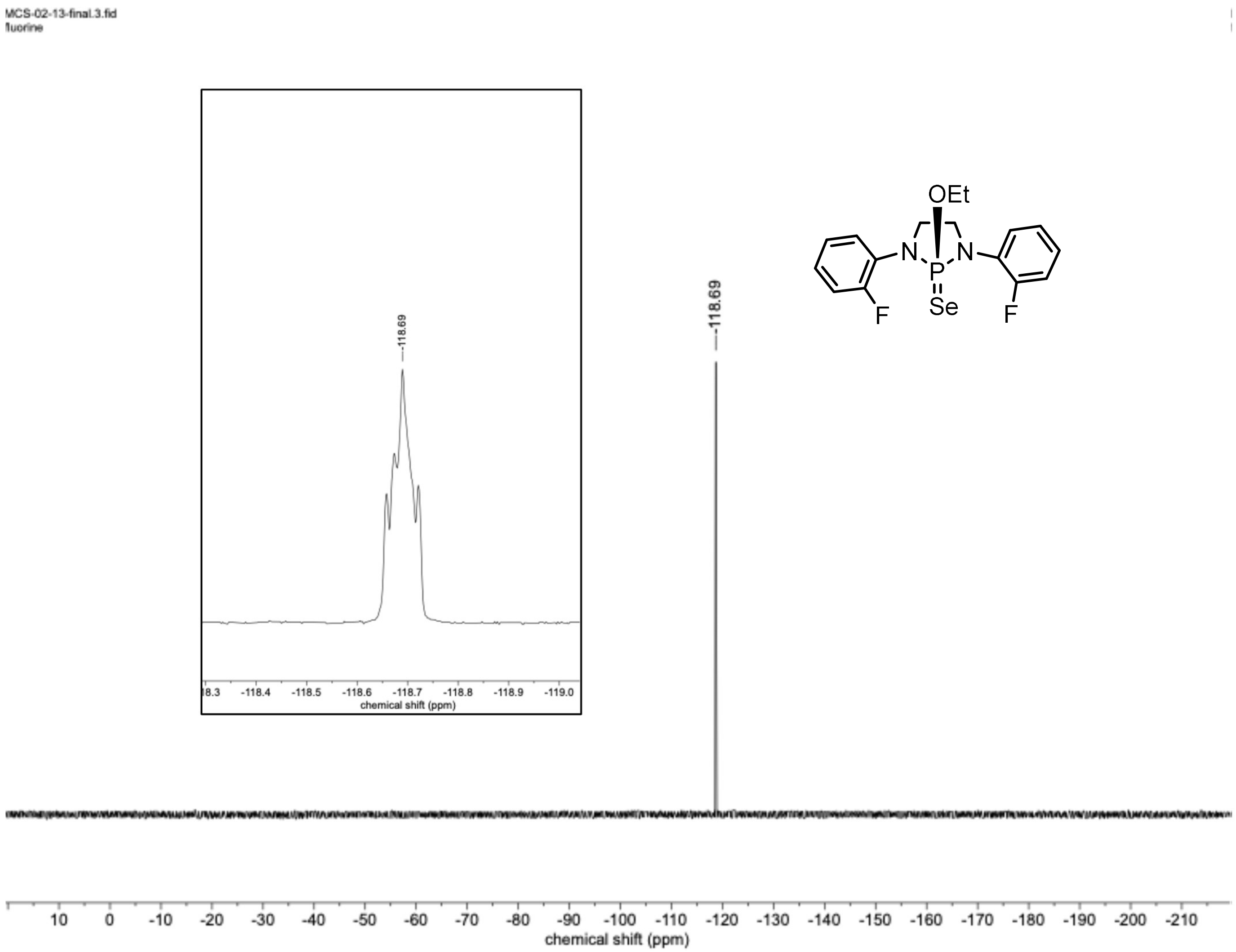

Figure S81: ^{19}F NMR ($CDCl_3$, 377 MHz) spectrum of (F(Se=)P^{OEt}F) (**41**).

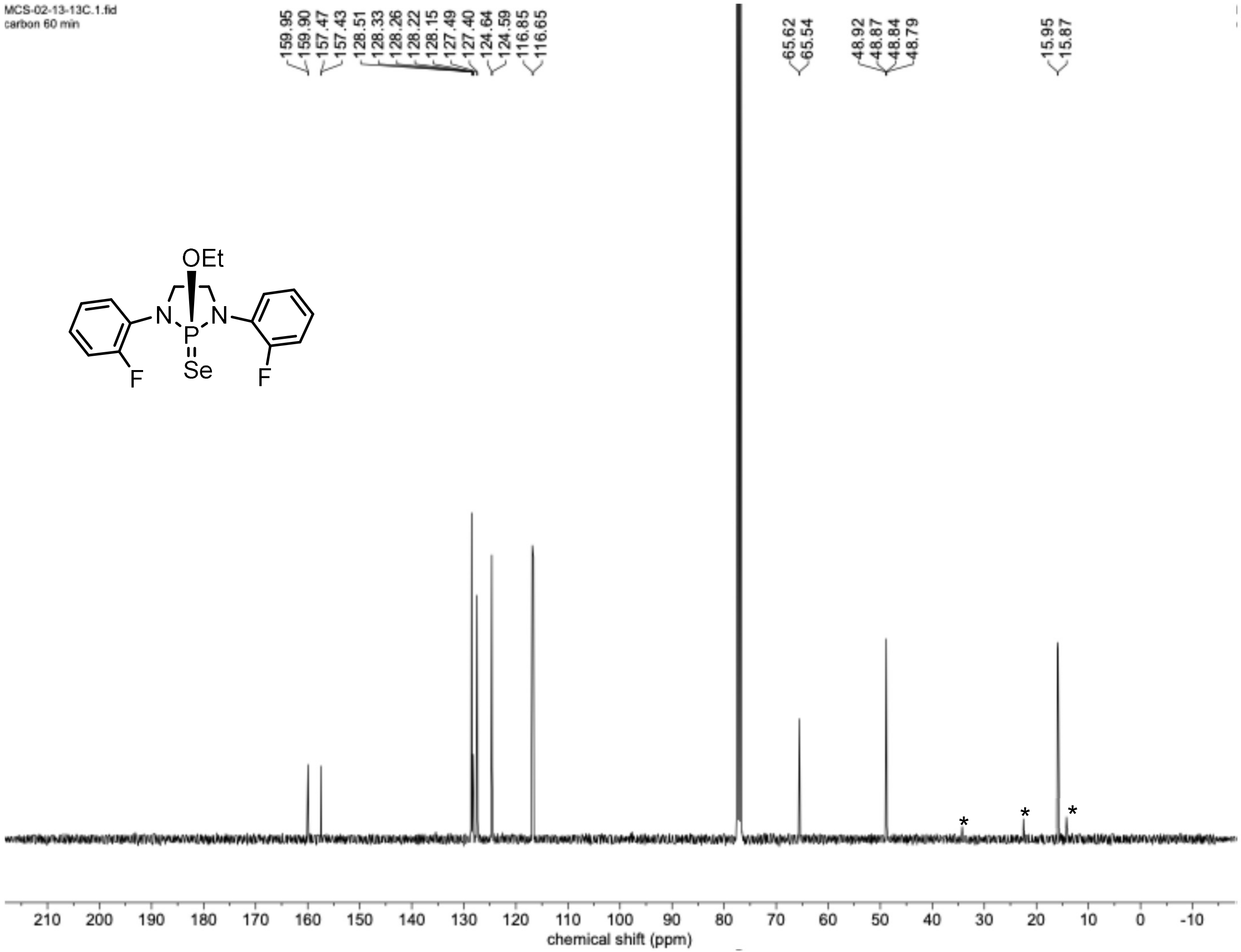

Figure S82: $^{13}C\{^1H\}$ NMR ($CDCl_3$, 151 MHz) spectrum of (F(Se=)P^{OEt}F) (**41**). Residual solvent denoted with an asterisk (*).

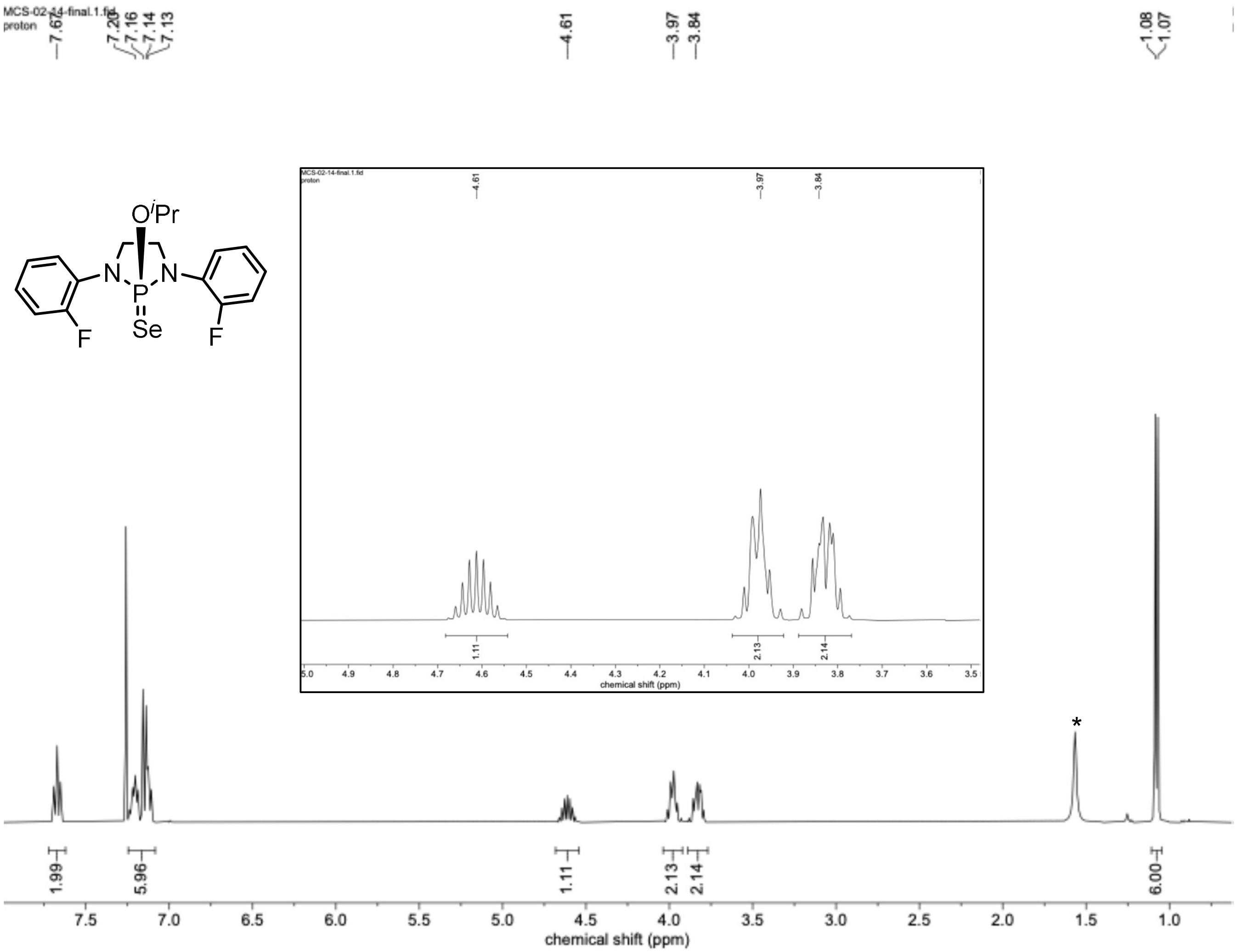

Figure S83: ^{1}H NMR ($CDCl_3$, 400 MHz) spectrum of (F(Se=)P^{OiPr}F) (**42**). Water denoted with an asterisk (*)

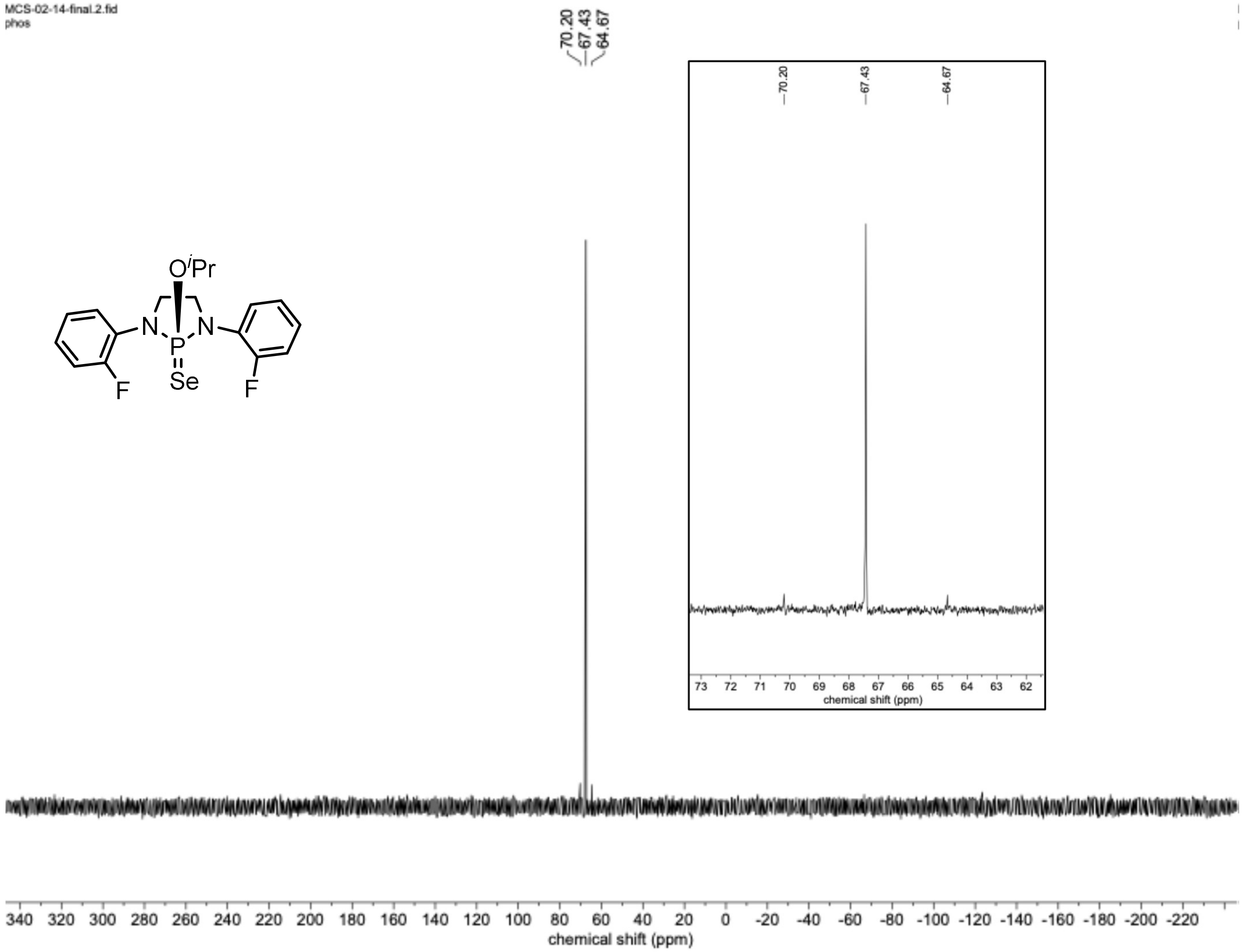

Figure S84: $^{31}P\{^{1}H\}$ NMR ($CDCl_3$, 162 MHz) spectrum of (F(Se=)P^{OiPr}F) (**42**).

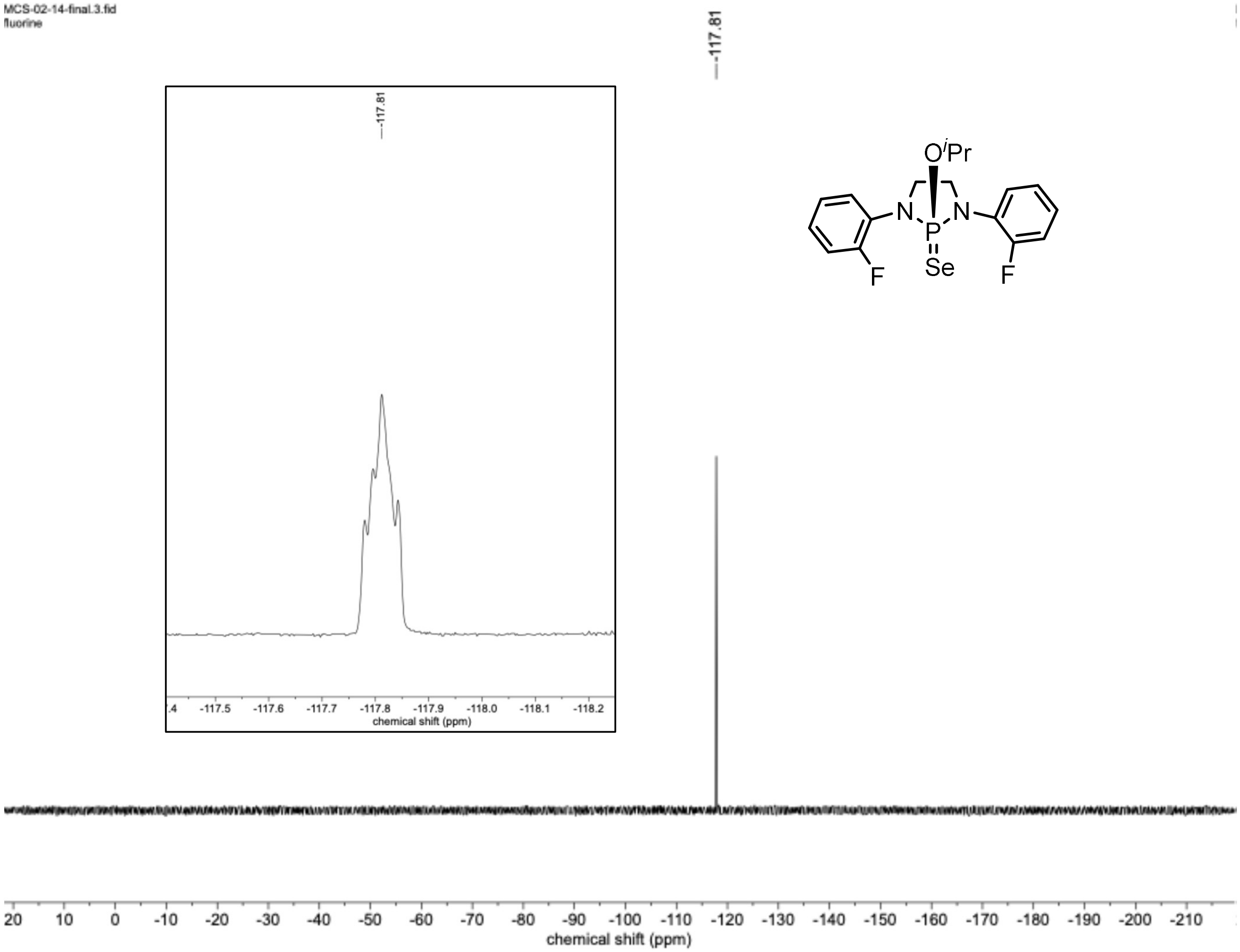

Figure S85: ^{19}F NMR ($CDCl_3$, 377 MHz) spectrum of (F(Se=)P^{OiPr}F) (**42**).

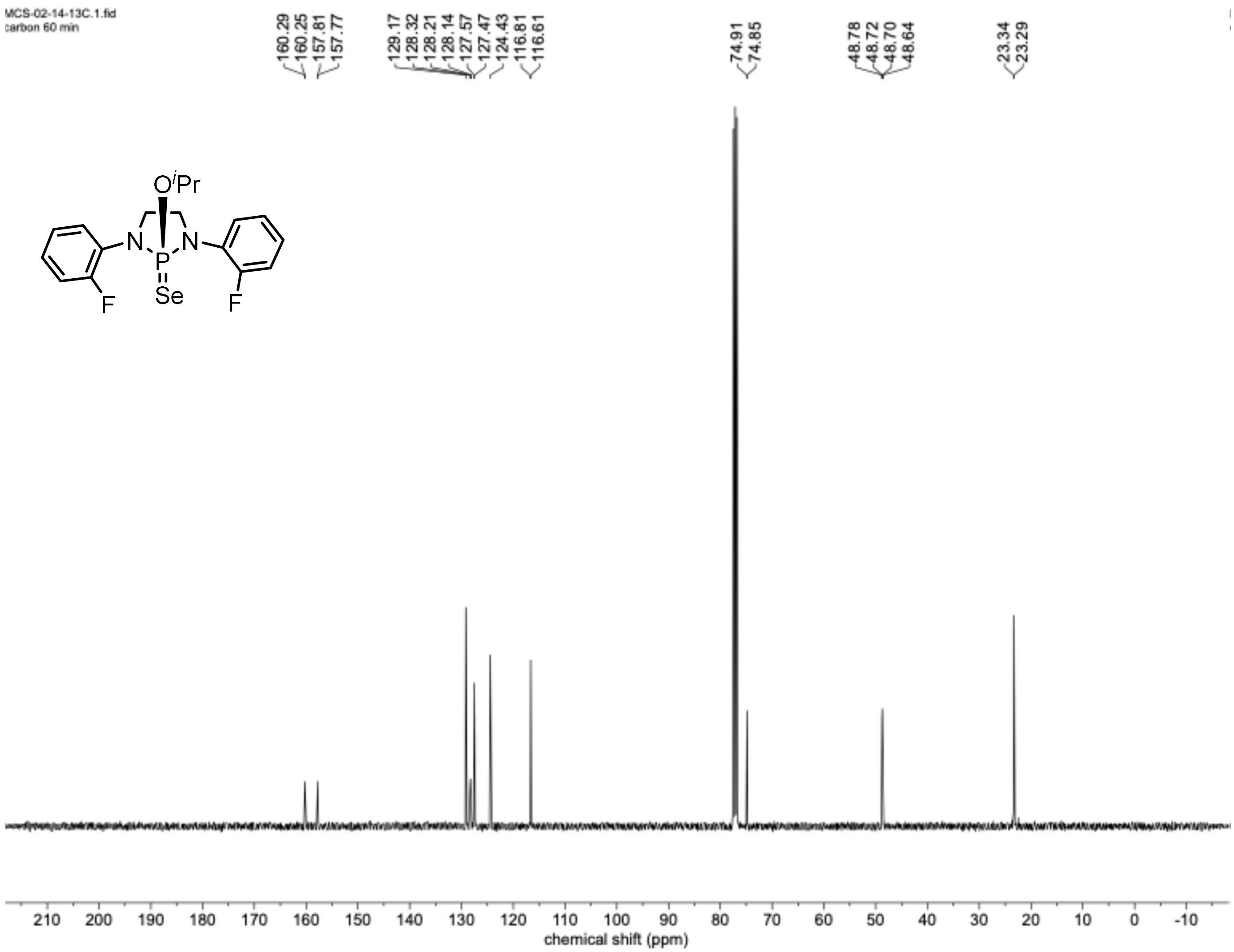

Figure S86: $^{13}C\{^1H\}$ NMR ($CDCl_3$, 151 MHz) spectrum of (F(Se=)P^{OiPr}F) (**42**).

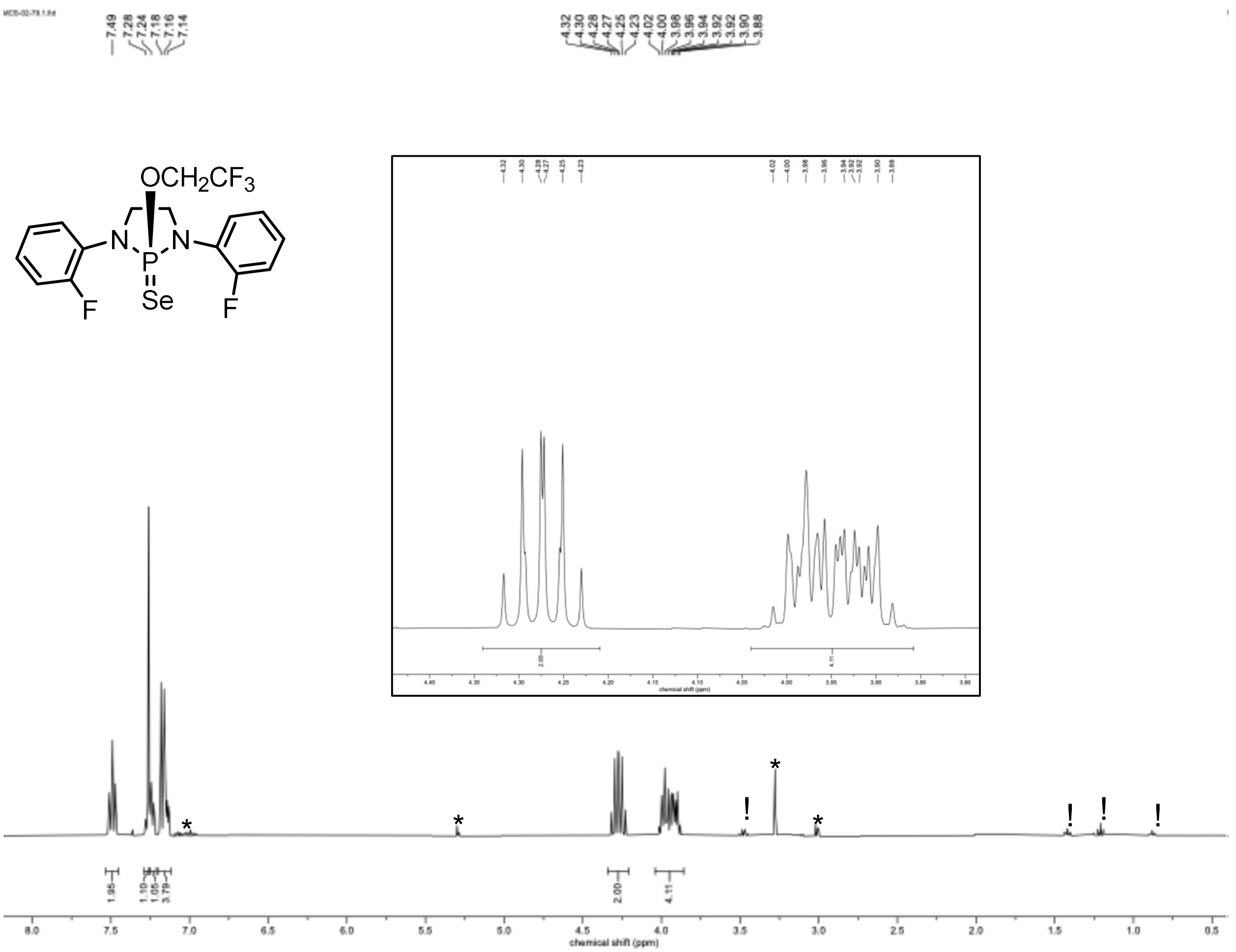

Figure S87: ^{1}H NMR ($CDCl_3$, 400 MHz) spectrum of (F(Se=)P^{OCH2CF3}F) (**43**). Unidentified minor impurity denoted with an asterisk (*). Residual solvent denoted with an exclamation point (!).

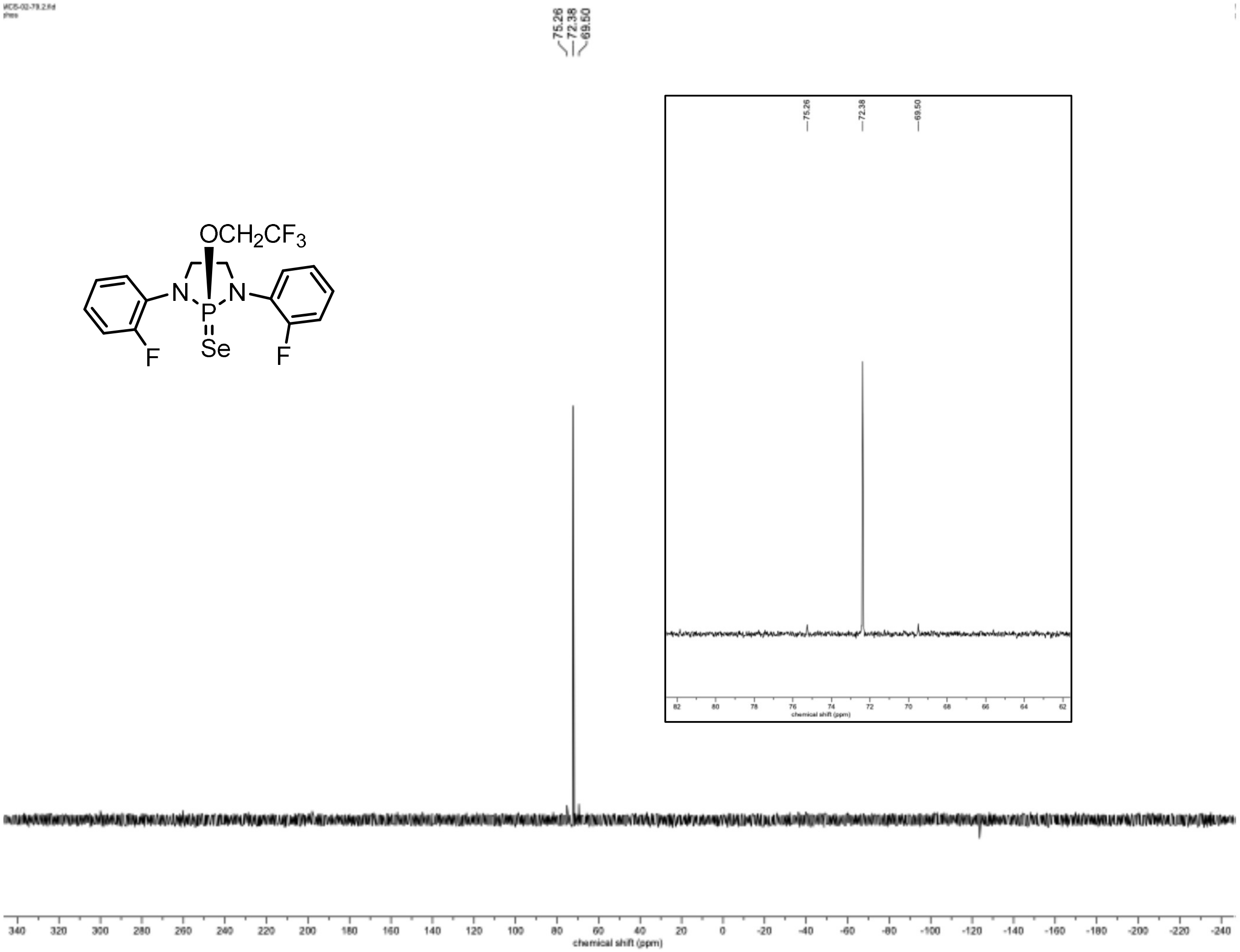

Figure S88: $^{31}P\{^1H\}$ NMR ($CDCl_3$, 162 MHz) spectrum of (F(Se=)$P^{OCH2CF3}$F) (**43**).

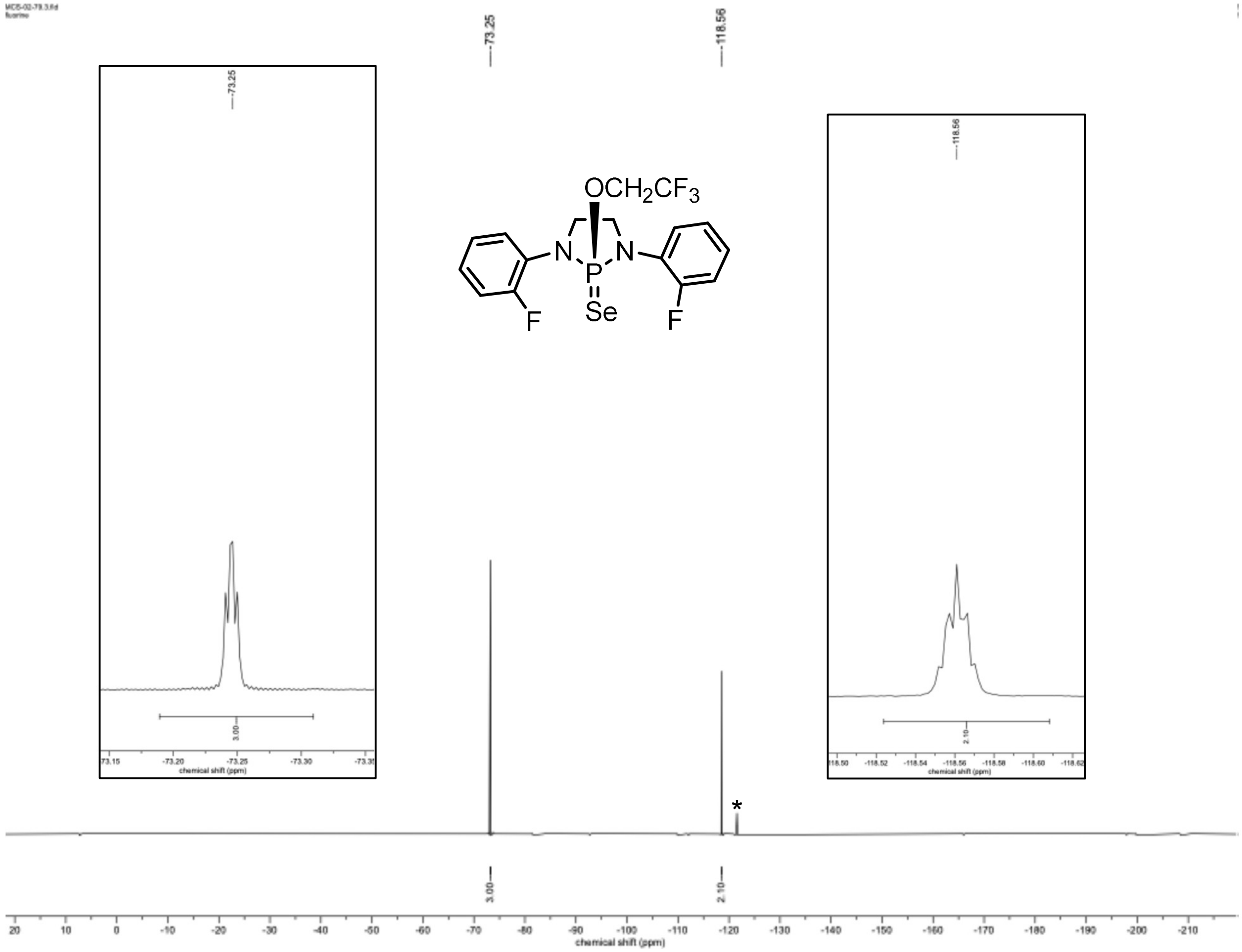

Figure S89: ^{19}F NMR ($CDCl_3$, 377 MHz) spectrum of (F(Se=)$P^{OCH2CF3}$F) (**43**). Unidentified minor impurity denoted with an asterisk (*).

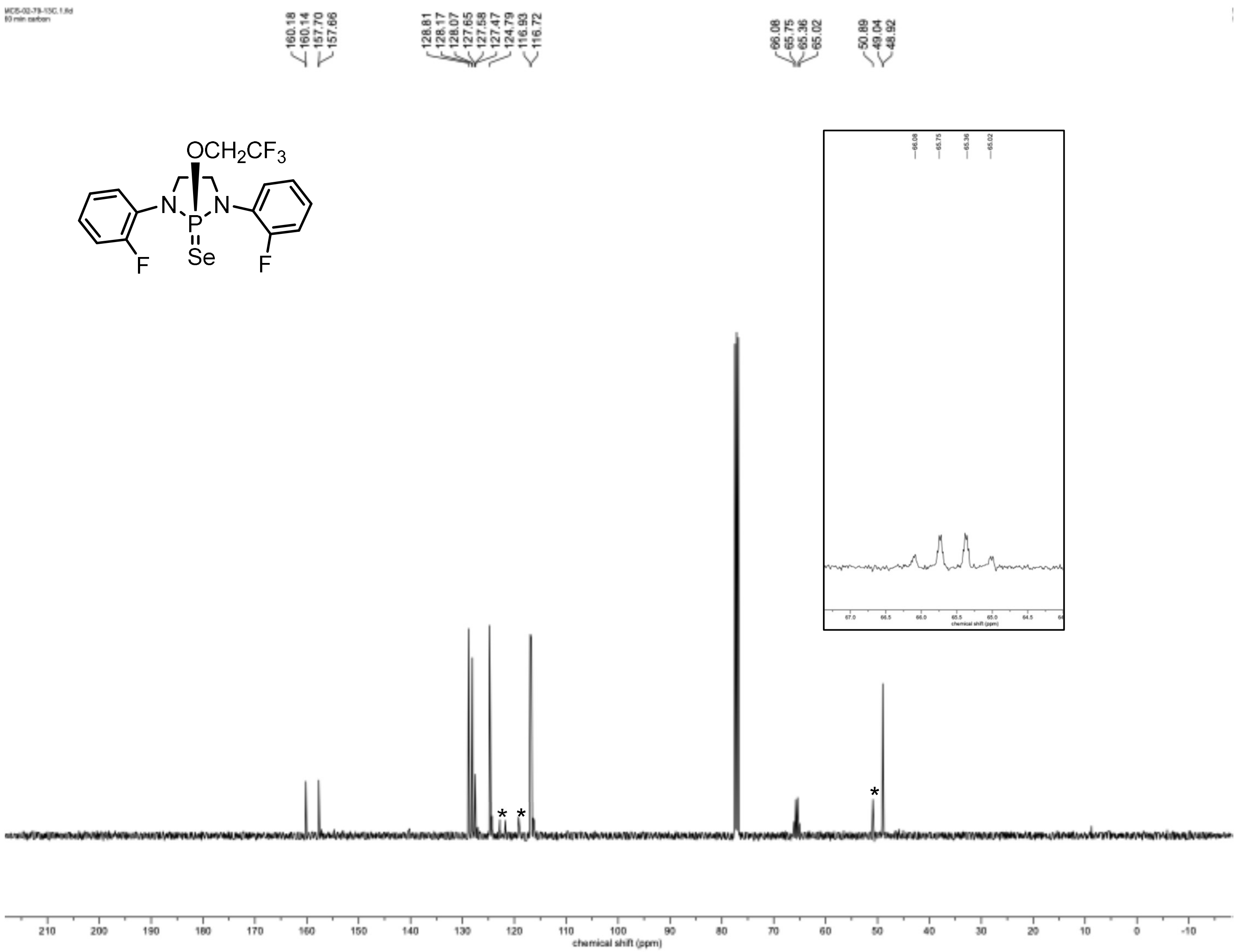

Figure S90: $^{13}C\{^{1}H\}$ NMR ($CDCl_3$, 151 MHz) spectrum of (F(Se=)$P^{OCH2CF3}$F) (**43**). Unidentified minor impurity denoted with an asterisk (*).

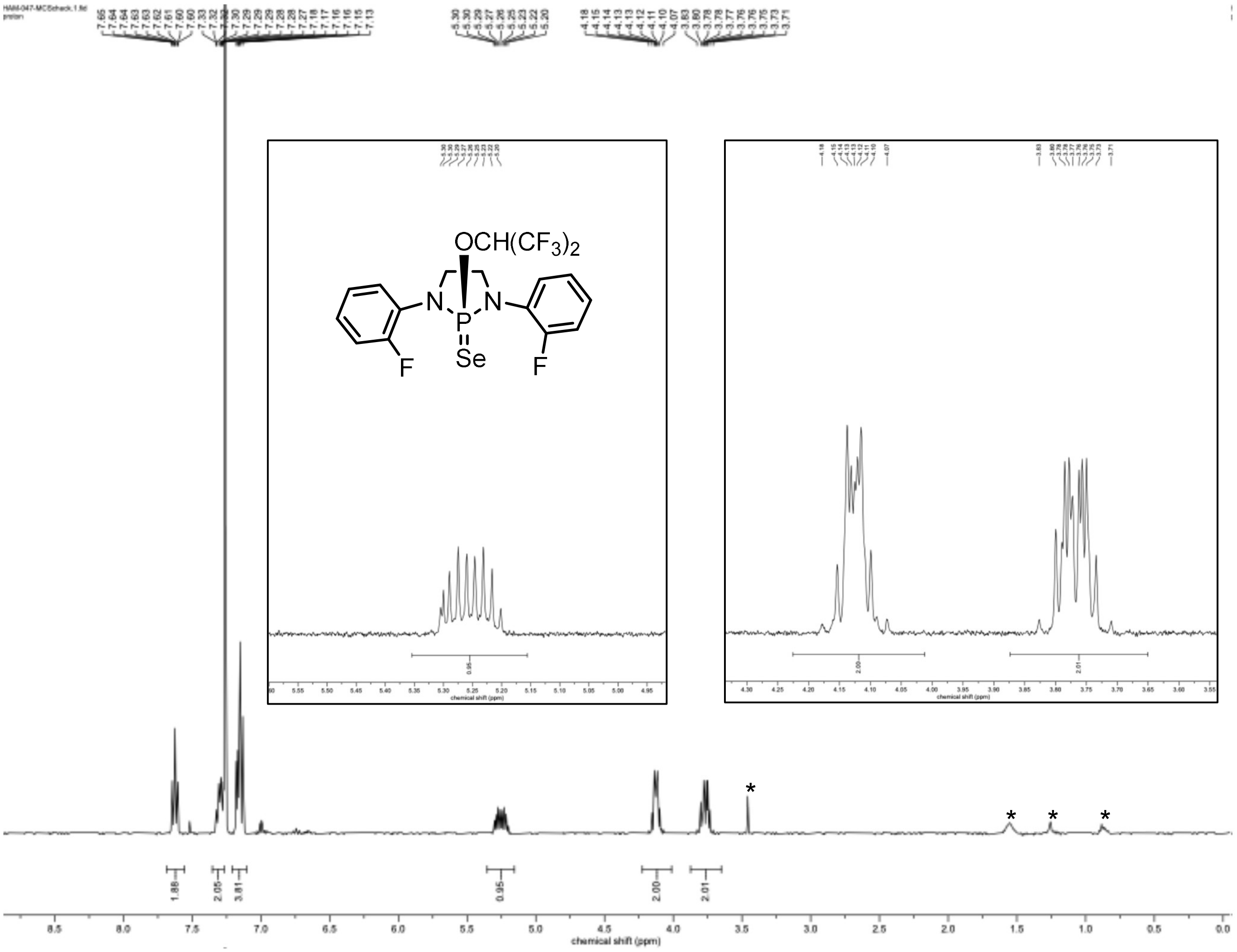

Figure S91: ^{1}H NMR ($CDCl_3$, 400 MHz) spectrum of (F(Se=)$P^{OCH(CF3)2}$F) (**44**). Residual solvent denoted with an asterisk (*)

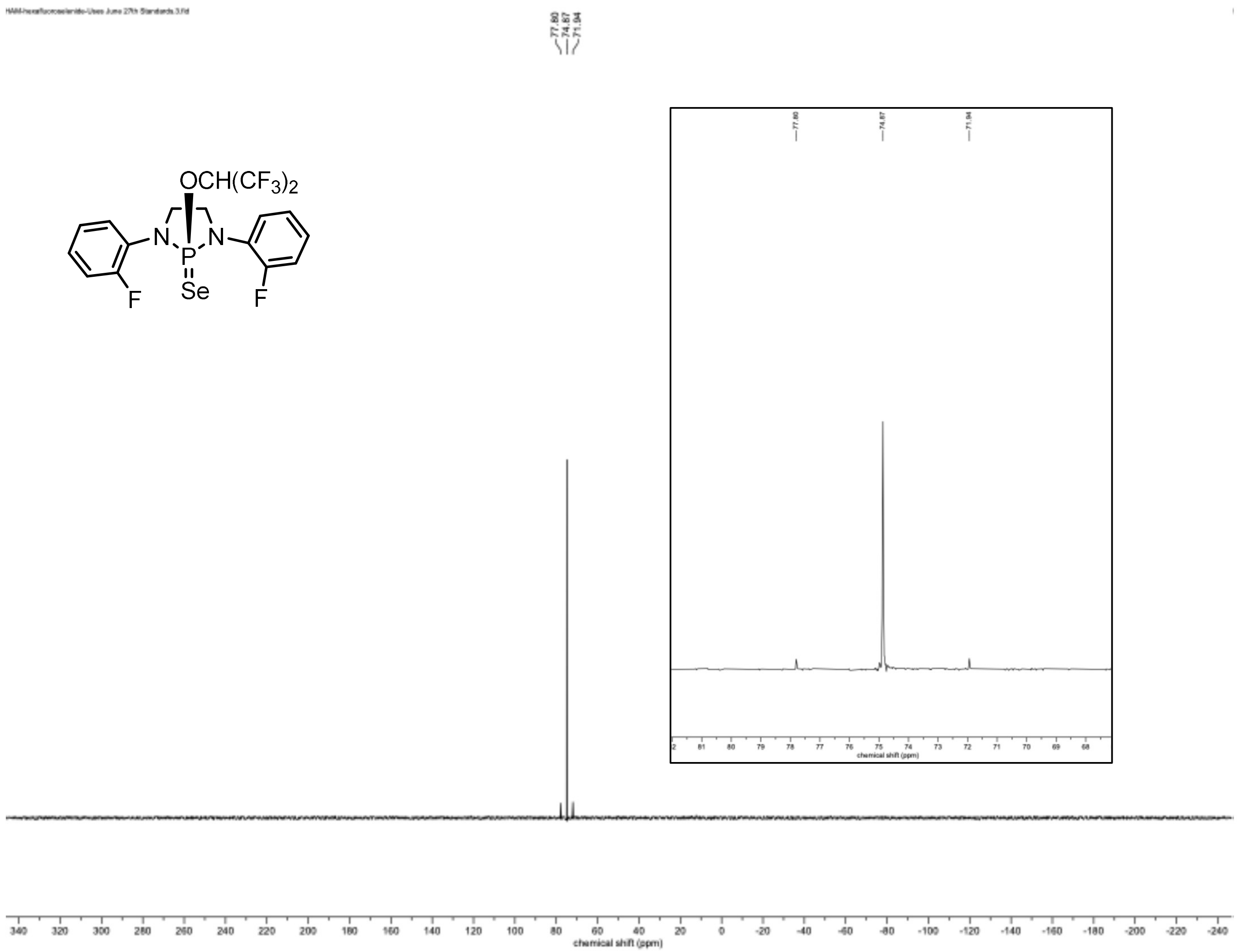

Figure S92: $^{31}P\{^1H\}$ NMR ($CDCl_3$, 162 MHz) spectrum of (F(Se=)$P^{OCH(CF3)2}$F) (**44**).

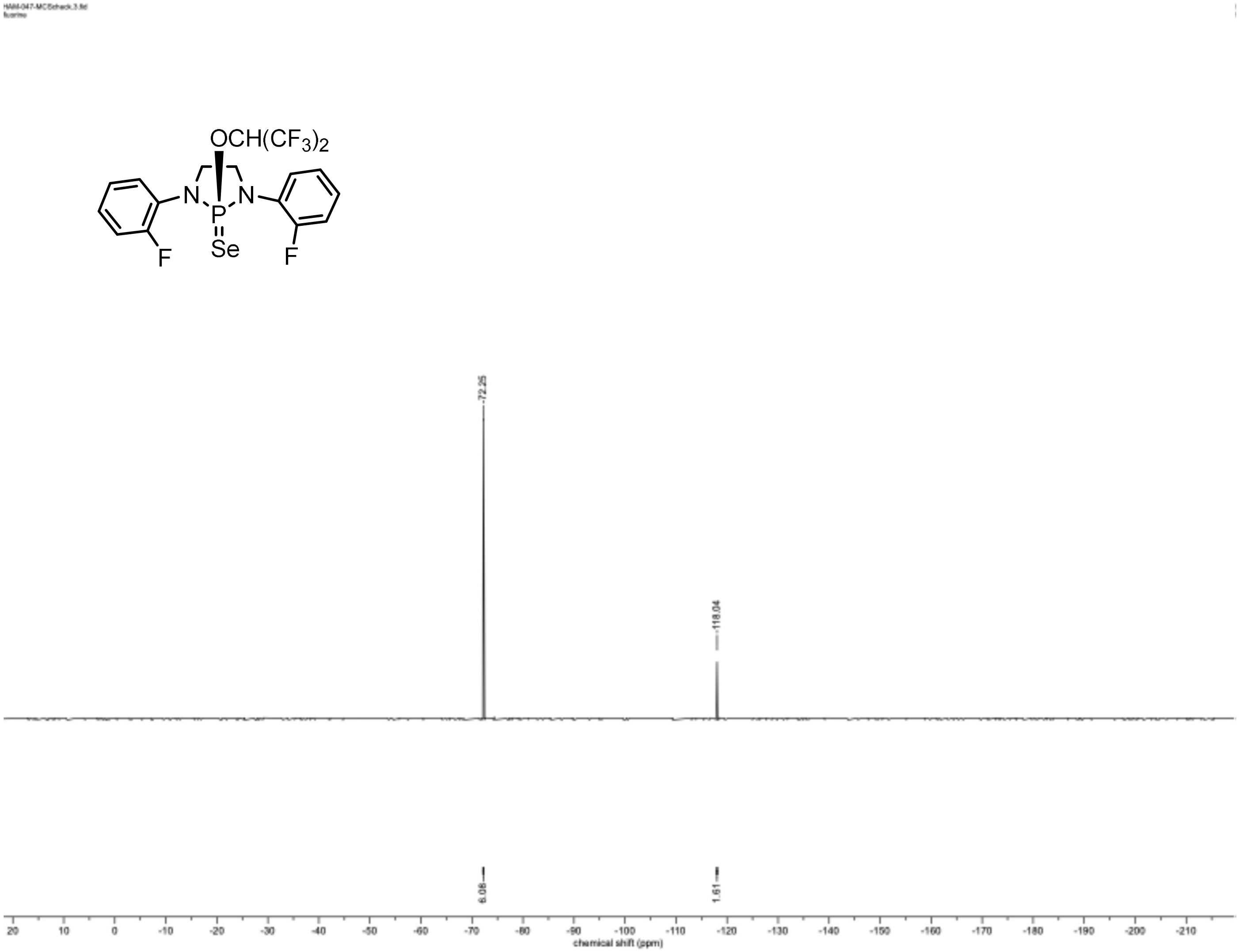

Figure S93: ^{19}F NMR ($CDCl_3$, 377 MHz) spectrum of (F(Se=)P$^{OCH(CF3)2}$F) (**44**).

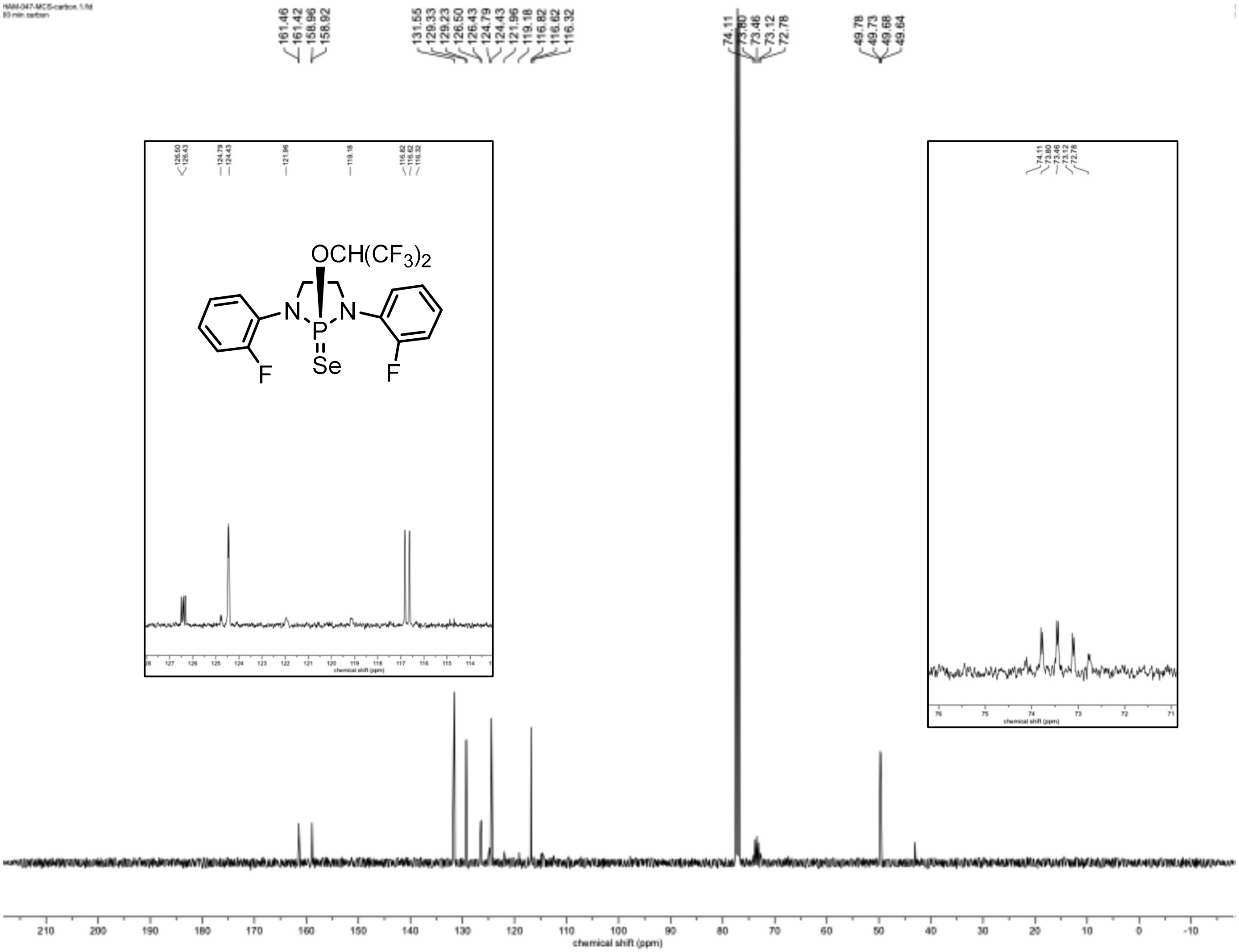

Figure S94: $^{13}C\{^{1}H\}$ NMR ($CDCl_3$, 151 MHz) spectrum of (F(Se=)$P^{OCH(CF3)2}$F) (**44**).

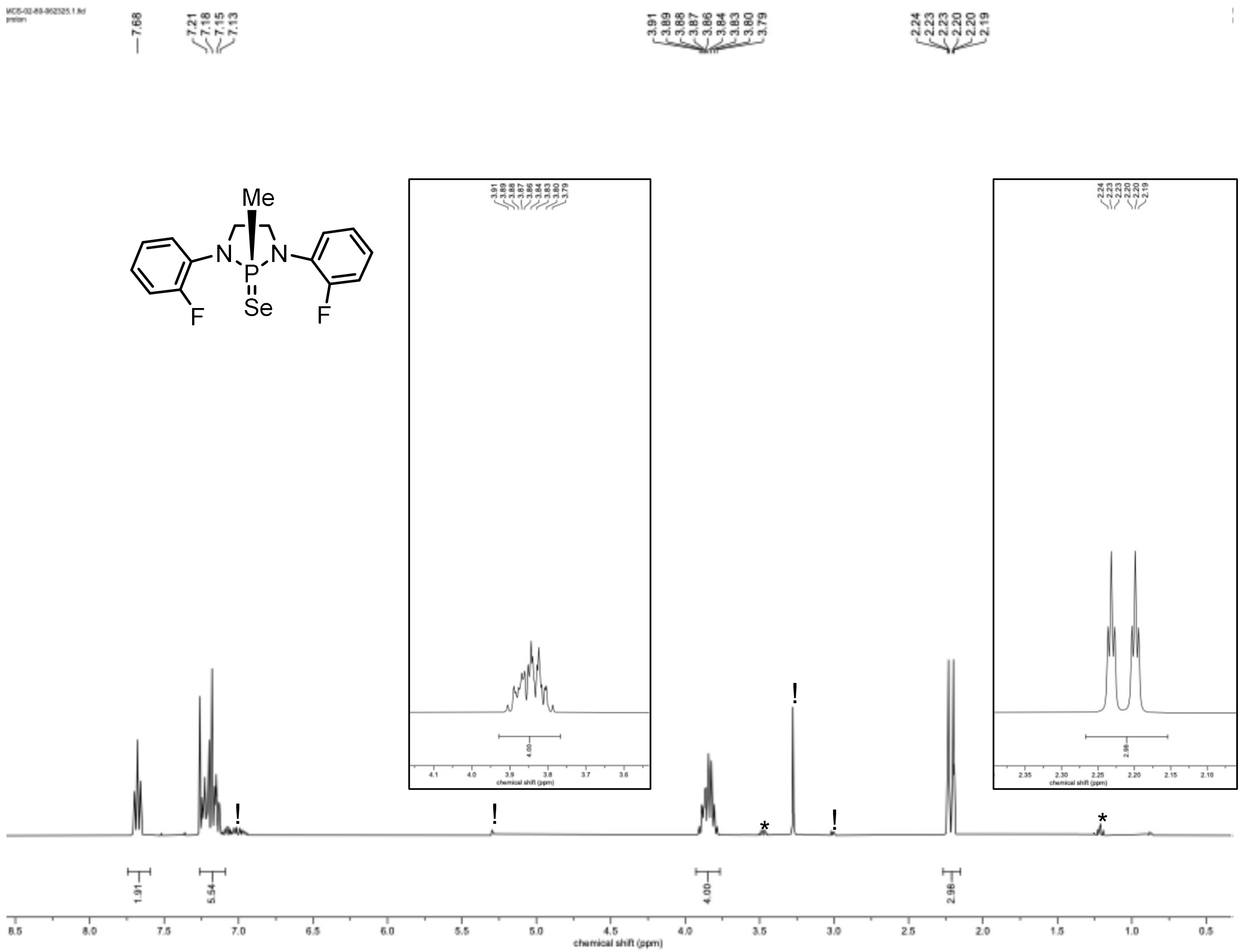

Figure S95: ^{1}H NMR ($CDCl_3$, 400 MHz) spectrum of (F(Se=)P^{Me}F) (**45**). Unidentified minor impurity denoted with an asterisk (*). Residual solvent denoted with an exclamation point (!).

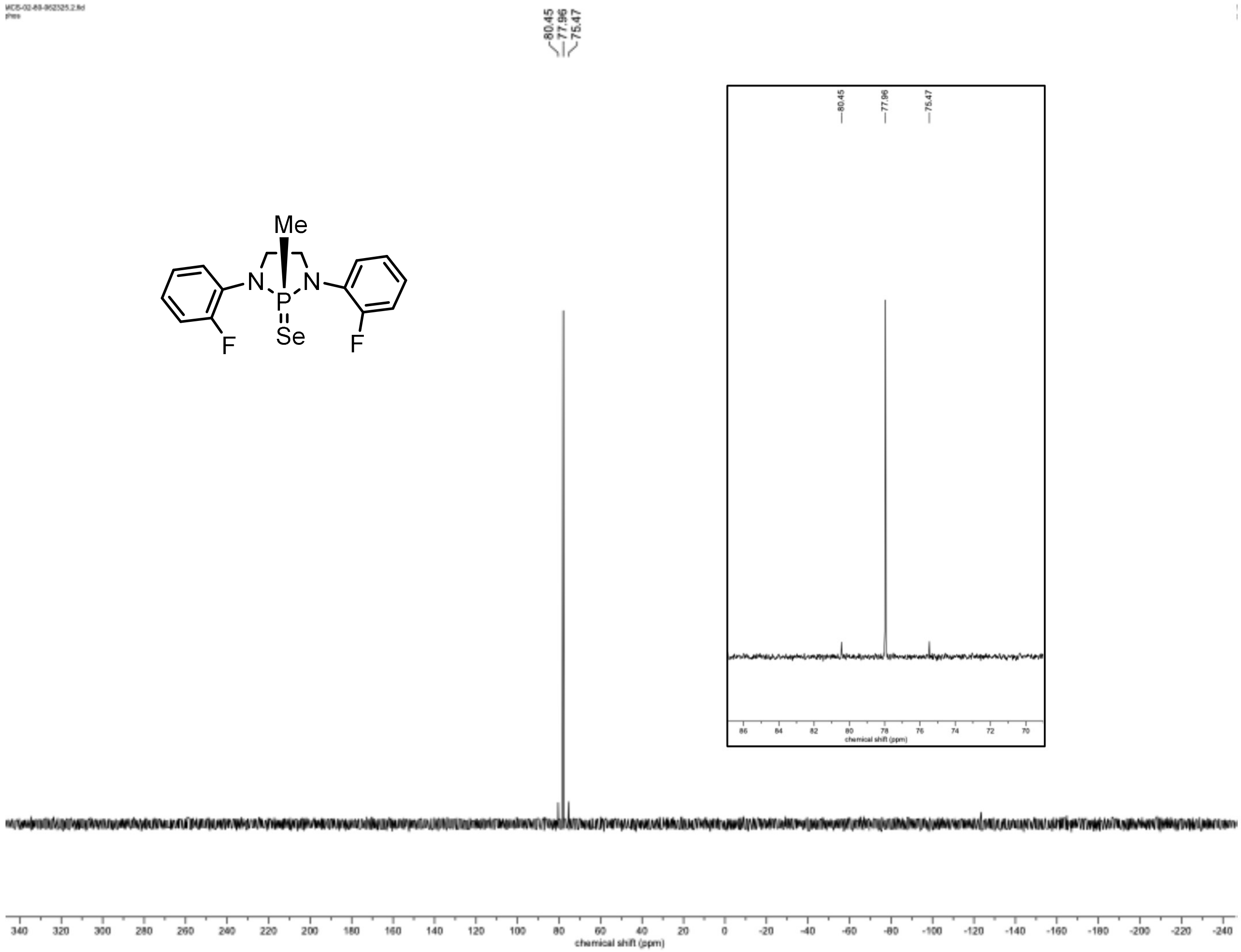

Figure S96: $^{31}P\{^1H\}$ NMR ($CDCl_3$, 162 MHz) spectrum of (F(Se=)P^{Me}F) (**45**).

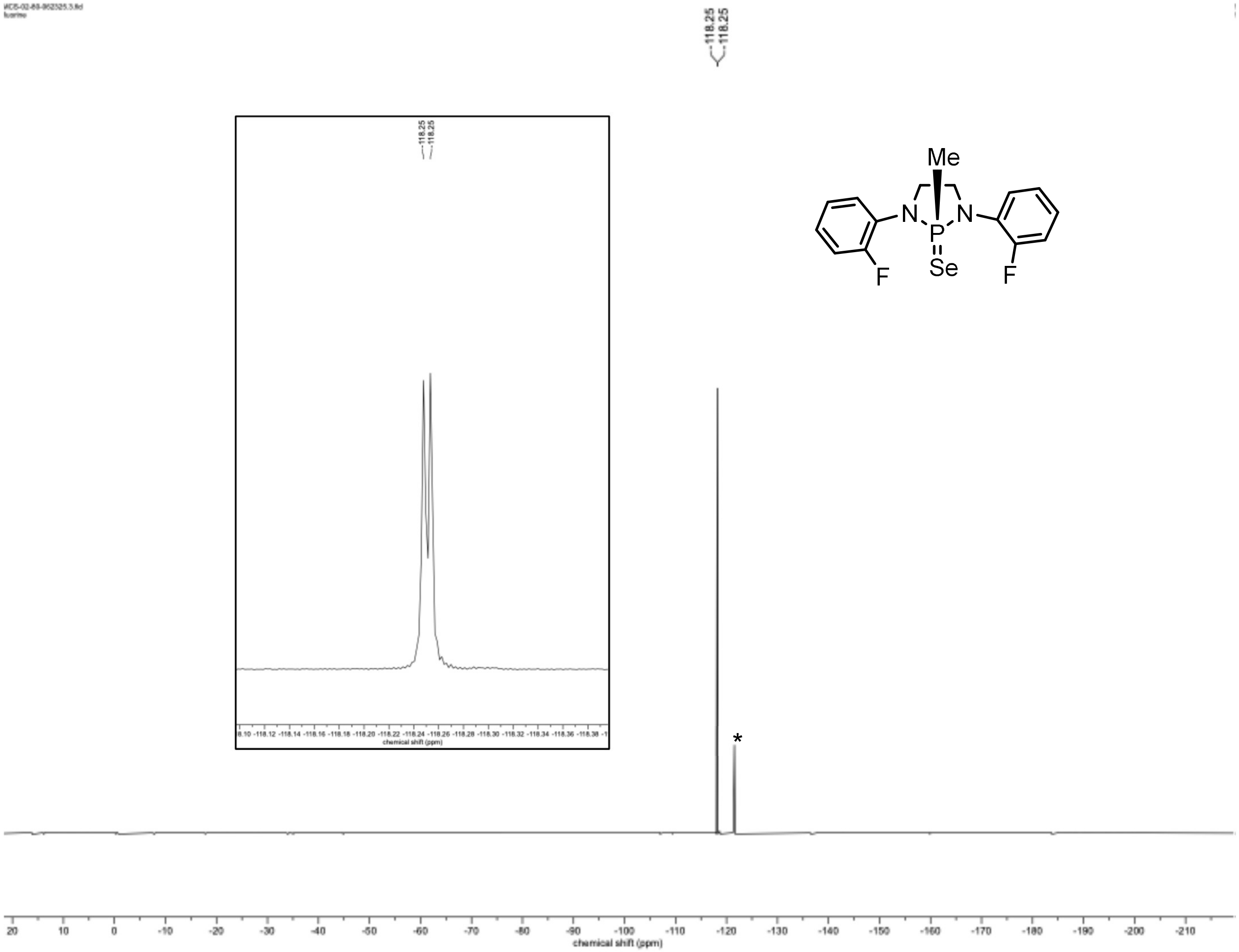

Figure S97: ^{19}F NMR ($CDCl_3$, 377 MHz) spectrum of (F(Se=)P^{Me}F) (**45**). Unidentified minor impurity denoted with an asterisk (*).

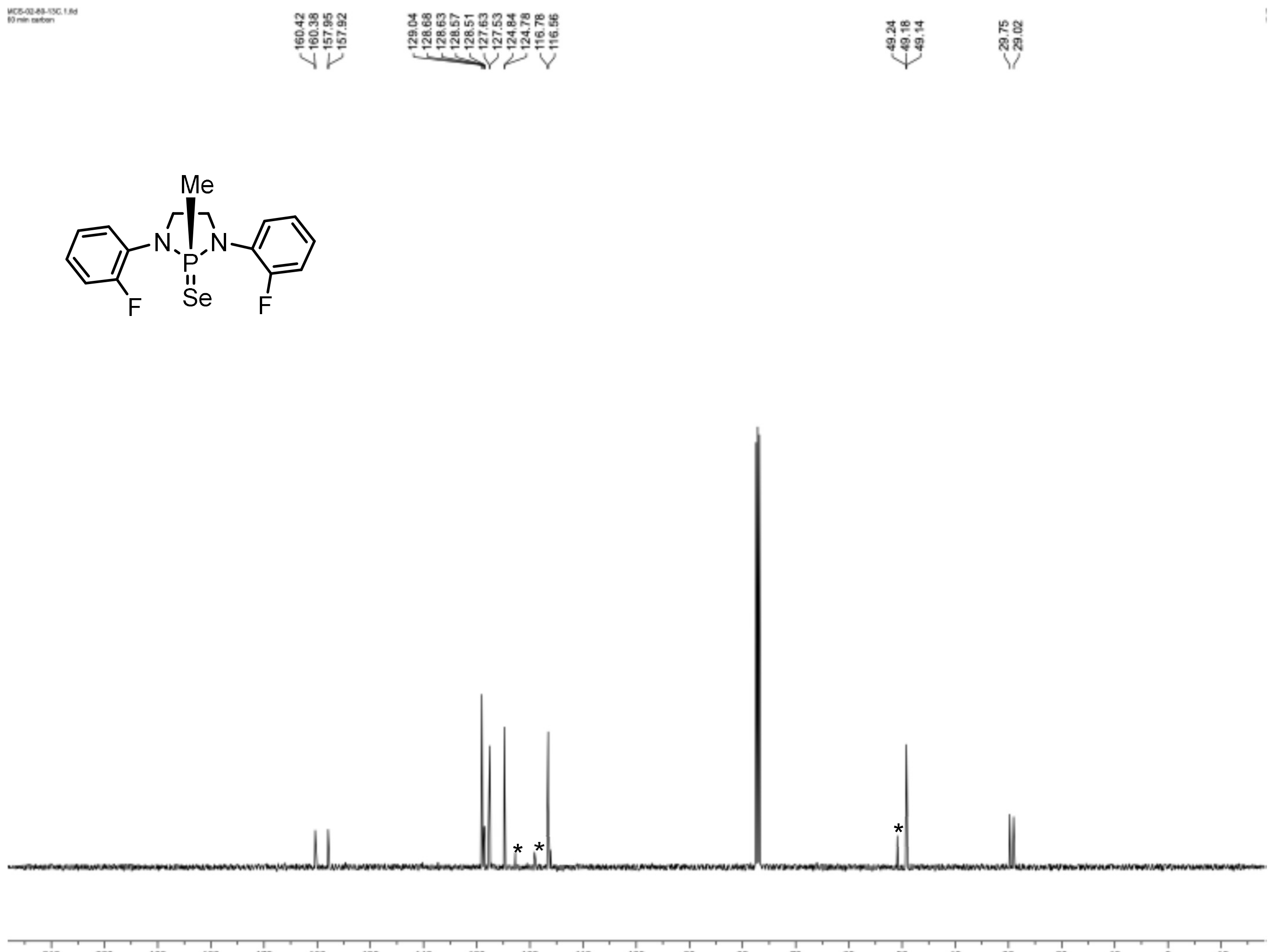

Figure S98: $^{13}C\{^{1}H\}$ NMR ($CDCl_3$, 151 MHz) spectrum of (F(Se=)P^{Me}F) (**45**). Unidentified minor impurity denoted with an asterisk (*).

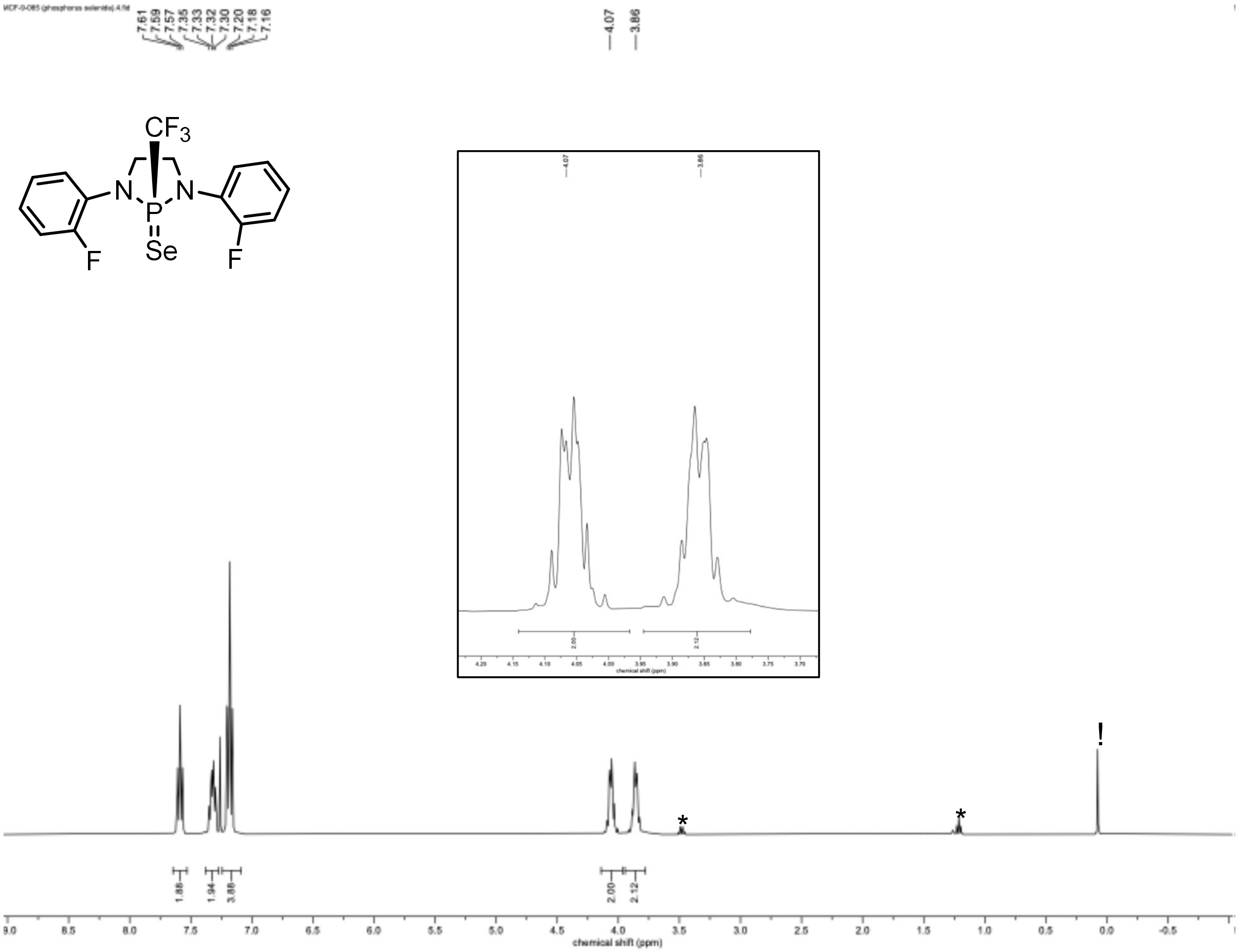

Figure S99: ^{1}H NMR (CDCl$_3$, 400 MHz) spectrum of (F(Se=)P^{CF3}F) (**46**). Residual solvent denoted with an asterisk (*). Silicone grease denoted with an exclamation point (!).

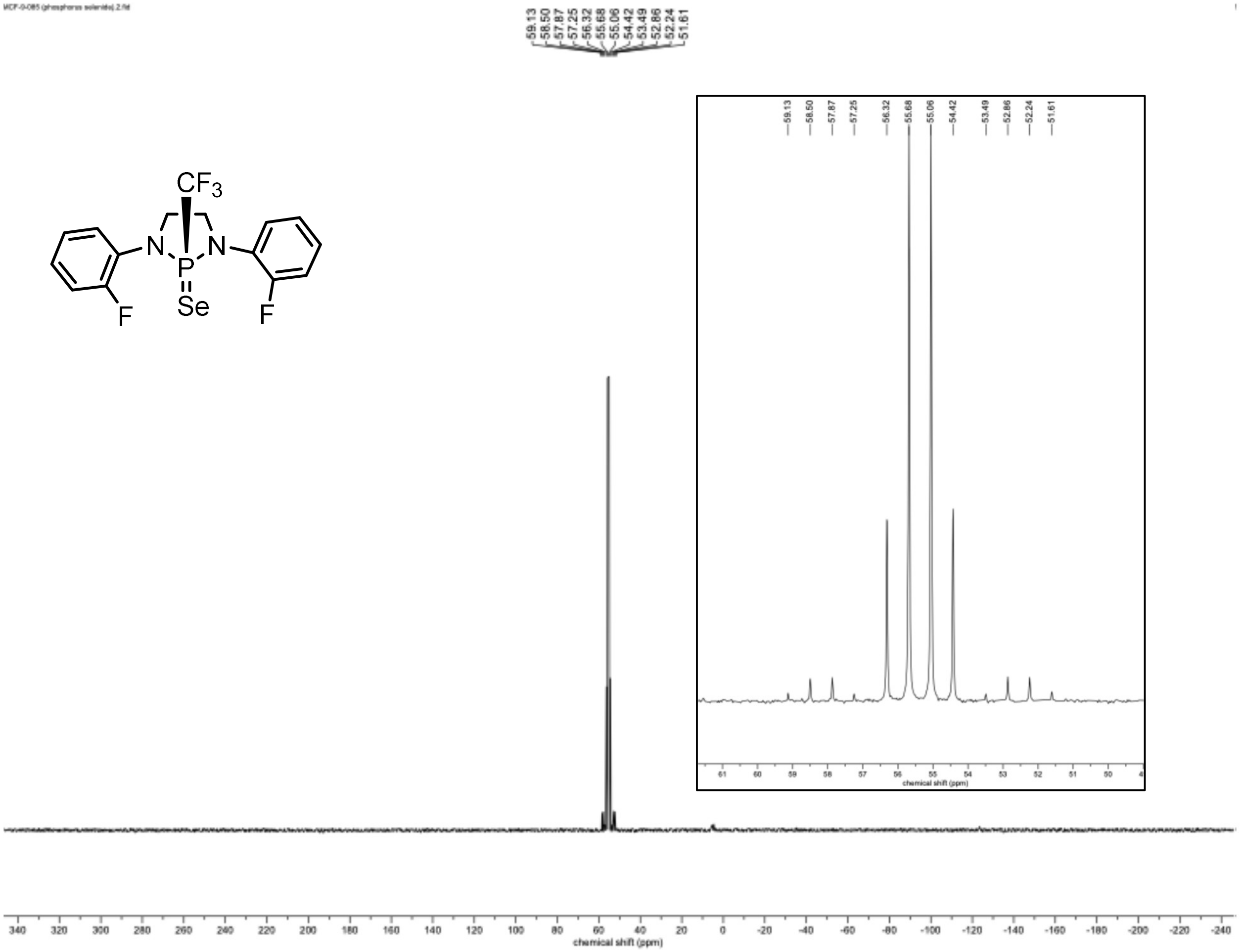

Figure S100: $^{31}P\{^{1}H\}$ NMR ($CDCl_3$, 162 MHz) spectrum of ($F(Se{=})P^{CF3}F$) (**46**).

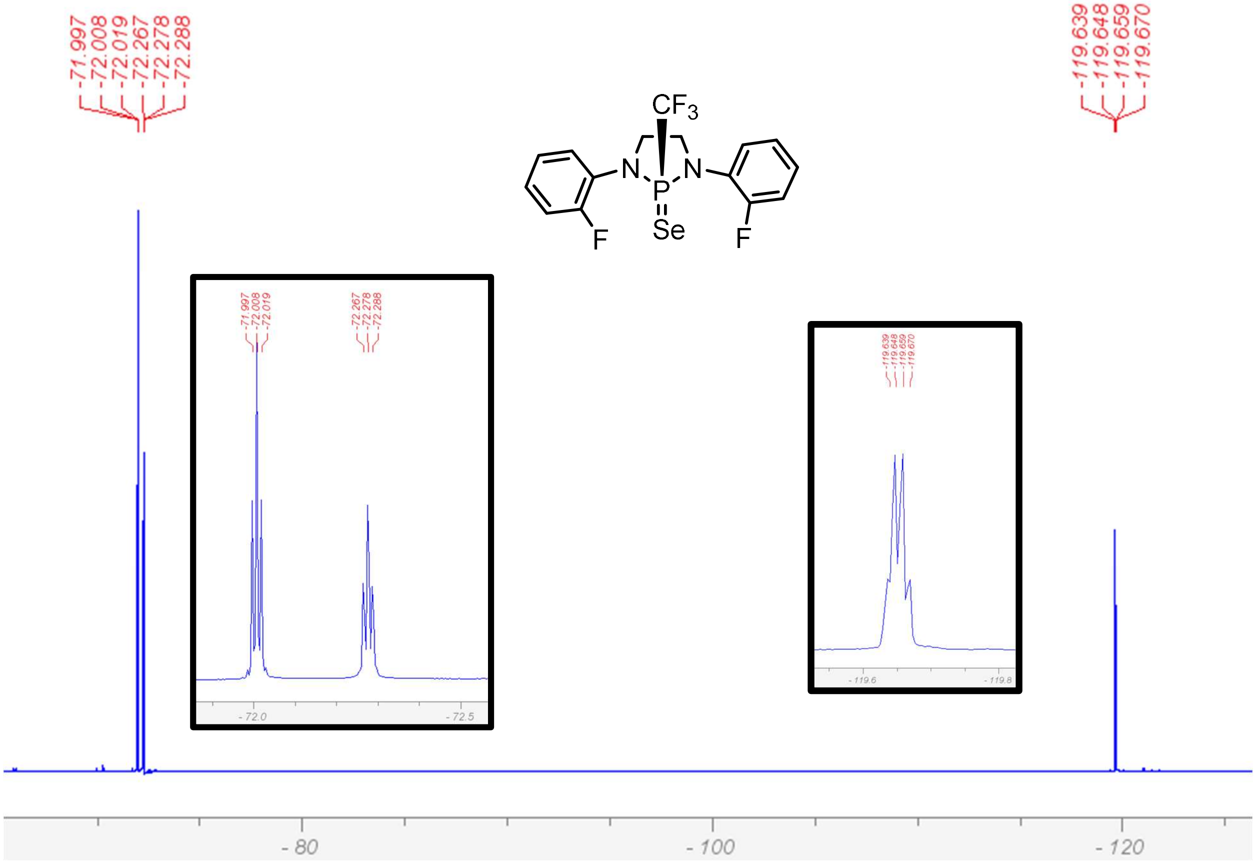

Figure S101: ^{19}F NMR ($CDCl_3$, 377 MHz) spectrum of (F(Se=)P^{CF3}F) (**46**).

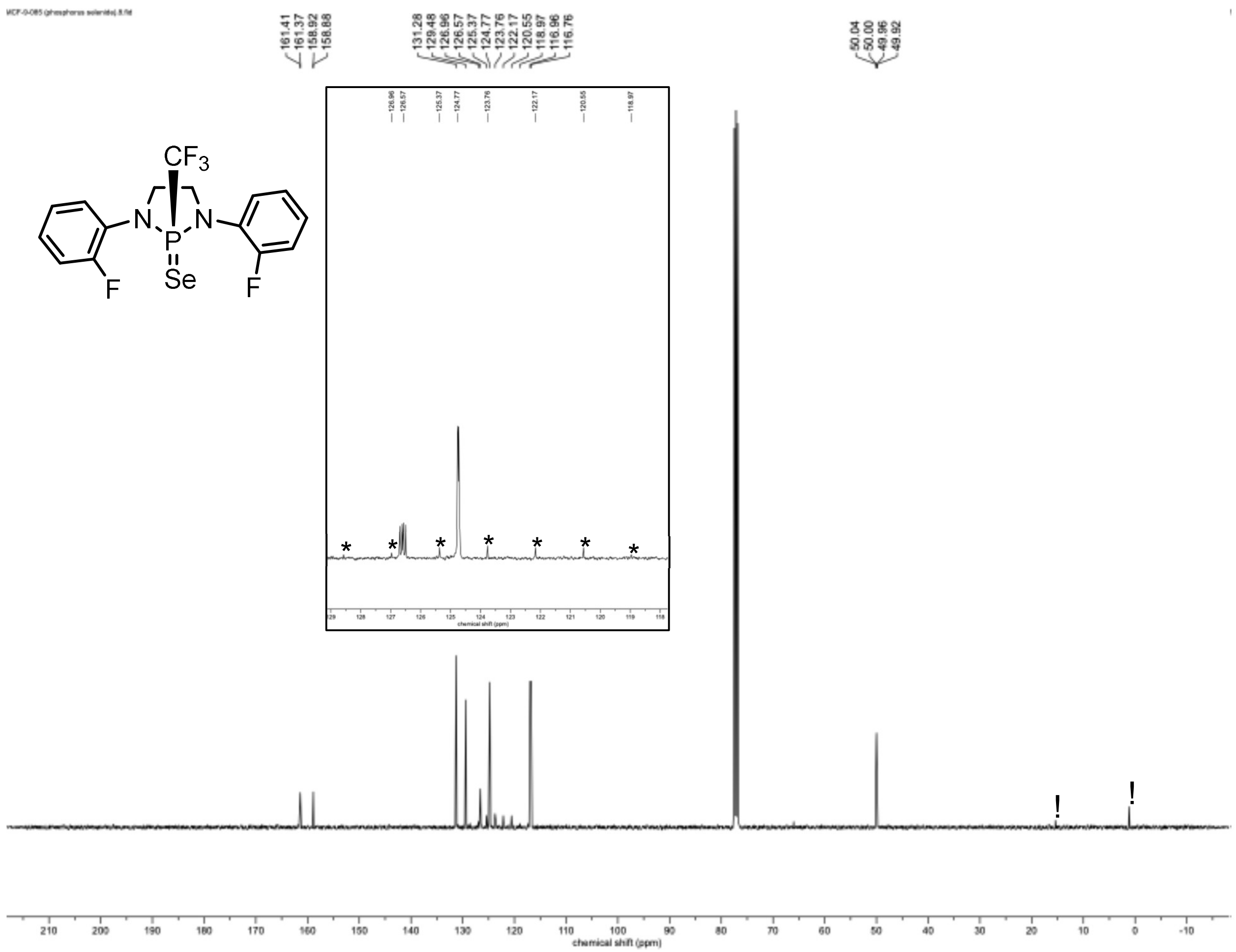

Figure S102: $^{13}C\{^1H\}$ NMR ($CDCl_3$, 151 MHz) spectrum of (F(Se=)P^{CF3}F) (**46**). The CF_3 carbon resonances are denoted with asterisks (*). Residual solvent denoted with exclamation points (!).

Cyclic Voltammetry Data

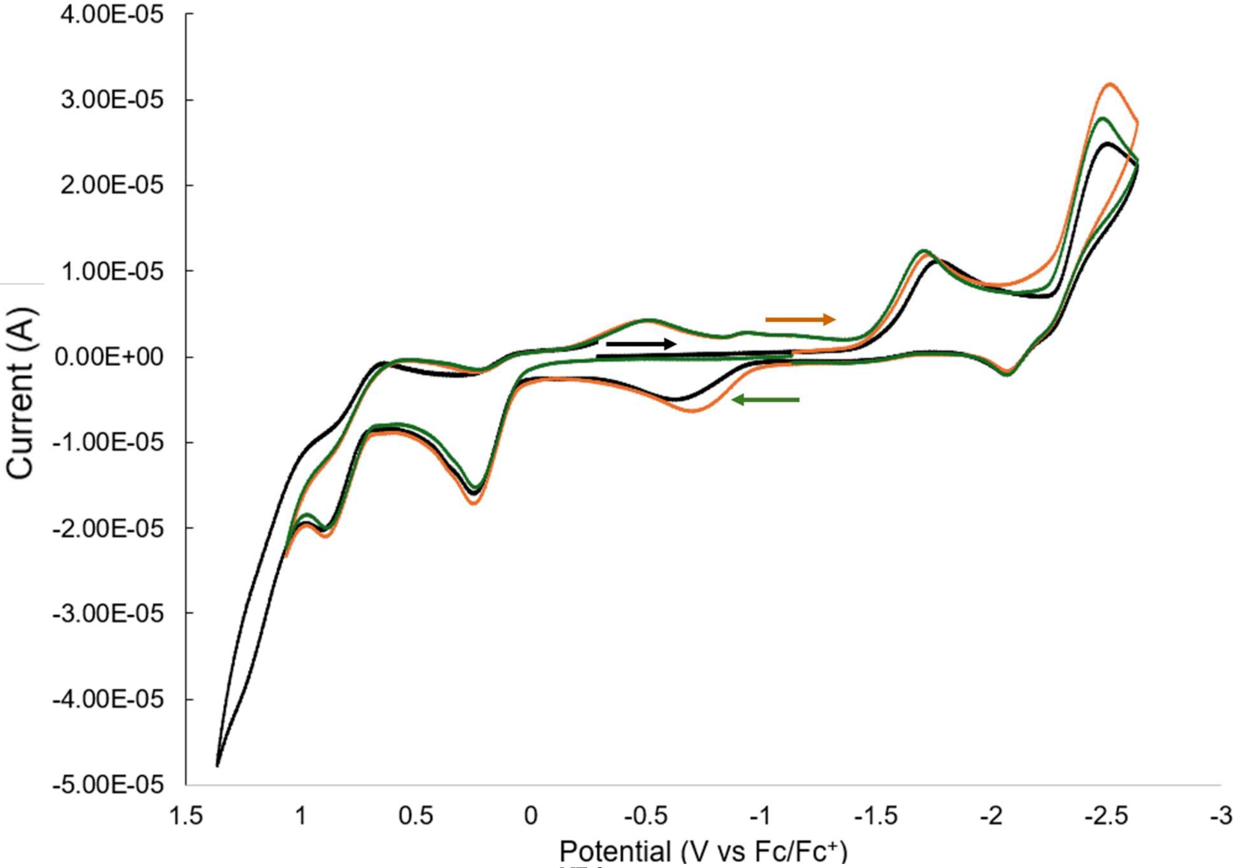

Figure S103: Cyclic voltammograms of $(PP^{NEt2}P)CoI_2$ (**13**) in different potential ranges and scan directions, as indicated by the arrows. Solvent = THF, temperature = 295 K, scan rate = 0.1 V/s, $[[^nBu_4N][PF_6]]$ = 100 mM.

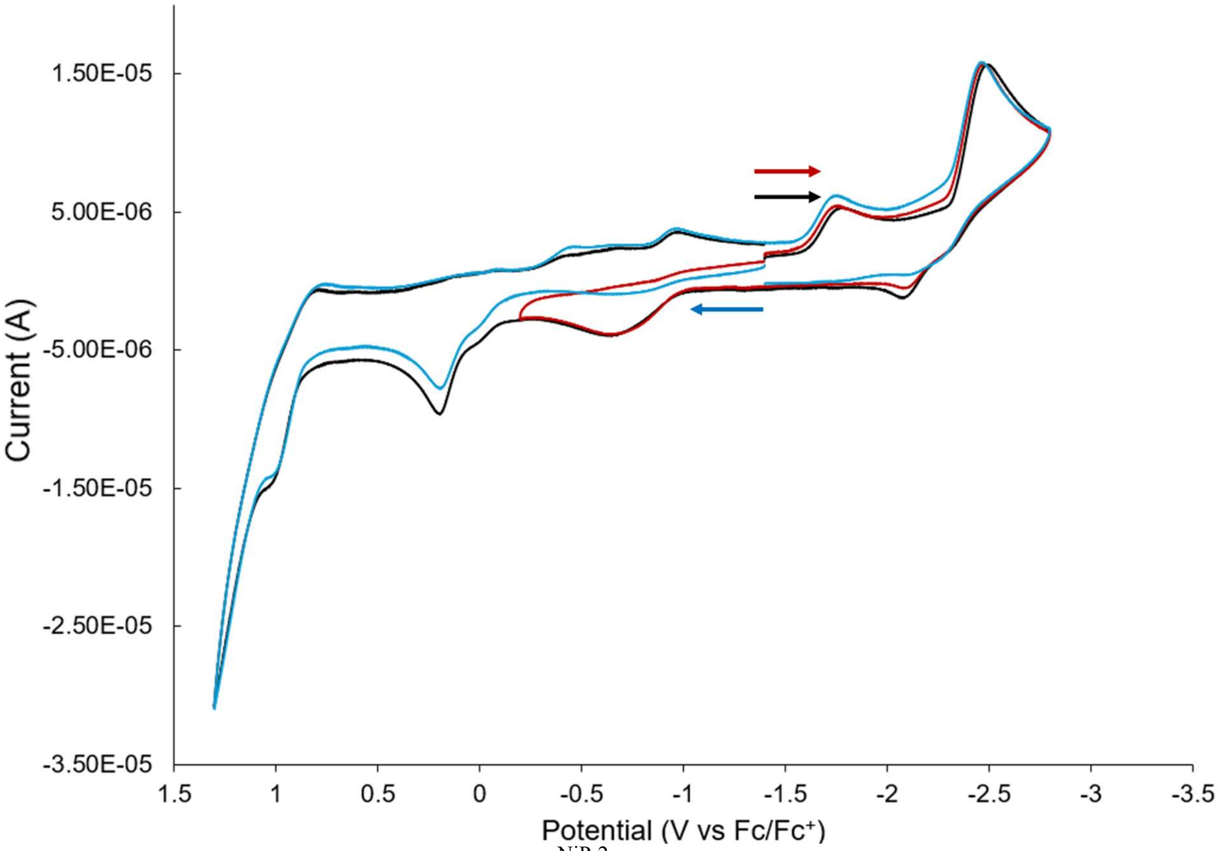

Figure S104: Cyclic voltammograms of $(PP^{NiPr2}P)CoI_2$ (**14**) in different potential ranges and scan directions, as indicated by the arrows. Solvent = THF, temperature = 295 K, scan rate = 0.1 V/s, $[[^{n}Bu_4N][PF_6]]$ = 100 mM.

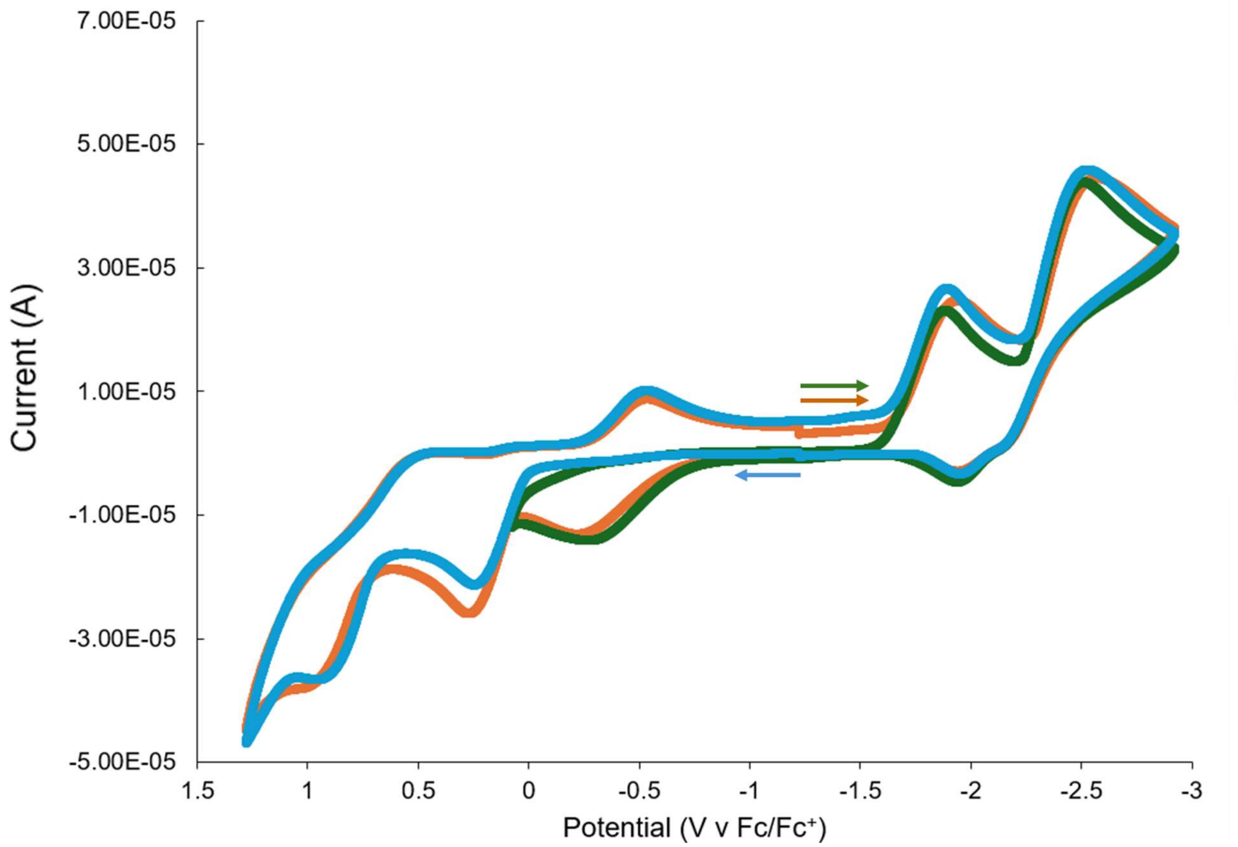

Figure S105: Cyclic voltammograms of (PP^{OEt}P)CoI$_2$ (**15**) in different potential ranges and scan directions, as indicated by the arrows. Solvent = THF, temperature = 295 K, scan rate = 0.1 V/s, [[^{n}Bu$_4$N][PF$_6$]] = 100 mM.

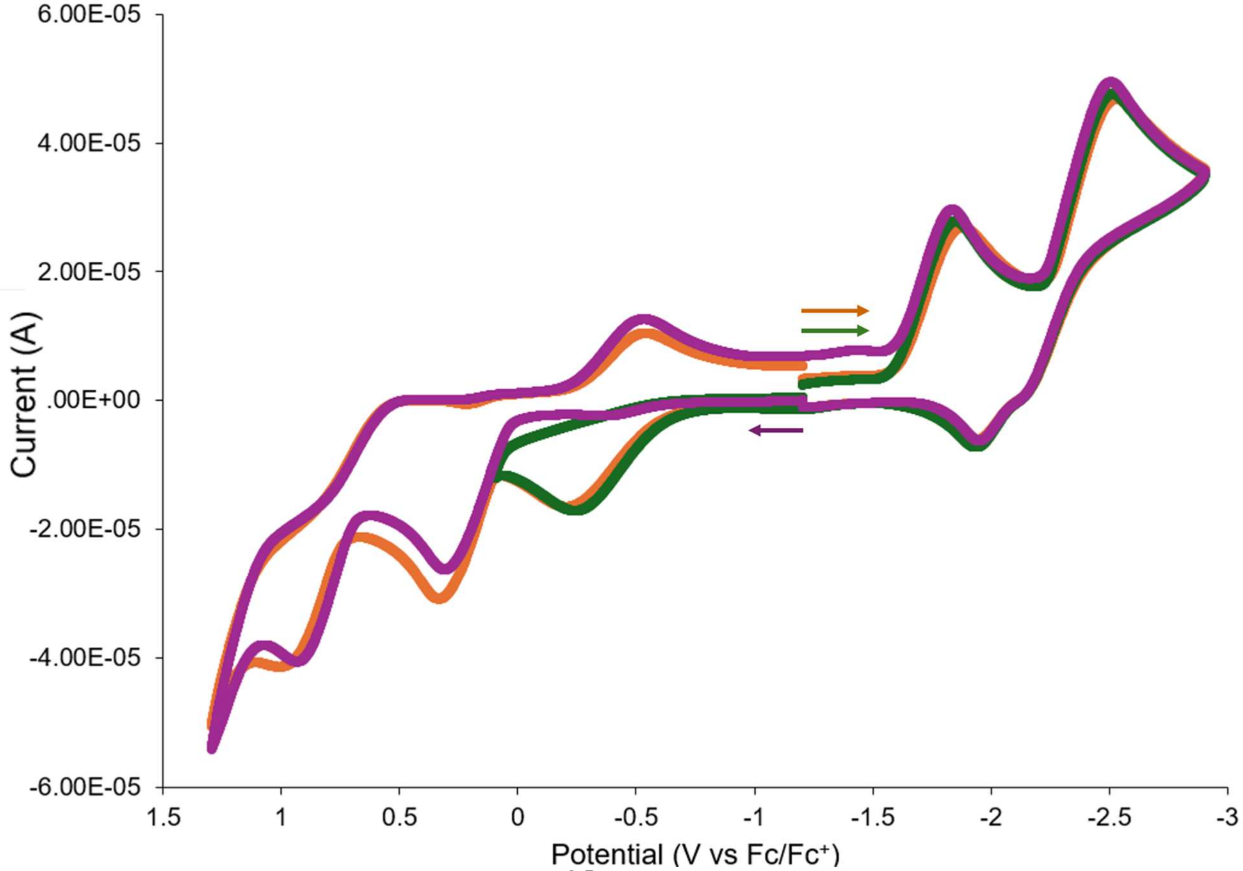

Figure S106: Cyclic voltammogram*s* of $(PP^{O\textit{i}Pr}P)CoI_2$ (**16**) in different potential ranges and scan directions, as indicated by the arrows. Solvent = THF, temperature = 295 K, scan rate = 0.1 V/s, $[[^nBu_4N][PF_6]]$ = 100 mM.

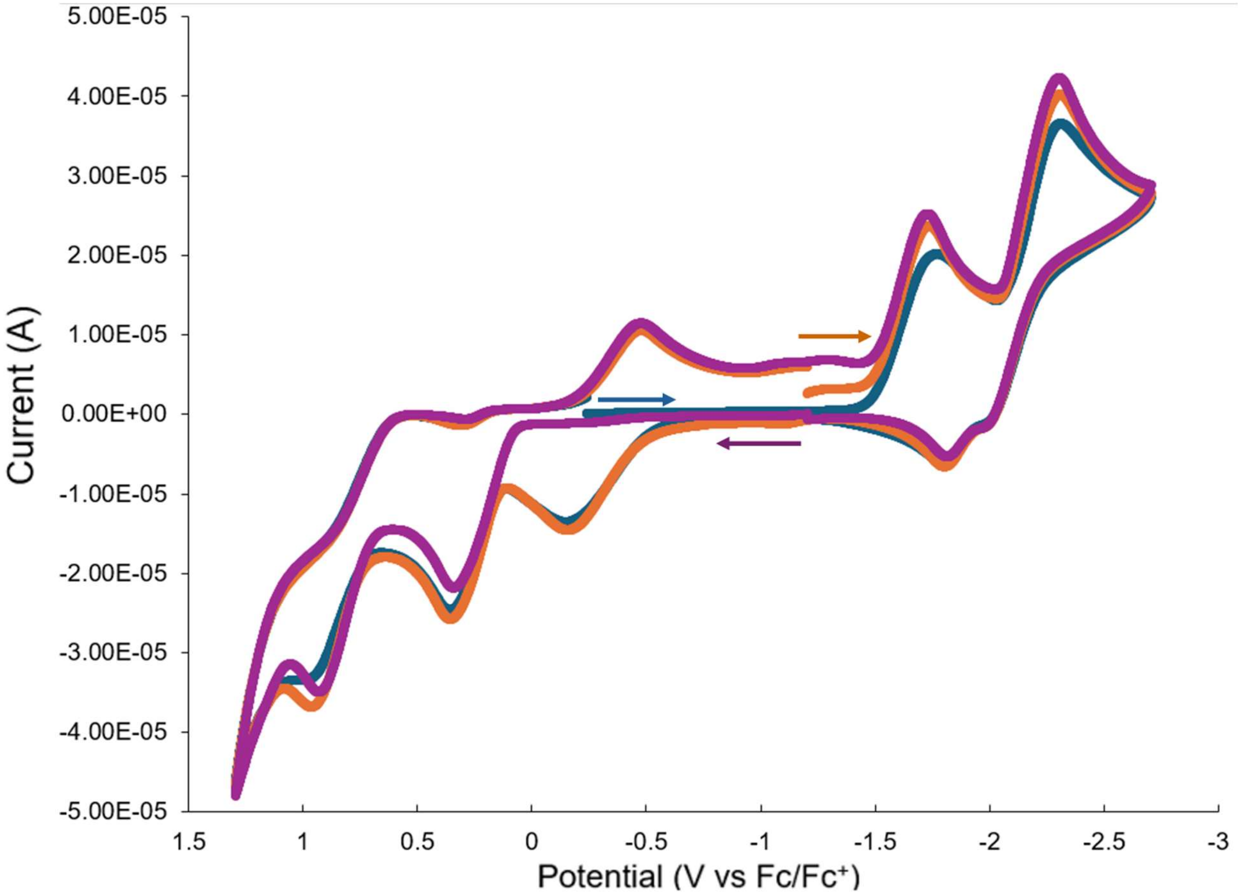

Figure S107: Cyclic voltammogram*s* of (PP^{OCH2CF3}P)CoI$_2$ (**17**) in different potential ranges and scan directions, as indicated by the arrows. Solvent = THF, temperature = 295 K, scan rate = 0.1 V/s, [[^{n}Bu$_4$N][PF$_6$]] = 100 mM.

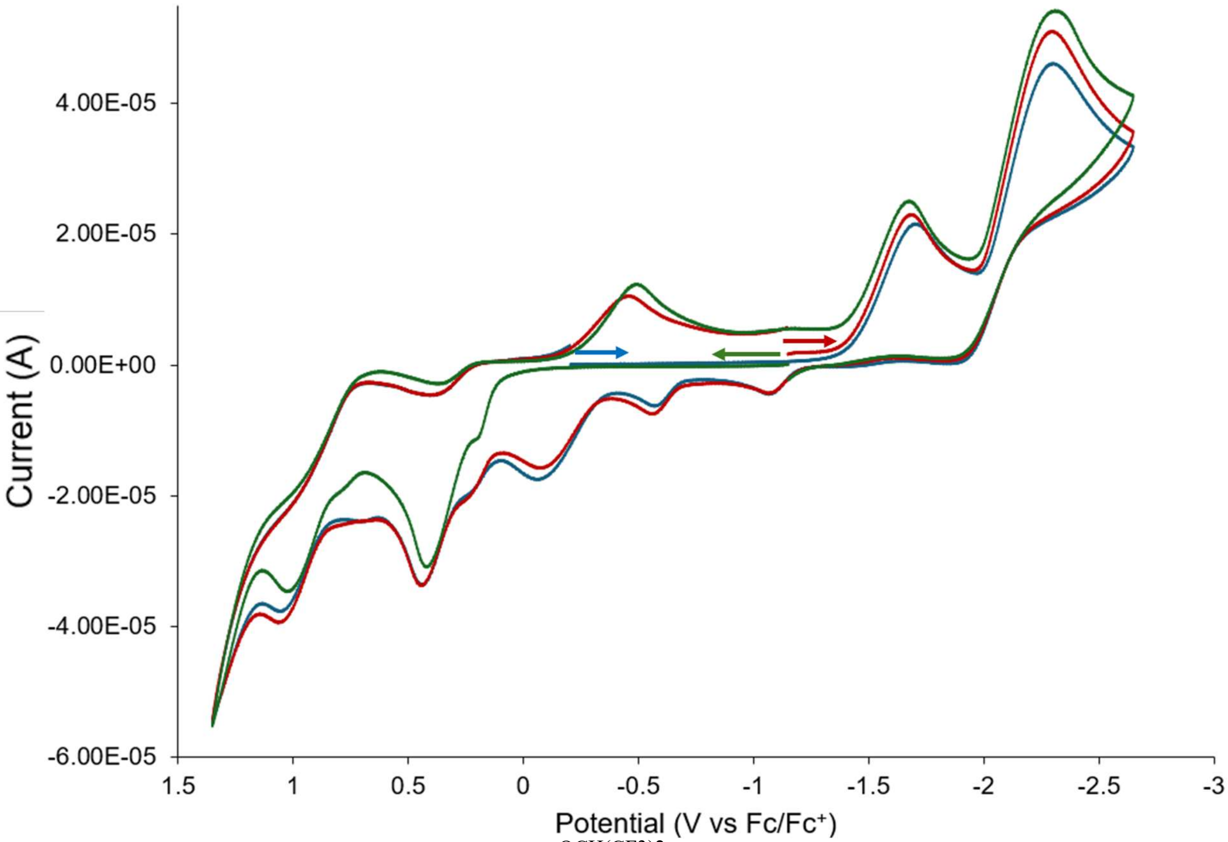

Figure S108: Cyclic voltammograms of (PP$^{OCH(CF3)2}$P)CoI$_2$ (**18**) in different potential ranges and scan directions, as indicated by the arrows. Solvent = THF, temperature = 295 K, scan rate = 0.1 V/s, [[^{n}Bu$_4$N][PF$_6$]] = 100 mM.

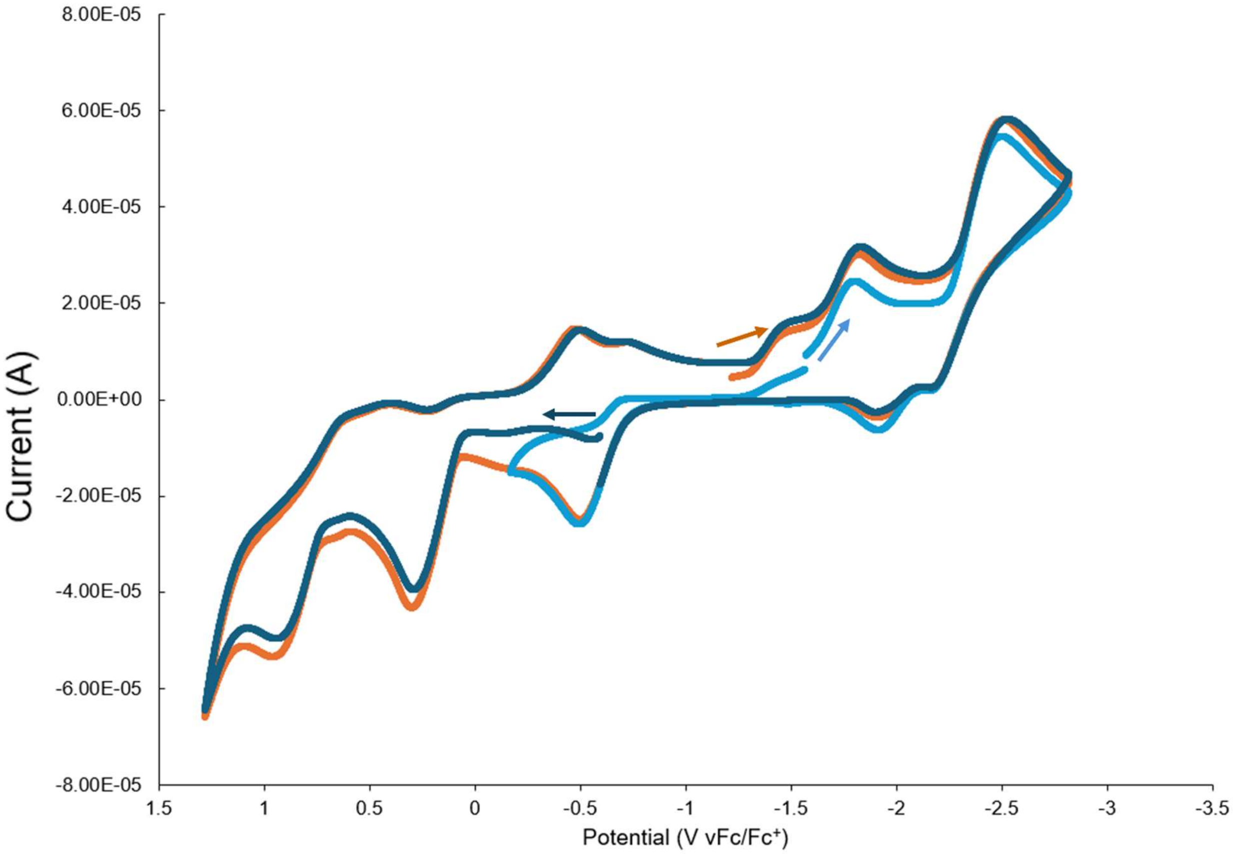

Figure S109: Cyclic voltammograms of (PP^{menthoxide}P)CoI_2 (**19**) in different potential ranges and scan directions, as indicated by the arrows. Solvent = THF, temperature = 295 K, scan rate = 0.1 V/s, [[$^nBu_4N$][PF_6]] = 100 mM.

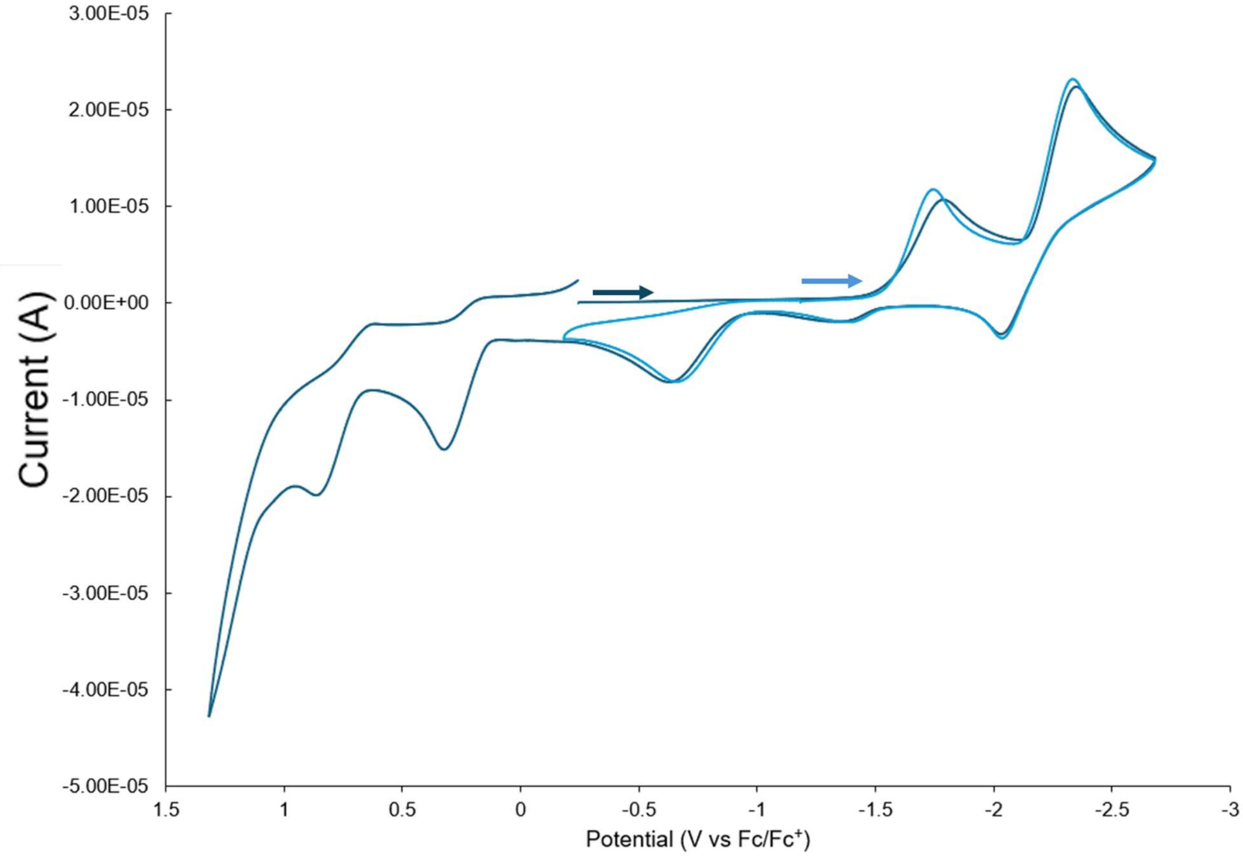

Figure S110: Cyclic voltammogram*s* of $(PP^{Me}P)CoI_2$ (**20**) in different potential ranges and scan directions, as indicated by the arrows. Solvent = THF, temperature = 295 K, scan rate = 0.1 V/s, $[[^nBu_4N][PF_6]]$ = 100 mM.

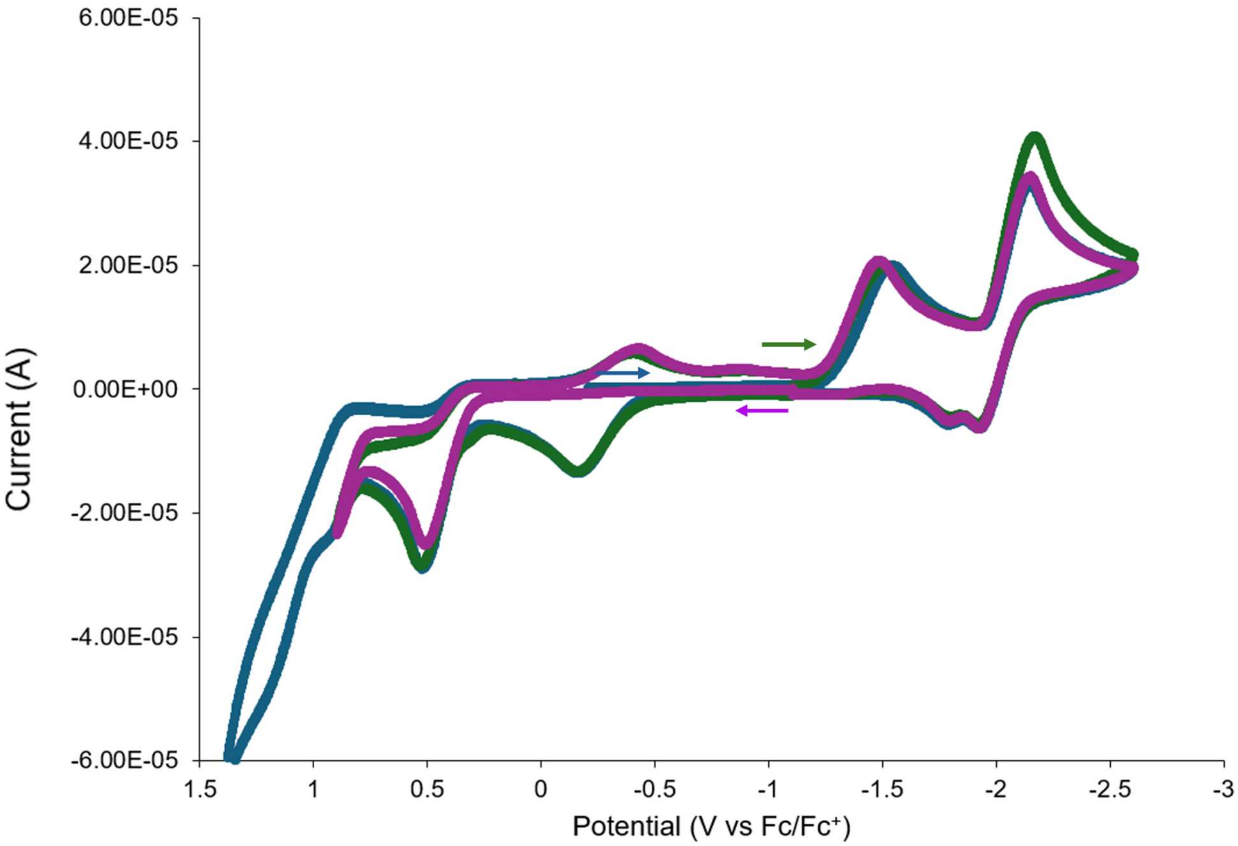

Figure S111: Cyclic voltammogram*s* of ($PP^{CF3}P$)CoI_2 (**3**) in different potential ranges and scan directions, as indicated by the arrows. Solvent = THF, temperature = 295 K, scan rate = 0.1 V/s, [[$^{n}Bu_4N$][PF_6]] = 100 mM.

EPR Data

Table S1: g_1, g_2, g_3, and g_{iso} values for compounds **3** and **13-20** obtained from simulation of low temperature (40 K) EPR spectra collected in frozen THF without accounting for hyperfine or superhyperfine coupling.

**Complex**	g_1	g_2	g_3	g_{iso}
$(PP^{NEt2}P)CoI_2$ (**13**)	2.21	2.21	2.04	2.15
$(PP^{N\textit{i}Pr2}P)CoI_2$ (**14**)	2.21	2.21	2.04	2.15
$(PP^{OEt}P)CoI_2$ (**15**)	2.20	2.20	2.03	2.14
$(PP^{O\textit{i}Pr}P)CoI_2$ (**16**)	2.20	2.20	2.03	2.14
$(PP^{OCH2CF3}P)CoI_2$ (**17**)	2.20	2.20	2.03	2.14
$(PP^{OCH(CF3)2}P)CoI_2$ (**18**)	2.20	2.20	2.03	2.14
$(PP^{menthoxide}P)CoI_2$ (**19**)	2.21	2.21	2.03	2.15
$(PP^{Me}P)CoI_2$ (**20**)	2.20	2.20	2.03	2.14
$(PP^{CF3}P)CoI_2$ (**3**)	2.20	2.20	2.03	2.14

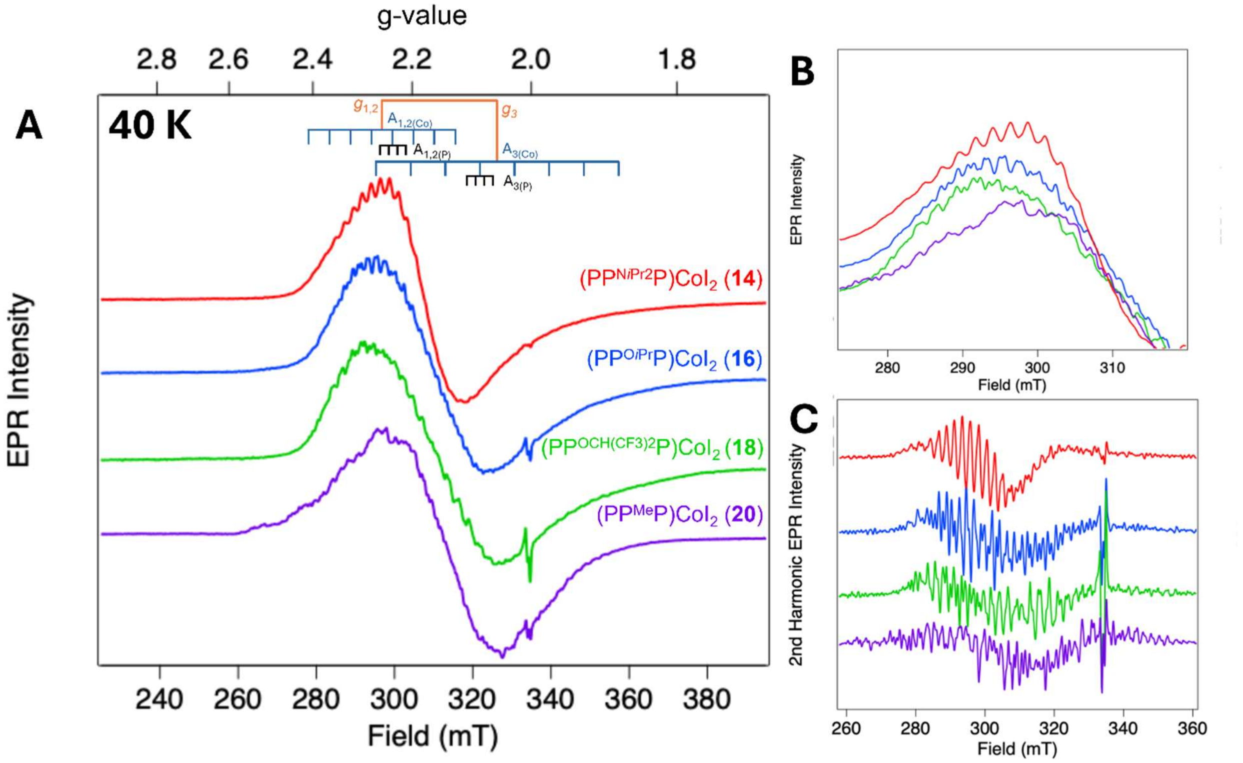

Figure S112. (A) X-band EPR spectra of **14** (red), **16** (blue), **18** (green), and **20** (purple) collected in frozen THF at 40 K, (B) Expansion of the ~g = 2.2 region of the EPR spectra of **14** (red), **16** (blue), **18** (green), and **20** (purple) to show the complex hyperfine splitting patterns associated with the spectra, and (C) Second derivative plots of the EPR spectra of **14** (red), **16** (blue), **18** (green), and **20** (purple), further illustrating the complexity of the hyperfine splitting patterns. Coupling to ^{59}Co (I = 7/2) is apparent in all of the EPR spectra, but is accompanied by additional superhyperfine coupling, likely to one or more ^{31}P (I = ½) nuclei and potentially one or both ^{127}I nuclei (I = 5/2). Due to the poor resolution of the hyperfine coupling and the multiple atoms with nuclear spins present in these molecules, EPR spectra could not be confidently simulated using a single set of parameters that would allow conclusions to be drawn about the magnitude of coupling constants to the ^{59}Co or central ^{31}P nuclei.

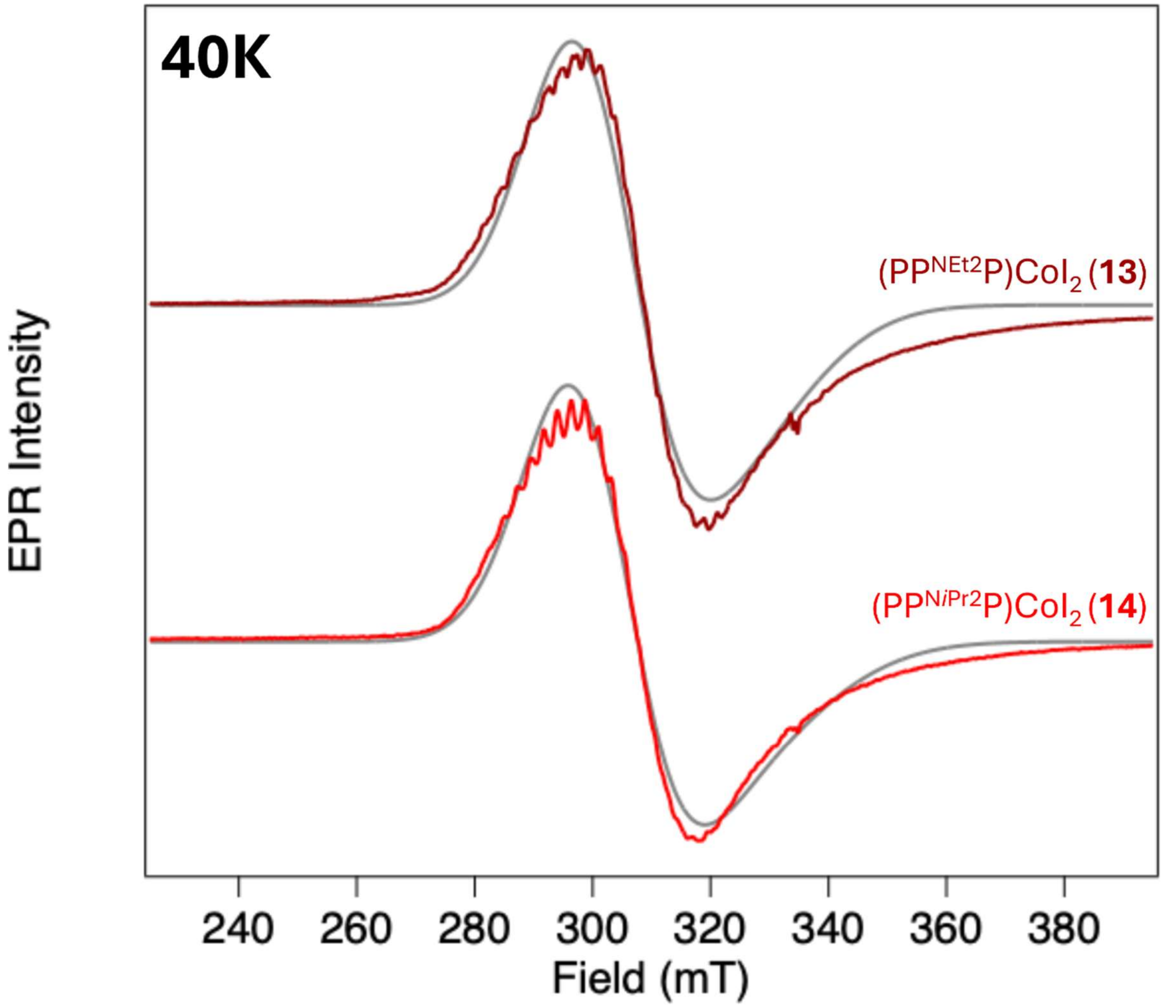

Figure S113. Experimental (red) and simulated (gray) X-band EPR spectra of **13** (top) and **14** (bottom) collected in frozen THF at 40 K. Simulations were performed with $g_x \neq g_x \neq g_x$, without including any hyperfine coupling parameters and the resulting *g* values are listed in Table S1. Simulations included inhomogenous line broadening due to unresolved hyperfine coupling (H-Strain).

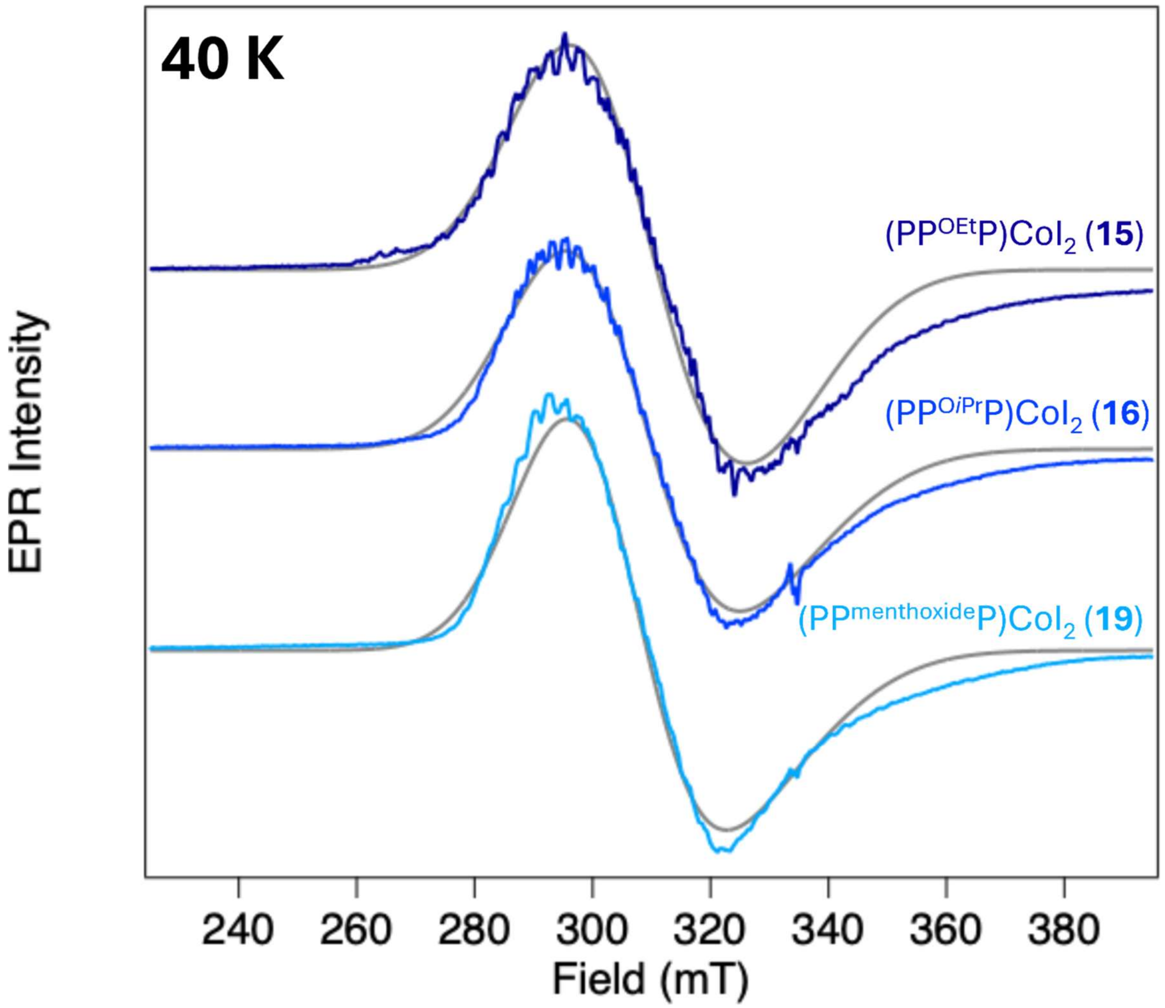

Figure S114. Experimental (blue) and simulated (gray) X-band EPR spectra of **15** (top), **16** (middle), and **19** (bottom) collected in frozen THF at 40 K. Simulations were performed with $g_x \neq g_x \neq g_x$, without including any hyperfine coupling parameters and the resulting *g* values are listed in Table S1. Simulations included inhomogenous line broadening due to unresolved hyperfine coupling (H-Strain).

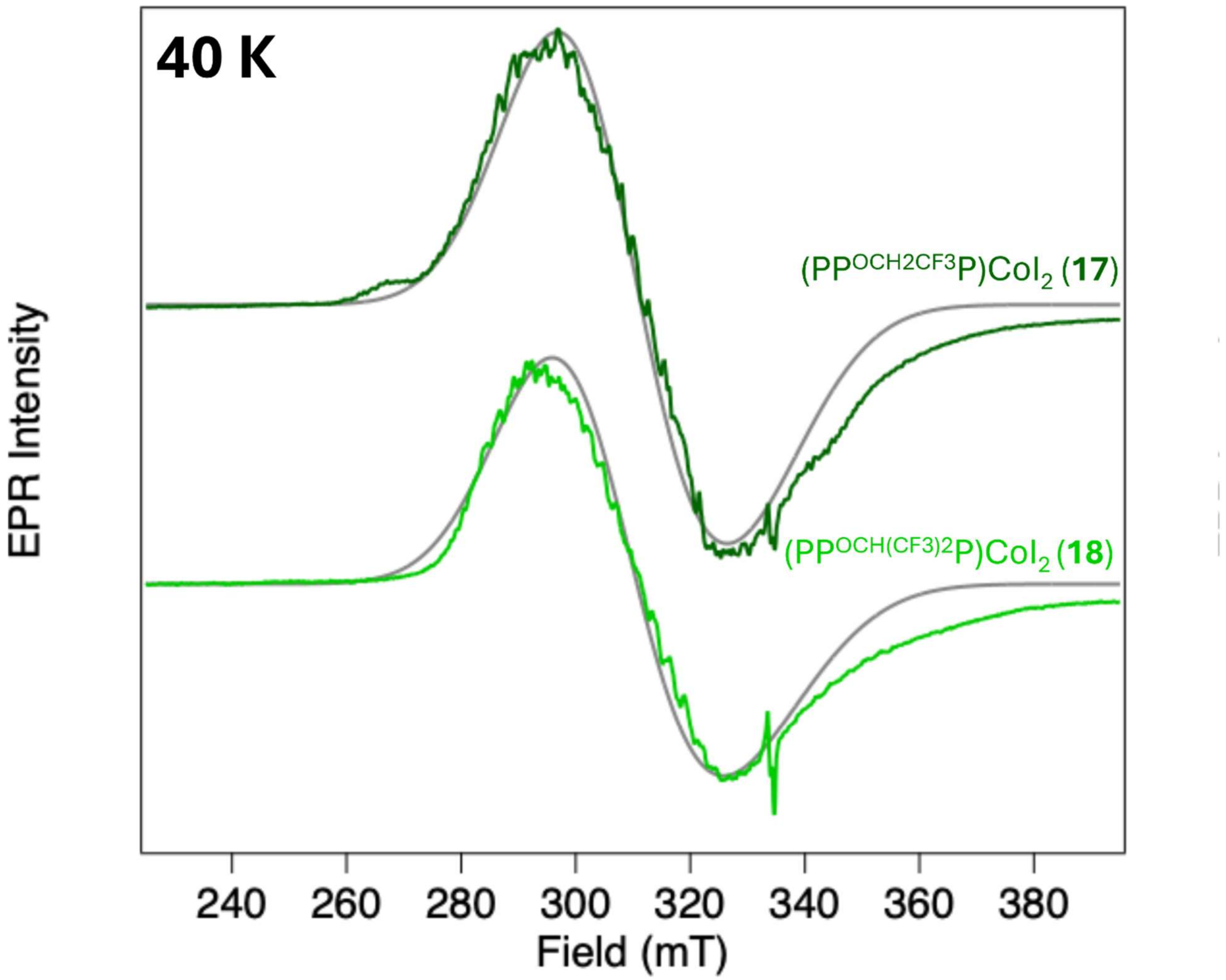

Figure S115. Experimental (green) and simulated (gray) X-band EPR spectra of **17** (top) and **18** (bottom) collected in frozen THF at 40 K. Simulations were performed with $g_x \neq g_x \neq g_x$, without including any hyperfine coupling parameters and the resulting g values are listed in Table S1. Simulations included inhomogenous line broadening due to unresolved hyperfine coupling (H-Strain).

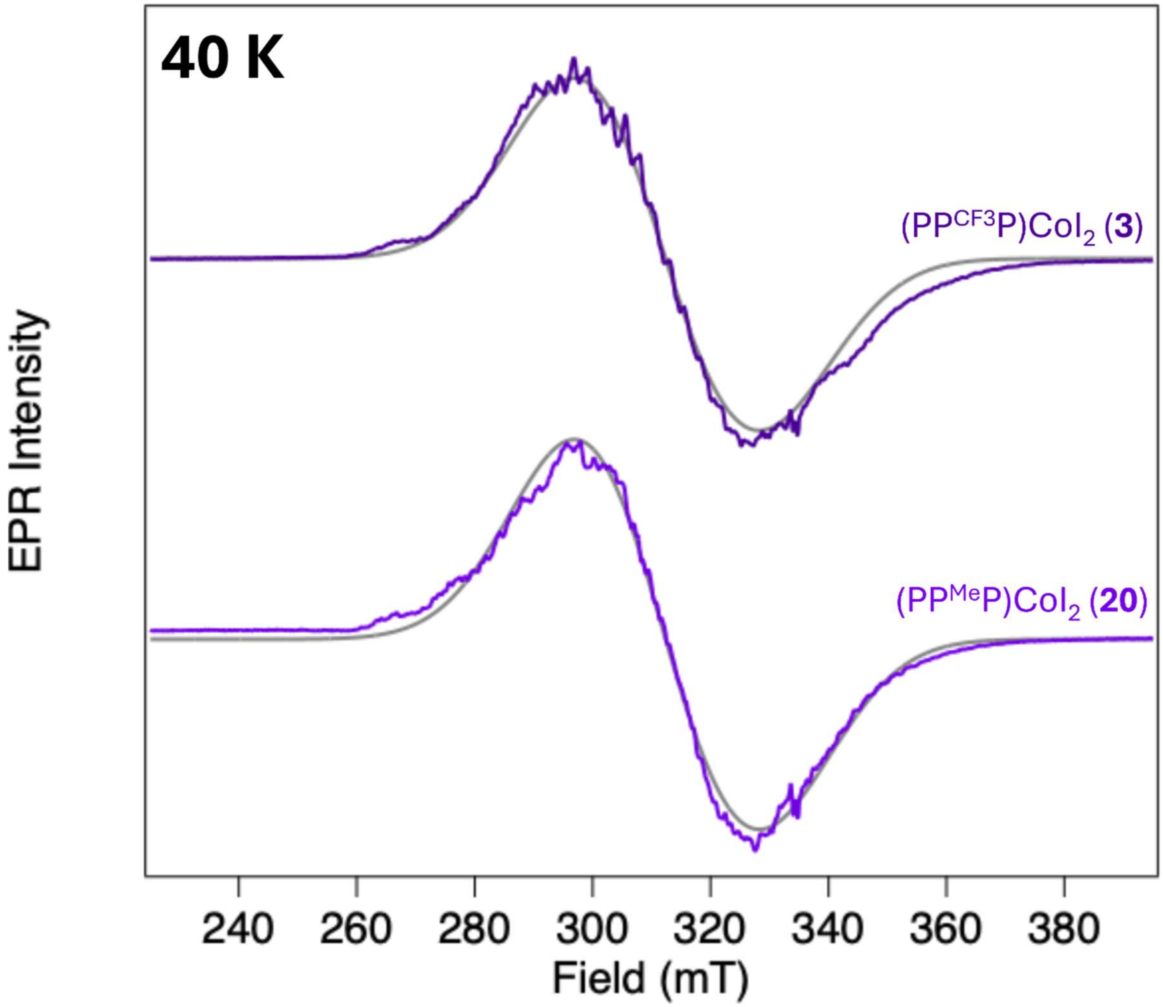

Figure S116. Experimental (purple) and simulated (gray) X-band EPR spectra of **3** (top) and **20** (bottom) collected in frozen THF at 40 K. Simulations were performed with $g_x \neq g_x \neq g_x$, without including any hyperfine coupling parameters and the resulting *g* values are listed in Table S1. Simulations included inhomogenous line broadening due to unresolved hyperfine coupling (H-Strain).

Table S2: g_{iso} and A_{iso} (^{59}Co) values for compounds **3**, **13**, **15-18**, and **20** obtained from simulation of room temperature (298 K) EPR spectra collected in THF. Although there are subtle differences in the A_{iso} values, they do not appear to correlate with any other trends related to Co-P distance or electron density at the Co center.

**Complex**	g_{iso}	**A_{iso} (Co) (MHz)**
($PP^{NEt2}P$)CoI_2 (**13**)	2.15	94.8
($PP^{OEt}P$)CoI_2 (**15**)	2.14	96.6
($PP^{O\textit{i}Pr}P$)CoI_2 (**16**)	2.14	93.0
($PP^{OCH2CF3}P$)CoI_2 (**17**)	2.13	92.0
($PP^{OCH(CF3)2}P$)CoI_2 (**18**)	2.13	93.1
($PP^{Me}P$)CoI_2 (**20**)	2.14	89.3

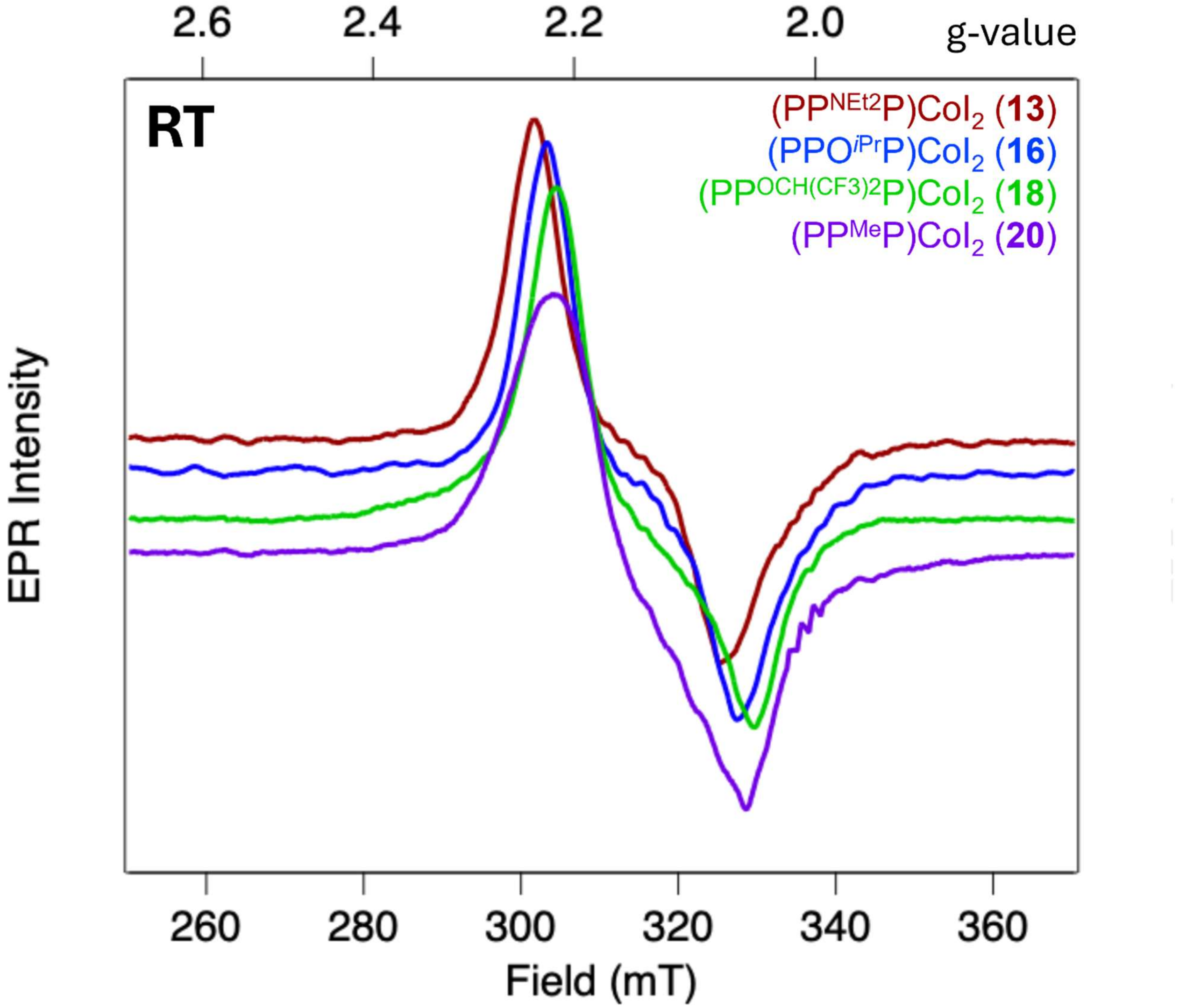

Figure S117. (A) X-band EPR spectra of **13** (red), **16** (blue), **18** (green), and **20** (purple) collected in THF solution at 298 K.

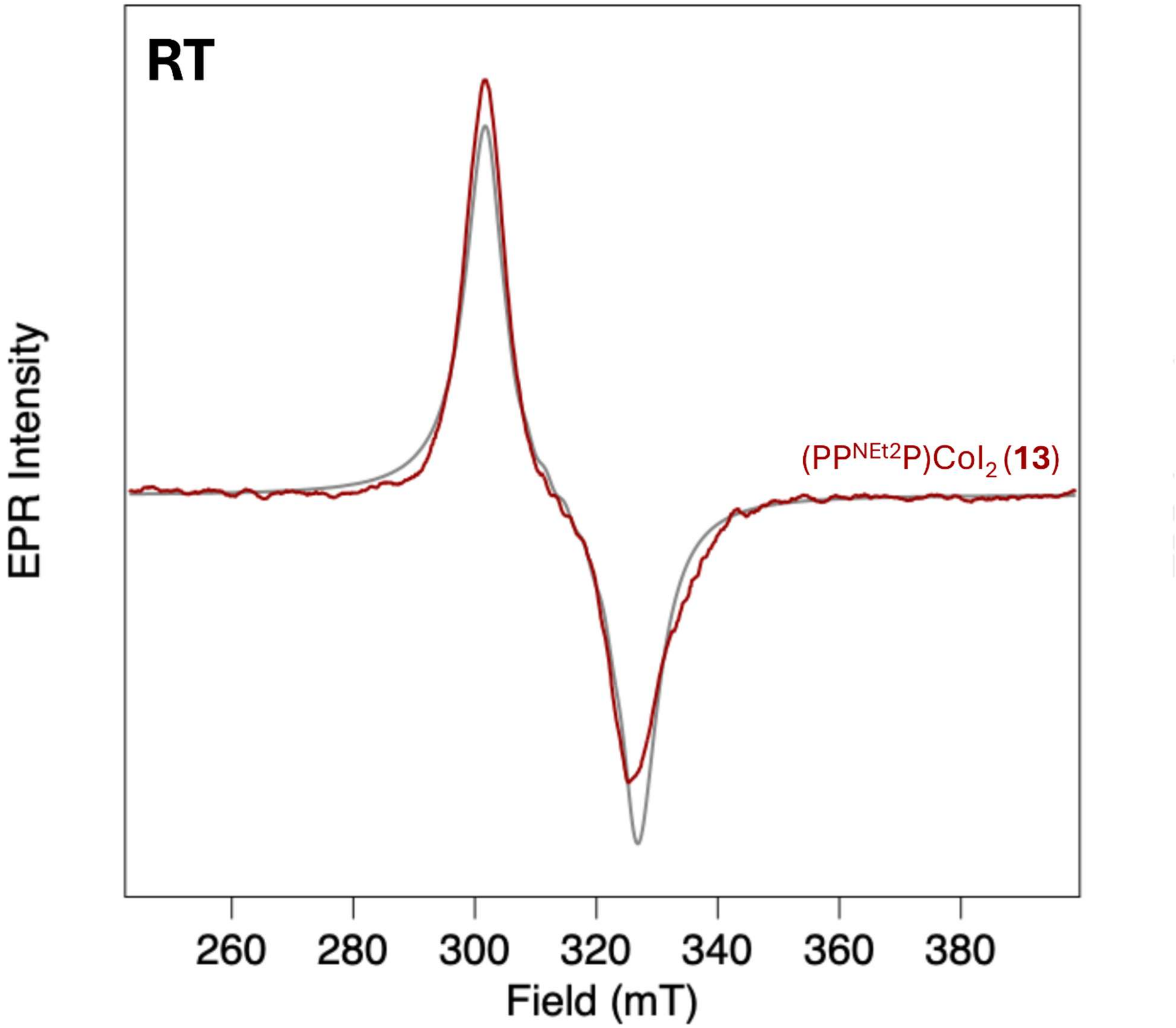

Figure S118. Experimental (red) and simulated (gray) X-band EPR spectra of **13** collected in THF solution at 298 K. Simulations were performed with $g_1 = g_2 = g_3 = g_{iso}$, and $A_1 = A_2 = A_3 = A_{iso}$ for hyperfine coupling to ^{59}Co (I = 7/2) and the resulting g_{iso} and A_{iso} values are listed in Table S2.

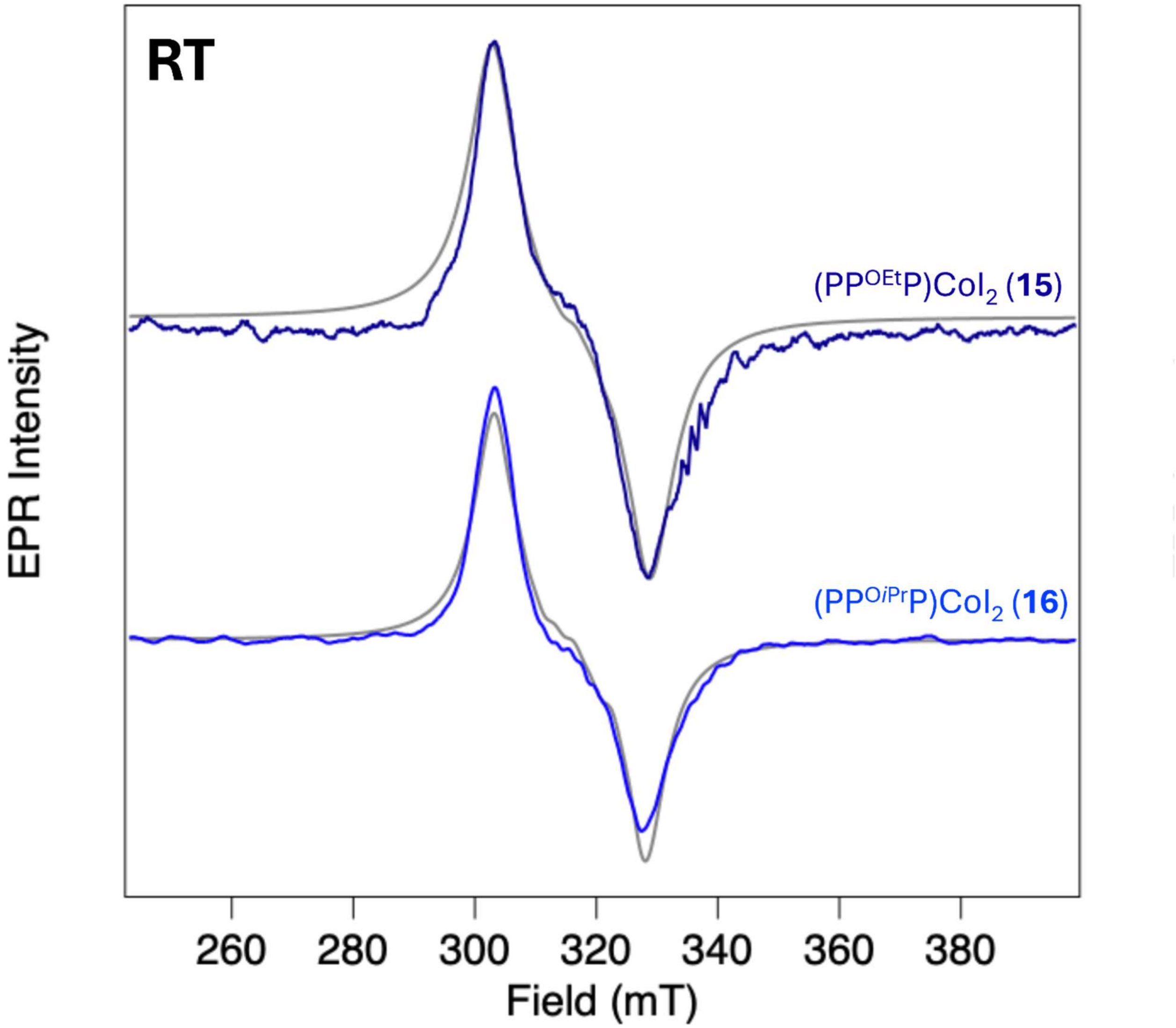

Figure S119. Experimental (blue) and simulated (gray) X-band EPR spectra of **15** (top) and **16** (bottom) collected in THF solution at 298 K. Simulations were performed with $g_1 = g_2 = g_3 = g_{iso}$, and $A_1 = A_2 = A_3 = A_{iso}$ hyperfine coupling to ^{59}Co ($I = 7/2$) and the resulting g_{iso} and A_{iso} values are listed in Table S2.

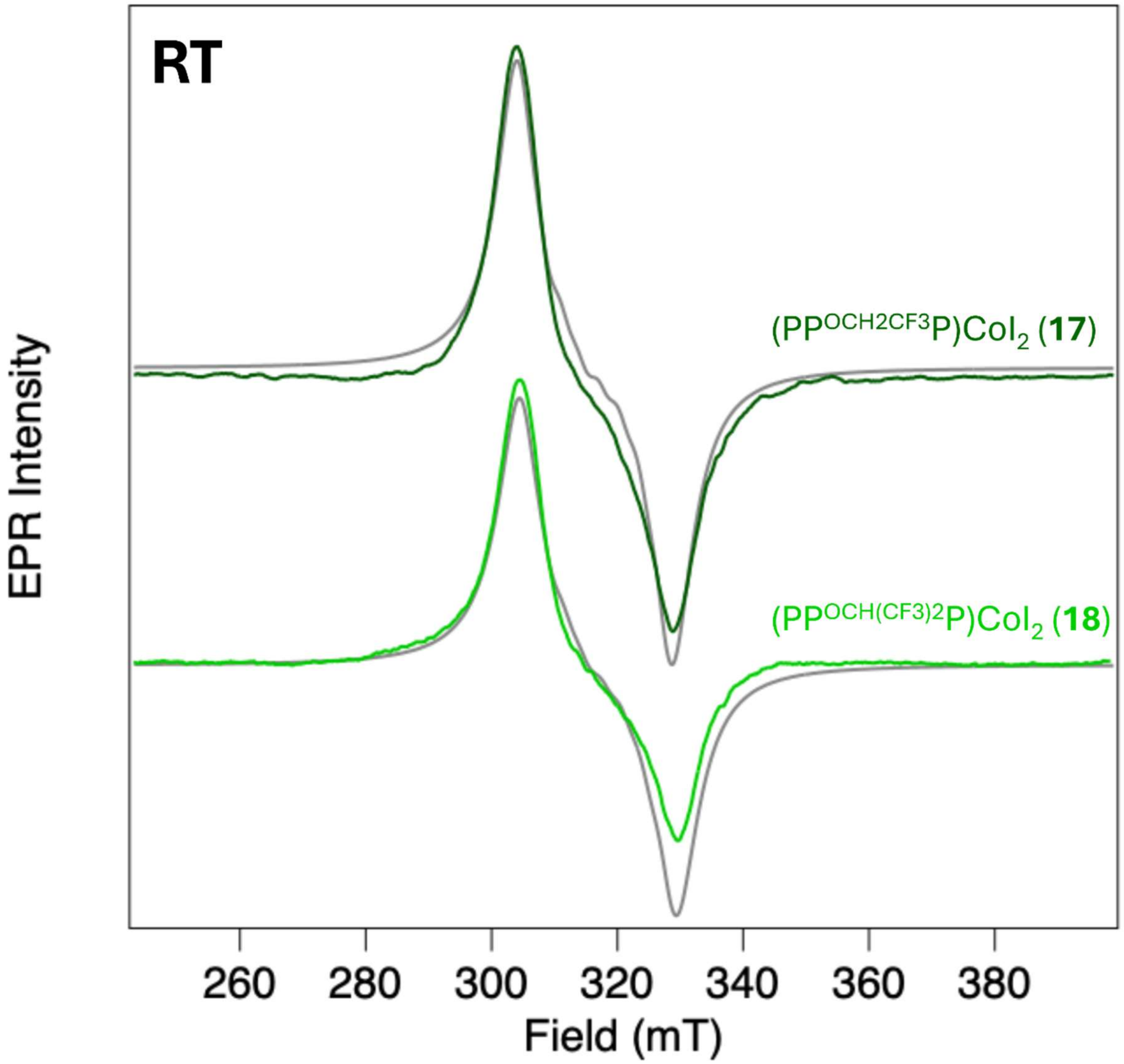

Figure S120. Experimental (green) and simulated (gray) X-band EPR spectra of **17** (top) and **18** (bottom) collected in THF solution at 298 K. Simulations were performed with $g_1 = g_2 = g_3 = g_{iso}$, and $A_1 = A_2 = A_3 = A_{iso}$ for hyperfine coupling to ^{59}Co (I = 7/2) and the resulting g_{iso} and A_{iso} values are listed in Table S2.

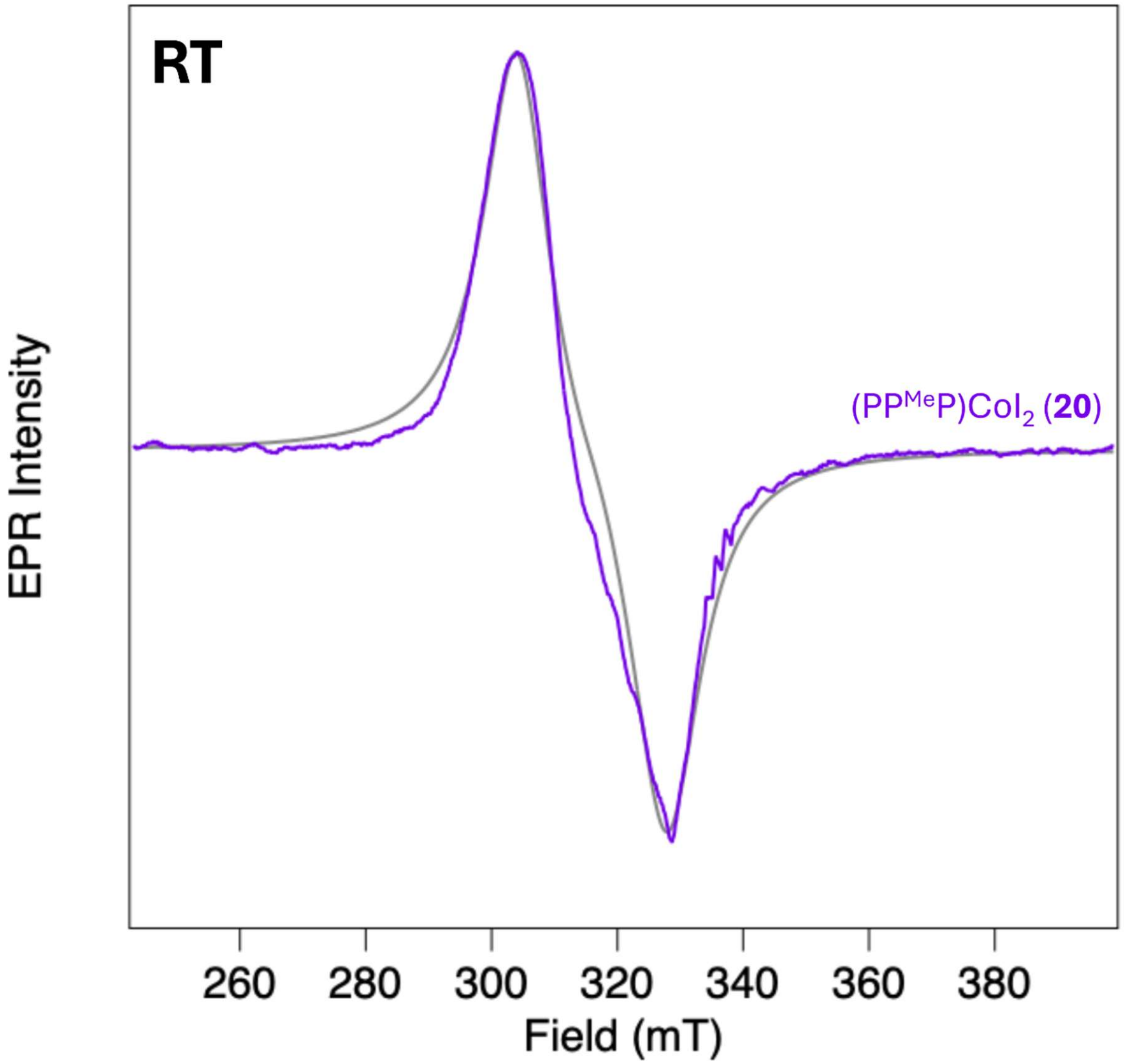

Figure S121. Experimental (purple) and simulated (gray) X-band EPR spectra of **20** (top) collected in THF solution at 298 K. Simulations were performed with $g_1 = g_2 = g_3 = g_{iso}$, and $A_1 = A_2 = A_3 = A_{iso}$ for hyperfine coupling to ^{59}Co (I = 7/2) and the resulting g_{iso} and A_{iso} values are listed in Table S2.

Kinetic Studies: Hydroboration of Styrene

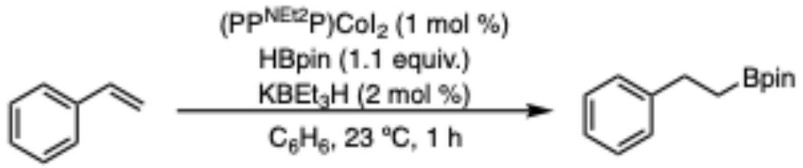

*Hydroboration of styrene with 1.0 mol % (PP^{NEt2}P)CoI$_2$ (**13**), trial one.*

A 20 mL scintillation vial was charged with **13** (5.8 mg, 5.8 mmol, 1.0 mol %) and a stir bar in an N_2 filled glovebox. To this was added C_6H_6 (235 mL), styrene (66.8 mL, 583.2 mmol, 1 equiv), HBpin (93.1 mL, 642 mmol, 1.1 equiv), and $KBEt_3H$ (116.64 mL, 100 mM in THF, 11.7 mmol, 2.0 mol %), resulting in an overall 11.4 mM solution with respect to catalyst. Upon addition of $KBEt_3H$, the solution was allowed to stir, and a timer was started. Aliquots were collected every 5 minutes for a total of 60 minutes.

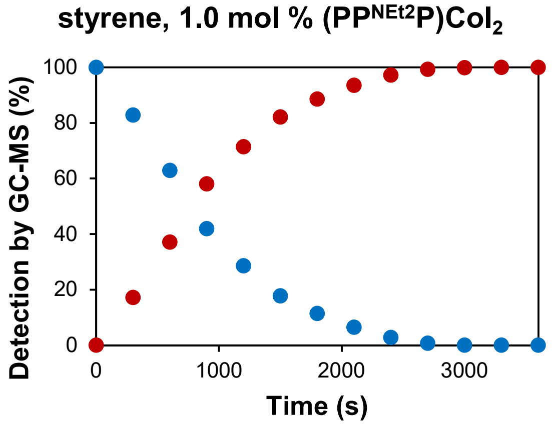

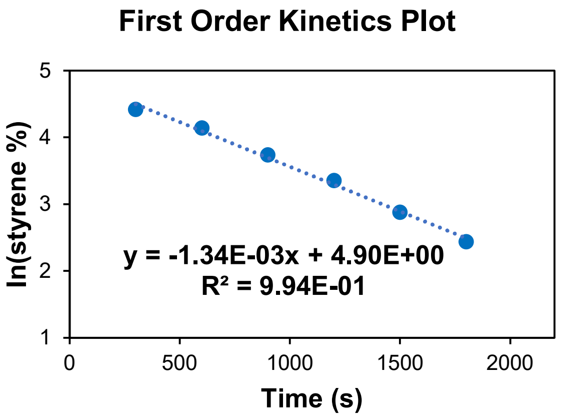

Figure S122: (left) Plotted timepoints of formation of linearly hydroborated styrene (red) and consumption of styrene (blue) versus time for 1.0 mol % loading of (PP^{NEt2}P)CoI$_2$ (**13**). (right) First order kinetics analysis of styrene %.

*Hydroboration of styrene with 1.0 mol % (PP^{NEt2}P)CoI$_2$ (**13**), trial two.*

A 20 mL scintillation vial was charged with **13** (5.2 mg, 5.2 mmol, 1.0 mol %) and a stir bar in an N_2 filled glovebox. To this was added C_6H_6 (214.8 mL), styrene (59.9 mL, 523 mmol, 1 equiv), HBpin (83.5 mL, 575 mmol, 1.1 equiv), and $KBEt_3H$ (104.6 mL, 100 mM in THF, 10.5 mmol, 2.0 mol %), resulting in an overall 11.3 mM solution with respect to catalyst. Upon addition of $KBEt_3H$, the solution was allowed to stir, and a timer was started. Aliquots were collected every 5 minutes for a total of 60 minutes.

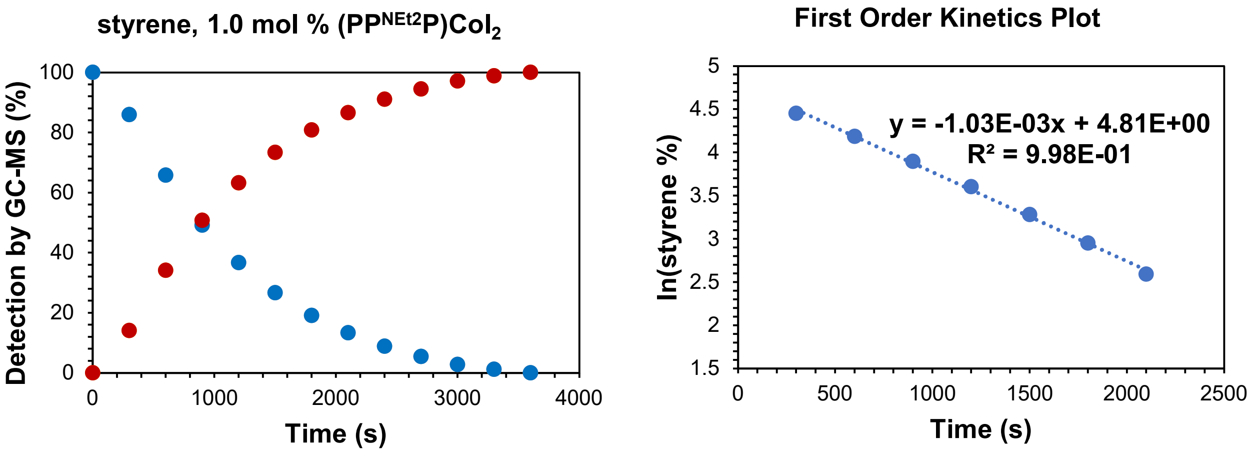

Figure 123: (left) Plotted timepoints of formation of linearly hydroborated styrene (red) and consumption of styrene (blue) versus time for 1.0 mol % loading of (PP^{NEt2}P)CoI$_2$ (**13**). (right) First order kinetics analysis of styrene %.

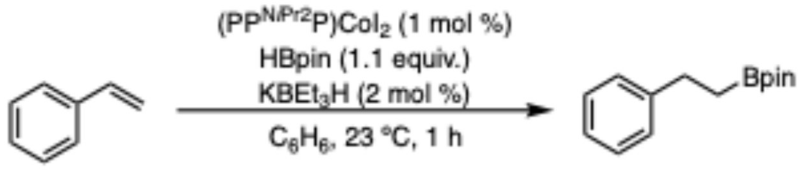

*Hydroboration of styrene with 1.0 mol % ($PP^{NiPr2}P$)CoI_2 (**14**), trial one.*

A 20 mL scintillation vial was charged with **14** (5.4 mg, 5.3 mmol, 1.0 mol %) and a stir bar in an N_2 filled glovebox. To this was added C_6H_6 (213 mL), styrene (60.5 mL, 528 mmol, 1 equiv), HBpin (84.3 mL, 581 mmol, 1.1 equiv), and $KBEt_3H$ (105.6 mL, 100 mM in THF, 10.6 mmol, 2.0 mol %), resulting in an overall 11.4 mM solution with respect to catalyst. Upon addition of $KBEt_3H$, the solution was allowed to stir, and a timer was started. Aliquots were collected every 5 minutes for a total of 60 minutes.

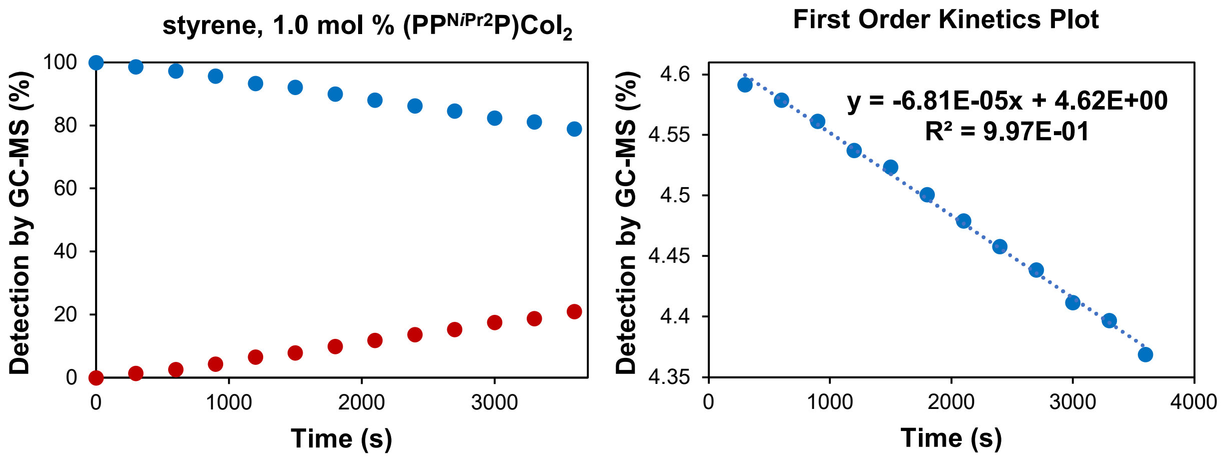

Figure S124: (left) Plotted timepoints of formation of linearly hydroborated styrene (red) and consumption of styrene (blue) versus time for 1.0 mol % loading of ($PP^{NiPr2}P$)CoI_2 (**14**). (right) First order kinetics analysis of styrene %.

*Hydroboration of styrene with 1.0 mol % ($PP^{NiPr2}P$)CoI_2 (**14**), trial two.*

A 20 mL scintillation vial was charged with **14** (5.8 mg, 5.7 mmol, 1.0 mol %) and a stir bar in an N_2 filled glovebox. To this was added C_6H_6 (233.0 mL), styrene (65.0 mL, 567 mmol, 1 equiv), HBpin (90.5 mL, 624 mmol, 1.1 equiv), and $KBEt_3H$ (113.4 mL, 100 mM in THF, 11.3 mmol, 2.0 mol %), resulting in an overall 11.3 mM solution with respect to catalyst. Upon addition of $KBEt_3H$, the solution was allowed to stir, and a timer was started. Aliquots were collected every 5 minutes for a total of 60 minutes.

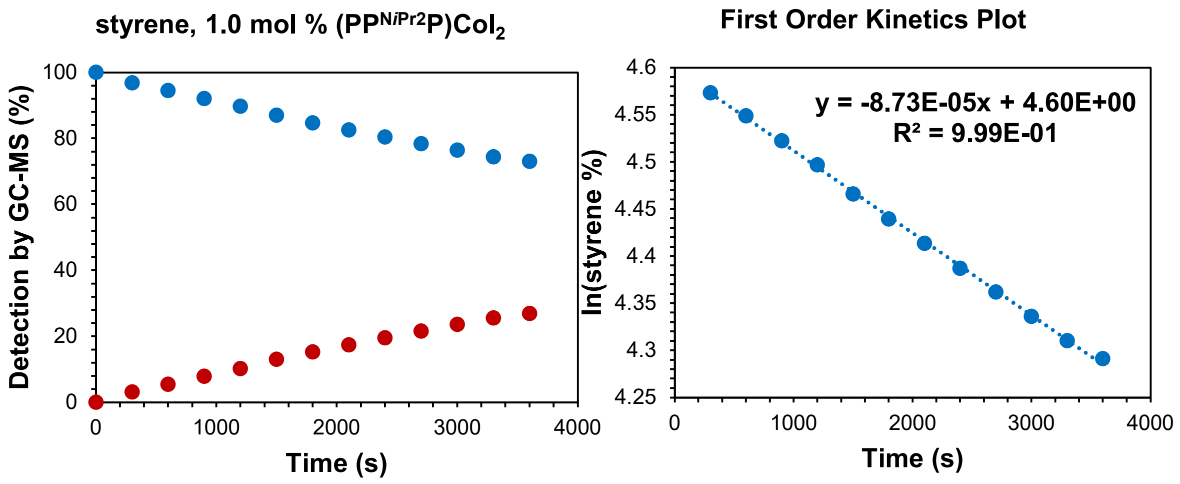

Figure S125: (left) Plotted timepoints of formation of linearly hydroborated styrene (red) and consumption of styrene (blue) versus time for 1.0 mol % loading of ($PP^{NiPr2}P$)CoI_2 (**14**). (right) First order kinetics analysis of styrene %.

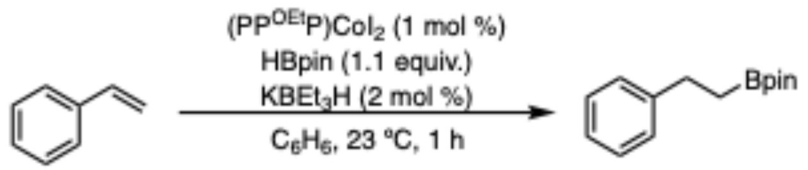

*Hydroboration of styrene with 1.0 mol % (PP^{OEt}P)CoI$_2$ (**15**), trial one.*

A 20 mL scintillation vial was charged with **15** (5.0 mg, 5.2 mmol, 1.0 mol %) and a stir bar in an N_2 filled glovebox. To this was added C_6H_6 (212.0 mL), styrene (59.2 mL, 517 mmol, 1 equiv), HBpin (82.5 mL, 568 mmol, 1.1 equiv), and $KBEt_3H$ (103.4 uL, 100 mM in THF, 10.3 mmol, 2.0 mol %), resulting in an overall 11.3 mM solution with respect to catalyst. Upon addition of $KBEt_3H$, the solution was allowed to stir, and a timer was started. Aliquots were collected every 5 minutes for a total of 50 minutes.

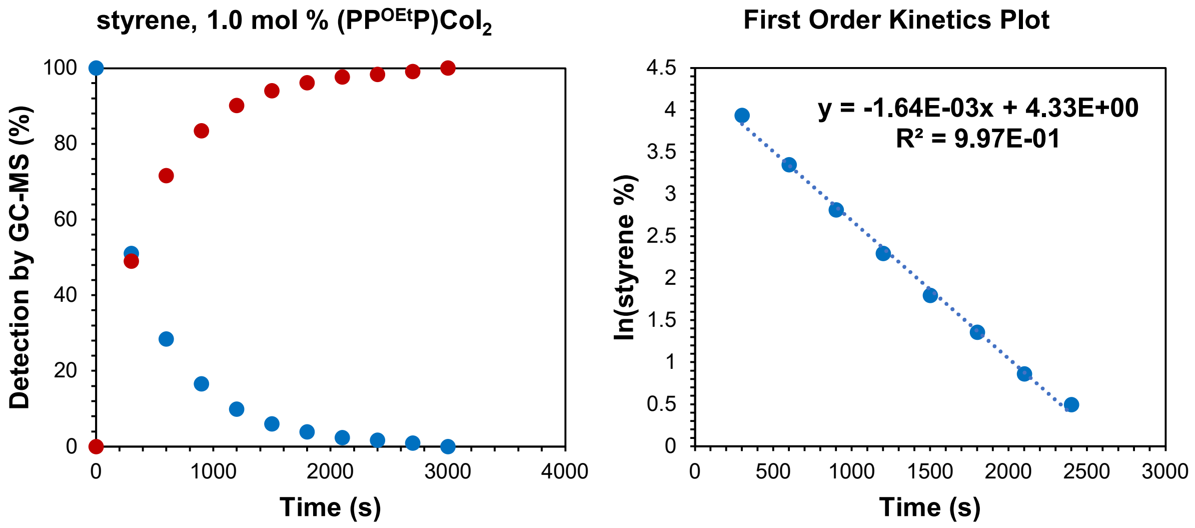

Figure 126: (left) Plotted timepoints of formation of linearly hydroborated styrene (red) and consumption of styrene (blue) versus time for 1.0 mol % loading of (PP^{OEt}P)CoI$_2$ (**15**). (right) First order kinetics analysis of styrene %.

*Hydroboration of styrene with 1.0 mol % ($PP^{OEt}P$)CoI_2 (**15**), trial two.*

A 20 mL scintillation vial was charged with **15** (3.9 mg, 4.0 mmol, 1.0 mol %) and a stir bar in an N_2 filled glovebox. To this was added C_6H_6 (165.6 mL), styrene (46.2 mL, 403 mmol, 1 equiv), HBpin (64.3 mL, 444 mmol, 1.1 equiv), and $KBEt_3H$ (80.6 mL, 100 mM in THF, 8.06 mmol, 2.0 mol %), resulting in an overall 11.3 mM solution with respect to catalyst. Upon addition of $KBEt_3H$, the solution was allowed to stir, and a timer was started. Aliquots were collected every 5 minutes for a total of 25 minutes.

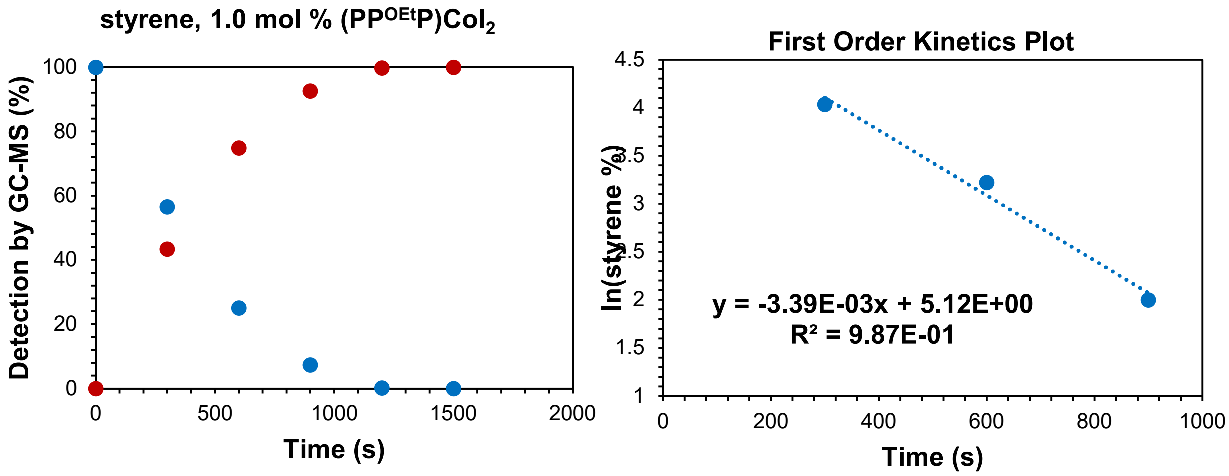

Figure S127: (left) Plotted timepoints of formation of linearly hydroborated styrene (red) and consumption of styrene (blue) versus time for 1.0 mol % loading of ($PP^{OEt}P$)CoI_2 (**15**). (right) First order kinetics analysis of styrene %.

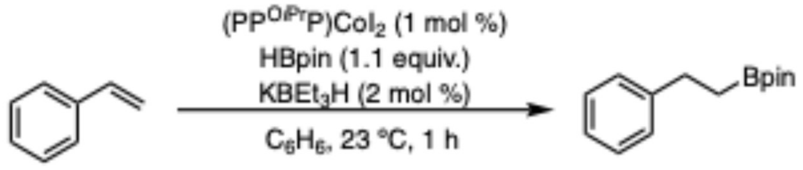

*Hydroboration of styrene with 1.0 mol % (PP^{OiPr}P)CoI$_2$ (**16**), trial one.*

A 20 mL scintillation vial was charged with **16** (4.7 mg, 4.8 mmol, 1.0 mol %) and a stir bar in an N_2 filled glovebox. To this was added C_6H_6 (196.7 mL), styrene (54.9 uL, 479 mmol, 1 equiv), HBpin (76.4 uL, 527 mmol, 1.1 equiv), and $KBEt_3H$ (95.8 mL, 100 mM in THF, 9.58 mmol, 2.0 mol %), resulting in an overall 11.3 mM solution with respect to catalyst. Upon addition of $KBEt_3H$, the solution was allowed to stir, and a timer was started. Aliquots were collected every 5 minutes for a total of 50 minutes.

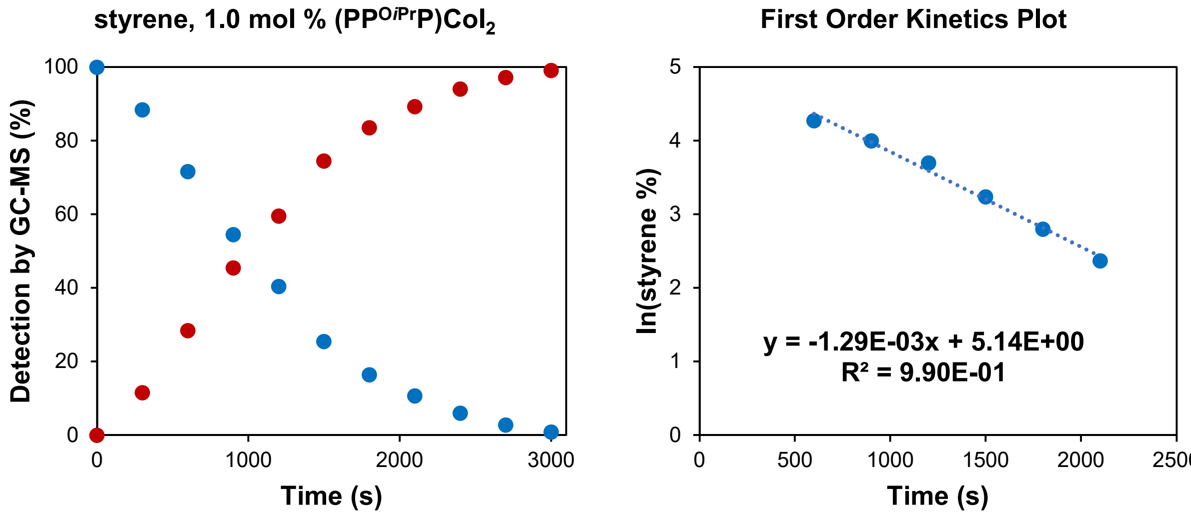

Figure S128: (left) Plotted timepoints of formation of linearly hydroborated styrene (red) and consumption of styrene (blue) versus time for 1.0 mol % loading of (PP^{OiPr}P)CoI$_2$ (**16**). (right) First order kinetics analysis of styrene %.

*Hydroboration of styrene with 1.0 mol % ($PP^{OiPr}P$)CoI_2 (**16**), trial two.*

A 20 mL scintillation vial was charged with **16** (4.9 mg, 5.0 mmol, 1.0 mol %) and a stir bar in an N_2 filled glovebox. To this was added C_6H_6 (205.1 mL), styrene (57.2 mL, 499 mmol, 1 equiv), HBpin (79.7 mL, 549 mmol, 1.1 equiv), and $KBEt_3H$ (99.9 mL, 100 mM in THF, 9.99 mmol, 2.0 mol %), resulting in an overall 11.3 mM solution with respect to catalyst. Upon addition of $KBEt_3H$, the solution was allowed to stir, and a timer was started. Aliquots were collected every 5 minutes for a total of 55 minutes.

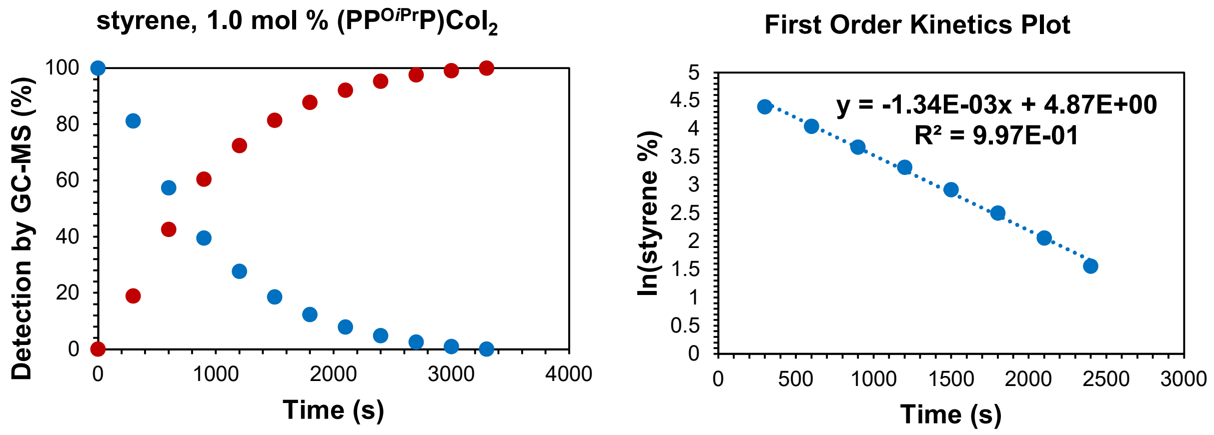

Figure S129: (left) Plotted timepoints of formation of linearly hydroborated styrene (red) and consumption of styrene (blue) versus time for 1.0 mol % loading of ($PP^{OiPr}P$)CoI_2 (**16**). (right) First order kinetics analysis of styrene %.

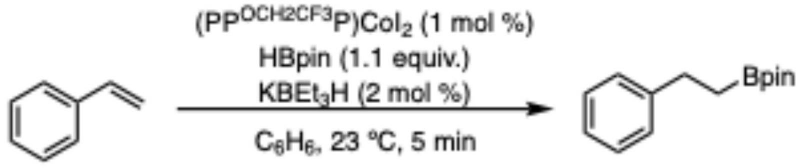

*Hydroboration of styrene with 1.0 mol % (PP^{OCH2CF3}P)CoI$_2$ (**17**), trial one.*

A 20 mL scintillation vial was charged with **17** (5.7 mg, 5.6 mmol, 1.0 mol %) and a stir bar in an N_2 filled glovebox. To this was added C_6H_6 (229.2 mL), styrene (63.9 mL, 558 mmol, 1 equiv), HBpin (89.1 mL, 614 mmol, 1.1 equiv), and KBEt$_3$H (111.6 mL, 100 mM in THF, 11.2 mmol, 2.0 mol %), resulting in an overall 11.3 mM solution with respect to catalyst. Upon addition of KBEt$_3$H, the solution was allowed to stir, and a timer was started. Aliquots were collected every 5 minutes for a total of 60 minutes. GC-FID of the 5-minute aliquot showed full conversion of styrene to hydroborated styrene.

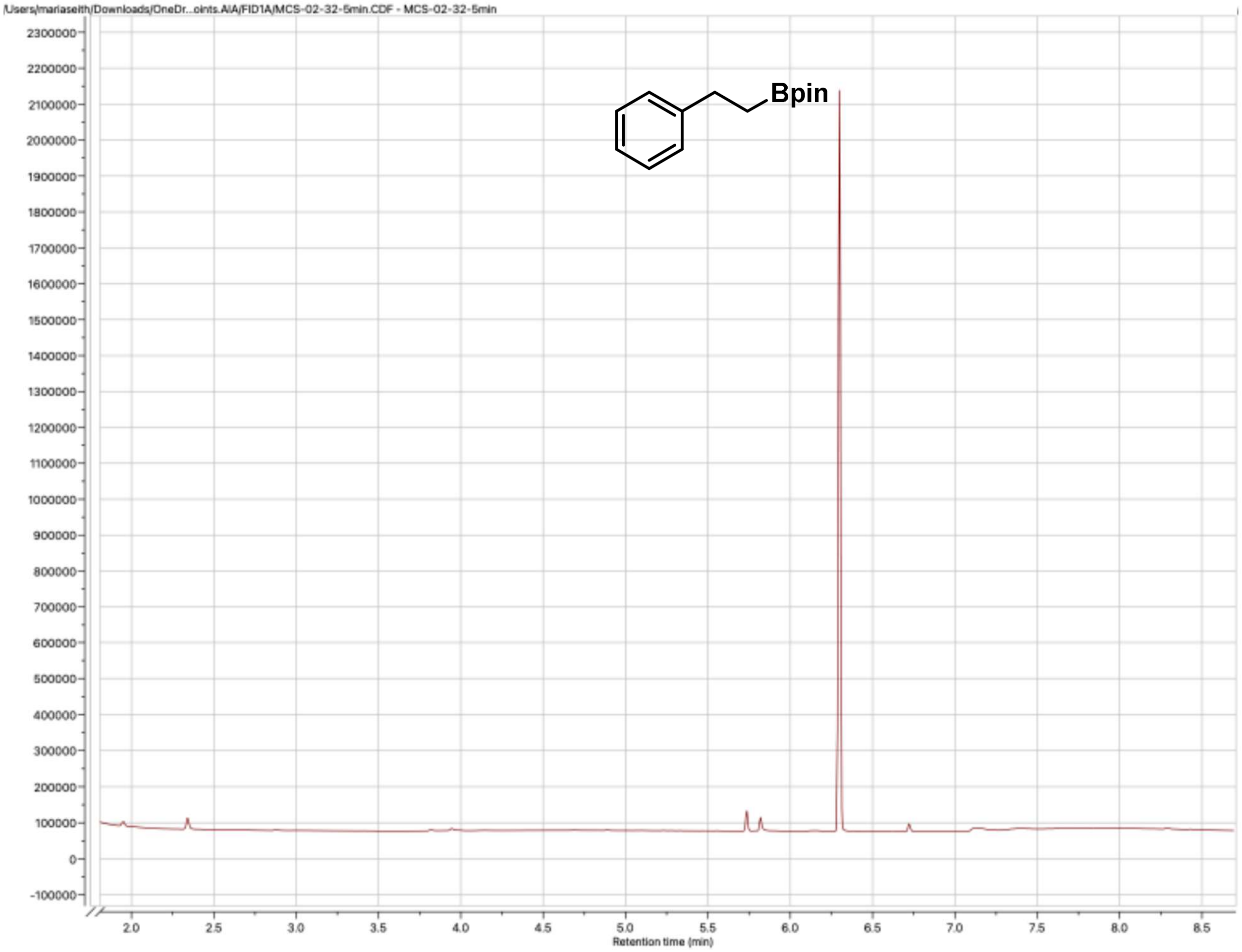

Figure S130. GC-FID of reaction mixture of catalysis carried out under the conditions above with 1.0 mol % **17** after 5 minutes.

*Hydroboration of styrene with 1.0 mol % ($PP^{OCH2CF3}P$)CoI_2 (**17**), trial two.*

A 20 mL scintillation vial was charged with **17** (5.7 mg, 5.6 mmol, 1.0 mol %) and a stir bar in an N_2 filled glovebox. To this was added C_6H_6 (229.3 mL), styrene (63.9 mL, 558 mmol, 1 equiv), HBpin (89.1 mL, 614 mmol, 1.1 equiv), and $KBEt_3H$ (111.6 mL, 100 mM in THF, 11.2 mmol, 2.0 mol %), resulting in an overall 11.3 mM solution with respect to catalyst. Upon addition of $KBEt_3H$, the solution was allowed to stir, and a timer was started. Aliquots were collected every 5 minutes for a total of 60 minutes. GC-FID of the 5-minute aliquot showed full conversion of styrene to hydroborated styrene.

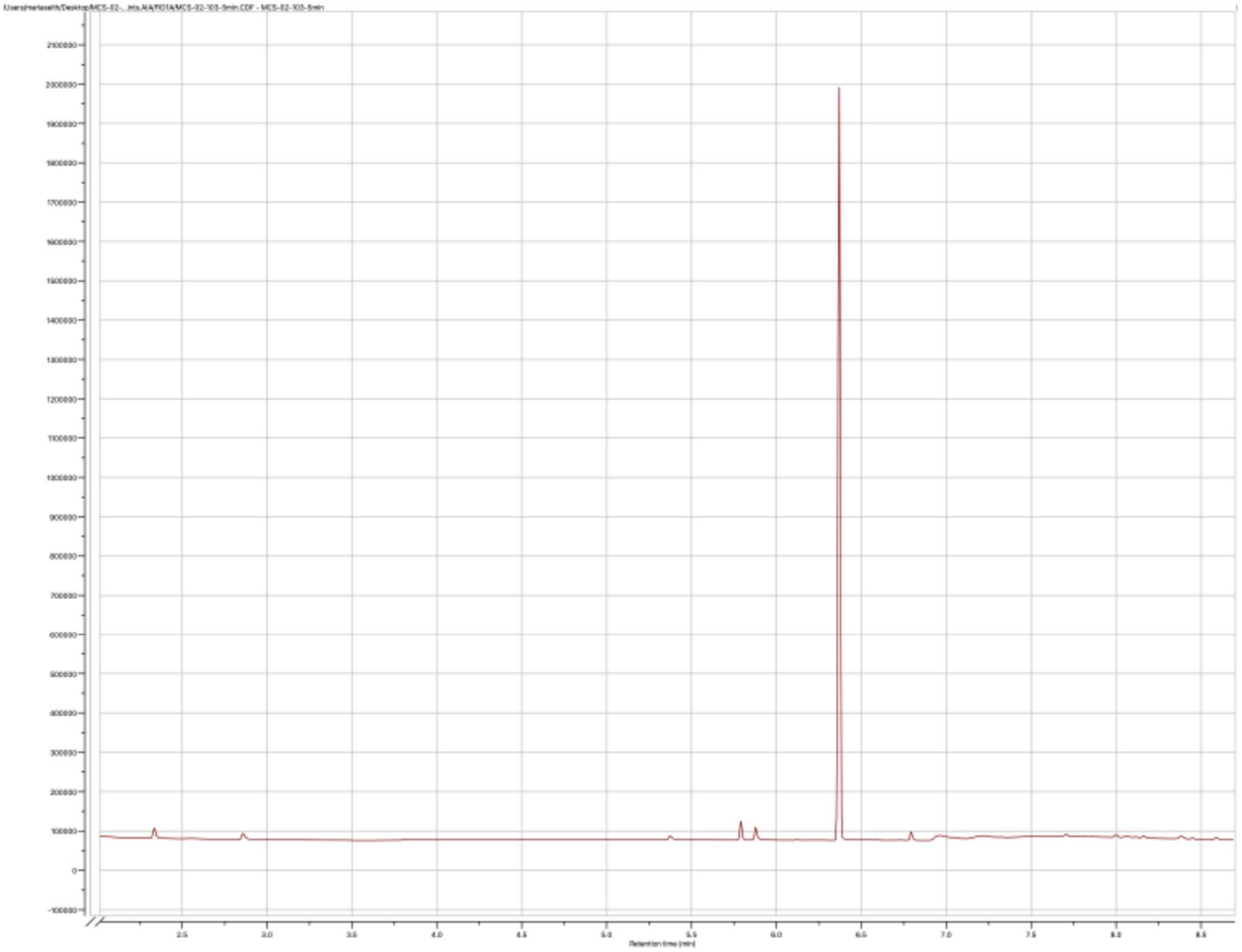

Figure S131. GC-FID of reaction mixture of catalysis carried out under the conditions above with 1.0 mol % **17** after 5 minutes.

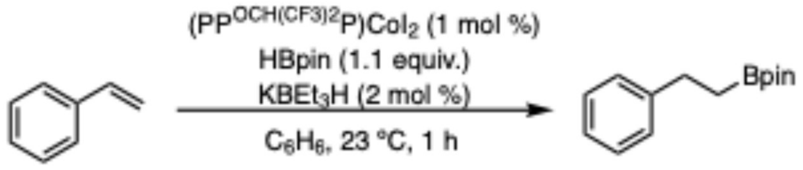

*Hydroboration of styrene with 1.0 mol % (PP$^{OCH(CF3)2}$P)CoI$_2$ (**18**), trial one.*

A 20 mL scintillation vial was charged with **18** (6.3 mg, 5.8 mmol, 1.0 mol%) and a stir bar in an N_2 filled glovebox. To this was added C_6H_6 (237.6 mL), styrene (66.3 mL, 578 mmol, 1 equiv), HBpin (92.3 mL, 636 mmol, 1.1 equiv), and $KBEt_3H$ (115.7 mL, 100 mM in THF, 11.6 mmol, 2.0 mol %), resulting in an overall 11.3 mM solution with respect to catalyst. Upon addition of $KBEt_3H$, the solution was allowed to stir, and a timer was started. Aliquots were collected every 5 minutes for a total of 30 minutes.

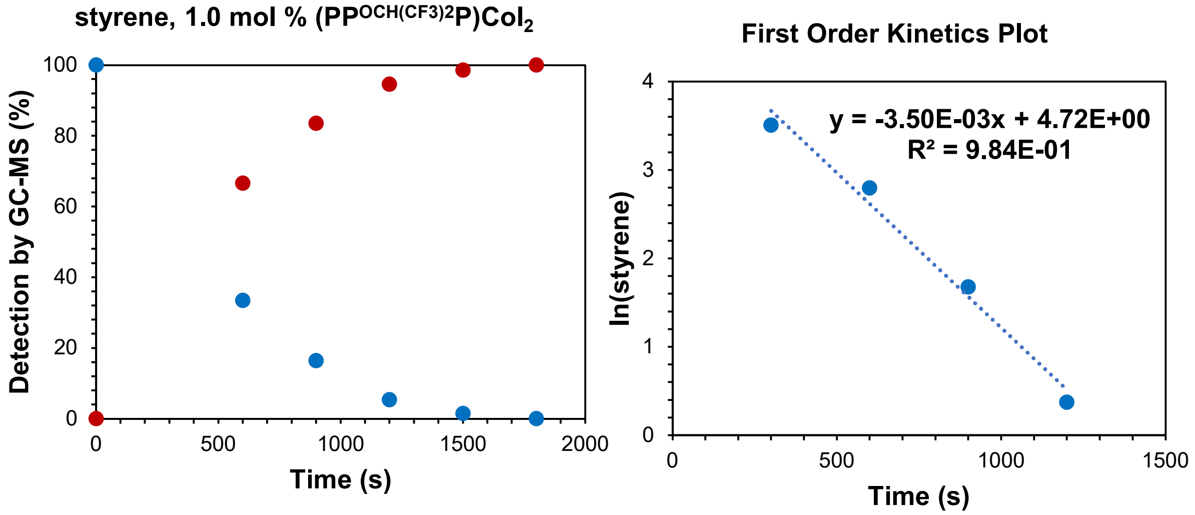

Figure S132: (left) Plotted timepoints of formation of linearly hydroborated styrene (red) and consumption of styrene (blue) versus time for 1.0 mol % loading of (PP$^{OCH(CF3)2}$P)CoI$_2$ (**18**). (right) First order kinetics analysis of styrene %.

*Hydroboration of styrene with 1.0 mol % ($PP^{OCH(CF3)2}P$)CoI_2 (**18**), trial two.*

A 20 mL scintillation vial was charged with **18** (4.7 mg, 4.3 mmol, 1.0 mol %) and a stir bar in an N_2 filled glovebox. To this was added C_6H_6 (177.2 mL), styrene (49.4 mL, 431 mmol, 1 equiv), HBpin (68.9 mL, 474 mmol, 1.1 equiv), and $KBEt_3H$ (86.3 mL, 100 mM in THF, 8.63 mmol, 2.0 mol %), resulting in an overall 11.3 mM solution with respect to catalyst. Upon addition of $KBEt_3H$, the solution was allowed to stir, and a timer was started. Aliquots were collected every 5 minutes for a total of 20 minutes.

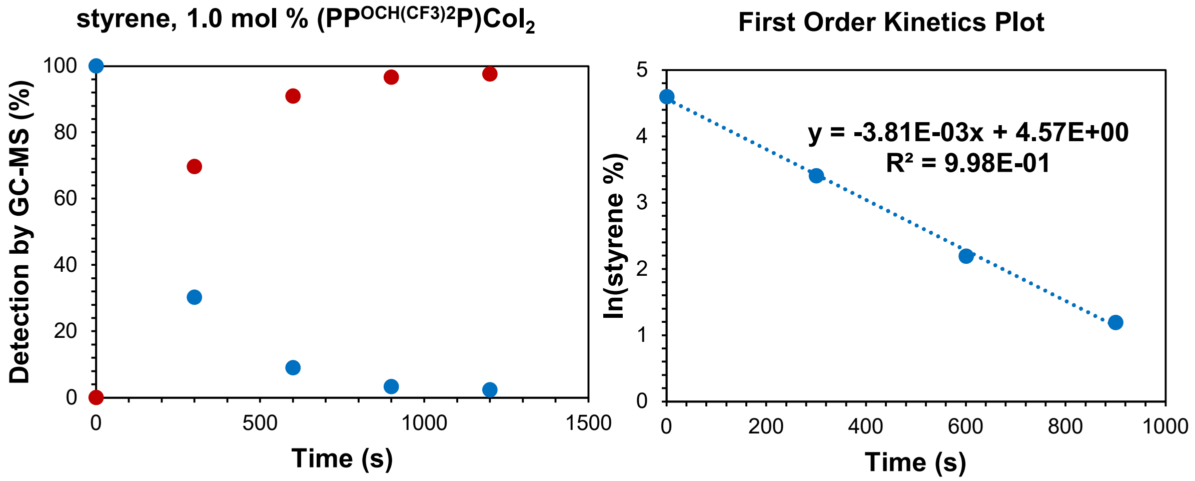

Figure S133: (left) Plotted timepoints of formation of linearly hydroborated styrene (red) and consumption of styrene (blue) versus time for 1.0 mol % loading of ($PP^{OCH(CF3)2}P$)CoI_2 (**18**). (right) First order kinetics analysis of styrene %.

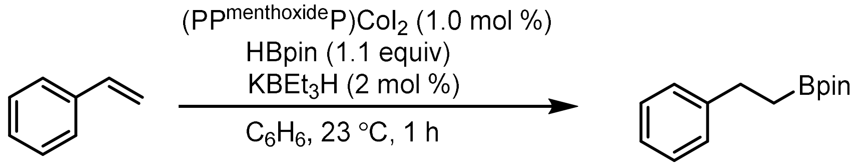

*Hydroboration of styrene with 1.0 mol % ($P^{menthoxide}P$)CoI_2 (**19**), trial one.*

A 20 mL scintillation vial was charged with **19** (4.1 mg, 3.8 mmol, 1.0 mol%) and a stir bar in an N_2 filled glovebox. To this was added C_6H_6 (156.3 mL), styrene (43.6 mL, 380 mmol, 1 equiv), HBpin (60.7 mL, 418 mmol, 1.1 equiv), and $KBEt_3H$ (76.1 mL, 100 mM in THF, 7.61 mmol, 2.0 mol %), resulting in an overall 11.3 mM solution with respect to catalyst. Upon addition of $KBEt_3H$, the solution was allowed to stir, and a timer was started. Aliquots were collected every 5 minutes for a total of 60 minutes.

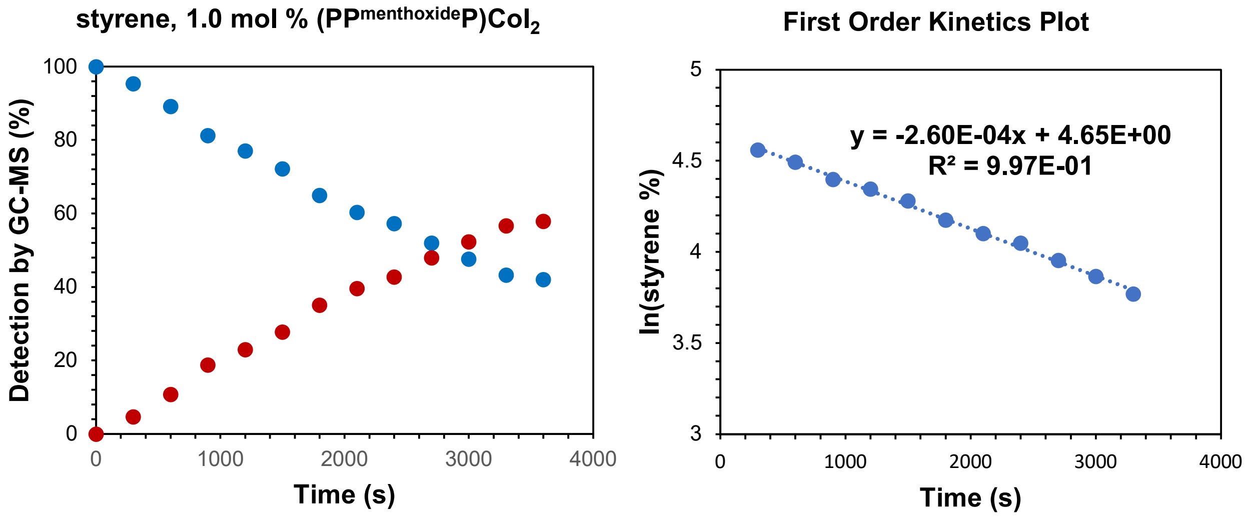

Figure S134: (left) Plotted timepoints of formation of linearly hydroborated styrene (red) and consumption of styrene (blue) versus time for 1.0 mol % loading of ($PP^{menthoxide}P$)CoI_2 (**19**). (right) First order kinetics analysis of styrene %.

*Hydroboration of styrene with 1.0 mol % (PP^{menthoxide}P)CoI_2 (**19**), trial two.*

A 20 mL scintillation vial was charged with **19** (10.8 mg, 10.0 mmol, 1.0 mol %) and a stir bar in an N_2 filled glovebox. To this was added C_6H_6 (411.7 mL), styrene (114.8 mL, 1.00 mmol, 1 equiv), HBpin (160.0 mL, 1.10 mmol, 1.1 equiv), and $KBEt_3H$ (200.4 mL, 100 mM in THF, 20.0 mmol, 2.0 molv%), resulting in an overall 11.3 mM solution with respect to catalyst. Upon addition of $KBEt_3H$, the solution was allowed to stir, and a timer was started. Aliquots were collected every 5 minutes for a total of 60 minutes.

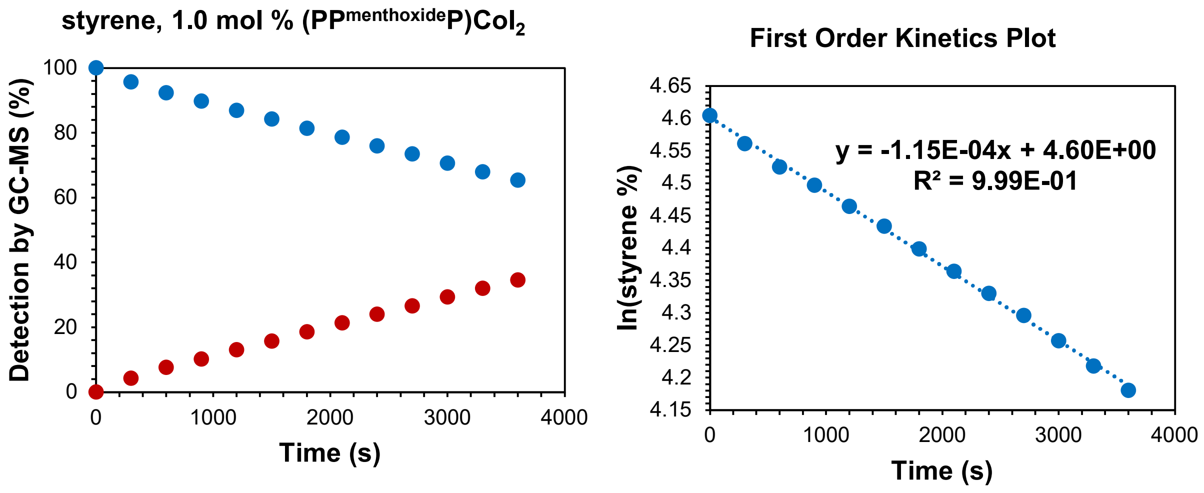

Figure S135: (left) Plotted timepoints of formation of linearly hydroborated styrene (red) and consumption of styrene (blue) versus time for 1.0 mol % loading of (PP^{menthoxide}P)CoI_2 (**19**). (right) First order kinetics analysis of styrene %.

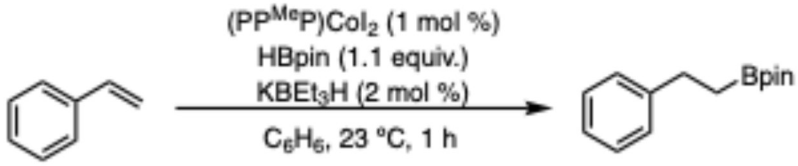

*Hydroboration of styrene with 1.0 mol % (PP^{Me}P)CoI$_2$ (**20**), trial one.*

A 20 mL scintillation vial was charged with **20** (7.6 mg, 8.1 mmol, 1.0 mol %) and a stir bar in an N_2 filled glovebox. To this was added C_6H_6 (333.0 mL), styrene (92.9 mL, 811 mmol, 1 equiv), HBpin (129.4 mL, 892 mmol, 1.1 equiv), and $KBEt_3H$ (162.2 mL, 100 mM in THF, 16.2 mmol, 2.0 mol%), resulting in an overall 11.3 mM solution with respect to catalyst. Upon addition of $KBEt_3H$, the solution was allowed to stir, and a timer was started. Aliquots were collected every 5 minutes for a total of 30 minutes.

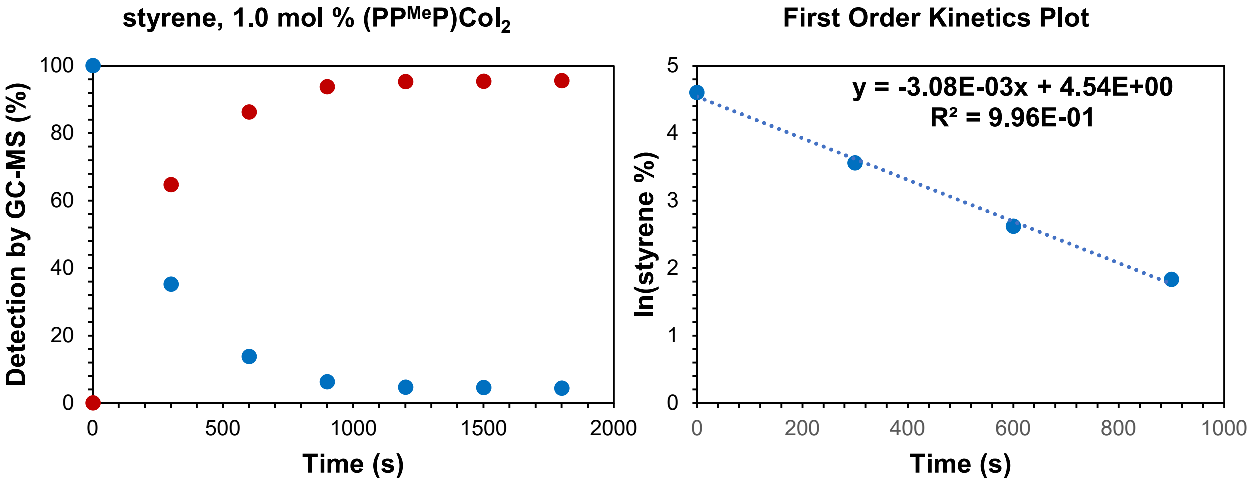

Figure S136: (left) Plotted timepoints of formation of linearly hydroborated styrene (red) and consumption of styrene (blue) versus time for 1.0 mol % loading of (PP^{Me}P)CoI$_2$ (**20**). (right) First order kinetics analysis of styrene %.

*Hydroboration of styrene with 1.0 mol % ($PP^{Me}P$)CoI_2 (**20**), trial two.*

A 20 mL scintillation vial was charged with **20** (10.6 mg, 11.3 mmol, 1.0 mol %) and a stir bar in an N_2 filled glovebox. To this was added C_6H_6 (464.5 mL), styrene (129.6 mL, 1.13 mmol, 1 equiv), HBpin (180.5 mL, 1.24 mmol, 1.1 equiv), and $KBEt_3H$ (226.2 mL, 100 mM in THF, 22.6 mmol, 2.0 mol %), resulting in an overall 11.3 mM solution with respect to catalyst. Upon addition of $KBEt_3H$, the solution was allowed to stir, and a timer was started. Aliquots were collected every 5 minutes for a total of 35 minutes.

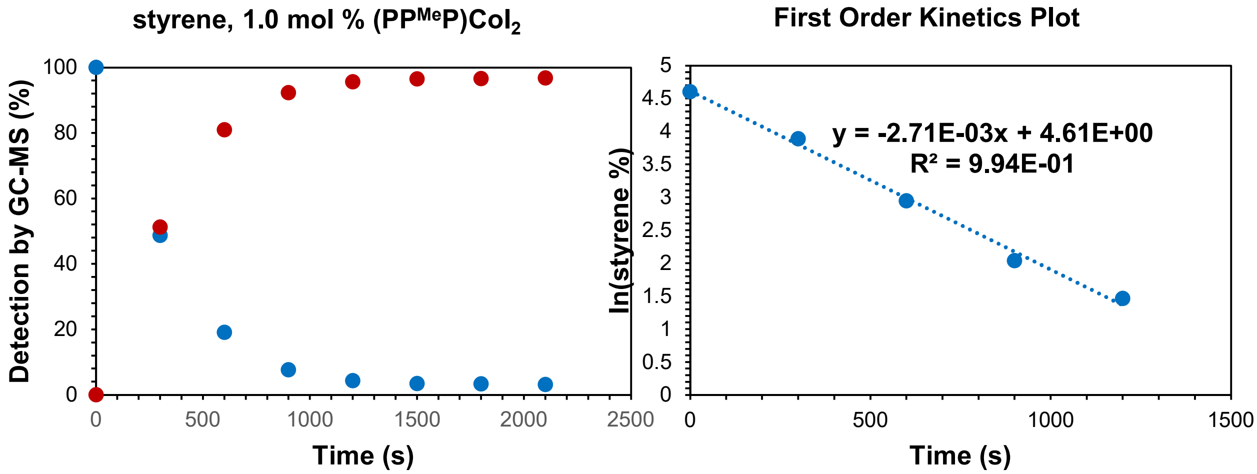

Figure S137: (left) Plotted timepoints of formation of linearly hydroborated styrene (red) and consumption of styrene (blue) versus time for 1.0 mol % loading of ($PP^{Me}P$)CoI_2 (**20**). (right) First order kinetics analysis of styrene %.

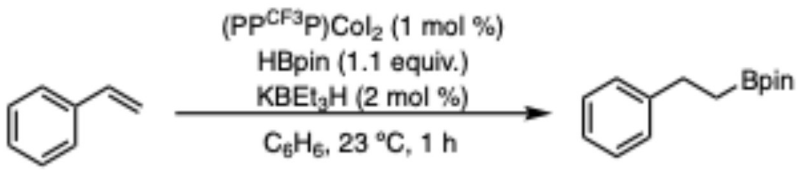

*Hydroboration of styrene with 1.0 mol % (PP^{CF3}P)CoI$_2$ (**3**), trial one.*

A 20 mL scintillation vial was charged with (PP^{CF3}P)CoI$_2$ (**3**) (6.9 mg, 7.0 mmol, 1.0 mol %) and a stir bar in an N_2 filled glovebox. To this was added C_6H_6 (285.9 mL), styrene (79.8 mL, 696 mmol, 1 equiv), HBpin (111.1 mL, 765.6 mmol, 1.1 equiv), and $KBEt_3H$ (139.2 mL, 100 mM in THF, 13.9 mmol, 2.0 mol %), resulting in an overall 11.3 mM solution with respect to catalyst. Upon addition of $KBEt_3H$, the solution was allowed to stir, and a timer was started. Aliquots were collected every 5 minutes for a total of 30 minutes.

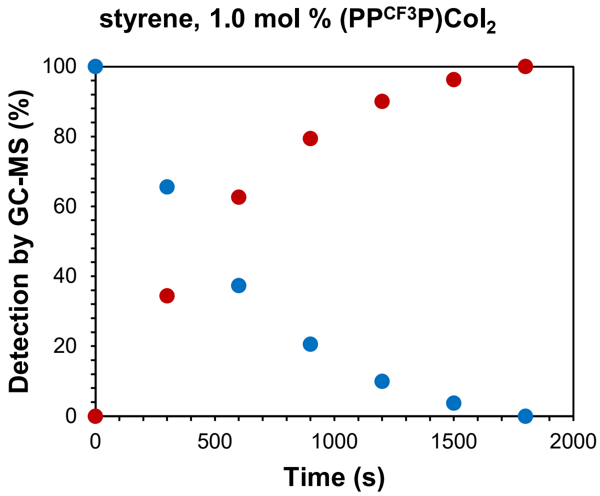

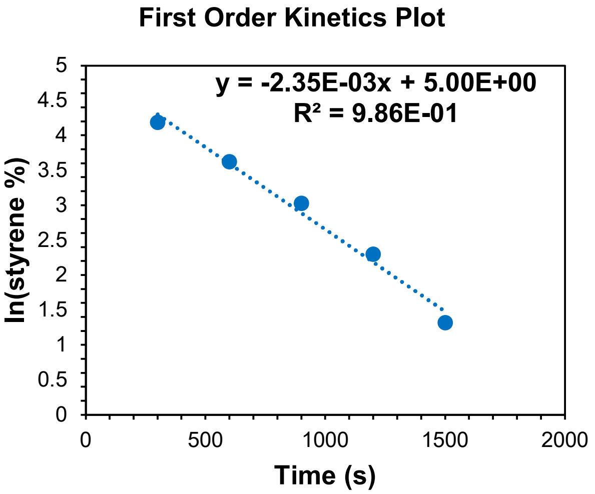

Figure S138: (left) Plotted timepoints of formation of linearly hydroborated styrene (red) and consumption of styrene (blue) versus time for 1.0 mol % loading of (PP^{CF3}P)CoI$_2$ (**3**). (right) First order kinetics analysis of styrene %.

*Hydroboration of styrene with 1.0 mol % ($PP^{CF3}P$)CoI_2 (**3**), trial two.*

A 20 mL scintillation vial was charged with ($PP^{CF3}P$)CoI_2 (**3**) (6.6 mg, 6.7 mmol, 1.0 mol %) and a stir bar in an N_2 filled glovebox. To this was added C_6H_6 (273.5 mL), styrene (76.3 mL, 666 mmol, 1 equiv), HBpin (106.3 mL, 732 mmol, 1.1 equiv), and $KBEt_3H$ (133.2 mL, 100 mM in THF, 13.3 mmol, 2.0 mol %), resulting in an overall 11.3 mM solution with respect to catalyst. Upon addition of $KBEt_3H$, the solution was allowed to stir, and a timer was started. Aliquots were collected every 5 minutes for a total of 20 minutes.

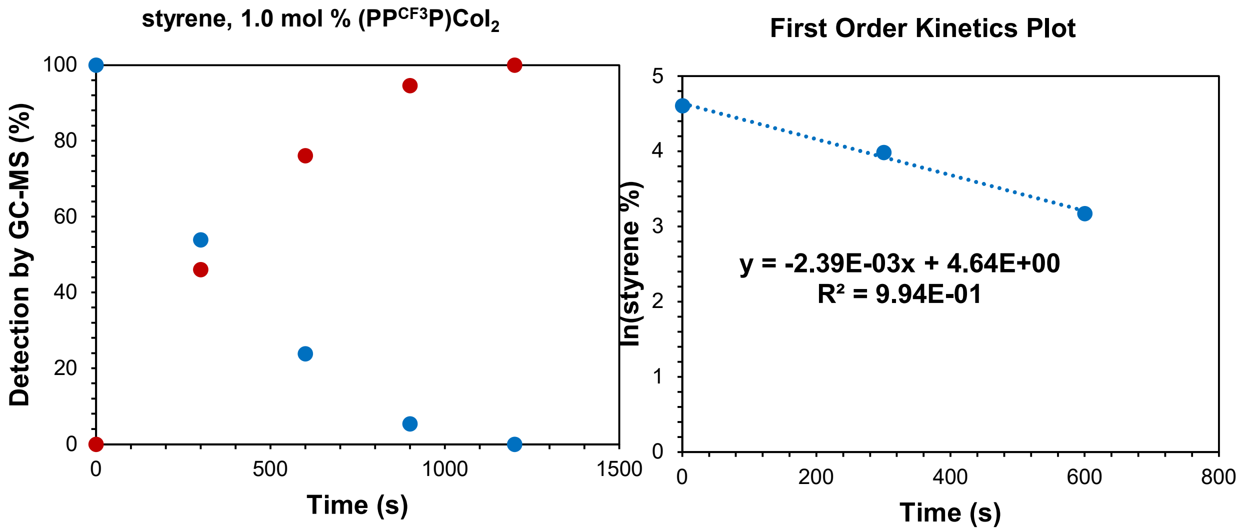

Figure S139: (left) Plotted timepoints of formation of linearly hydroborated styrene (red) and consumption of styrene (blue) versus time for 1.0 mol % loading of ($PP^{CF3}P$)CoI_2 (**3**). (right) First order kinetics analysis of styrene %.

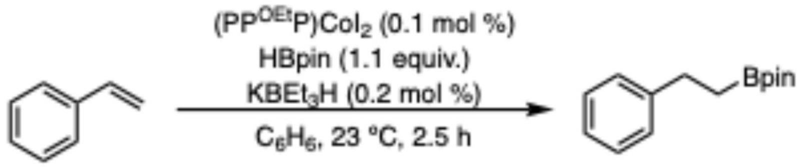

*Hydroboration of styrene with 0.1 mol % (PP^{OEt}P)CoI$_2$ (**15**), trial one.*

A 20 mL scintillation vial was charged with **15** (4.1 mg, 4.2 mmol, 0.1 mol %) and a stir bar in an N_2 filled glovebox. To this was added C_6H_6 (2.51 mL), styrene (485.6 mL, 4.24 mmol, 1 equiv), HBpin (676.4 mL, 4.66 mmol, 1.1 equiv), and $KBEt_3H$ (84.8 mL, 100 mM in THF, 8.48 mmol, 0.2 mol %), resulting in an overall 1.13 mM solution with respect to catalyst. Upon addition of $KBEt_3H$, the solution was allowed to stir, and a timer was started. Aliquots were collected every 15 minutes for a total of 2.5 h.

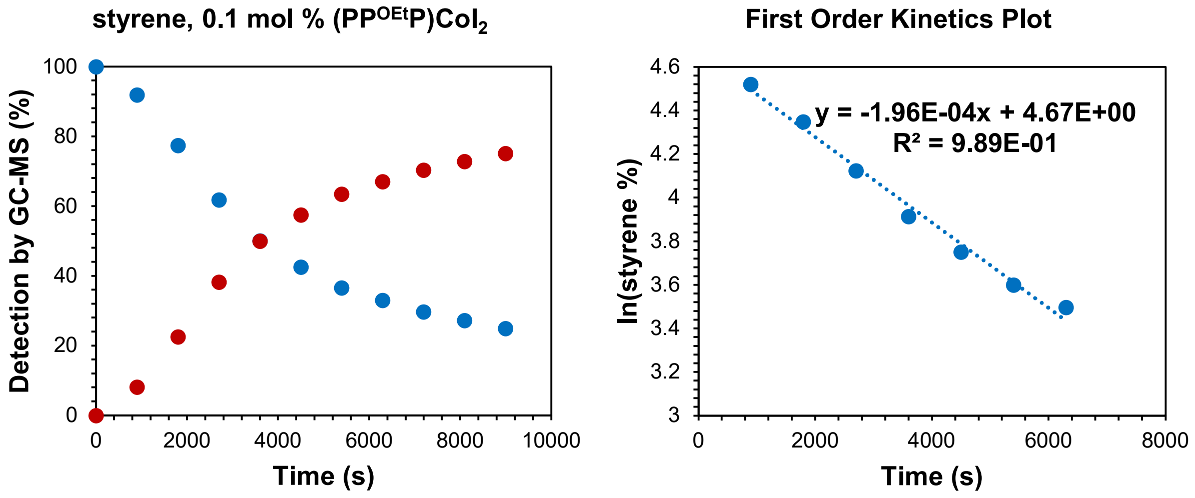

Figure 140: (left) Plotted timepoints of formation of linearly hydroborated styrene (red) and consumption of styrene (blue) versus time for 0.1 mol % loading of (PP^{OEt}P)CoI$_2$ (**15**). (right) First order kinetics analysis of styrene %.

*Hydroboration of styrene with 0.1 mol % (PP^{OEt}P)CoI$_2$ (**15**), trial two.*

A 20 mL scintillation vial was charged with (PP^{OEt}P)CoI$_2$ (3.6 mg, 3.7 mmol, 0.1 mol %) and a stir bar in an N_2 filled glovebox. To this was added C_6H_6 (2.21 mL), styrene (426.4 mL, 3.72 mmol, 1 equiv), HBpin (594.0 mL, 4.09 mmol, 1.1 equiv), and KBEt$_3$H (74.4 mL, 100 mM in THF, 7.44 mmol, 0.2 mol %), resulting in an overall 1.13 mM solution with respect to catalyst. Upon addition of KBEt$_3$H, the solution was allowed to stir, and a timer was started. Aliquots were collected every 15 minutes for a total of 2.5 h.

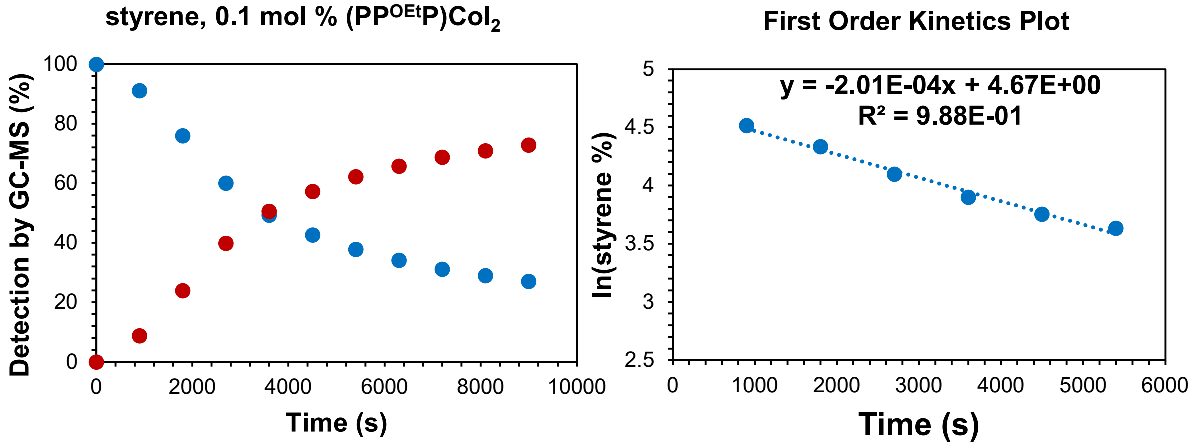

Figure 141: (left) Plotted timepoints of formation of linearly hydroborated styrene (red) and consumption of styrene (blue) versus time for 0.1 mol % loading of (PP^{OEt}P)CoI$_2$ (**15**). (right) First order kinetics analysis of styrene %.

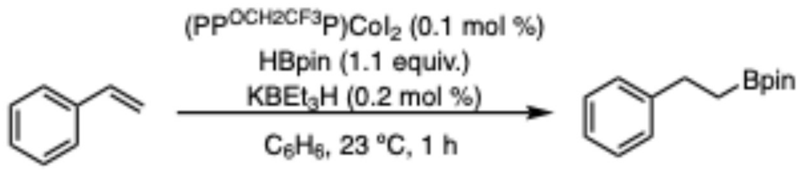

*Hydroboration of styrene with 0.1 mol % (PP^{OCH2CF3}P)CoI$_2$ (**17**), trial one.*

A 20 mL scintillation vial was charged with **17** (3.1 mg, 3.0 mmol, 0.1 mol %) and a stir bar in an N_2 filled glovebox. To this was added C_6H_6 (1.79 mL), styrene (347.8 mL, 3.04 mmol, 1 equiv), HBpin (484.4 mL, 3.34 mmol, 1.1 equiv), and $KBEt_3H$ (60.7 mL, 100 mM in THF, 6.07 mmol, 0.2 mol %), resulting in an overall 1.13 mM solution with respect to catalyst. Upon addition of $KBEt_3H$, the solution was allowed to stir, and a timer was started. Aliquots were collected every 5 minutes for a total of 1 h.

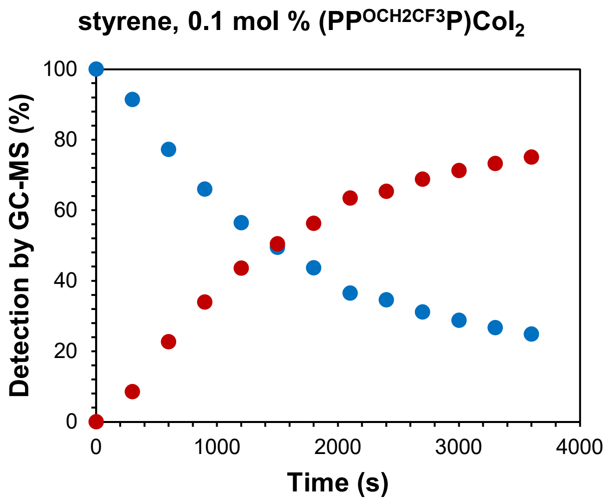

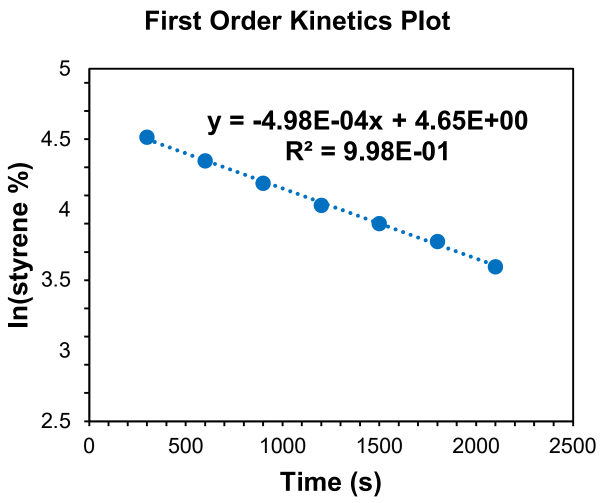

Figure 142: (left) Plotted timepoints of formation of linearly hydroborated styrene (red) and consumption of styrene (blue) versus time for 0.1 mol % loading of (PP^{OCH2CF3}P)CoI$_2$ (**17**). (right) First order kinetics analysis of styrene %.

*Hydroboration of styrene with 0.1 mol % ($PP^{OCH2CF3}P$)CoI_2 (**17**), trial two.*

A 20 mL scintillation vial was charged with **17** (4.5 mg, 4.4 mmol, 0.1 mol %) and a stir bar in an N_2 filled glovebox. To this was added C_6H_6 (2.61 mL), styrene (504.8 mL, 4.41 mmol, 1 equiv), HBpin (703.2 mL, 4.85 mmol, 1.1 equiv), and $KBEt_3H$ (88.1 mL, 100 mM in THF, 8.81 mmol, 0.2 mol %), resulting in an overall 1.13 mM solution with respect to catalyst. Upon addition of $KBEt_3H$, the solution was allowed to stir, and a timer was started. Aliquots were collected every 5 minutes for a total of 1 h.

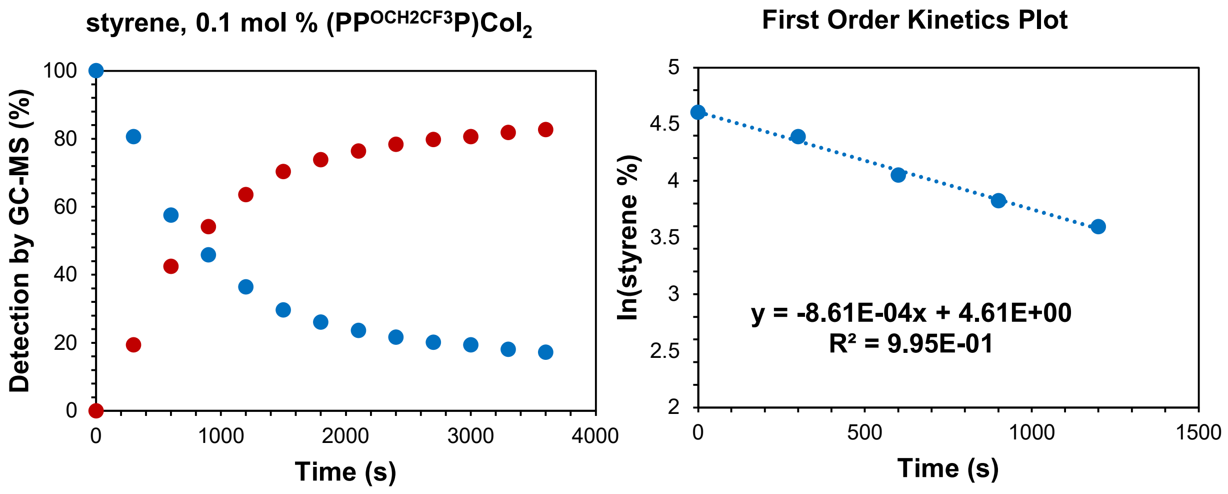

Figure 143: (left) Plotted timepoints of formation of linearly hydroborated styrene (red) and consumption of styrene (blue) versus time for 0.1 mol % loading of ($PP^{OCH2CF3}P$)CoI_2 (**17**). (right) First order kinetics analysis of styrene %.

Kinetic Studies: Hydroboration of α-methylstyrene

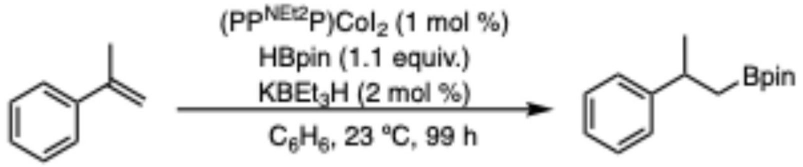

*Hydroboration of α-methylstyrene with 1.0 mol % (PP^{NEt2}P)CoI$_2$ (**13**), trial one.*

A 20 mL scintillation vial was charged with **13** (7.2 mg, 7.2 mmol, 1.0 mol %) and a stir bar in an N_2 filled glovebox. To this was added C_6H_6 (286.3 mL), α-methylstyrene (94.0 mL, 724 mmol, 1 equiv), HBpin (115.6 mL, 796.4 mmol, 1.1 equiv), and $KBEt_3H$ (144.8 mL, 100 mM in THF, 14.5 mmol, 2.0 mol %), resulting in an overall 11.3 mM solution with respect to catalyst. Upon addition of $KBEt_3H$, the solution was allowed to stir, and a timer was started. Aliquots were collected at the following timepoints: 2 h, 4 h, 10 h, 22 h, 34 h, 47 h, 59 h, 71 h, and 99 h.

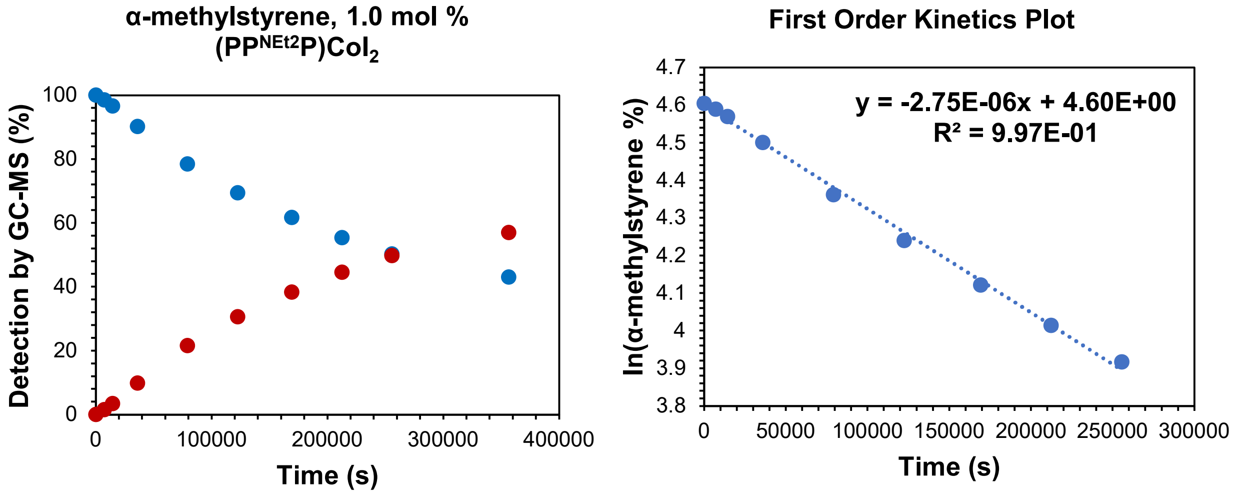

Figure 144: (left) Plotted timepoints of formation of linearly hydroborated *α*-methylstyrene (red) and consumption of α-methylstyrene (blue) versus time for 1.0 mol % loading of (PP^{NEt2}P)CoI$_2$ (**13**). (right) First order kinetics analysis of α-methylstyrene %

*Hydroboration of α-methylstyrene with 1.0 mol % (PP^{NEt2}P)CoI$_2$ (**13**), trial two.*

A 20 mL scintillation vial was charged with **13** (9.3 mg, 9.4 mmol, 1.0 mol %) and a stir bar in an N_2 filled glovebox. To this was added C_6H_6 (369.8 mL), α-methylstyrene (121.5 mL, 935.2 mmol, 1 equiv), HBpin (149.3 mL, 1.03 mmol, 1.1 equiv), and $KBEt_3H$ (187.0 mL, 100 mM in THF, 18.7 mmol, 2.0 mol %), resulting in an overall 11.3 mM solution with respect to catalyst. Upon addition of $KBEt_3H$, the solution was allowed to stir, and a timer was started. Aliquots were collected at the following timepoints: 2 h, 4 h, 10 h, 22 h, 34 h, 47 h, 59 h, 71 h, and 99 h.

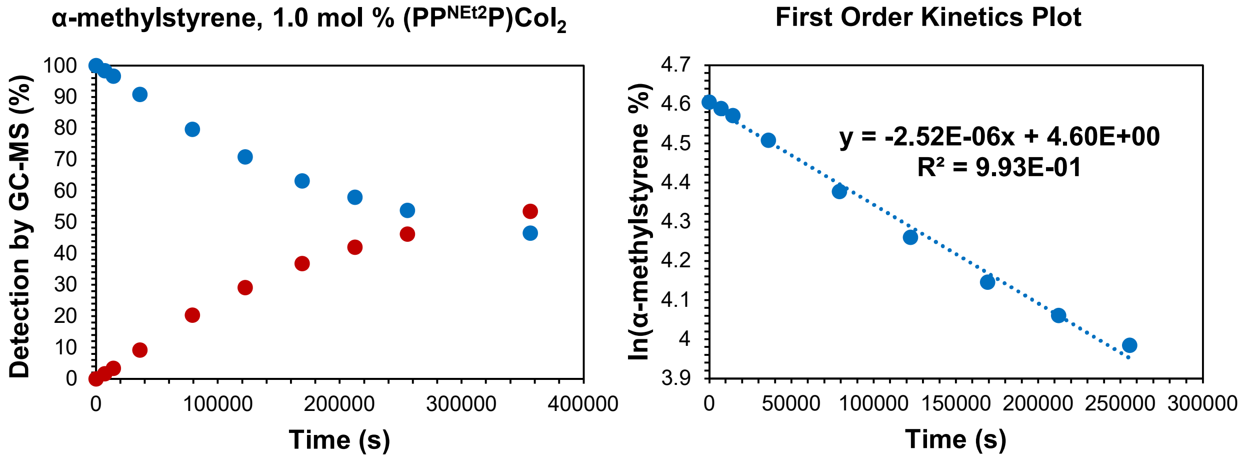

Figure 145: (left) Plotted timepoints of formation of linearly hydroborated *α*-methylstyrene (red) and consumption of *α*-methylstyrene (blue) versus time for 1.0 mol % loading of (PP^{NEt2}P)CoI$_2$ (**13**). (right) First order kinetics analysis of *α*-methylstyrene %.

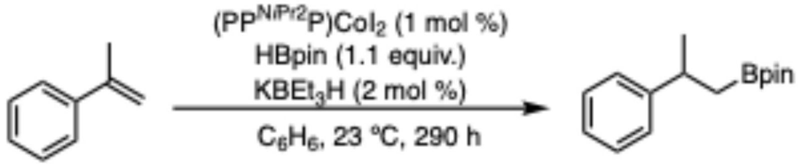

*Hydroboration of α-methylstyrene with 1.0 mol % (PP^{NiPr2}P)CoI$_2$ (**14**), trial one.*

A 20 mL scintillation vial was charged with **14** (6.5 mg, 6.4 mmol, 1.0 mol %) and a stir bar in an N_2 filled glovebox. To this was added C_6H_6 (251.4 mL), α-methylstyrene (82.6 mL, 636 mmol, 1 equiv), HBpin (101.5 mL, 699.2 mmol, 1.1 equiv), and $KBEt_3H$ (127.1 mL, 100 mM in THF, 12.7 mmol, 2.0 mol %), resulting in an overall 11.3 mM solution with respect to catalyst. Upon addition of $KBEt_3H$, the solution was allowed to stir, and a timer was started. Aliquots were collected at the following timepoints: 4 h, 12 h, 20 h, 28 h, 36 h, 48 h, 60 h, 74 h, 98 h, 171 h, and 290 h.

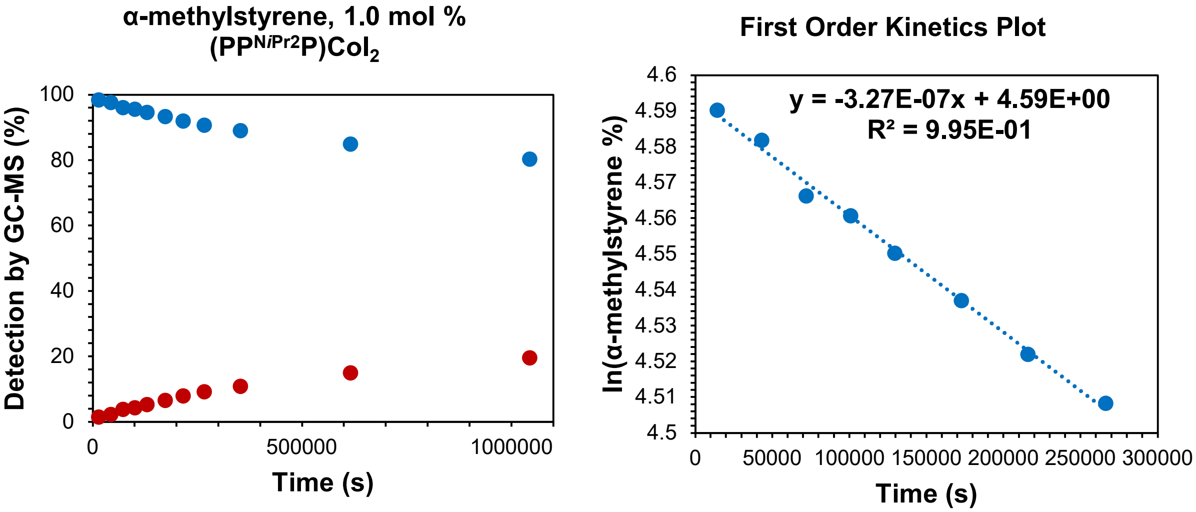

Figure 146: (left) Plotted timepoints of formation of linearly hydroborated α-methylstyrene (red) and consumption of α-methylstyrene (blue) versus time for 1.0 mol % loading of (PP^{NiPr2}P)CoI$_2$ (**14**). (right) First order kinetics analysis of α-methylstyrene %.

*Hydroboration of α-methylstyrene with 1.0 mol % ($PP^{NiPr2}P$)CoI_2 (**14**), trial two.*

A 20 mL scintillation vial was charged with **14** (7.2 mg, 7.0 mmol, 1.0 mol %) and a stir bar in an N_2 filled glovebox. To this was added C_6H_6 (278.5 mL), α-methylstyrene (91.4 mL, 704 mmol, 1 equiv), HBpin (112.4 mL, 774.5 mmol, 1.1 equiv), and $KBEt_3H$ (140.8 mL, 100 mM in THF, 14.1 mmol, 2.0 mol %), resulting in an overall 11.3 mM solution with respect to catalyst. Upon addition of $KBEt_3H$, the solution was allowed to stir, and a timer was started. Aliquots were collected at the following timepoints: 4 h, 12 h, 20 h, 28 h, 36 h, 48 h, 60 h, 74 h, 98 h, 171 h, and 290 h.

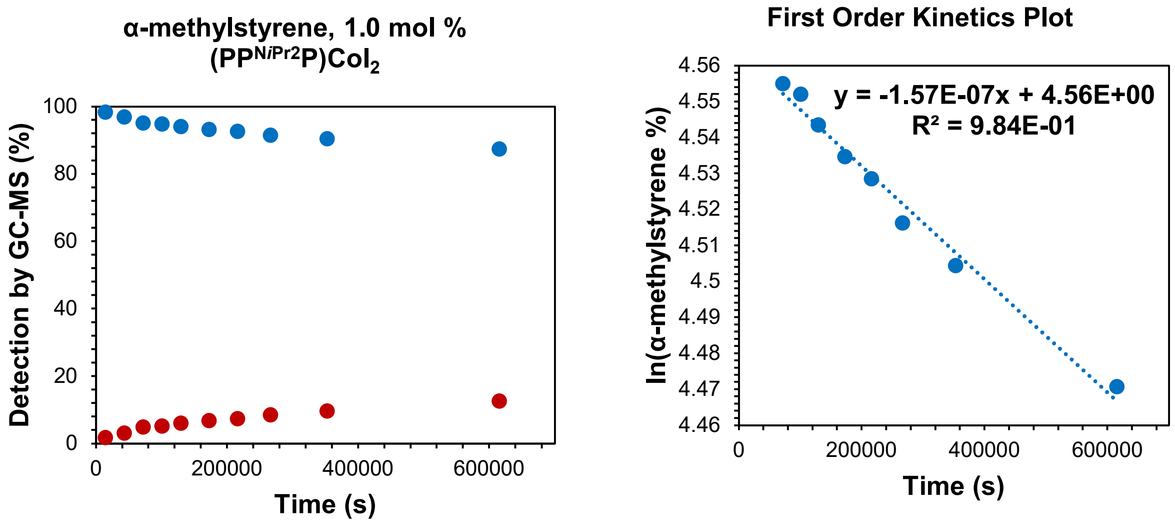

Figure 147: (left) Plotted timepoints of formation of linearly hydroborated *α*-methylstyrene (red) and consumption of *α*-methylstyrene (blue) versus time for 1.0 mol % loading of ($PP^{NiPr2}P$)CoI_2 (**14**). (right) First order kinetics analysis of *α*-methylstyrene %.

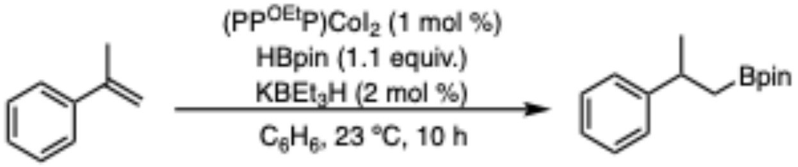

*Hydroboration of α-methylstyrene with 1.0 mol % (PP^{OEt}P)CoI$_2$ (**15**), trial one.*

A 20 mL scintillation vial was charged with **15** (3.3 mg, 3.4 mmol, 1.0 mol %) and a stir bar in an N_2 filled glovebox. To this was added C_6H_6 (134.9 mL), α-methylstyrene (44.3 mL, 341 mmol, 1 equiv), HBpin (54.5 mL, 375 mmol, 1.1 equiv), and $KBEt_3H$ (68.2 mL, 100 mM in THF, 6.82 mmol, 2.0 mol %), resulting in an overall 11.3 mM solution with respect to catalyst. Upon addition of $KBEt_3H$, the solution was allowed to stir, and a timer was started. Aliquots were collected every hour for a total of 10 h.

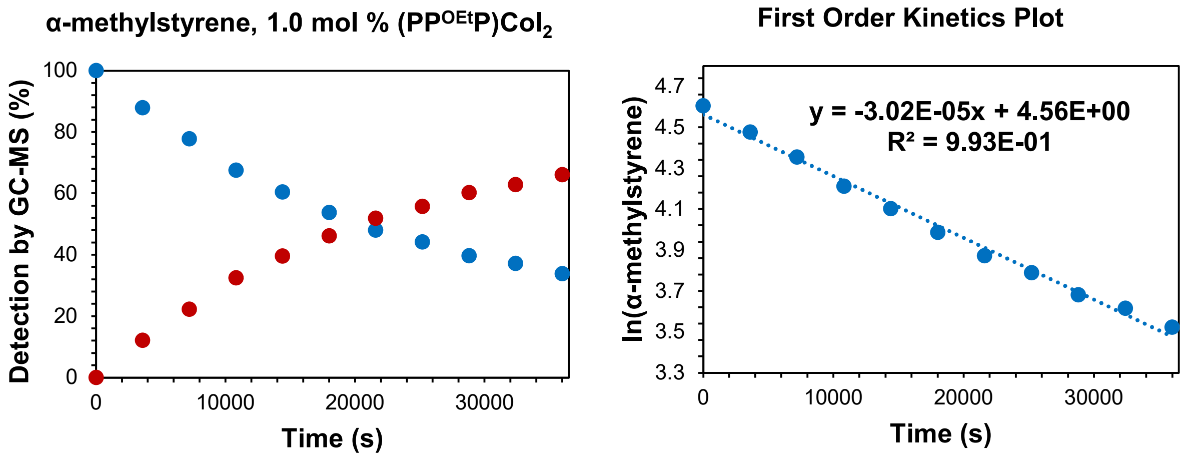

Figure 148: (left) Plotted timepoints of formation of linearly hydroborated α-methylstyrene (red) and consumption of α-methylstyrene (blue) versus time for 1.0 mol % loading of (PP^{OEt}P)CoI$_2$ (**15**). (right) First order kinetics analysis of α-methylstyrene %.

*Hydroboration of α-methylstyrene with 1.0 mol % (PP^{OEt}P)CoI$_2$ (**15**), trial two.*

A 20 mL scintillation vial was charged with **15** (2.4 mg, 2.5 mmol, 1.0 mol %) and a stir bar in an N_2 filled glovebox. To this was added C_6H_6 (98.1 mL), α-methylstyrene (32.2 mL, 248 mmol, 1 equiv), HBpin (39.6 mL, 273 mmol, 1.1 equiv), and $KBEt_3H$ (49.6 mL, 100 mM in THF, 4.96 mmol, 2.0 mol %), resulting in an overall 11.3 mM solution with respect to catalyst. Upon addition of $KBEt_3H$, the solution was allowed to stir, and a timer was started. Aliquots were collected every hour for a total of 10 h.

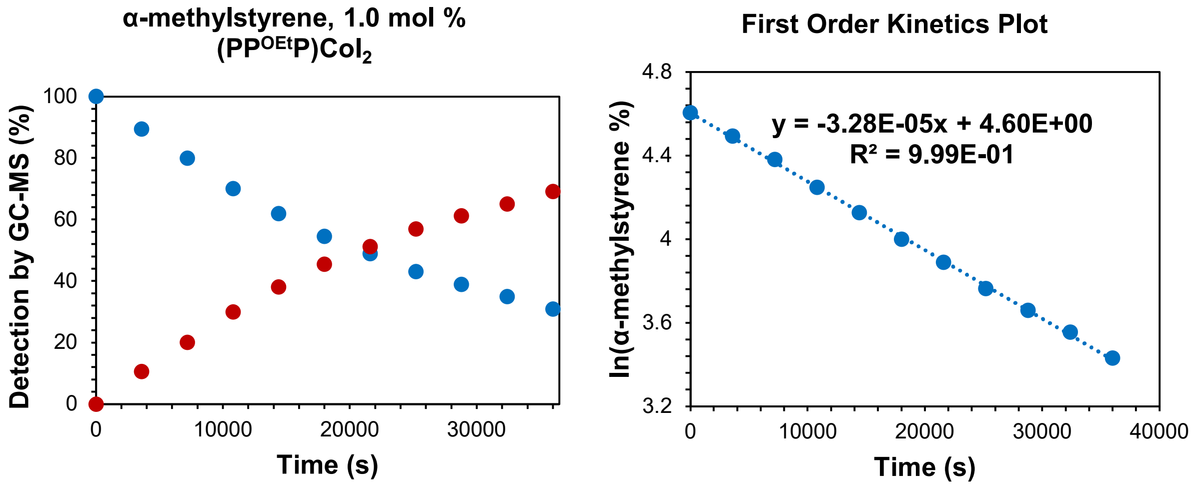

Figure 149: (left) Plotted timepoints of formation of linearly hydroborated *α*-methylstyrene (red) and consumption of *α*-methylstyrene (blue) versus time for 1.0 mol % loading of (PP^{OEt}P)CoI$_2$ (**15**). (right) First order kinetics analysis of *α*-methylstyrene %.

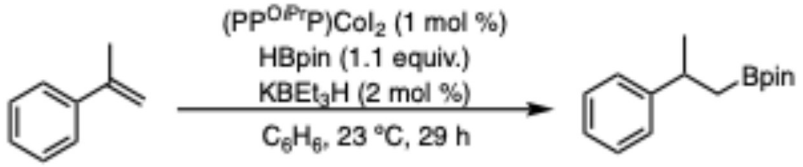

*Hydroboration of α-methylstyrene with 1.0 mol % (PP^{OiPr}P)CoI$_2$ (**16**), trial one.*

A 20 mL scintillation vial was charged with **16** (4.8 mg, 4.9 mmol, 2.0 mol %) and a stir bar in an N_2 filled glovebox. To this was added C_6H_6 (193.4 mL), α-methylstyrene (63.5 mL, 489 mmol, 1 equiv), HBpin (78.1 mL, 538 mmol, 1.1 equiv), and $KBEt_3H$ (97.8 mL, 100 mM in THF, 9.78 mmol, 2.0 mol %), resulting in an overall 11.3 mM solution with respect to catalyst. Upon addition of $KBEt_3H$, the solution was allowed to stir, and a timer was started. Aliquots were collected every two hours for a total of 14 h, with additional timepoints at 25 h and 29 h.

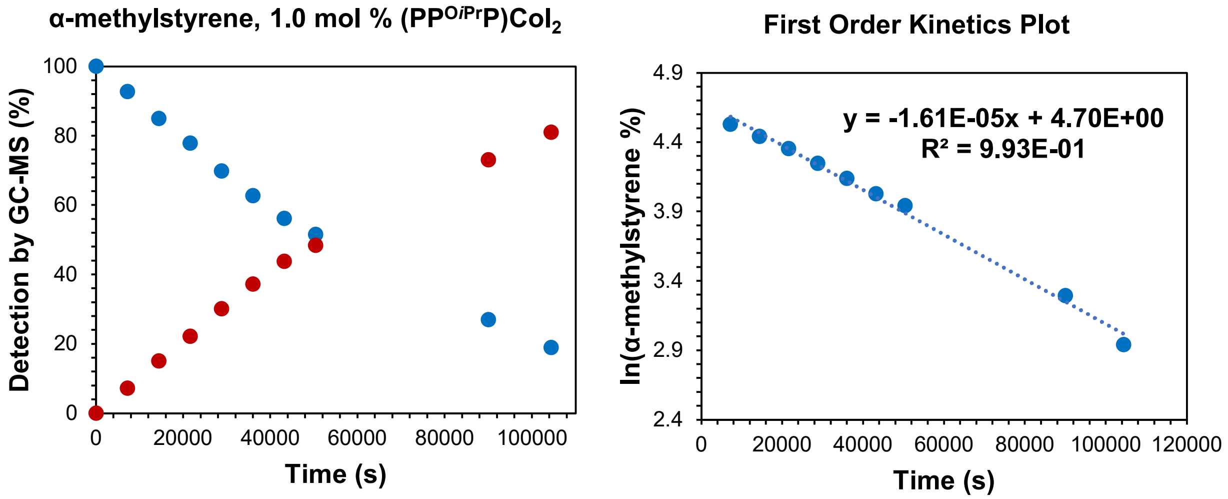

Figure 150: (left) Plotted timepoints of formation of linearly hydroborated α-methylstyrene (red) and consumption of α-methylstyrene (blue) versus time for 1.0 mol % loading of (PP^{OiPr}P)CoI$_2$ (**16**). (right) First order kinetics analysis of α-methylstyrene %.

*Hydroboration of α-methylstyrene with 1.0 mol % (PP^{OiPr}P)CoI$_2$ (**16**), trial two.*

A 20 mL scintillation vial was charged with **16** (3.2 mg, 3.3 mmol, 1.0 mol %) and a stir bar in an N_2 filled glovebox. To this was added C_6H_6 (129.0 mL), α-methylstyrene (42.3 mL, 326 mmol, 1 equiv), HBpin (52.0 mL, 359 mmol, 1.1 equiv), and $KBEt_3H$ (65.2 mL, 100 mM in THF, 6.52 mmol, 2.0 mol %), resulting in an overall 11.3 mM solution with respect to catalyst. Upon addition of $KBEt_3H$, the solution was allowed to stir, and a timer was started. Aliquots were collected every two hours for a total of 14 h, with additional timepoints at 25 h and 29 h.

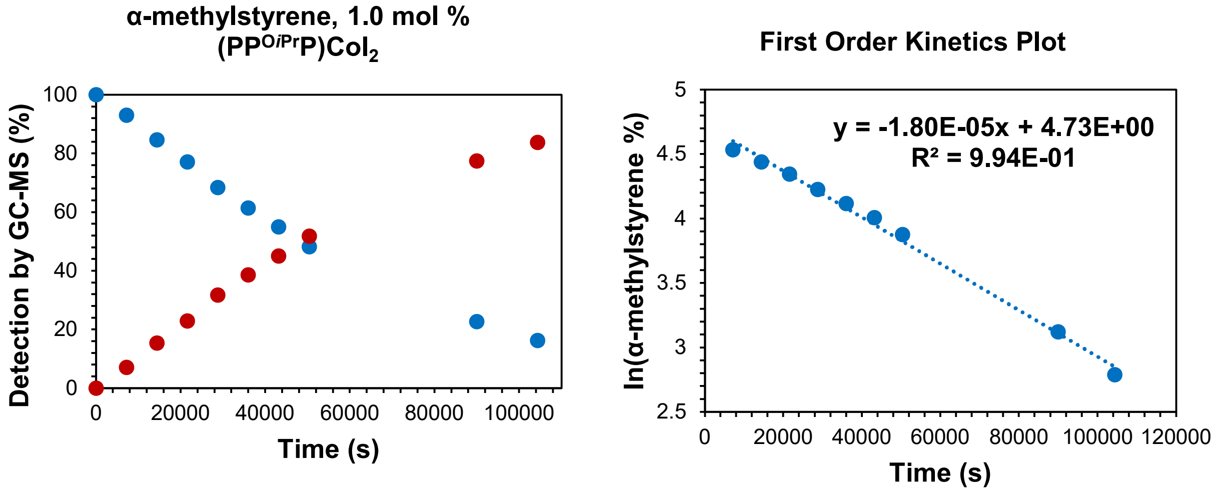

Figure 151: (left) Plotted timepoints of formation of linearly hydroborated α-methylstyrene (red) and consumption of α-methylstyrene (blue) versus time for 1.0 mol % loading of (PP^{OiPr}P)CoI$_2$ (**16**). (right) First order kinetics analysis of α-methylstyrene %.

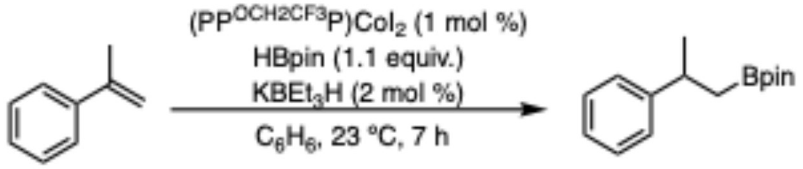

*Hydroboration of α-methylstyrene with 1.0 mol % ($PP^{OCH2CF3}P$)CoI_2 (**17**), trial one.*

A 20 mL scintillation vial was charged with **17** (6.7 mg, 6.6 mmol, 1.0 mol %) and a stir bar in an N_2 filled glovebox. To this was added C_6H_6 (259.4 mL), α-methylstyrene (85.2 mL, 656 mmol, 1 equiv), HBpin (104.7 mL, 721.6 mmol, 1.1 equiv), and $KBEt_3H$ (131.2 mL, 100 mM in THF, 13.1 mmol, 2.0 mol %), resulting in an overall 11.3 mM solution with respect to catalyst. Upon addition of $KBEt_3H$, the solution was allowed to stir, and a timer was started. Aliquots were collected every hour for a total of 7 h.

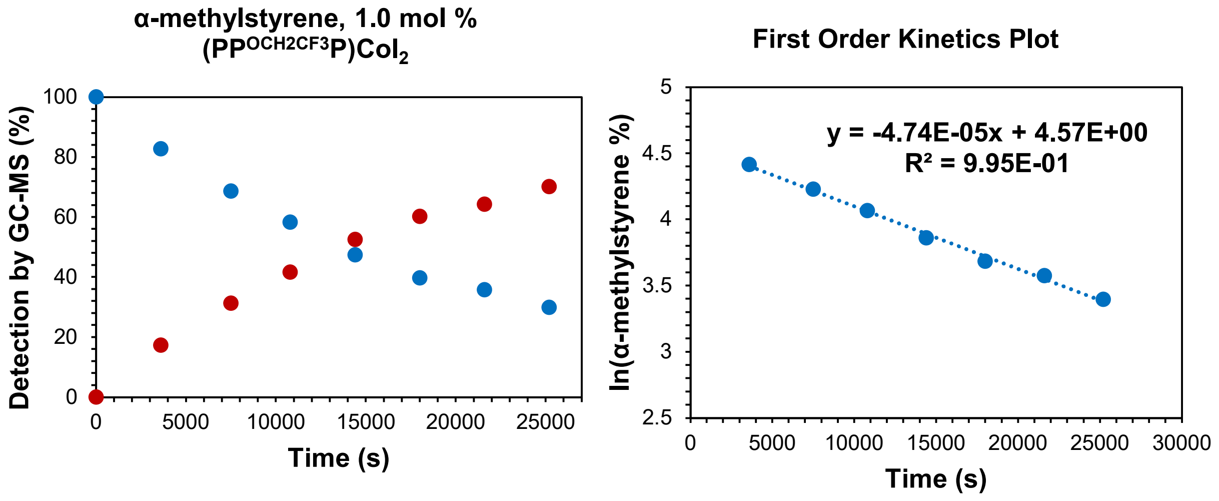

Figure 152: (left) Plotted timepoints of formation of linearly hydroborated α-methylstyrene (red) and consumption of α-methylstyrene (blue) versus time for 1.0 mol % loading of ($PP^{OCH2CF3}P$)CoI_2 (**17**). (right) First order kinetics analysis of α-methylstyrene.

*Hydroboration of α-methylstyrene with 1.0 mol % ($PP^{OCH2CF3}P$)CoI_2 (**17**), trial two.*

A 20 mL scintillation vial was charged with **17** (9.5 mg, 9.3 mmol, 1.0 mol %) and a stir bar in an N_2 filled glovebox. To this was added C_6H_6 (367.8 mL), αmethylstyrene (120.8 mL, 930.1 mmol, 1 equiv), HBpin (148.5 mL, 1.02 mmol, 1.1 equiv), and $KBEt_3H$ (186.0 mL, 100 mM in THF, 18.6 mmol, 2.0 mol %), resulting in an overall 11.3 mM solution with respect to catalyst. Upon addition of $KBEt_3H$, the solution was allowed to stir, and a timer was started. Aliquots were collected every hour for a total of 7 h.

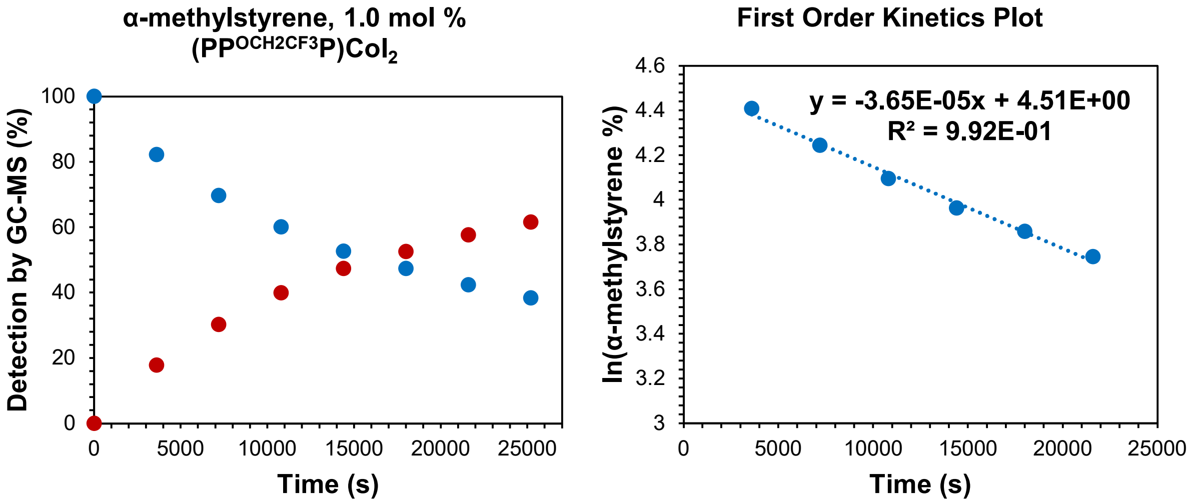

Figure 153: (left) Plotted timepoints of formation of linearly hydroborated *α*-methylstyrene (red) and consumption of *α*-methylstyrene (blue) versus time for 1.0 mol % loading of ($PP^{OCH2CF3}P$)CoI_2 (**1**7). (right) First order kinetics analysis of *α*-methylstyrene %.

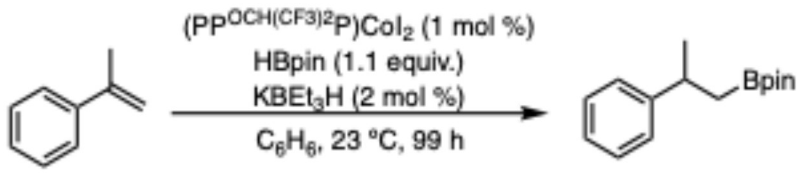

*Hydroboration of α-methylstyrene with 1.0 mol % (PP$^{OCH(CF3)2}$P)CoI$_2$ (**18**), trial one.*

A 20 mL scintillation vial was charged with **18** (8.0 mg, 7.3 mmol, 1.0 mol %) and a stir bar in an N_2 filled glovebox. To this was added C_6H_6 (290.4 mL), α-methylstyrene (95.4 mL, 734 mmol, 1 equiv), HBpin (117.2 mL, 808 mmol, 1.1 equiv), and KBEt$_3$H (146.9 mL, 100 mM in THF, 14.7 mmol, 2.0 mol %), resulting in an overall 11.3 mM solution with respect to catalyst. Upon addition of KBEt$_3$H, the solution was allowed to stir, and a timer was started. Aliquots were collected at the following timepoints: 1 h, 2 h, 4 h, 6 h, 10 h, 22 h, 34 h, 47 h, 59 h, 71 h, and 99 h.

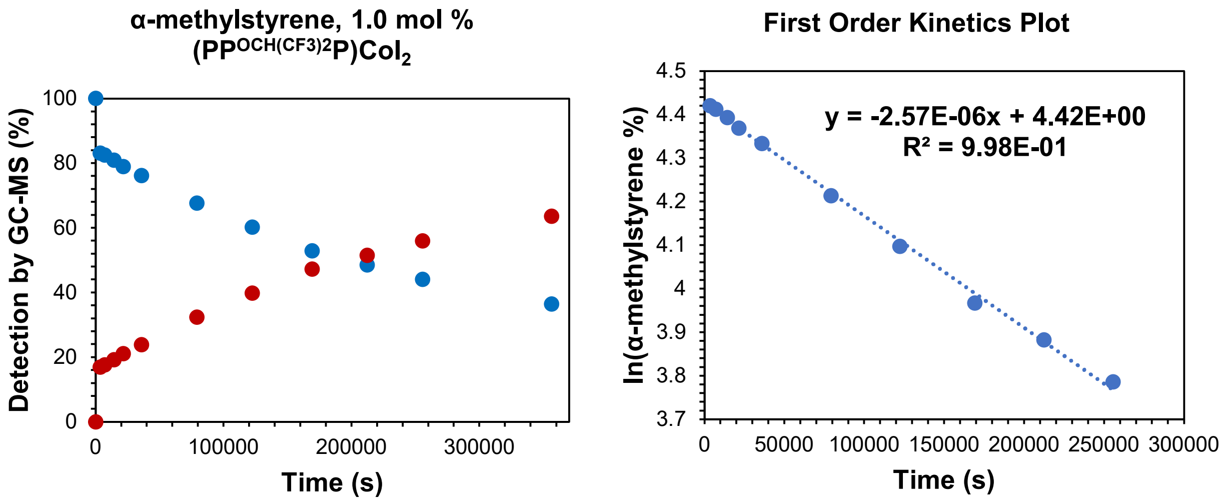

Figure 154: (left) Plotted timepoints of formation of linearly hydroborated α-methylstyrene (red) and consumption of α-methylstyrene (blue) versus time for 1.0 mol % loading of (PP$^{OCH(CF3)2}$P)CoI$_2$ (**18**). (right) First order kinetics analysis of α-methylstyrene %.

*Hydroboration of α-methylstyrene with 1.0 mol % (PP$^{OCH(CF3)2}$P)CoI$_2$ (**18**), trial two.*

A 20 mL scintillation vial was charged with **18** (7.4 mg, 6.8 mmol, 1.0 mol%) and a stir bar in an N_2 filled glovebox. To this was added C_6H_6 (268.6 mL), α-methylstyrene (88.2 mL, 679 mmol, 1 equiv), HBpin (108.4 mL, 747.2 mmol, 1.1 equiv), and KBEt$_3$H (135.9 mL, 100 mM in THF, 13.6 mmol, 2.0 mol %), resulting in an overall 11.3 mM solution with respect to catalyst. Upon addition of KBEt$_3$H, the solution was allowed to stir, and a timer was started. Aliquots were collected at the following timepoints: 1 h, 2 h, 4 h, 6 h, 10 h, 22 h, 34 h, 47 h, 59 h, 71 h, and 99 h.

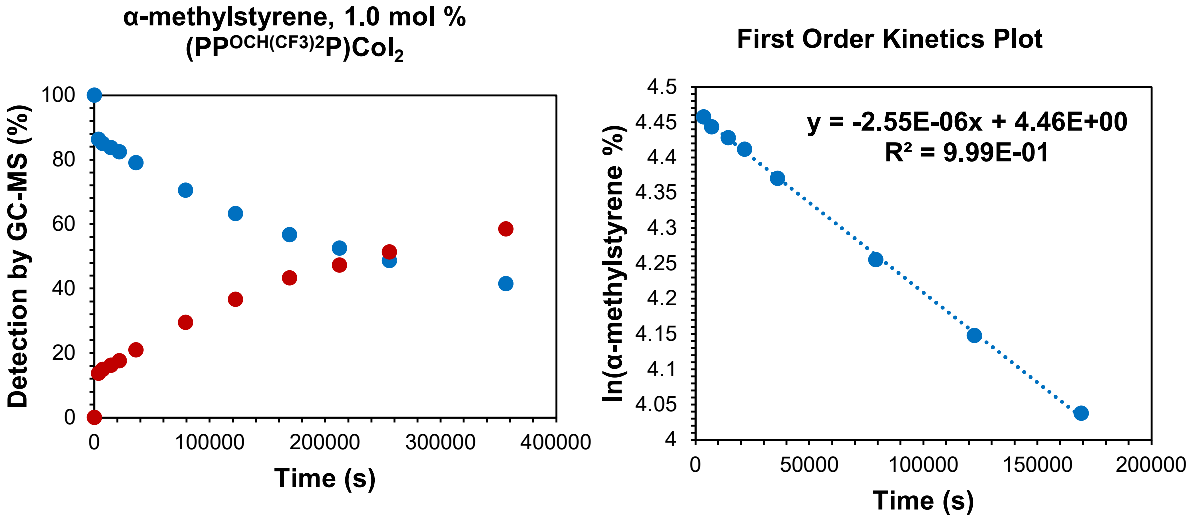

Figure 155: (left*)* Plotted timepoints of formation of linearly hydroborated α-methylstyrene (red) and consumption of α-methylstyrene (blue) versus time for 1.0 mol % loading of (PP$^{OCH(CF3)2}$P)CoI$_2$ (***18***). (right) First order kinetics analysis of α-methylstyrene %.

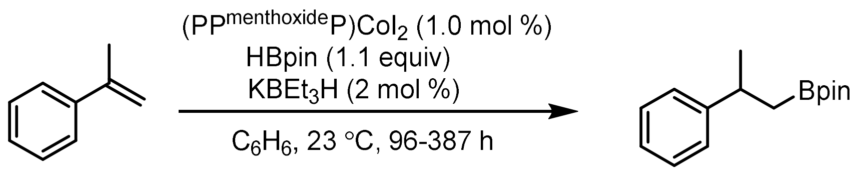

*Hydroboration of α-methylstyrene with 1.0 mol % (PP^{menthoxide}P)CoI$_2$ (**19**), trial one.*

A 20 mL scintillation vial was charged with **19** (9.7 mg, 9.0 mmol, 1.0 mol %) and a stir bar in an N_2 filled glovebox. To this was added C_6H_6 (356.0 mL), α-methylstyrene (116.9 mL, 900.1 mmol, 1 equiv), HBpin (143.7 mL, 990.2 mmol, 1.1 equiv), and $KBEt_3H$ (180.0 mL, 100 mM in THF, 18.0 mmol, 2.0 mol %), resulting in an overall 11.3 mM solution with respect to catalyst. Upon addition of $KBEt_3H$, the solution was allowed to stir, and a timer was started. Aliquots were collected at the following timepoints: 4 h, 12 h, 20 h, 28 h, 36 h, 48 h, 60 h, 74 h, 98 h, 121.95 h, 171 h, 290 h, and 387 h.

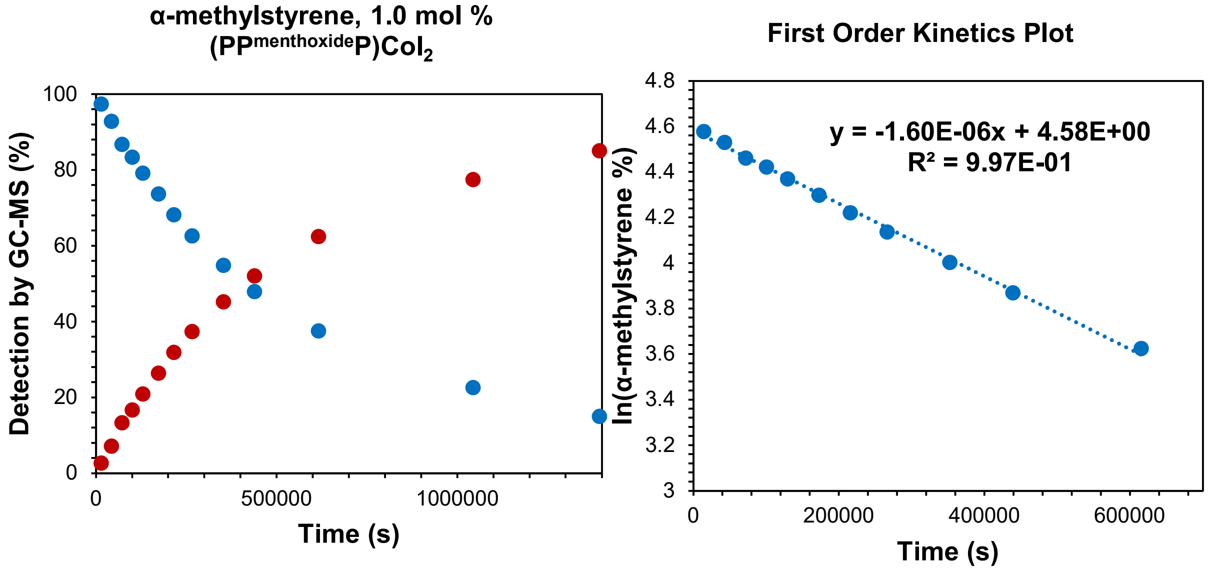

Figure 156: (left) Plotted timepoints of formation of linearly hydroborated α-methylstyrene (red) and consumption of α-methylstyrene (blue) versus time for 1.0 mol % loading of (PP^{menthoxide}P)CoI$_2$ (**19**). (right) First order kinetics analysis of α-methylstyrene %.

*Hydroboration of α-methylstyrene with 1.0 mol % (PP^{menthoxide}P)CoI$_2$ (**19**), trial two.*

A 20 mL scintillation vial was charged with **19** (8.8 mg, 8.2 mmol, 1.0 mol %) and a stir bar in an N_2 filled glovebox. To this was added C_6H_6 (323.0 mL), α-methylstyrene (106.1 mL, 816.6 mmol, 1 equiv), HBpin (130.3 mL, 898.3 mmol, 1.1 equiv), and $KBEt_3H$ (163.3 mL, 100 mM in THF, 16.3 mmol, 2.0 mol %), resulting in an overall 11.3 mM solution with respect to catalyst. Upon addition of $KBEt_3H$, the solution was allowed to stir, and a timer was started. Aliquots were collected at the following timepoints: 12 h, 25 h, 36 h, 48 h, 60 h, 72 h, and 96 h.

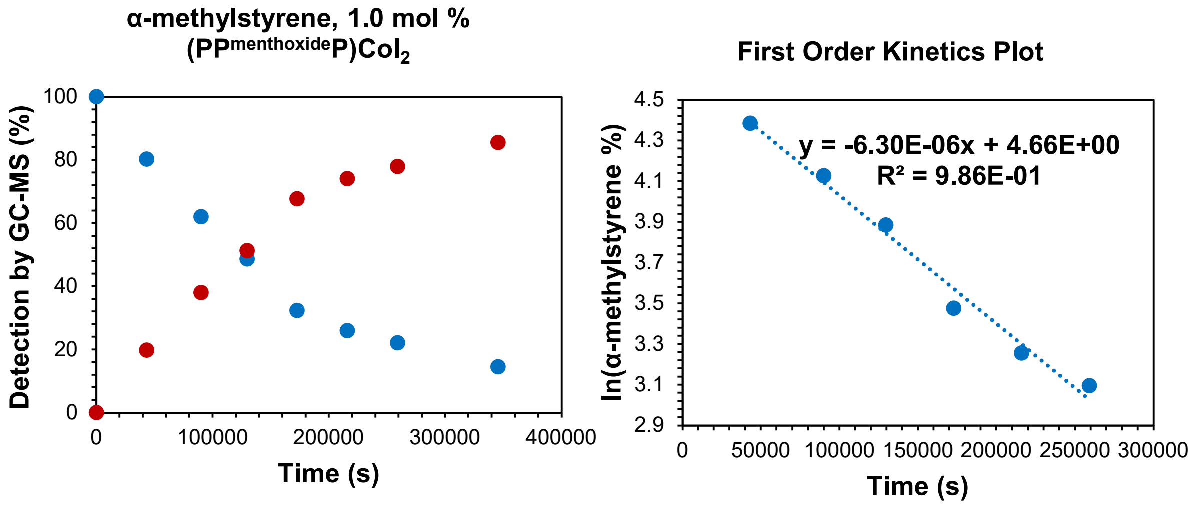

Figure 157: (left) Plotted timepoints of formation of linearly hydroborated α-methylstyrene (red) and consumption of α-methylstyrene (blue) versus time for 1.0 mol % loading of (PP^{menthoxide}P)CoI$_2$ (**19**). (right) First order kinetics analysis of α-methylstyrene %.

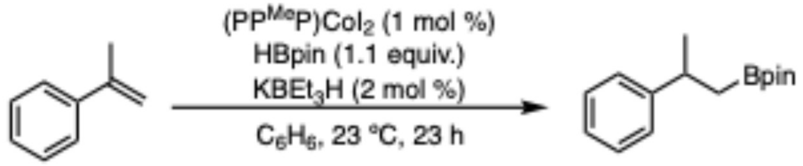

*Hydroboration of α-methylstyrene with 1.0 mol % (PP^{Me}P)CoI$_2$ (**20**), trial one.*

A 20 mL scintillation vial was charged with **20** (4.3 mg, 4.6 mmol, 1.0 mol %) and a stir bar in an N_2 filled glovebox. To this was added C_6H_6 (181.4 mL), α-methylstyrene (59.6 mL, 459 mmol, 1 equiv), HBpin (73.2 mL, 505 mmol, 1.1 equiv), and $KBEt_3H$ (91.7 mL, 100 mM in THF, 9.17 mmol, 2.0 mol %), resulting in an overall 11.3 mM solution with respect to catalyst. Upon addition of $KBEt_3H$, the solution was allowed to stir, and a timer was started. Aliquots were collected at the following timepoints: 1 h, 3 h, 5 h, 7 h, 12 h, and 23 h.

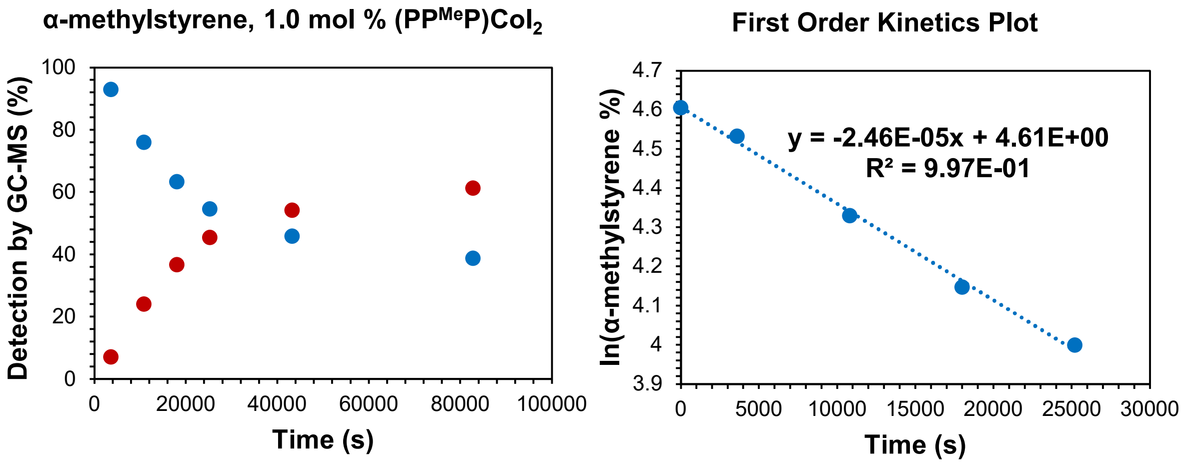

Figure 158: (left) Plotted timepoints of formation of linearly hydroborated α-methylstyrene (red) and consumption of α-methylstyrene (blue) versus time for 1.0 mol % loading of (PP^{Me}P)CoI$_2$ (**20**). (right) First order kinetics analysis of α-methylstyrene %.

*Hydroboration of α-methylstyrene with 1.0 mol % ($PP^{Me}P$)CoI_2 (**20**), trial two.*

A 20 mL scintillation vial was charged with **20** (2.9 mg, 3.1 mmol, 1.0 mol %) and a stir bar in an N_2 filled glovebox. To this was added C_6H_6 (122.3 mL), α-methylstyrene (40.2 mL, 309 mmol, 1 equiv), HBpin (49.4 mL, 340 mmol, 1.1 equiv), and $KBEt_3H$ (61.9 mL, 100 mM in THF, 6.19 mmol, 2.0 mol %), resulting in an overall 11.3 mM solution with respect to catalyst. Upon addition of $KBEt_3H$, the solution was allowed to stir, and a timer was started. Aliquots were collected at the following timepoints: 1 h, 3 h, 5 h, 7 h, 12 h, and 23 h.

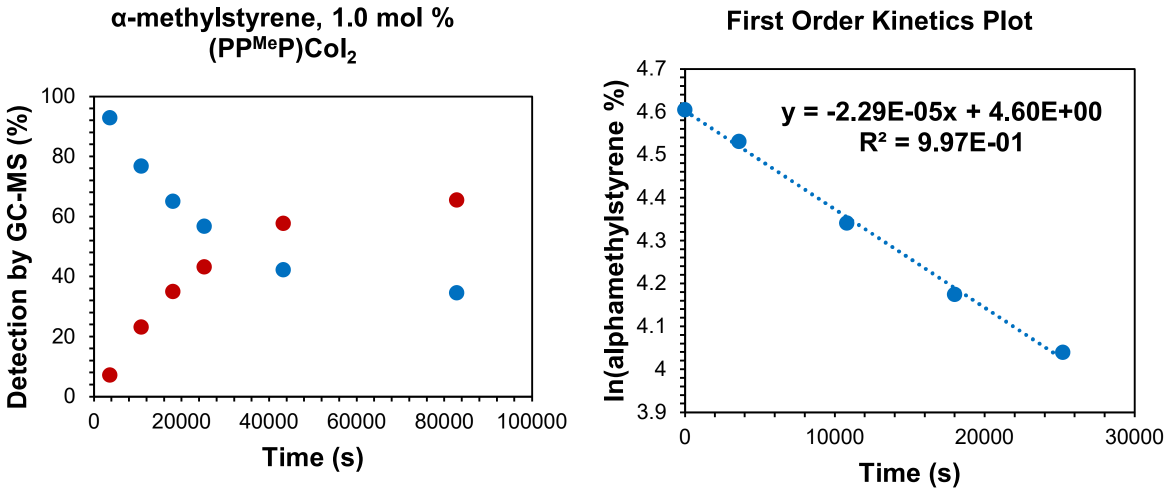

Figure 159: (left) Plotted timepoints of formation of linearly hydroborated *α*-methylstyrene (red) and consumption of *α*-methylstyrene (blue) versus time for 1.0 mol % loading of ($PP^{Me}P$)CoI_2 (**2*0***). (right) First order kinetics analysis of *α*-methylstyrene %.

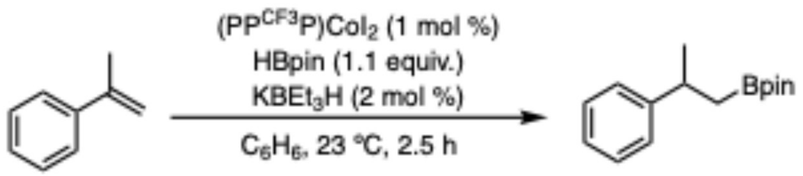

*Hydroboration of α-methylstyrene with 1.0 mol % (PP^{CF3}P)CoI$_2$ (**3**), trial one.*

A 20 mL scintillation vial was charged with (PP^{CF3}P)CoI$_2$ (**3**) (6.9 mg, 7.0 mmol, 1.0 mol %) and a stir bar in an N_2 filled glovebox. To this was added C_6H_6 (275.3 mL), α-methylstyrene (90.4 mL, 696 mmol, 1 equiv), HBpin (111.1 mL, 765.6 mmol, 1.1 equiv), and $KBEt_3H$ (139.2 mL, 100 mM in THF, 13.9 mmol, 2.0 mol %), resulting in an overall 11.3 mM solution with respect to catalyst. Upon addition of $KBEt_3H$, the solution was allowed to stir, and a timer was started. Aliquots were collected every 15 min for a total of 2.5 h.

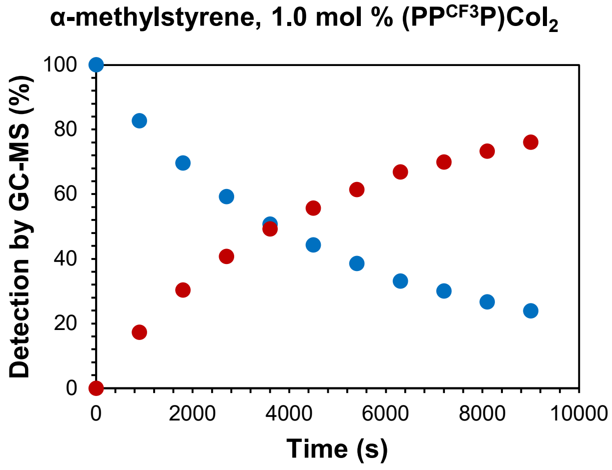

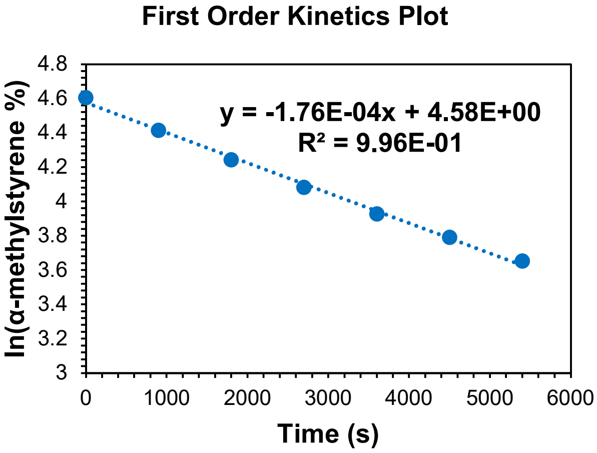

Figure 160: (left) Plotted timepoints of formation of linearly hydroborated α-methylstyrene (red) and consumption of α-methylstyrene (blue) versus time for 1.0 mol % loading of (PP^{CF3}P)CoI$_2$ (**3**). (right) First order kinetics analysis of α-methylstyrene %.

*Hydroboration of α-methylstyrene with 1.0 mol % ($PP^{CF3}P$)CoI_2 (**3**), trial two.*

A 20 mL scintillation vial was charged with ($PP^{CF3}P$)CoI_2 (**3**) (4.2 mg, 4.22 mmol, 1.0 mol %) and a stir bar in an N_2 filled glovebox. To this was added C_6H_6 (164.3 mL), alphamethylstyrene (55.0 mL, 424 mmol, 1 equiv), HBpin (67.6 mL, 466 mmol, 1.1 equiv), and $KBEt_3H$ (84.7 mL, 100 mM in THF, 8.47 mmol, 2.0 mol %), resulting in an overall 11.3 mM solution with respect to catalyst. Upon addition of $KBEt_3H$, the solution was allowed to stir, and a timer was started. Aliquots were collected every 15 min for a total of 2.5 h.

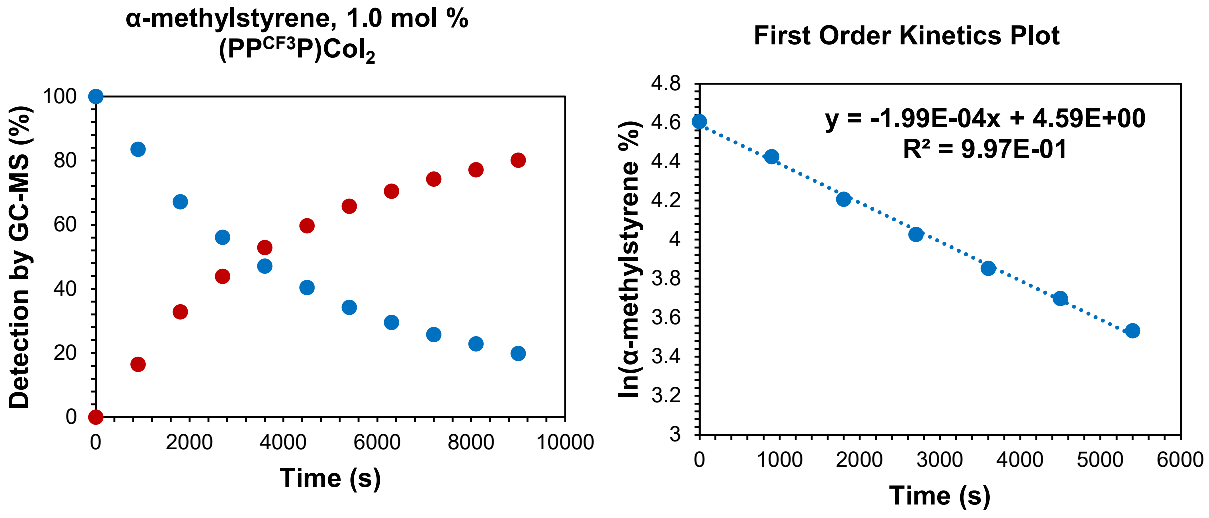

Figure 161: (left) Plotted timepoints of formation of linearly hydroborated α-methylstyrene (red) and consumption of α-methylstyrene (blue) versus time for 1.0 mol % loading of ($PP^{CF3}P$)CoI_2 (**3**). (right*)* First order kinetics analysis of α-methylstyrene %.

X-Ray Crystallography Data Collection and Refinement Details

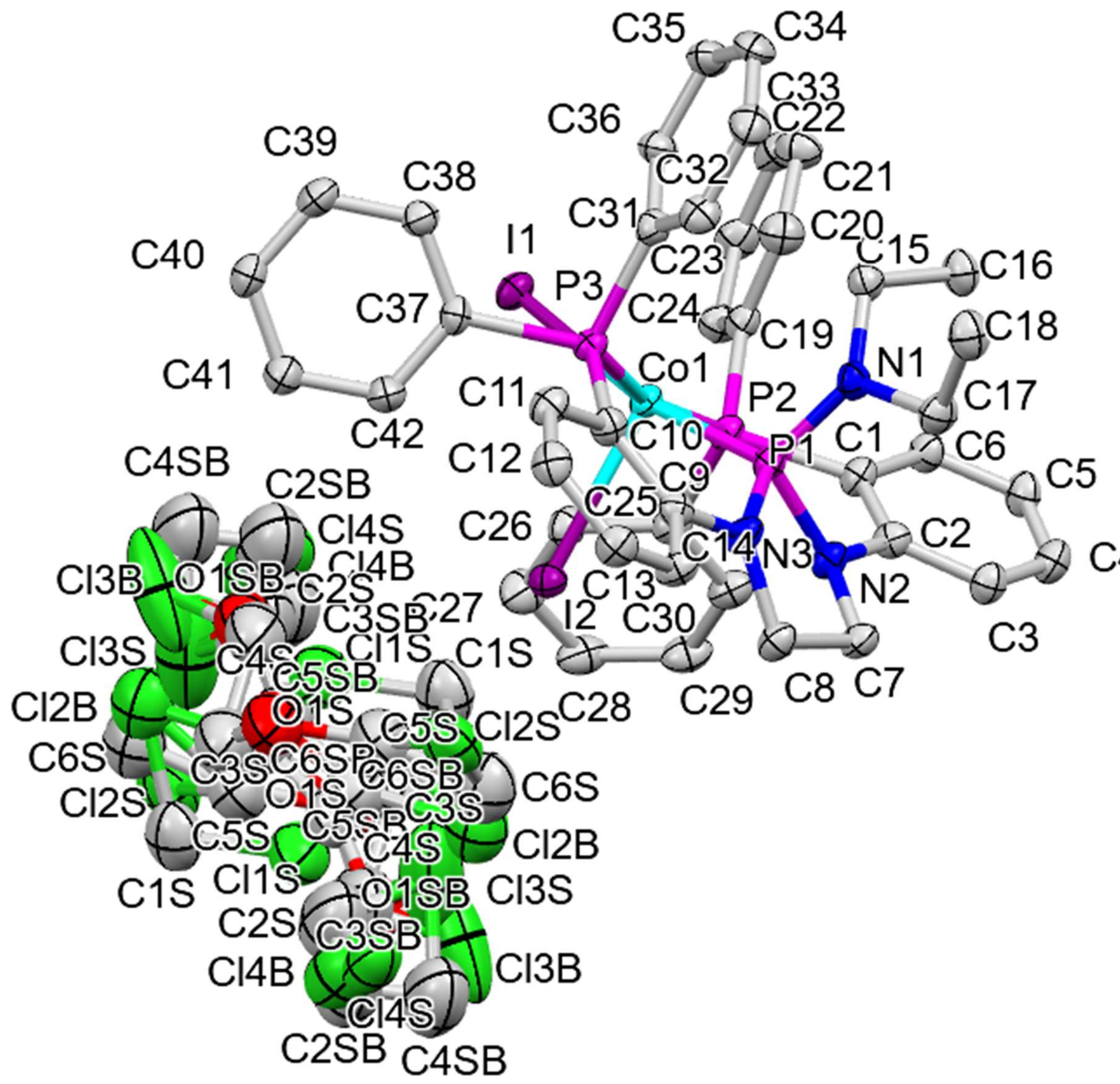

Figure S162. Fully labelled displacement ellipsoid representation of $(PP^{NEt2}P)CoI_2$ (**13**•$1.5(CH_2Cl_2)$, $0.25(Et_2O)$).

Data Collection and Refinement Details for 13.

The single crystal X-ray diffraction studies were carried out on a Bruker Kappa Photon III CPAD diffractometer equipped with Mo K_α radiation (λ = 0.71073 Å). A 0.221 x 0.102 x 0.063 mm piece of a red plate was mounted on a MiTeGen MicroMount with Paratone 24EX oil. Data were collected in a nitrogen gas stream at 100(2) K using ϕ and ϖ scans. Crystal-to-detector distance was 60 mm using variable exposure

time (1s-2s) depending on θ with a scan width of 1.0°. Data collection was 99.8% complete to 25.00° in θ (0.83 Å). A total of 117476 reflections were collected covering the indices, -13 ≤ h ≤ 13, -26 ≤ k ≤ 26, -30 ≤ l ≤ 30. 11443 reflections were found to be symmetry independent, with a R_{int} of 0.0383. Indexing and unit cell refinement indicated a primitive, monoclinic lattice. The space group was found to be $P2_1/n$. The data were integrated using the Bruker SAINT software program and scaled using the SADABS software program. Solution by dual-space method (SHELXT) produced a complete phasing model for refinement.

All nonhydrogen atoms were refined anisotropically by full-matrix least-squares (SHELXL-2014). All hydrogen atoms were placed using a riding model. Their positions were constrained relative to their parent atom using the appropriate HFIX command in SHELXL-2014. Crystallographic data are summarized in Table S3.

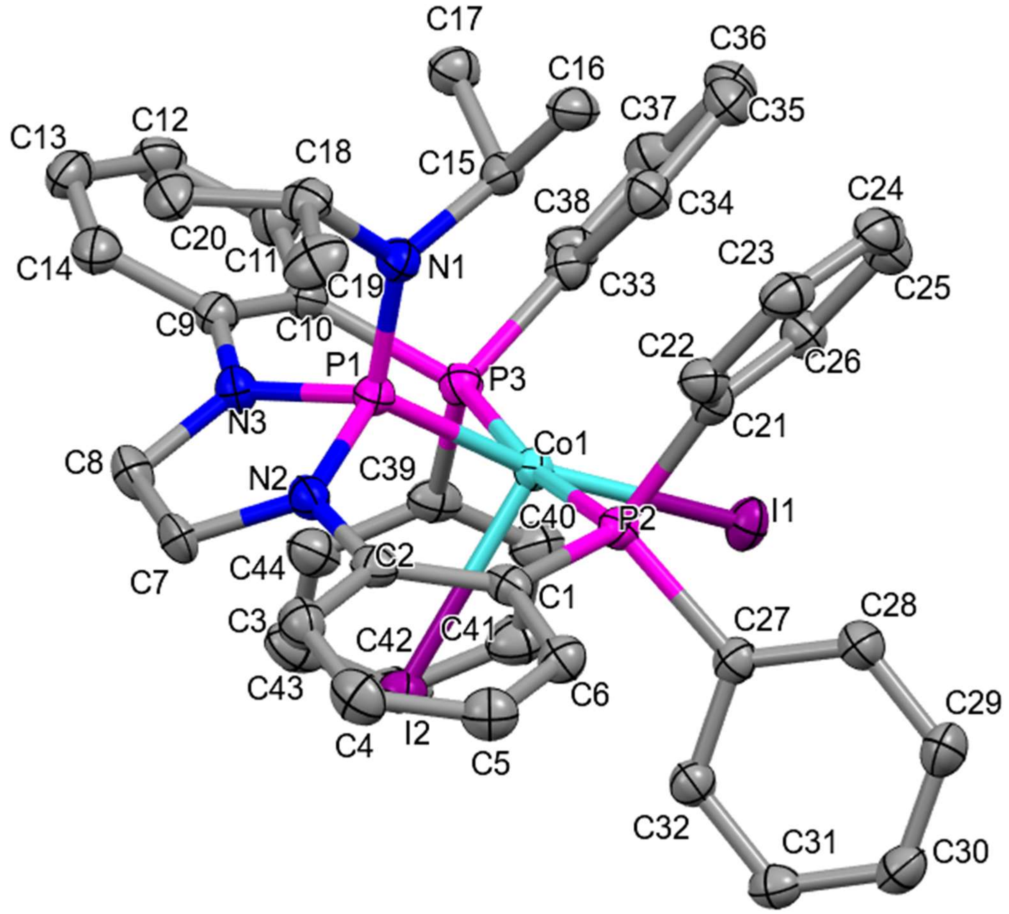

Figure S163. Fully labelled displacement ellipsoid representation of $(PP^{N\textit{i}Pr2}P)CoI_2$ (**14**).

Data Collection and Refinement Details for 14.

The single crystal X-ray diffraction studies were carried out on a Bruker Kappa Photon III CPAD diffractometer equipped with Mo K_α radiation (λ = 0.71073 Å). A 0.099 x 0.078 x 0.033 mm piece of an orange plate was mounted on a MiTeGen MicroMount with Paratone 24EX oil. Data were collected in a nitrogen gas stream at 100(2) K using ϕ and ϖ scans. Crystal-to-detector distance was 60 mm using variable exposure time (2s-5s) depending on θ with a scan width of 1.0°. Data collection was 99.9% complete to 25.00° in θ (0.83 Å). A total of 108233 reflections were collected covering the indices, $-13 \leq h \leq 12$, $-30 \leq k \leq 30$, $-21 \leq l \leq 21$. 8978 reflections were found to be symmetry independent, with a R_{int} of 0.0516. Indexing and unit cell refinement indicated a primitive, monoclinic lattice. The space group was found to be $P2_1/n$.

The data were integrated using the Bruker SAINT software program and scaled using the SADABS software program. Solution by dual-space method (SHELXT) produced a complete phasing model for refinement.

All nonhydrogen atoms were refined anisotropically by full-matrix least-squares (SHELXL-2014). All hydrogen atoms were placed using a riding model. Their positions were constrained relative to their parent atom using the appropriate HFIX command in SHELXL-2014. Crystallographic data are summarized in Table S3.

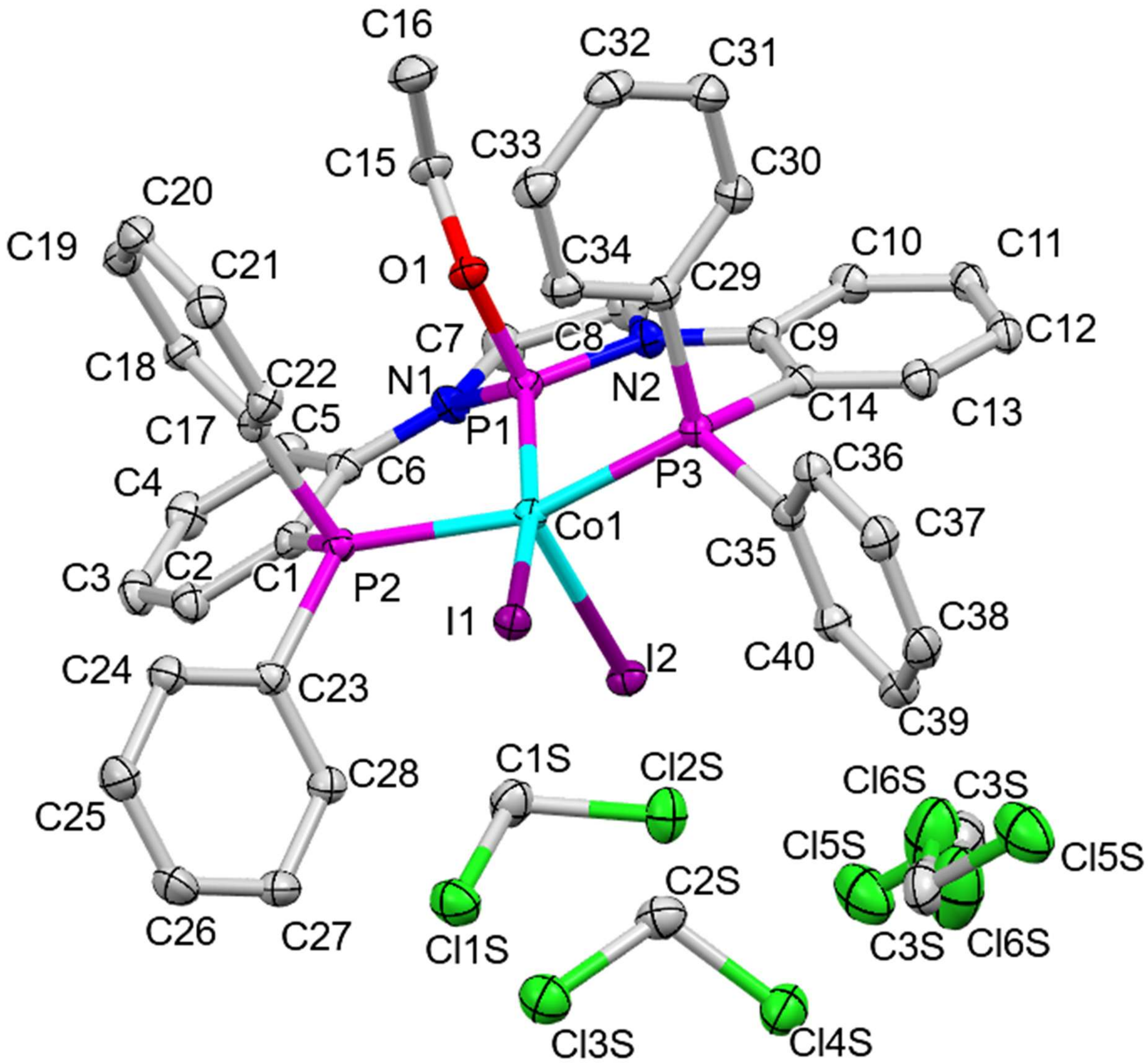

Figure S164. Fully labelled displacement ellipsoid representation of $(PP^{OEt}P)CoI_2$ (**15**).

Data Collection and Refinement Details for 15.

The single crystal X-ray diffraction studies were carried out on a Bruker Kappa Photon III CPAD diffractometer equipped with Mo K_α radiation (λ = 0.71073 Å). A 0.203 x 0.135 x 0.041 mm piece of a red block was mounted on a MiTeGen MicroMount with Paratone 24EX oil. Data were collected in a nitrogen gas stream at 100(2) K using ϕ and ϖ scans. Crystal-to-detector distance was 70 mm using variable exposure time (1s-4s) depending on θ with a scan width of 1.0°. Data collection was 99.0% complete to 25.00° in θ (0.83 Å). A total of 110477 reflections were collected covering the indices, -17 ≤ h ≤ 16, -17 ≤ k ≤ 17, -21

$\leq l \leq 21$. 11290 reflections were found to be symmetry independent, with a R_{int} of 0.0305. Indexing and unit cell refinement indicated a primitive, triclinic lattice. The space group was found to be *P*-1. The data were integrated using the Bruker SAINT software program and scaled using the SADABS software program. Solution by dual-space method (SHELXT) produced a complete phasing model for refinement.

All nonhydrogen atoms were refined anisotropically by full-matrix least-squares (SHELXL-2014). All hydrogen atoms were placed using a riding model. Their positions were constrained relative to their parent atom using the appropriate HFIX command in SHELXL-2014. Crystallographic data are summarized in Table S3.

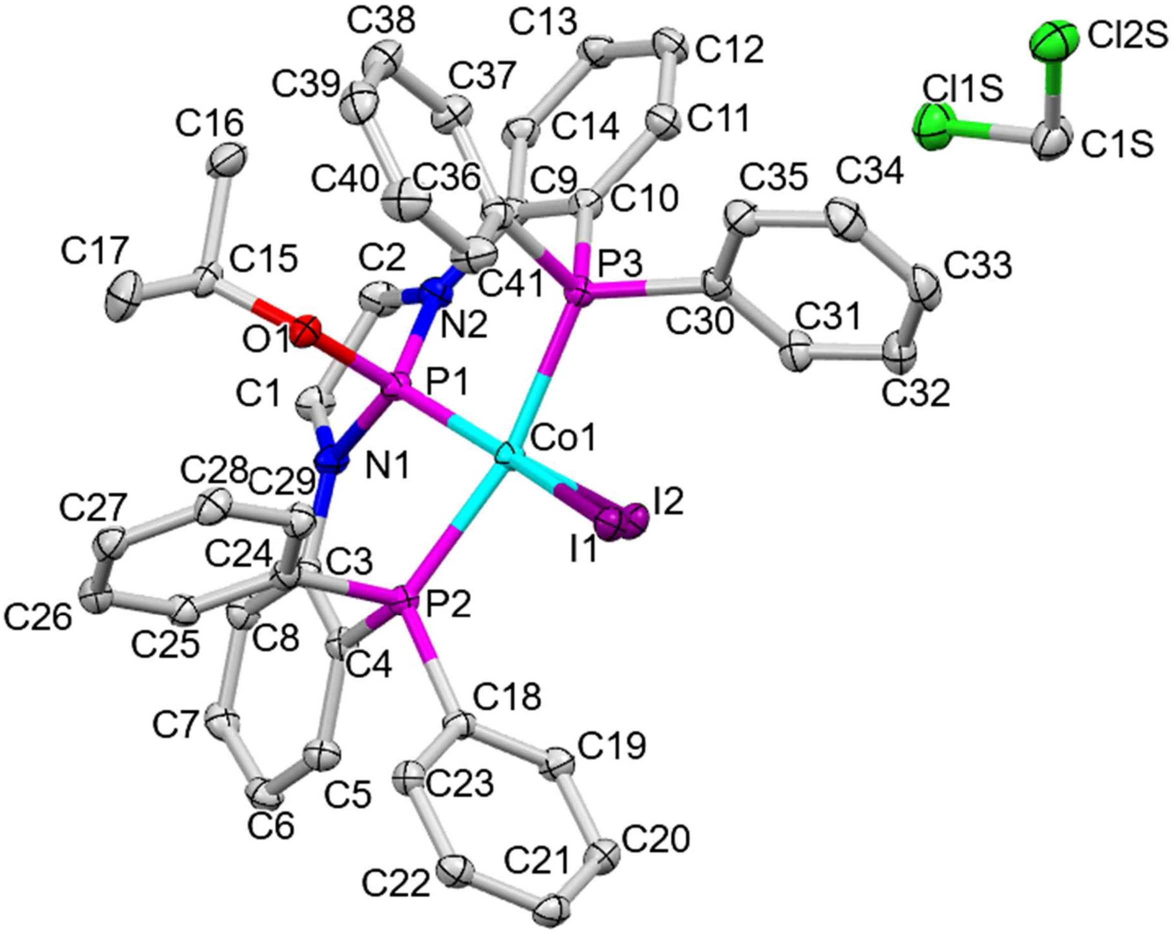

Figure S165. Fully labelled displacement ellipsoid representation of (PP^{OiPr}P)CoI_2 (**16**).

Data Collection and Refinement Details for 16.

The single crystal X-ray diffraction studies were carried out on a Bruker Kappa Photon III CPAD diffractometer equipped with Mo K_α radiation (λ = 0.71073 Å). A 0.121 x 0.091 x 0.076 mm piece of an orange block was mounted on a MiTeGen MicroMount with Paratone 24EX oil. Data were collected in a nitrogen gas stream at 100(2) K using ϕ and ϖ scans. Crystal-to-detector distance was 60 mm using variable exposure time (1s-2s) depending on θ with a scan width of 1.0°. Data collection was 99.3% complete to 25.00° in θ (0.83 Å). A total of 91523 reflections were collected covering the indices, $-13 \leq h \leq 13$, $-17 \leq k \leq 17$, $-22 \leq l \leq 22$. 10002 reflections were found to be symmetry independent, with a R_{int} of 0.0331. Indexing and unit cell refinement indicated a primitive, triclinic lattice. The space group was found to be *P*-1. The data were integrated using the Bruker SAINT software program and scaled using the SADABS

software program. Solution by dual-space method (SHELXT) produced a complete phasing model for refinement.

All nonhydrogen atoms were refined anisotropically by full-matrix least-squares (SHELXL-2014). All hydrogen atoms were placed using a riding model. Their positions were constrained relative to their parent atom using the appropriate HFIX command in SHELXL-2014. Crystallographic data are summarized in Table S4.

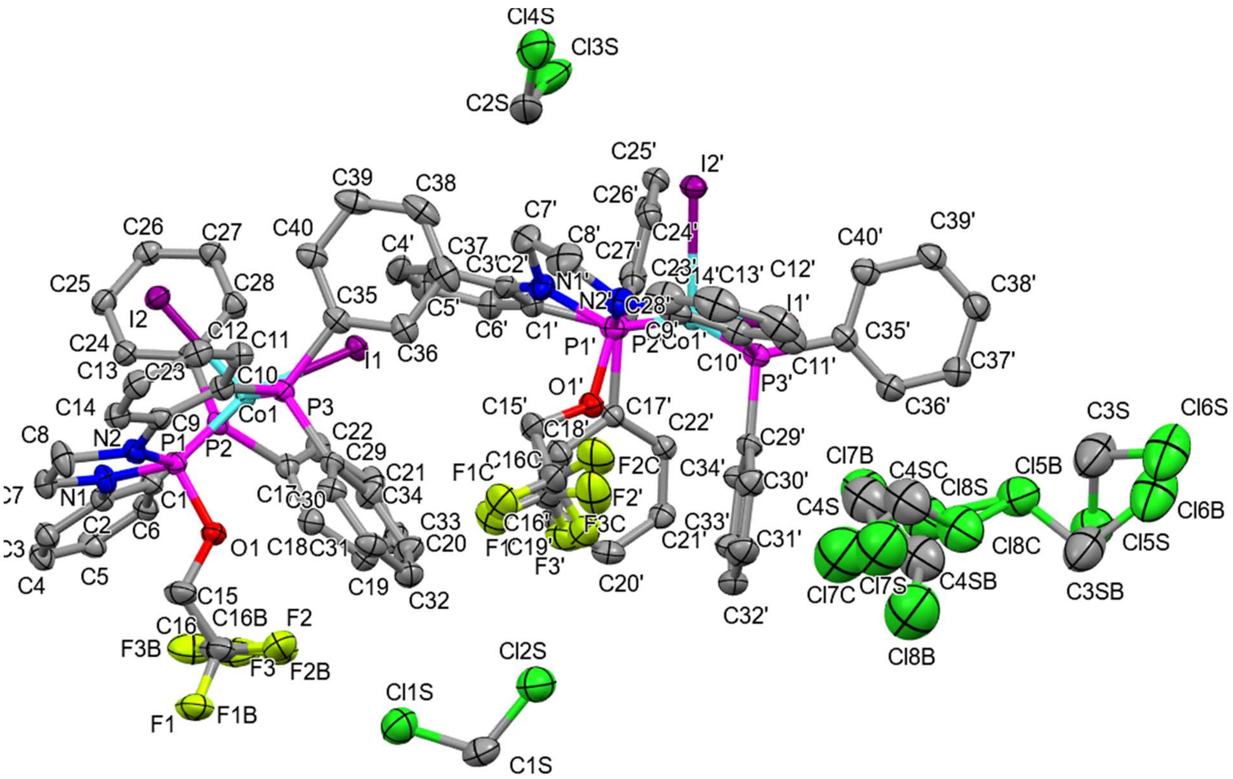

Figure S166. Fully labelled displacement ellipsoid representation of (PP^{OCH2CF3}P)CoI_2 (**17**).

Data Collection and Refinement Details for 17.

The single crystal X-ray diffraction studies were carried out on a Bruker Kappa Photon III CPAD diffractometer equipped with Mo K_α radiation (λ = 0.71073 Å). A 0.162 x 0.101 x 0.064 mm piece of an orange block was mounted on a MiTeGen MicroMount with Paratone 24EX oil. Data were collected in a nitrogen gas stream at 100(2) K using ϕ and ϖ scans. Crystal-to-detector distance was 40 mm using variable exposure time (1s-3s) depending on θ with a scan width of 1.0°. Data collection was 99.8% complete to 25.00° in θ(0.83 Å). A total of 284067 reflections were collected covering the indices, $-19 \leq h \leq 19$, $-23 \leq k \leq 23$, $-23 \leq l \leq 24$. 20329 reflections were found to be symmetry independent, with a R_{int} of 0.0480. Indexing and unit cell refinement indicated a primitive, triclinic lattice. The space group was found to be *P*-1. The data were integrated using the Bruker SAINT software program and scaled using the SADABS software program. Solution by dual-space method (SHELXT) produced a complete phasing model for refinement.

All nonhydrogen atoms were refined anisotropically by full-matrix least-squares (SHELXL-2014). All hydrogen atoms were placed using a riding model. Their positions were constrained relative to their parent atom using the appropriate HFIX command in SHELXL-2014. Crystallographic data are summarized in Table S4.

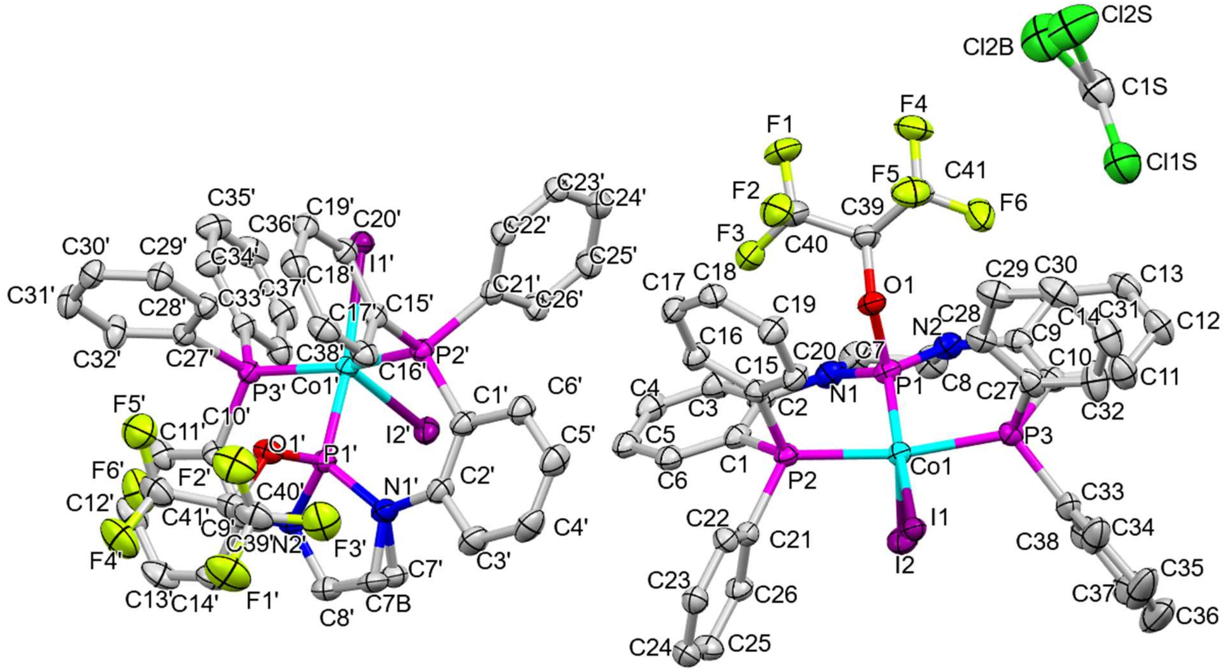

Figure S167. Fully labelled displacement ellipsoid representation of (PP$^{OCH(CF3)2}$P)CoI$_2$ (**18**).

Data Collection and Refinement Details for 18.

The single crystal X-ray diffraction studies were carried out on a Bruker Kappa Photon III CPAD diffractometer equipped with Mo K_α radiation (λ = 0.71073 Å). A 0.067 x 0.064 x 0.021 mm piece of an orange plate was mounted on a MiTeGen MicroMount with Paratone 24EX oil. Data were collected in a nitrogen gas stream at 100(2) K using ϕ and ϖ scans. Crystal-to-detector distance was 60 mm using variable exposure time (2s-15s) depending on θ with a scan width of 0.5°. Data collection was 99.9% complete to 25.00° in θ (0.83 Å). A total of 265261 reflections were collected covering the indices, $-16 \leq h \leq 16$, $-24 \leq k \leq 24$, $-28 \leq l \leq 28$. 22079 reflections were found to be symmetry independent, with a R_{int} of 0.0530. Indexing and unit cell refinement indicated a primitive, triclinic lattice. The space group was found to be *P*-1. The data were integrated using the Bruker SAINT software program and scaled using the SADABS software program. Solution by dual-space method (SHELXT) produced a complete phasing model for refinement.

All nonhydrogen atoms were refined anisotropically by full-matrix least-squares (SHELXL-2014). All hydrogen atoms were placed using a riding model. Their positions were constrained relative to their

parent atom using the appropriate HFIX command in SHELXL-2014. Due to unmodelable solvent disorder, OLEX2 solvent-mask was used to remove the electron density from the lattice due to the disordered solvent contribution. The solvent appeared to be dichloromethane. One void was found with approximately 89 electrons, which is consistent with 1 molecule of dichloromethane per asymmetric unit. Crystallographic data are summarized in Table S4.

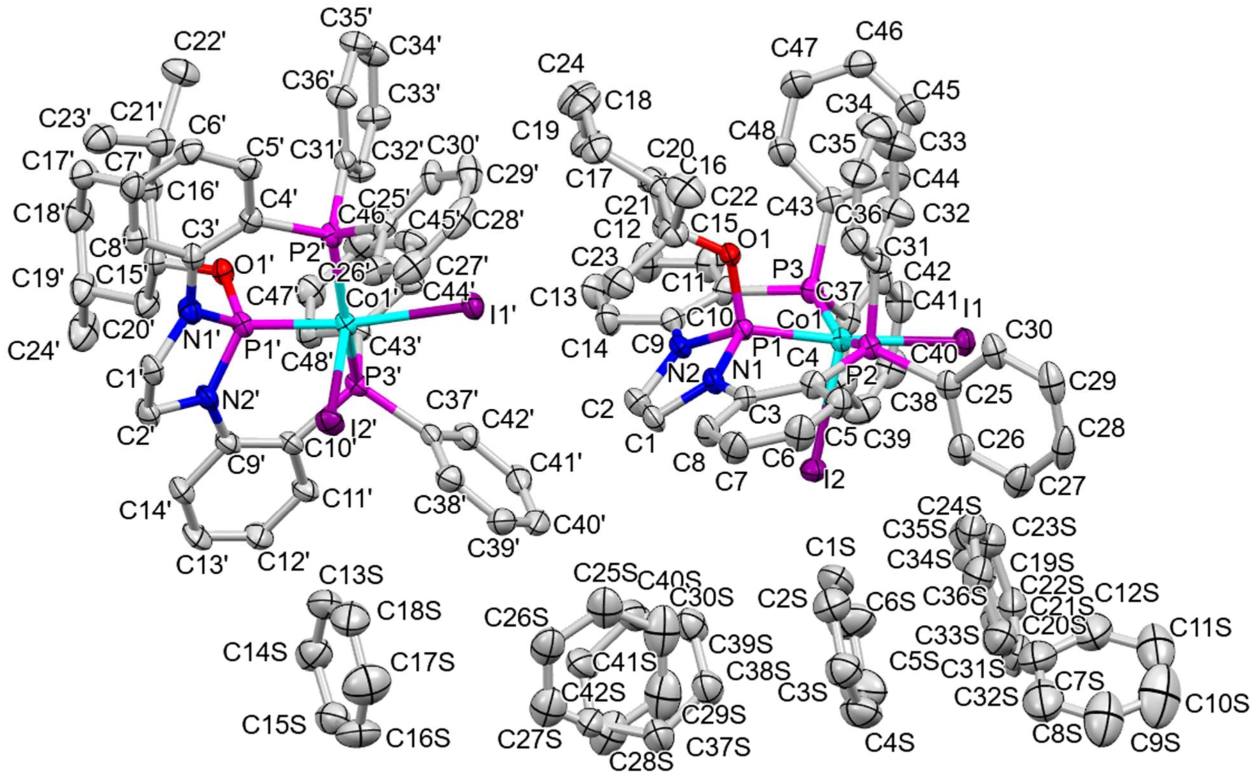

Figure S168. Fully labelled displacement ellipsoid representation of $(PP^{menthoxide}P)CoI_2$ (**19**).

Data Collection and Refinement Details for 19.

The single crystal X-ray diffraction studies were carried out on a Bruker Kappa Photon III CPAD diffractometer equipped with Mo K_α radiation (λ = 0.71073 Å). A 0.227 x 0.039 x 0.028 mm piece of an orange rod was mounted on a MiTeGen MicroMount with Paratone 24EX oil. Data were collected in a nitrogen gas stream at 100(2) K using ϕ and ϖ scans. Crystal-to-detector distance was 60 mm using variable exposure time (2s-5s) depending on θ with a scan width of 1.0°. Data collection was 99.9% complete to 25.00° in θ (0.83 Å). A total of 345528 reflections were collected covering the indices, $-23 \leq h \leq 33$, $-20 \leq k \leq 20$, $-24 \leq l \leq 25$. 23290 reflections were found to be symmetry independent, with a R_{int} of 0.0545. Indexing and unit cell refinement indicated a primitive, monoclinic lattice. The space group was found to be $P2_1$. The data were integrated using the Bruker SAINT software program and scaled using the SADABS software program. Solution by dual-space method (SHELXT) produced a complete phasing model for refinement.

All nonhydrogen atoms were refined anisotropically by full-matrix least-squares (SHELXL-2014). All hydrogen atoms were placed using a riding model. Their positions were constrained relative to their parent atom using the appropriate HFIX command in SHELXL-2014. The absolute stereochemistry of the molecule was established by anomalous dispersion using the Parson's method with a Flack parameter of -0.023(5). Crystallographic data are summarized in Table S5.

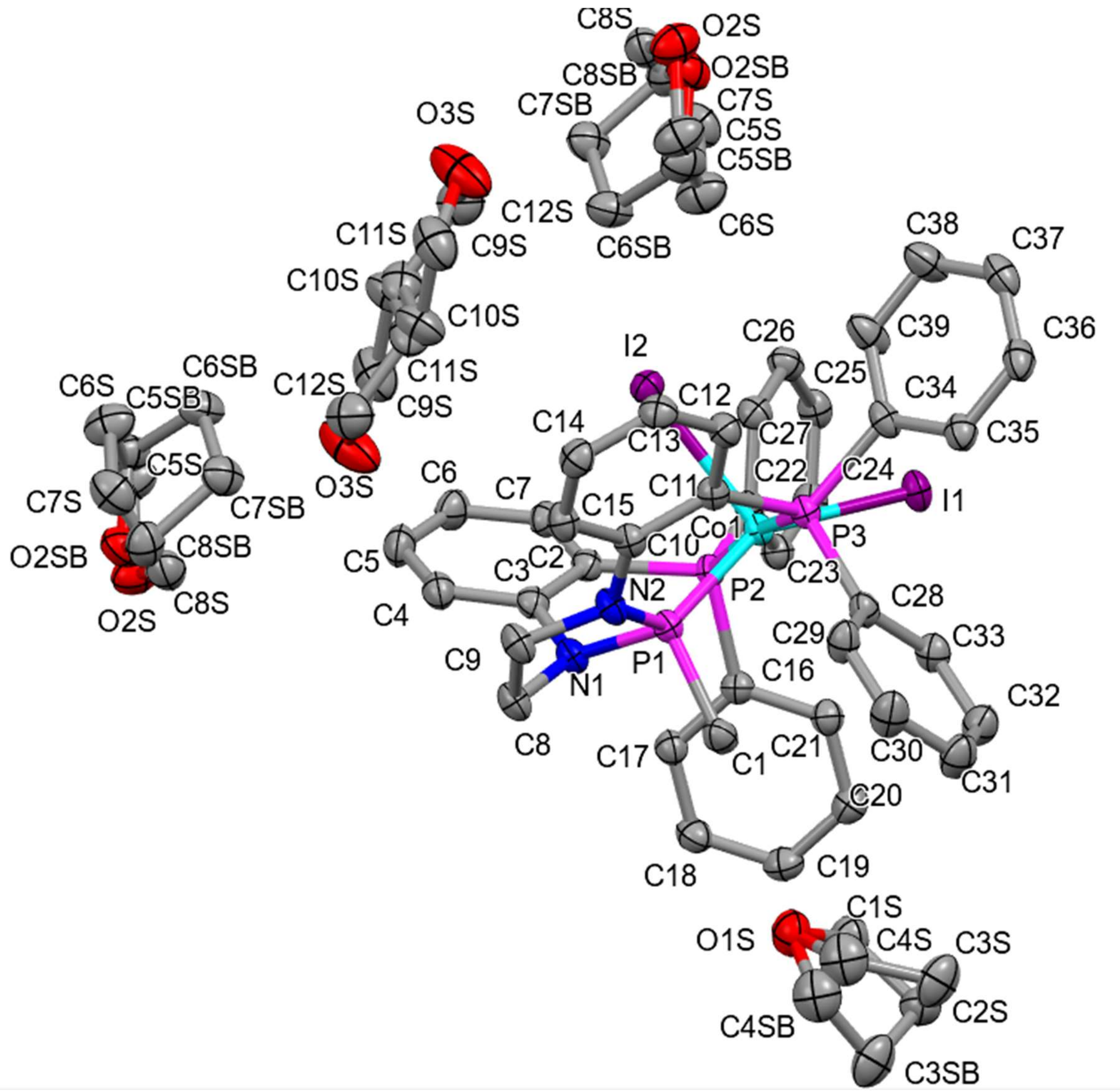

Figure S169. Fully labelled displacement ellipsoid representation of $(PP^{Me}P)CoI_2$ (**20**).

Data Collection and Refinement Details for 20.

The single crystal X-ray diffraction studies were carried out on a Bruker Kappa Photon III CPAD diffractometer equipped with Mo K_α radiation (λ = 0.71073 Å). A 0.227 x 0.071 x 0.057 mm piece of a green block was mounted on a MiTeGen MicroMount with Paratone 24EX oil. Data were collected in a nitrogen gas stream at 100(2) K using ϕ and ϖ scans. Crystal-to-detector distance was 60 mm using variable exposure time (1s-5s) depending on θ with a scan width of 1.0°. Data collection was 99.8% complete to 25.00° in θ (0.83 Å). A total of 106476 reflections were collected covering the indices, $-15 \leq h \leq 15$, $-17 \leq$

k ≤ 17, -20 ≤ l ≤ 20. 10106 reflections were found to be symmetry independent, with a R_{int} of 0.0317. Indexing and unit cell refinement indicated a primitive, triclinic lattice. The space group was found to be *P*-1. The data were integrated using the Bruker SAINT software program and scaled using the SADABS software program. Solution by dual-space method (SHELXT) produced a complete phasing model for refinement.

All nonhydrogen atoms were refined anisotropically by full-matrix least-squares (SHELXL-2014). All hydrogen atoms were placed using a riding model. Their positions were constrained relative to their parent atom using the appropriate HFIX command in SHELXL-2014. Crystallographic data are summarized in Table S5.

Table S3. Crystal data and structure refinement details for **13**•1.5(CH_2Cl_2),0.25(Et_2O), **14**, and **15**•2.5(CH_2Cl_2).

	**13**•1.5(CH_2Cl_2), 0.25(Et_2O)	**14**	**15**•2.5(CH_2Cl_2)
CCDC No.	2543406	2543404	2543403
Empirical formula	$C_{44.75}H_{48}Cl_{3.50}CoI_2N_3O_{0.25}P_3$	$C_{44}H_{46}CoI_2N_3P_3$	$C_{42.50}H_{42}Cl_5CoI_2N_2OP_3$
Molecular formula	$C_{42}H_{42}CoI_2N_3P_3$,1.75(CH_2Cl_2), 0.25($C_4H_{10}O$)	$C_{44}H_{46}CoI_2$ N3 P_3	$C_{40}H_{37}CoI_2N_2OP_3$, 2.5(CH_2Cl_2)
Formula weight	1161.57	1022.48	1179.67
Temperature	100.0 K	100.0 K	100.0 K
Wavelength	0.71073 Å	0.71073 Å	0.71073 Å
Crystal system	Monoclinic	Monoclinic	Triclinic
Space group	$P2_1$/n	$P2_1$/n	*P*-1
a	10.0028(4) Å	10.3893(3) Å	12.9297(9) Å
b	20.0603(7) Å	23.4039(7) Å	13.0020(10) Å
c	22.9347(9) Å	16.7139(5) Å	16.2787(12) Å
α	90°	90°	71.117(2)°
β	93.3320(10)°	92.4450(10)°	86.690(2)°
γ	90°	90°	62.158(2)°
Volume	4594.3(3) $Å^3$	4060.3(2) $Å^3$	2275.3(3) $Å^3$
Z	4	4	2
Density (calculated)	1.679 mg/cm^3	1.673 mg/cm^3	1.722 mg/cm^3
Absorption coefficient	2.062 mm^{-1}	2.098 mm^{-1}	2.169 mm^{-1}
F(000)	2308	2036	1164
Crystal size	0.221 x 0.102 x 0.063 mm^3	0.099 x 0.078 x 0.033 mm^3	0.203 x 0.135 x 0.041 mm^3
Crystal color, habit	red plate	orange plate	red block
θ range for data collection	2.700 to 28.366°	2.125 to 27.131°	2.877 to 48.423°
Reflections collected	117476	108233	110477
Independent reflections	11443 [R(int) = 0.0383, R(sigma) = 0.0191]	8978 [R(int) = 0.0516, R(sigma) = 0.0234]	11290 [R(int) = 0.0305, R(sigma) = 0.0163]
Completeness to θ = 25.000°	99.8 %	99.9%	99.0%
Absorption correction	Semi-empirical from equivalents	Semi-empirical from equivalents	Semi-empirical from equivalents
Max. and min. transmission	0.0479 and 0.0243	0.0462 and 0.0248	0.0962 and 0.0655
Refinement method	Full-matrix least-squares on F^2	Full-matrix least-squares on F^2	Full-matrix least-squares on F^2
Data / restraints / parameters	11443 / 68 / 585	8978 / 3 / 492	11290 / 9 / 524
Goodness-of-fit on F^2	1.083	1.169	1.071
Final R indices [I>2sigma(I)]	R1 = 0.0497, wR2 = 0.1806	R1 = 0.0297, wR2 = 0.0621	R1 = 0.0191, wR2 = 0.0462
R indices (all data)	R1 = 0.0526, wR2 = 0.1834	R1 = 0.0370, wR2 = 0.0647	R1 = 0.0217, wR2 = 0.0479
Extinction coefficient	n/a	n/a	n/a
Largest diff. peak and hole	1.315 and -1.325 $Å^{-3}$	0.608 and -0.465 $Å^{-3}$	0.727 and -0.338 $Å^{-3}$

Table S4. Crystal data and structure refinement details for **16**•(CH_2Cl_2), **17**•2(CH_2Cl_2), and **18**•(CH_2Cl_2).

	**16**•(CH_2Cl_2)	**17**•2(CH_2Cl_2)	**18**•(CH_2Cl_2)
CCDC No.	2543402	XXXXX	2543407
Empirical formula	$C_{42}H_{41}Cl_2CoI_2N_2OP_3$	$C_{42}H_{38}Cl_4CoF_3I_2N_2OP_3$	$C_{42}H_{35}Cl_2CoF_6I_2N_2OP_3$
Molecular formula	$C_{41}H_{39}CoI_2N_2OP_3$,CH_2Cl_2	$C_{40}H_{34}CoF_3I_2N_2OP_3$, 2($CH_2Cl_2$)	$C_{41}H_{33}CoF_6I_2N_2OP_3$, 0.5($CH_2Cl_2$), 0.5[$CH_2Cl_2$]
Formula weight	1066.31	1191.18	1174.26
Temperature	100.0 K	100.0 K	100.0 K
Wavelength	0.71073 Å	0.71073 Å	0.71073 Å
Crystal system	Triclinic	Triclinic	Triclinic
Space group	*P*-1	*P*-1	*P*-1
a	10.4036(8) Å	15.0982(7) Å	12.2510(7) Å
b	12.9116(11) Å	18.4485(9) Å	18.5286(10) Å
c	17.0580(13) Å	18.7473(7) Å	21.1729(11) Å
α	105.409(2)°	72.090(2)°	73.436(2)°
β	92.426(2)°	68.0780(10)°	75.865(2)°
γ	111.521(2)°	87.790(2)°	78.208(2)°
Volume	2029.9(3) $Å^3$	4592.3(4) $Å^3$	4420.0(4) $Å^3$
Z	2	4	4
Density (calculated)	1.745 mg/cm^3	1.723 mg/cm^3	1.765 mg/cm^3
Absorption coefficient	2.230 mm^{-1}	2.103 mm^{-1}	2.076 mm^{-1}
F(000)	1054	2340	2300
Crystal size	0.121 x 0.091 x 0.076 mm^3	0.162 x 0.101 x 0.064 mm^3	0.067 x 0.064 x 0.021 mm^3
Crystal color, habit	orange block	orange block	orange plate
θ range for data collection	2.130 to 28.284°	1.946 to 27.159°	2.048 to 28.373°
Reflections collected	91523	284067	265261
Independent reflections	10002 [R(int) = 0.0331, R(sigma) = 0.0243]	20329 [R(int) = 0.0480, R(sigma) = 0.0173]	22079 [R(int) = 0.0530, R(sigma) = 0.0259]
Completeness to θ = 25.000°	99.3%	99.8%	99.9%
Absorption correction	Semi-empirical from equivalents	Semi-empirical from equivalents	Semi-empirical from equivalents
Max. and min. transmission	0.0962 and 0.0698	0.0463 and 0.0253	0.0479 and 0.0246
Refinement method	Full-matrix least-squares on F^2	Full-matrix least-squares on F^2	Full-matrix least-squares on F^2
Data / restraints / parameters	10002 / 0 / 480	20329 / 204 / 1138	22079 / 7 / 1056
Goodness-of-fit on F^2	1.035	1.057	1.021
Final R indices [I>2sigma(I)]	R1 = 0.0177, wR2 = 0.0407	R1 = 0.0287, wR2 = 0.0737	R1 = 0.0276, wR2 = 0.0621
R indices (all data)	R1 = 0.0191, wR2 = 0.0416	R1 = 0.0357, wR2 = 0.0804	R1 = 0.0406, wR2 = 0.0673
Extinction coefficient	n/a	n/a	n/a
Largest diff. peak and hole	0.473 and -0.327 $Å^{-3}$	1.423 and -1.656 $Å^{-3}$	0.878 and -0.664 $Å^{-3}$

Table S5. Crystal data and structure refinement details for **19**•2.5(C_6H_6) and **20**•2.5(THF).

	**19**•2.5(C_6H_6)	**20**•2.5(THF)
CCDC No.	2543409	2543405
Empirical formula	$C_{63}H_{66}CoI_2N_2OP_3$	$C_{49}H_{55}CoI_2N_2O_{2.50}P_3$
Molecular formula	$C_{48}H_{51}CoI_2N_2OP_3$, 2.5(C_6H_6)	$C_{39}H_{35}CoI_2N_2P_3$, 2.5(C_4H_8O)
Formula weight	1272.81	1117.59
Temperature	100.0 K	100.0 K
Wavelength	0.71073 Å	0.71073 Å
Crystal system	Monoclinic	Triclinic
Space group	$P2_1$	*P*-1
a	18.7673(7)) Å	12.3681(6) Å
b	16.7796(7) Å	13.2655(6) Å
c	20.0754(7) Å	15.8614(8) Å
α	90°	92.079(2)°
β	115.7950(10)°	103.271(2)°
γ	90°	114.4200(10)°
Volume	5692.0(4) Å^3	2280.87(19) Å^3
Z	4	2
Density (calculated)	1.485 mg/cm^3	1.627 mg/cm^3
Absorption coefficient	1.514 mm^{-1}	1.878 mm^{-1}
F(000)	2576	1122
Crystal size	0.227 x 0.039 x 0.028 mm^3	0.227 x 0.071 x 0.057 mm^3
Crystal color, habit	orange rod	green block
θ range for data collection	1.976 to 26.410°	1.989 to 27.227°
Reflections collected	345528	106479
Independent reflections	23290 [R(int) = 0.0545, R(sigma) = 0.0226]	10106 [R(int) = 0.0317, R(sigma) = 0.0159]
Completeness to θ = 25.000°	99.9 %	99.8 %
Absorption correction	Semi-empirical from equivalents	Semi-empirical from equivalents
Max. and min. transmission	0.0932 and 0.0680	0.0463 and 0.0252
Refinement method	Full-matrix least-squares on F^2	Full-matrix least-squares on F^2
Data / restraints / parameters	23290 / 145 / 1401	10106 / 20 / 612
Goodness-of-fit on F^2	1.068	1.049
Final R indices [I>2sigma(I)]	R1 = 0.0237, wR2 = 0.0546	R1 = 0.0200, wR2 = 0.0501
R indices (all data)	R1 = 0.0295, wR2 = 0.0586	R1 = 0.0236, wR2 = 0.0532
Extinction coefficient	n/a	n/a
Largest diff. peak and hole	0.618 and -0.528 Å^{-3}	0.653 and -0.293 Å^{-3}

DFT Calculations of 21-29

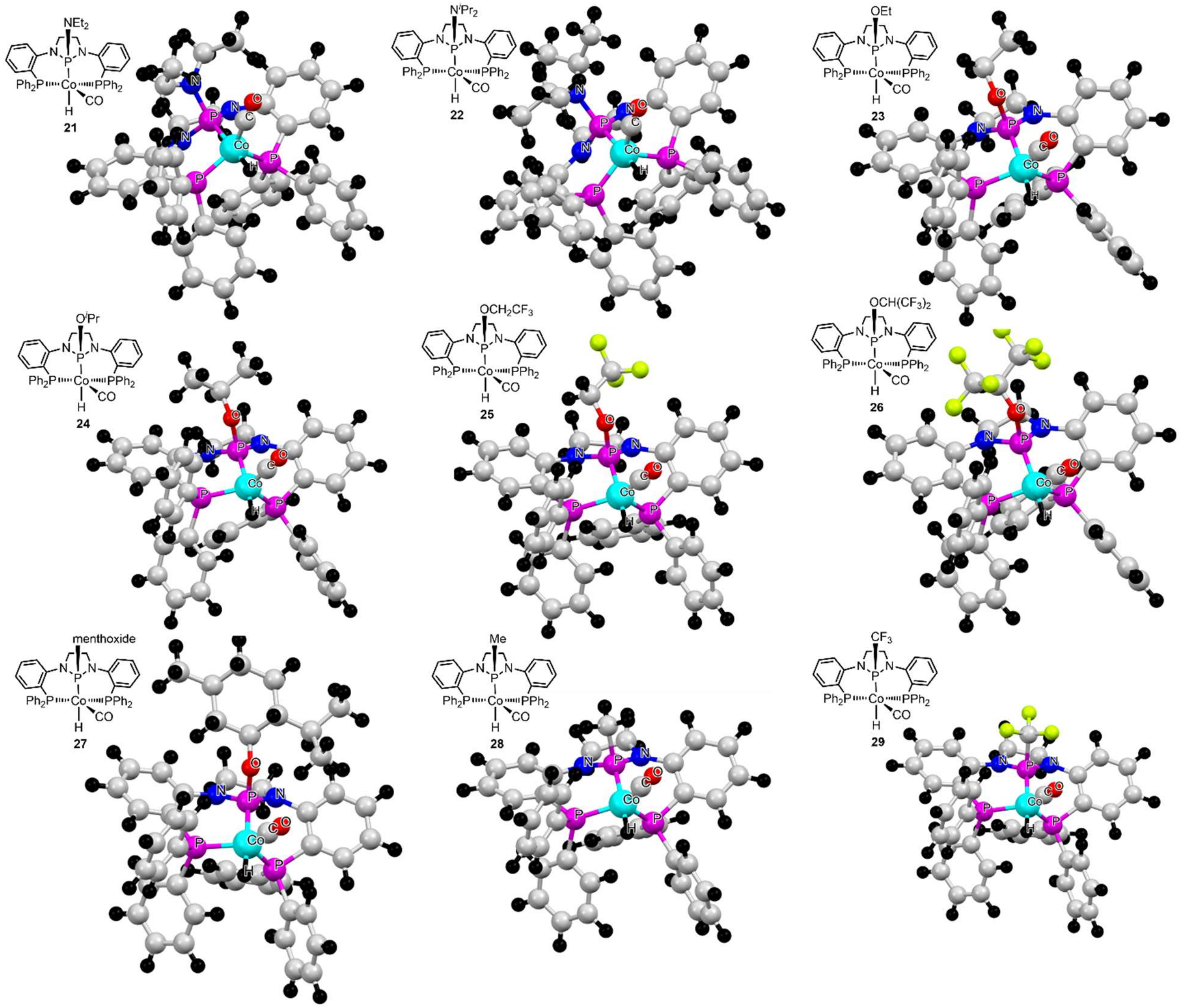

Figure S170. DFT-optimized geometries of $(PP^RP)Co(H)(CO)$ compounds (R = NEt_2 (**21**), N^iPr_2 (**22**), OEt (**23**), O^iPr (**24**), OCH_2CF_3 (**25**), $OCH(CF_3)_2$ (**26**), menthoxide (**27**), Me (**28**), CF_3 (**29**)).

Table S6. Selected natural charges and Wiberg bond indices (WBIs) calculated for **21**-**29**.

**Compound**	**Natural charge**		**Wiberg bond index (WBI)**	
	**Co**	**P**	**Co-P$_{central}$**	**Co-P$_{sidearm}$**
(PP^{NEt2}P)Co(CO)(H) (**21**)	-1.44	1.79	1.03	0.84, 0.89
(PP$^{N\textit{i}Pr2}$P)Co(CO)(H) (**22**)	-1.37	1.74	1.00	0.82, 0.89
(PP^{OEt}P)Co(CO)(H) (**23**)	-1.45	1.87	1.05	0.86, 0.88
(PP$^{O\textit{i}Pr}$P)Co(CO)(H) (**24**)	-1.45	1.87	1.05	0.86, 0.88
(PP^{OCH2CF3}P)Co(CO)(H) (**25**)	-1.45	1.85	1.08	0.85, 0.88
(PP$^{OCH(CF3)2}$P)Co(CO)(H) (**26**)	-1.45	1.85	1.08	0.85, 0.88
(PP^{menthoxide}P)Co(CO)(H) (**27**)	-1.48	1.87	1.05	0.86, 0.89
(PP^{Me}P)Co(CO)(H) (**28**)	-1.44	1.60	1.04	0.84, 0.89
(PP^{CF3}P)Co(CO)(H) (**29**)	-1.47	1.51	1.07	0.85, 0.88
